# Regioselective approach to colchiceine tropolone ring functionalization at C(9) and C(10) yielding new anticancer hybrid derivatives containing heterocyclic structural motifs

**DOI:** 10.1080/14756366.2022.2028782

**Published:** 2022-01-24

**Authors:** Krystian Pyta, Natalia Skrzypczak, Piotr Ruszkowski, Franz Bartl, Piotr Przybylski

**Affiliations:** a Faculty of Chemistry, Adam Mickiewicz University, Poznan, Poland; b Department of Pharmacology, Poznan University of Medical Sciences, Poznan, Poland; c Lebenswissenschaftliche Fakultät, Institut für Biologie, Biophysikalische Chemie Humboldt-Universität zu Berlin Invalidenstraße 42, Berlin, Germany

**Keywords:** Colchiceine tautomers, click, heck reaction, tubulin inhibitors, anticancer

## Abstract

The influence of base type, temperature, and solvent on regioselective C(9)/C(10) “*click*” modifications within the tropolone ring of colchiceine (**2**) is investigated. New ether derivatives of **2**, bearing alkyne, azide, vinyl, or halide aryl groups enable assembly of the alkaloid part with heterocycles or important biomolecules such as saccharides, geldanamycin or AZT into hybrid scaffolds by dipolar cycloaddition (CuAAC) or Heck reaction. Compared to colchicine (**1**) or colchiceine (**2**), ether congeners, as e.g. **3e** [IC_50_s_(_**_3e)_** ∼ 0.9 nM], show improved or similar anticancer effects, whereby the bulkiness of the substituents and the substitution pattern of the tropolone proved to be essential. Biological studies reveal that expanding the ether arms by terminal basic heterocycles as quinoline or pyridine, decreases the toxicity in HDF cells at high anticancer potency (IC_50_s ∼ 1–2 nM). Docking of ether and hybrid derivatives into the colchicine pocket of α_GTP_/β tubulin dimers reveals a relationship between the favourable binding mode and the attractive anticancer potency.

## Graphical Abstract

**Figure:**
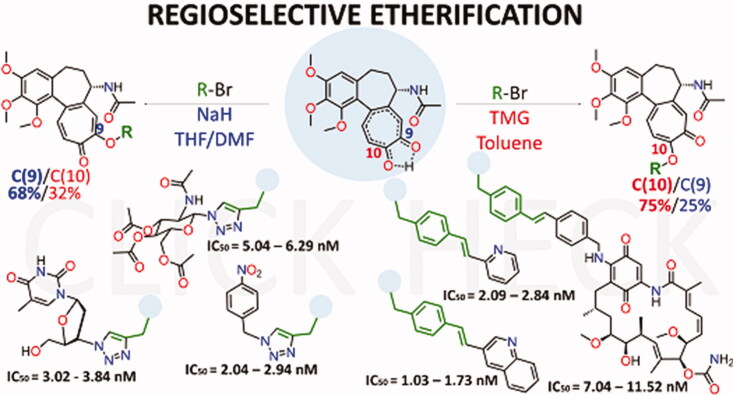

## Introduction

**Figure 1.**
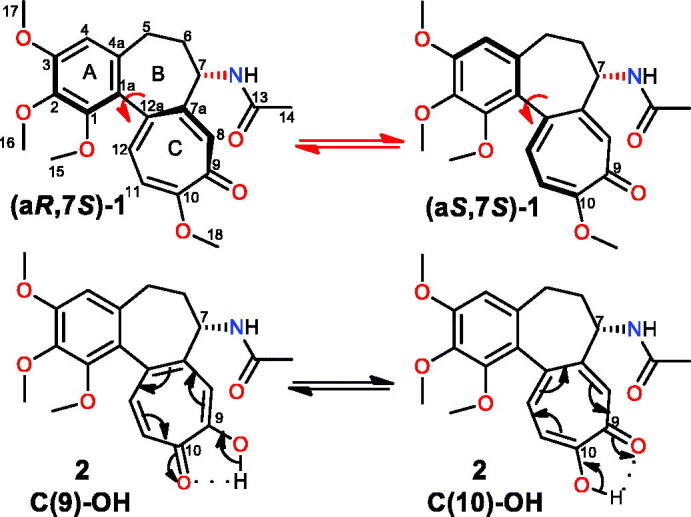
Structures of colchicine (**1**) and colchiceine (**2**) together with the atom numbering. Atropisomerization (top) and tautomerization (bottom) processes within colchicine (**1**) and colchiceine (**2**) scaffolds, respectively.

Colchicine (**1**, Figure 1), showing anticancer and other useful biological effects^1^^,^^2^, is a natural tropolone alkaloid, and as the other natural tropolones, is produced by autumn crocus (*Colchicum autumnale*)^3–9^. Its metabolite called colchiceine (**2**, Figure 1) also exhibits, albeit lower, anticancer activity, at the expense of increased antifungal properties, as compared to **1**^10^. In the structure of **2**, due to a possible rotation around the single bond C(1a)–C(12a), two diastereomeric forms can exist, similarly as for **1** (Figure 1). Furthermore, H-bonding between the C=O and OH groups within the tropolone of **2** contributes to an equilibrium between the C(9)-OH and C(10)-OH keto-enol tautomeric forms (Figure 1)^11^. Asymmetric total syntheses of colchiceine, β-lumicolchicine and allocolchicinoid derivative were performed by Liu et al.^12^ In order to improve anticancer potency and to decrease the toxic effects of colchicine, its transformations were mainly performed at C(10), C(7), and C(4) or *via* destruction of the tropolone ring^13–22^. Another type of modifications of colchiceine scaffold was the formation of an extra ring, fused with the C-ring of the parent alkaloid *via* different approaches^23–29^. The synthetic challenges of regioselective functionalization of hydroxyl group within the tropolone of **2** and the other troponoid systems were undertaken in the past, however with different outcomes^11^^,^^30^.

Hybrids of bioactive compounds of lower cytotoxicity towards normal cells, serve as drug delivery platforms, prodrugs, adjuvants, molecular probes, or agents active against drug-resistant cancer cell lines, parasites, or bacteria^22^^,^^31–37^. Colchiceine hybrids have been studied relatively rarely up to now. The amide-alkyl-ester bridge at C(7) was used to conjugate **1** with cobalamine in order to obtain tumour-targeted cytotoxin, whereas the presence of an amide linkage with a disulphide bond at C(7) enabled the formation of the thiocolchicine-podophyllotoxin hybrid^38^^,^^39^. Thiocolchicine conjugates bearing at C(7) long polyamide-lactone chains shown to be active in ovarian carcinoma line A2780 at IC_50_ ∼ 200 nM^40^. Dipolar cycloaddition and other modern synthetic methods as the Heck reaction yield chemically stable bonds and are worth considering at combining different bioactive blocks into a hybrid scaffold^41^^,^^42^. Dipolar cycloaddition reaction of CuAAC type was performed to obtain triazole-bridged hybrids of **1**, but exclusively at the C(7) position^43–47^. Recently, new colchicine-mimicking quinoline derivatives have been obtained which showed good tubulin polymerisation inhibitory effects as well as antiproliferative potency higher than that of **1**^48^. Earlier functionalization of the **1** framework with sulphur substituents at C(10) also suggested the influence of the bulkiness of the thioether arm on the anticancer effects^49^.

Colchicine interacts with tubulin units mainly *via* H-bond and hydrophobic interactions involving rings A and C^50^ and hence the incorporation of new functional arms at alternative C(9) or C(10) positions within the ring C should allow designing derivatives, which fit better to the molecular target. Therefore, here we obtain functionalised colchiceine-ether intermediates at C(9) and C(10) enabling the assembly of new colchicine conjugates, using dipolar cycloaddition of CuAAC type or Heck reactions. It should be mentioned that these intermediates, bearing ether portion at alternative sites of the tropolone ring, can be helpful at designing other-type conjugates in the future. Our regioselective approach to functionalise the colchicine scaffold at C(9)/C(10) allows systematic studying of the influence of substitution pattern of tropolone ring on the anticancer activity.

## Results and discussion

### Chemistry studies

**Figure 2.**
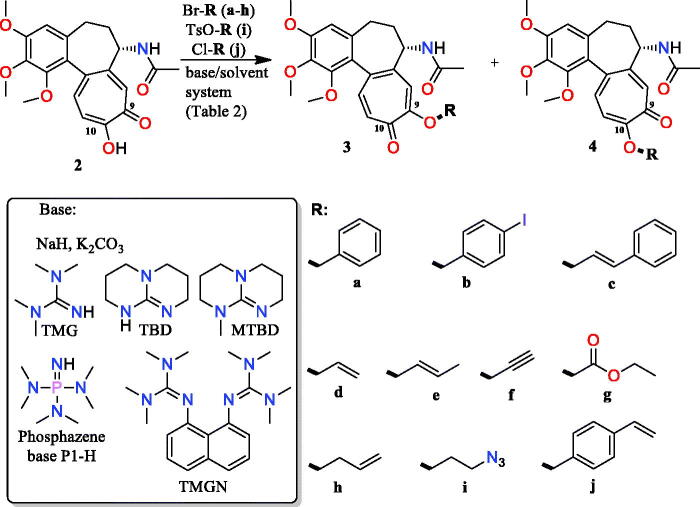
Regioselective etherification of **2** with different alkyl bromides, performed at C(9) – products **3a**–**j** and at C(10) – products **4a**–**j** of the tropolone.

In order to functionalise **2** towards linkers for the construction of hybrids, we performed S_N_2-type etherifications of the tropolone hydroxyl group (Figure 2, Tables 1 and 1S; Supplemental Material). Regarding the tautomerization process of **2**, the influence of base type, solvent, and substituent structure on the reaction course with the competitive formation of the two **3**- or **4**-type products (Figure 2) was tested. We focussed first on benzyl bromide as a reactant (Table 1). The most favourable conditions for the formation of the **3**-type products were found using the inorganic bases NaH or K_2_CO_3_. Under those conditions the privileged formation of C(9)-ether derivative occurred (∼70%). The presence of THF as a solvent contributes to the highest ratio of C(9)/C(10) products (Table 1). The change of the inorganic base into the organic one (MTBD) in THF evokes the lack of regioselectivity because a nearly equimolar mixture of **3** with **4** was formed. A similar result was obtained when acetonitrile, acetone, and DMF were used as solvents. Favourable formation of C(10) products [ratio C(9)/C(10) was 30/70] took place when MTBD was dissolved in aromatic-type solvents (xylene or toluene). The use of other organic bases such as TMG, phosphazene-base P_1_-H, TMGN, or TBD yielded predominantly the **4**-type product. The phosphazene base allowed to obtain a similar C(9)/C(10) ratio, as for MTBD, whereas the best regioselectivity towards the formation of the **4**-type product (75%) was achieved with TMG. Thus, as indicated above, the use of the inorganic base/THF system is beneficial for the formation of C(9) analogues (of **3**-type), whereas application of the organic base/toluene system alters the regioselectivity towards the favourable formation of C(10) analogues (of **4**-type). In the next step, the influence of the alkyl bromide structure on the etherification site within **2** was studied (Table 1). With the NaH/THF-DMF system the highest regioselectivity was observed for cinnamyl bromide (up to 77% **3c**, Table 1S; Supplemental Material). In turn, the use of propargyl bromide and ethyl bromoacetate limited the regioselectivity of the reaction. The use of the MTBD/toluene system with propargyl bromide and ethyl bromoacetate was quite beneficial (>70% of **4f**, **4g**) whereas the use of cinnamyl and 4-iodobenzyl bromides led to a less favourable formation of **4**-type product (Table S1).

**Table 1.** Comparison of S_N_2 reaction of **2** with benzyl bromide (BnBr) in different reaction conditions.

S_N_2 reactant	Base	Solvent	Time (h)	T (°C)	Yield (%) **3a** + **4a**	Ratio (%) of **3a**:**4a**
BnBr	NaH	DMF	4	70	87.3	59.8: 40.2
BnBr	NaH	THF/DMF (1:1)	4	70	83.5	67.1: 32.9
BnBr	NaH	THF/DMF (1:0.5)	4	70	95.1	68.3: 31.7
BnBr	K_2_CO_3_	Toluene	1	70	99.9	54.8: 45.2
BnBr	K_2_CO_3_	THF	1	66	78.1	66.1: 33.9
BnBr	K_2_CO_3_	Acetone	4	56	99.2	61.6: 38.4
BnBr	K_2_CO_3_	DMF	1	115	97.9	56.6: 43.4
BnBr	MTBD	Toluene	1	70	99.5	28.6: 71.4
BnBr	MTBD	ACN	1	70	92.8	52.6: 47.4
BnBr	MTBD	Acetone	4	56	92.6	50.0: 50.0
BnBr	MTBD	THF	1	66	94.5	50.9: 49.1
BnBr	MTBD	DMF	1	70	86.4	51.0: 49.0
BnBr	MTBD	Xylene	1	115	99.9	30.4: 69.6
BnBr	TMG	Toluene	1	115	99.1	25.3: 74.7
BnBr	P_1_-H	Toluene	1	70	99.1	29.8: 70.2
BnBr	TMGN	Toluene	16	70	99.9	36.0: 64.0
BnBr	TBD	Toluene	3	70	93.4	37.6: 62.4

**Figure 3.**
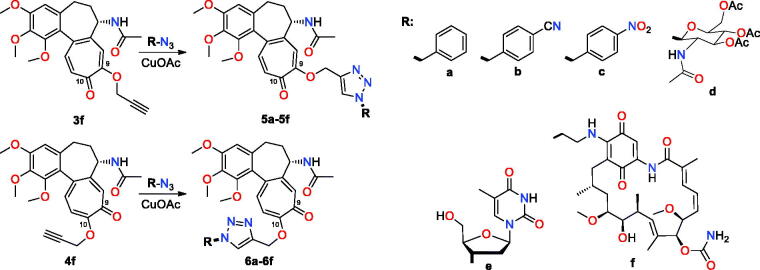
Structure of new colchiceine hybrids with arms at C(9) (**5a**–**5f**) and C(10) (**6a**–**6f**), obtained *via* Huisgen dipolar cycloaddition (CuAAC).

To explain the observed regioselectivity, DFT calculations (Figure S1) and FT-IR (Figure 2S and 3S) studies were performed. The calculated structures of complexes **2**-MTBD with involvement of C(9)-O^−^ or C(10)-O^−^ alkoxylates (Figure 1Sa) showed that significantly enhanced electron density at one of the oxygens occurs/at O(10)^−^/only when C(10)-O^−^ alkoxylate takes part in a medium strength H-bond with MTBD (distance D^…^A equal 2.74 Å; angle D-H^…^A equal 172°). In turn, the interaction between C(9)-O^−^ alkoxylate and MTBD contributes to the almost equal negative partial charge distribution between O(9) and O(10) atoms resulting in the almost equimolar formation of the C(9)- and C(10)-ether products. Moreover, a direct comparison of the energy (E) values for complexes **2**-MTBD (Figure S1) reveals that participation of C(9)-alkoxylate is less favourable than C(10)-alkoxylate in interaction with MTBD. Thus, the readily formation of C(10)-ether products **4a**–**j**, in the presence of MTBD, is explained by an increased electron density at oxygen O(10)^−^ within H-bonded complex **2**-MTBD. DFT calculations of complexes formed between C(9)-O^−^ or C(10)-O^−^ alkoxylates and Na^+^ were performed for the octahedral coordination sphere of Na^+^ (Figure 1Sb). In contrast to the C(10)-O^−^ alkoxylate complex with Na^+^, an analogous C(9)-O^−^ complex is stabilised involving the oxygen of the acetamide (Figure 1Sb). Greater discrimination in the negative partial charge distribution between oxygens O(9) and O(10) was noted when O(9)^−^ alkoxylate together with the oxygen of the acetamide is coordinated to the Na^+^ cation. This result explains the preferential formation of C(9)-ether products when the NaH/THF-DMF system was applied. The formation of H-bonded complex between **2** and MTBD is proved by the FT-IR spectra (Figure 2S and 3S). Protonation of the MTBD is reflected in the presence of ν(C = N^+^) and δ(N^+^-H) bands at 1624 and 1511 cm^−1^, respectively. In turn, the broad absorption band at ∼2750 cm^−1^ confirms the formation of an intermolecular N^+^-H^…−^O-C(10) H-bond between MTBD and alkoxylate of **2**.

**Figure 4.**
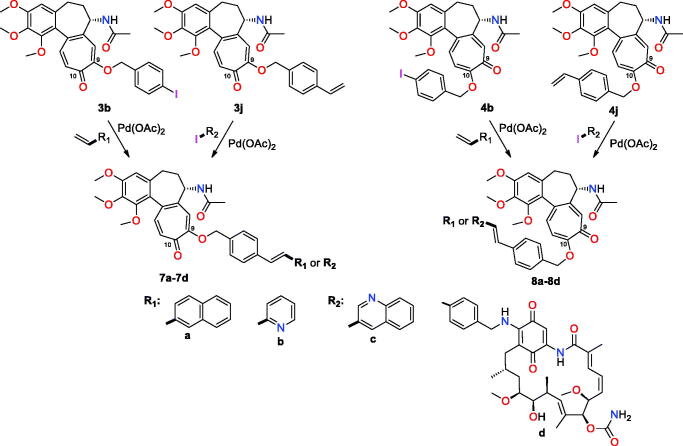
Structure of new colchiceine conjugates with arms at C(9) (**7a**–**7d**) and C(10) (**8a**–**8d**), obtained *via* Heck reaction.

In order to demonstrate the utility of C(9)- or C(10)-ether intermediates towards the formation of conjugates, those decorated with alkyne were subjected to Huisgen dipolar cycloaddition of CuAAC type (Figure 3). Alkyne intermediates **3f** and **4f** were used with benzyl, saccharide, and nucleoside azides as well as with azide congener of the ansamycin antibiotic – geldanamycin to afford triazole-bridged conjugates **5a**–**f** and **6a**–**f**, respectively (Figure 3). Reactions were performed predominantly in THF/methanol, whereas TBA/H_2_O was a convenient solvent system for the synthesis of hybrids **5f** and **6f**. In turn, the earlier obtained **3b**, **4b, 3j**, and **4j** ether products were used for assembling conjugates *via* Heck reactions (Figure 4). Structures of these hybrids were confirmed by NMR, FT-IR, and HR-MS (see Supplemental Material, exemplary ^1^H-^13^C HMBC couplings shown in Figure 4S). Irrespectively on the type of group installed at the colchicine scaffold (4-iodobenzyl, 4-vinylbenzyl) the ether-4-vinylbenzyl bridge was formed (products **7** and **8**, Figure 4). When ether-allyl reactants were used, the analogous Heck reactions did not yield the expected product and we observed decomposition of **3d** and **4d** into compound **2**.

### Anticancer and toxicity studies of new C(9) and C(10) ether intermediates, triazole and styryl hybrids of 2

Simple ether derivatives with substituents at C(9) **3a**–**3j** and at C(10) **4a**–**j** were tested in four cancer cell lines: SKBR-3, SKOV-3, PC-3, and U-87, and in healthy cell line HDF (Table 2). Analysis of the data in Table 2 shows that ether derivatives with a smaller substituent (**3d**–**3i** and **4d**–**4i**) are more active than those containing aromatic ring at the introduced part (**3a–c**, **3j**, and **4a**–**c**, **4j**), irrespectively on the substitution site [C(9) or C(10)]. The activities of derivatives with smaller substituents are comparable at nM level to the parent compound colchicine (**1**, Figure 1), despite their relatively high lipophilicities (ilogP >3, Table 2) and limited solubility in water (< 0.1 mg/mL). Compound **3e,** with crotyl substituent at C(9), exhibited the highest anticancer potency (IC_50_ = 0.94 − 0.98 nM). The activity of compound **3e** is even better than that of **1** (IC_50_ = 1.06 − 1.28 nM) in SKBR-3, SKOV-3, PC-3, whereas **1** and **3e** show almost the same potency towards U-87 cancer cell line (IC_50 (_**_3e_**_)_ = 0.95 nM; IC_50 (_**_1_**_)_= 0.94 nM). For the most biologically desired crotyl moiety at the tropolone ring, the C(9) substitution pattern was more beneficial than the C(10) one. Overall, considering ether derivatives with smaller substituents, substitution at C(9) favours better activity than substitution at C(10), except for those with the attached ester moieties (**3g** and **4g**). It should be mentioned that for derivatives comprising aromatic rings (**3a**–**c, 3j**, and **4a**–**c**, **4j**) an analogous relationship cannot be postulated. Expanding of C(9)- and C(10)-ether arms of **3f** and **4f** by the formation of triazole bridges (derivatives of **5** and **6** types, Figure 3), decreases the anticancer potency when compared to **3f** and **4f** (Table 2). As seen from Table 2, with the increasing bulkiness of the terminal substituent at the triazole portion, the anticancer potency markedly decreases. Hence, the benzyl-triazole hybrids **5a**–**c** and **6a**–**c**, with less bulky ends of the arm, are the most active ones (IC_50_s = 2.04 − 3.97 nM) among this group derivatives. In contrast to these derivatives, combining of colchiceine with geldanamycin into one scaffold *via* triazole linkage, resulted in a decreased anticancer activity, when referred to **1**, and enhanced potency, when referred to geldanamycin itself^52^. In turn, the result of the anticancer studies of **7**- and **8**-type derivatives is quite surprising (Table 2), taking into account the length and bulkiness of substituents introduced by the Heck reaction (Figure 4). Fusion of geldanamycin with colchiceine into one framework (**7d** and **8d**) *via E*-vinyl bridge was biologically slightly more favourable than *via* a triazole bridge (**5f** and **6f**). Derivatives, where the *E*-vinyl arms are terminated with a 2-naphthyl moiety (**7a** and **8a**), do not show anticancer activities close to **1,** similarly as was observed for the **7d** and **8d**. In turn, quinoline derivatives **7c** and **8c**, which are isostructural with **7a** and **8a**, showed attractive potencies (IC_50_ = 1.03 − 1.73 nM), close to potencies of the most active simple ether derivatives **3d**–**3i** and **4d**–**4i**, despite the presence of long and bulky arms at C(9) or C(10). Compound **8c** showed a very good anticancer potency together with slightly decreased toxicity when referred to **1** (Table 2). Moreover, heterocyclic hybrids **7b** and **8b** decorated with pyridine, showed markedly higher activities than the respective naphthyl derivatives **7a** and **8a**, together with lower toxicities (IC_50_^HDF^_(_**_7b_**_)_ = 5.11 nM; IC_50_^HDF^_(_**_8b_**_)_ = 4.83 nM) than those of reference alkaloids **1** (IC_50_^HDF^_(_**_1_**_)_ = 2.37 nM) or **2** (IC_50_^HDF^_(_**_2_**_)_ = 3.57 nM).

**Table 2.** Anticancer activities [IC_50_ (nM) ± SD] of **1**, **2**, **3a**–**3j**, **4a**–**4j**, **5a**–**5f**, **6a**–**6f**, **7a**–**7d** and **8a**–**8d** in SKBR-3, SKOV-3, PC-3, U-87 cells and toxicity (in Human Dermal Fibroblasts; HDF) [IC_50_ (nM) ± SD], and *c*logP, all data compared with those of other type standards as cytarabine (**C**), actinomycin D (**ActD**) and mitomycin C (**MitC**).

Compd.	SKBR-3	SKOV-3	PC-3	U-87	HDF	ilogP*
**1**	1.07 ± 0.05	1.28 ± 0.11	1.06 ± 0.03	0.94 ± 0.02	2.37 ± 0.15	3.28
**2**	1.04 ± 0.07	1.88 ± 0.02	1.37 ± 0.02	1.26 ± 0.04	3.57 ± 0.83	2.88
**3a**	2.04 ± 0.51	2.19 ± 0.07	2.27 ± 0.04	2.83 ± 0.06	6.39 ± 1.04	3.67
**3b**	2.55 ± 0.12	2.13 ± 0.40	2.94 ± 0.08	2.47 ± 0.91	3.94 ± 0.27	3.99
**3c**	3.09 ± 0.02	3.74 ± 0.18	3.11 ± 0.91	3.38 ± 0.05	5.24 ± 0.26	4.02
**3d**	1.09 ± 0.01	1.68 ± 0.09	1.05 ± 0.01	1.63 ± 0.04	2.83 ± 0.05	3.53
**3e**	0.94 ± 0.06	0.98 ± 0.02	0.96 ± 0.01	0.95 ± 0.01	2.13 ± 0.06	3.82
**3f**	1.37 ± 0.02	1.05 ± 0.07	1.05 ± 0.03	1.14 ± 0.01	2.83 ± 0.44	3.31
**3g**	1.31 ± 0.08	1.37 ± 0.01	1.62 ± 0.29	1.41 ± 0.03	2.72 ± 0.03	3.50
**3h**	1.04 ± 0.33	1.99 ± 0.07	1.53 ± 0.21	1.06 ± 0.03	1.84 ± 0.11	3.62
**3i**	1.32 ± 0.05	1.05 ± 0.02	1.43 ± 0.02	1.88 ± 0.05	2.85 ± 0.13	3.90
**3j**	4.19 ± 0.62	4.55 ± 0.07	4.59 ± 0.03	4.55 ± 0.06	5.38 ± 0.42	3.81
**4a**	2.01 ± 0.02	1.63 ± 0.03	2.26 ± 0.01	2.08 ± 0.01	4.12 ± 0.06	4.08
**4b**	2.77 ± 0.19	2.83 ± 0.26	2.41 ± 0.66	2.85 ± 0.03	4.12 ± 0.01	4.10
**4c**	3.46 ± 0.07	3.05 ± 0.03	3.19 ± 0.01	3.82 ± 0.22	4.94 ± 0.15	4.22
**4d**	1.73 ± 0.11	1.73 ± 0.07	1.71 ± 0.03	1.78 ± 0.03	2.39 ± 0.17	3.64
**4e**	1.97 ± 0.04	1.92 ± 0.11	1.98 ± 0.05	1.93 ± 0.02	2.66 ± 0.03	3.89
**4f**	1.48 ± 0.09	1.52 ± 0.02	1.46 ± 0.13	1.06 ± 0.04	1.85 ± 0.03	3.54
**4g**	1.28 ± 0.17	1.06 ± 0.22	1.15 ± 0.03	1.27 ± 0.01	2.05 ± 0.02	3.61
**4h**	1.69 ± 0.05	1.27 ± 0.16	1.22 ± 0.03	1.07 ± 0.12	2.71 ± 0.08	3.85
**4i**	1.38 ± 0.03	1.37 ± 0.09	1.49 ± 0.16	1.19 ± 0.03	2.58 ± 0.12	3.79
**4j**	3.22 ± 0.36	3.01 ± 0.04	3.05 ± 0.01	3.11 ± 0.03	5.17 ± 0.28	4.30
**5a**	3.07 ± 0.11	3.28 ± 0.06	3.21 ± 0.15	3.74 ± 0.19	5.28 ± 0.22	3.65
**5b**	2.07 ± 0.08	2.88 ± 0.47	2.13 ± 0.02	2.57 ± 0.13	4.92 ± 0.51	3.59
**5c**	2.18 ± 0.02	2.94 ± 0.71	2.09 ± 0.33	2.04 ± 0.08	3.12 ± 0.41	5.79
**5d**	6.29 ± 0.25	6.03 ± 0.19	6.39 ± 0.08	6.11 ± 0.26	7.99 ± 0.04	4.42
**5e**	3.71 ± 0.05	3.74 ± 0.03	3.51 ± 0.09	3.05 ± 0.01	6.22 ± 0.32	3.49
**5f**	8.91 ± 0.17	8.05 ± 0.58	8.38 ± 0.09	8.25 ± 0.11	10.36 ± 0.39	5.22
**6a**	2.33 ± 0.09	2.79 ± 0.07	2.04 ± 0.44	2.78 ± 0.05	3.88 ± 0.14	3.70
**6b**	3.06 ± 0.03	3.97 ± 0.12	3.15 ± 0.04	3.74 ± 0.06	5.83 ± 0.31	3.34
**6c**	2.88 ± 0.06	2.42 ± 0.11	2.05 ± 0.03	2.81 ± 0.18	4.08 ± 0.26	5.79
**6d**	5.21 ± 0.39	5.93 ± 0.12	5.04 ± 0.02	5.83 ± 0.15	7.29 ± 0.41	4.20
**6e**	3.02 ± 0.31	3.33 ± 0.11	3.50 ± 0.49	3.84 ± 0.02	5.18 ± 0.18	3.20
**6f**	17.40 ± 0.64	15.29 ± 0.31	17.84 ± 0.16	17.29 ± 0.35	18.03 ± 0.24	4.93
**7a**	8.02 ± 0.08	8.54 ± 0.71	8.61 ± 0.38	8.63 ± 0.14	8.19 ± 0.92	4.86
**7b**	2.09 ± 0.33	2.69 ± 0.02	2.45 ± 0.52	2.43 ± 0.12	5.11 ± 0.85	4.23
**7c**	1.51 ± 0.02	1.03 ± 0.09	1.52 ± 0.01	1.73 ± 0.09	2.16 ± 0.60	4.85
**7d**	7.22 ± 0.19	7.04 ± 0.15	7.49 ± 0.32	7.06 ± 0.44	9.04 ± 0.29	6.36
**8a**	6.30 ± 0.09	6.49 ± 0.25	6.03 ± 0.26	6.44 ± 0.07	8.72 ± 0.48	5.31
**8b**	2.77 ± 0.04	2.84 ± 0.03	2.63 ± 0.77	2.49 ± 0.05	4.83 ± 0.27	4.68
**8c**	1.05 ± 0.06	1.41 ± 0.02	1.47 ± 0.05	1.65 ± 0.03	2.77 ± 0.25	5.07
**8d**	11.37 ± 0.15	10.52 ± 0.05	10.97 ± 0.44	10.84 ± 0.74	17.94 ± 1.04	6.63
**C**	870 ± 10	990 ± 110	810 ± 10	850 ± 31	5940 ± 70	0.99
**ActD**	1140 ± 60	1140 ± 10	1170 ± 30	1610 ± 90	2810 ± 150	4.04
**MitC**	670 ± 10	610 ± 20	580 ± 110	650 ± 40	1380 ± 70	1.62

*-ilogP calculated by SwissADME [51]; **C:** cytarabine; **ActD:** actinomycin D; **MitC:** mitomycin C.

Overall, the toxicity of compounds **3**–**8** increases together with increasing anticancer effects (Table 2), irrespectively on the substitution pattern C(9)/C(10). Compound **3a** showed the most beneficial selectivity index (SI) (e.g. SI ∼3, for SKBR-3). Taking into account the ratio of anticancer activity relative to toxicity, the most interesting derivative is heterocyclic hybrid **8c**, since its potency is on the level IC_50_ ∼1 nM at SI ∼2.6 in SKBR-3 cells. The lowest toxic effect exhibited compound **3f**, where its SIs are ∼2.7 for SKOV-3 and PC-3 cancer cell lines. In turn, compound **4h** with a relatively small substituent at C(10) and attractive anticancer activity (average IC_50_ ∼1.5 nM), revealed the most beneficial SI ∼2.5 for U-87 cancer cell line. Unfortunately, the most potent derivative **3e** showed the highest toxicity in all studied cancer cell lines at SIs ∼ 2.2. In turn, the most promising derivatives, i.e. of the lowest toxicity in HDF cells and the most anticancer activity in SKOV-3, PC-3, and SKBR-3 cancer cell lines are intermediate **3f** and quinoline-based hybrid **8c**.

### Docking insight into SAR of colchiceine hybrids

**Figure 5.**
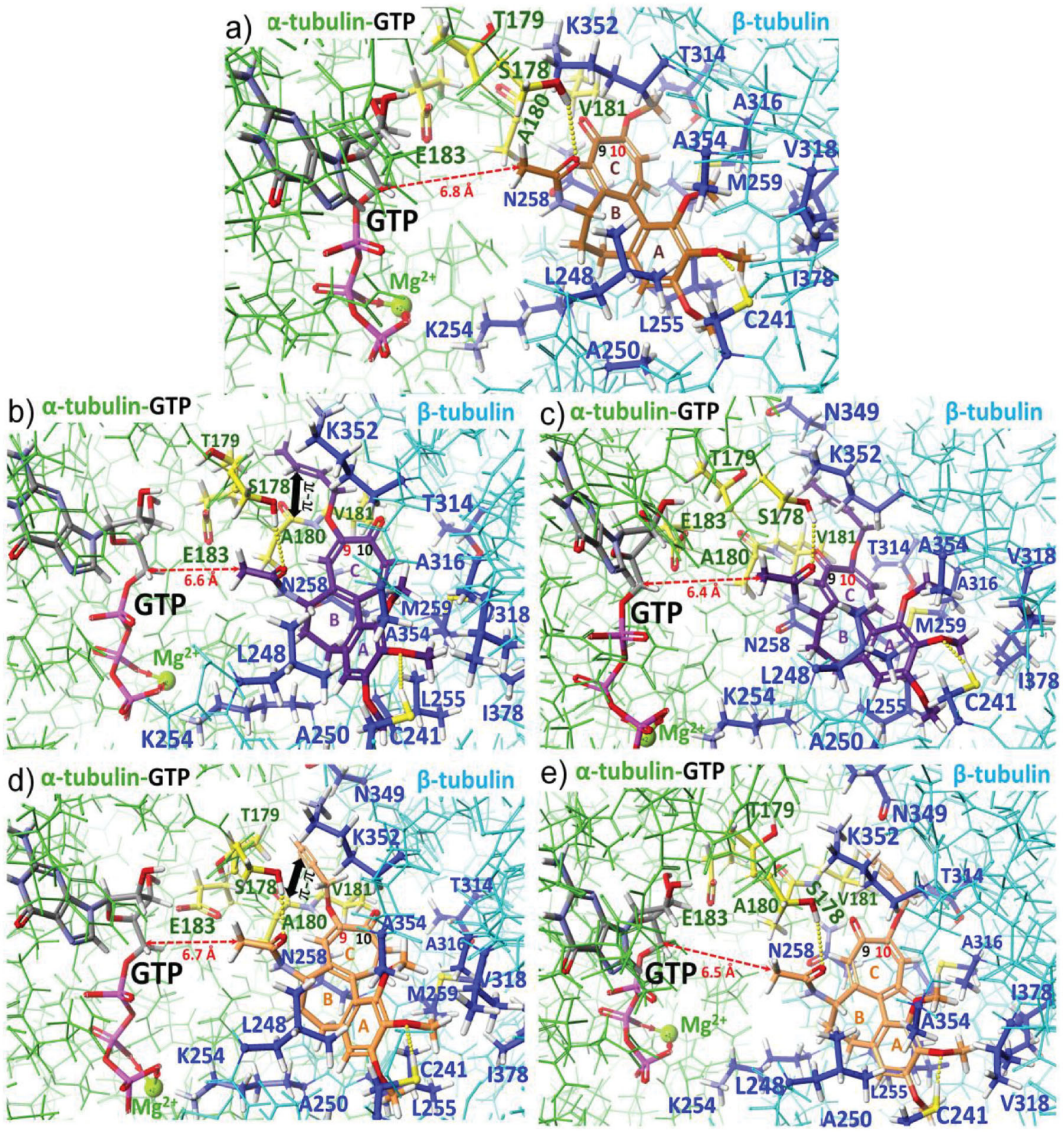
Docking models for colchiceine and its new derivatives with small ether moieties, attached to C(9) or C(10), at the binding pocket of tubulin dimer α_GTP_/β (PDB 1SA0) ^53^: (a) **2**-brown; (b) **3e** – dark violet, C(9)-substitution pattern (c) **4e** – dark violet, C(10)-substitution pattern; (d) **3f** – orange, C(9)-substitution pattern; and (e) **4f** – orange, C(10)-substitution pattern, optimised *via* MO-G PM6 semi-empirical method using MOZYME algorithm for huge molecules (*Scigress* package FJ. 2.6, 3.1.9, 2008–2019) ^54^. Tubulin units are distinguished by different colours: α-tubulin (green) and its key amino acids (yellow) and β-tubulin (pale blue) and its key amino acids (dark blue) whereas intermolecular interactions between new colchiceine derivatives and the key binding amino acids of α_GTP_/β dimer are marked by yellow dots.

**Figure 6.**
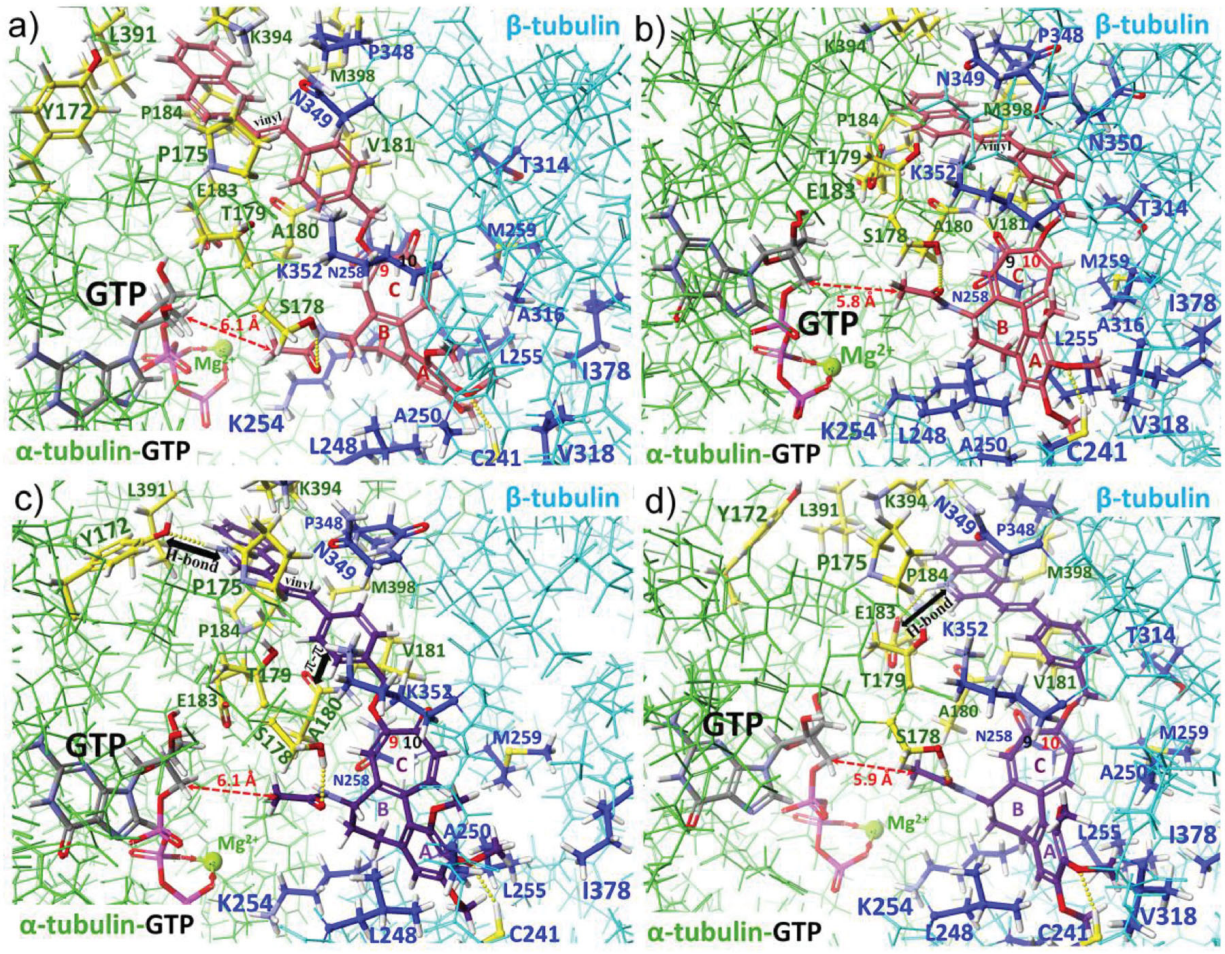
Docking models for colchicine (**1**) and colchiceine hybrids with extended ether arms, attached to C(9) or C(10), at the binding pocket of tubulin dimer α_GTP_/β (PDB 1SA0)^53^: (a) **7a** – rose, C(9)-substitution pattern; (b) **8a** – rose, C(10)-substitution pattern; (c) **7c** – violet, C(9)-substitution pattern; and (d) **8c** – violet, C(10)-substitution pattern; optimised *via* MO-G PM6 semi-empirical method using MOZYME algorithm for huge molecules (*Scigress* package 3.1.9, 2008–2019) ^54^. Tubulin units are distinguished by different colours: α-tubulin (green) and its key amino acids (yellow) and β-tubulin (pale blue) and its key amino acids (dark blue), intermolecular interactions between new colchiceine derivatives and the key binding amino acids of α_GTP_/β dimer are marked by yellow dots, distance to C5’ carbon atom of GTP is marked by the red dashed line.

To get a deeper insight into the SAR and in order to explain the observed differences in anticancer effects for colchiceine hybrids, docking studies into the binding site of colchicine (**1**), i.e. between dimeric α_GTP_/β tubulins, were performed (Table 2S, Figure 5, Figure 5S). Interactions between **1** and dimeric α_GTP_/β tubulins were also optimised (Figure 5a) for comparison. Compound **1** is stabilised between α_GTP_/β tubulin units (PDB 1SA0) ^53^, in the vicinity of the GTP binding site, mainly *via* hydrophobic interactions in the pocket formed by A180α, V181α, L248β, A250β, K254β, L255β, N258β, M259β, T314β, A316β, V318β, K352β and A354β and I378β and by two H-bonds with S178α (O-H) and C241β (S-H) as well as by a very weak interaction with N-H group of V181 (not marked in Figure 5a). In turn, the methoxy group at C(10) of **1** is stabilised in the binding pocket by hydrophobic interactions with T314β. Docking of the ether derivatives **3e** and **4e** at tubulin dimers revealed that substitution at C(9) with crotyl moiety is energetic more favourable than at C(10) (Table 2S). Furthermore, the binding energy of **3e** is more beneficial than for **1** (by ∼ 10 kcal/mol), which is in line with the observed trend for their anticancer activities. The replacement of the C(10)-methoxy group (in **1**) with a crotyloxy group (in **4e**) was less favourable for the binding energy with the target (Table 2S). The explanation of this result is a fact that with the changed substitution pattern from C(10) to C(9) one for the crotyloxy substituent (**3e**), extra stabilising interactions of the ether moiety at the binding pocket are realised, i.e. π-π stacking with the carbonyl group of A180α and hydrophobic contact to T179α. Analogous binding modes and relationship as for **3e** and **4e** are observed for derivatives with a small substituent as propargyloxy group, whereby differences between binding energies of **3f** and **4f** are lower (^C9^ΔH°_f_ – ^C10^ΔH°_f_ = ∼ 2 kcal/mol), which corresponds well with their similar anticancer activities in all studied cancer cell lines. An increase in the length of C(9) or C(10) ether substituents, as for the pairs **3a** and **3j** and **4a** and **4j**, contributes to a decrease in binding energy profit with the tubulins and could explain the lower potency of such derivatives. Thus, a general overview of the data collected in Table 2S related to **3**- and **4**-type derivatives suggests that compounds with less bulky substituents better fit into the pocket of α_GTP_β tubulin dimers than those with bigger ether substituents, whereby the C(9) substitution pattern seems to be more energetic favourable than the C(10) one. The worse fitting into the binding pocket within dimeric tubulins is also well reflected by the decreasing distance between the acetamide group (carbon atom of the methyl group) and C(5′) carbon atom of GTP caused by the change in the substitution pattern from C(9) to C(10) in the tropolone (Figures 5,6). An increase in the length and bulkiness of C(9) or C(10) ether-triazole arms, as for compounds **5b** and **6b**, makes the binding energies to tubulin dimers less favourable, if compared to their synthetic precursors **3f** and **4f** (Table 2S). Similar results were expected for Heck reaction products of **7**- and **8**-types, containing lengthy and bulky arms at C(9) or C(10). This is only true for the conjugate with the naphthalene moiety **7a** and **8a**, for which the lowest binding energy profits and the lowest anticancer effects were together noted (Tables 2 and 2S). Surprisingly, for hybrids **7c** and **8c** the binding energies to α_GTP_/β tubulins were close to those calculated for simple ether derivatives with small substituents such as **3f** and **4f**. This result may explain the high anticancer potency of **7c** and **8c** compared to those of **3**- and **4**-types, in the light of similar and high lipophilicities (ilogPs >3.3, Table 2) and limited water solubilities (<0.1 mg/mL) for all of them. The reason for better stabilisation of hybrids **7c** and **8c,** bearing quinoline-vinylbenzyloxy arms, at the binding pocket of α_GTP_/β dimer, is the formation of H-bonds between the terminal and protonated quinoline and the phenol group of Y172α (Figure 6c) or the carboxylate of E183α (Figure 6d), respectively. This favourable H-bonding along with hydrophobic stabilisation of the arms between prolines P175α and P184α partially compensate the loss in binding energy profit, due to the fitting of the lengthy and bulky arms of **7c** and **8c** into the cavity between the tubulin units.

Furthermore, the attractive anticancer potency of **7c** and **8c** may result from their basic properties reflected also in better water solubility and lower milogP in the protonated forms (milogP_neutral_ = 5.05 and milogP_protonated_ = 2.51, for **7c** and **8c**)^55^. The altered arrangement of the nitrogen of the heterocyclic moiety relative to the vinyl bridge in **7b** and **8b**, contributes to their lower binding energy profits to the target (ΔH°_f_), compared to **7c** and **8c** (Table 2S). Less favourable binding of **7b** and **8b** with α_GTP_/β tubulin dimers is partial a result of a weaker H-bond stabilisation of the introduced arms due to unfavourable H-bond angles and distances with the proton acceptors of tubulins, i.e. with the carbonyl group of P173α (Figure 5Sg) or the carboxylate of E183α (Figure 5Sh). Thus, the decreased anticancer activities of **7b** and **8b**, compared to **7c** and **8c**, seems to result rather from a weaker H-bonding of the arms **7b** and **8b** with tubulins, than from their bulkiness (Table 2).

## Conclusions

Using various alkyl bromides, the S_N_2-type etherification at C(9)/C(10) of the tropolone ring of **2** has been performed. Reactivity tests indicated that regioselectivity of etherification/C(9) or C(10)/can be altered using different bases and solvent systems. The use of the inorganic base/THF system favours the formation of C(9)-ether products, whereas application of the organic base/toluene system yielded favourably C(10)-ether products. Dipolar cycloaddition (CuAAC) and Heck reactions using C(9)-ether and C(10)-ether products were performed to obtain structurally diverse hybrids with biological relevant blocks. Among the simple ether intermediates at C(9) or C(10), those with less bulky substituents (**3d**–**3i**, **4d**–**4i**) showed the best anticancer properties at IC_50_s ∼ 1–2 nM, which is a comparable or even better result than that for **1**. Comparing the binding modes this-type simple ether derivatives revealed that the presence of less bulky and unsaturated substituents at C(9) increases the binding energy profit with α_GTP_/β dimeric tubulins due to an extra π-π stacking and hydrophobic contacts, as calculated for **3e**, **4e**, **3f**, and **4f**. Overall, the presence of lengthy and bulky substituents within most hybrids, containing triazole or vinyl-benzyl bridges (compounds of **5**, **6**, **7**, and **8** types), destabilises binding with dimeric tubulins α_GTP_/β and decreases anticancer potency. Exceptions of this relationship are hybrids with basic and terminal heterocycles (**7c** and **8c**) for which beneficial binding mode and an improved water solubility/in protonated forms/contribute to relatively high anticancer effects. It should be mentioned that the toxicities of hybrids **7b** and **8b** containing pyridine as an end-motif, are lower (IC_50_s _HDF_ ∼ 4–5 nM) than those of the most active simple ether products and **1**, at the attractive anticancer activity (IC_50_ ∼ 2–2.5 nM).

## Supplementary Material

Supplemental Material
