# Supplementary material for: Regioselective approach to colchiceine tropolone ring functionalization at C(9) and C(10) yielding new anticancer hybrid derivatives containing heterocyclic structural motifs
Source: J Enzyme Inhib Med Chem. 2022 Jan 24;37(1):597–605. doi: 10.1080/14756366.2022.2028782 (PMC8788354; doi:10.1080/14756366.2022.2028782)
Supplement: Supplemental Material [file IENZ_A_2028782_SM6785.pdf]

Supplemental Material

Regioselective approach to colchiceine tropolone ring functionalization at C(9) and C(10) yielding new anticancer hybrid derivatives containing heterocyclic structural motifs

Krystian Pyta[a], Natalia Skrzypczak[a], Piotr Ruszkowski[b], Franz Bartl[c], Piotr Przybylski[a]*

[a]Faculty of Chemistry, Adam Mickiewicz University, Uniwersytetu Poznanskiego 8, 61-614, Poznan, Poland; [b]Department of Pharmacology, Poznan University of Medical Sciences, Rokietnicka 5a, 60-806 Poznan, Poland; [c]Lebenswissenschaftliche Fakultät, Institut für Biologie, Biophysikalische Chemie Humboldt-Universität zu Berlin Invalidenstrasse 42 10099 Berlin, Germany

*Corresponding author.
E-mail address: piotrp@amu.edu.pl (P. Przybylski).

Contents:

Table 1S. Comparison of S_N2 reaction of **2** with different bromides, performed in different reaction times and solvents, and in the presence of two types bases (inorganic NaH and organic MTBD). 7

Table 2S. Binding energies [$\Delta H°_f$ (kcal/mol)] of colchicine (**1**), simple ether derivatives of types **3** and **4** and hybrids of types **7** and **8** to dimeric α_{GTP}/β tubulins (PDB 1SA0)[1], calculated by MOG-PM6 method (*Scigress* package FJ 2.6, EU 3.1.9).[2] 8

Figure 1S. Structures and the partial charges of: (a) salts formed between 2 and MTBD *via* C(10) alkoxylate (left) or *via* C(9) alkoxylate (right), and (b) salts with Na^+ cation where coordination sphere is fulfilled with DMF and THF solvent molecules; formed *via* C(10) alkoxylates (left) or C(9) alkoxylates (right); calculated by B88-LYP (GGA) DFT method (*Scigress* F.J. 2.6, EU 3.1.9., 2008-2019 Fujitsu); E units are expressed in a.u. [2].................. 9

Figure 2S. Comparison FT-IR spectra of: 2 (black), 1:1 complex of 2-MTBD (red), C(9)-ether derivative 3f (blue) and C(10)-derivative 4f (green); in ranges: (a) 4000-400 cm^{-1}, (b) 1800-1250 cm^{-1}, all recorded in CH_2Cl_2. 10

Figure 3S. Comparison FT-IR spectra of: 2 (black), 1:1 complex of 2-MTBD (red), 2-NaH (pink) and MTBD (green); in ranges: 4000-400 cm^{-1} and 1800-1250 cm^{-1}, 2, 2-MTBD, MTBD recorded in CH_2Cl_2, 2-NaH recorded in KBr pellet.. 11

Figure 4S. Experimentally recorded ^1H-^{13}C HMBC long-range couplings within structure of hybrid 5f, optimized by B88 LYP (GGA) DFT method (*Scigress* package).[2] 12

Figure 5S. Docking models for colchiceine and its derivatives containing arms attached to C(9) or C(10) positions, at the binding pocket of tubulin dimer αGTP/β (PDB 1SA0)[1]: (a) 3a – pink, C(9)-substitution pattern; (b) 4a – pink, C(10)-substitution pattern; (c) 3j – rose, C(9)-substitution pattern; (d) 4j - rose, C(10)-substitution

pattern; (e) 5b – violet, C(9)-substitution pattern; (f) 6b – violet, C(10)-substitution pattern; (g) 7b – brown, C(9)-substitution pattern; (h) 8b – brown, C(10)-substitution pattern; optimized *via* MO-G PM6 semi-empirical method using MOZYME algorithm for huge molecules (Scigress package 3.1.9, 2008-2019)[2]. Tubulin units are distinguished by different colors: α-tubulin and its key amino acids (bold green sticks) and β-tubulin and its key amino acids (blue bold sticks). 13

Scheme 1S. Synthesis and structure new geldanamycin derivatives (9 and 10). 18

Figure 6S. DFT calculated geometries (xyz coordinates) and partial charges for complexes of 2 with Na^+ and MTBD. 19

2(10-**O**¯)→Na^+(2DMF+3THF) 19

2(9-**O**¯)→Na^+(2DMF+2THF) 24

2(10-**O**¯)→$[H\text{-}MTBD]^+$ 28

2(9-**O**¯)→$[H\text{-}MTBD]^+$ 31

Figure 7S. DFT calculated geometries (xyz coordinates) of **5f**. 35

XYZ data of hybrid 5f 35

EXPERIMENTAL SECTION: 41

FT-IR spectra 70

Figure 8S. FT-IR spectrum of compound 3a (in KBr). 70

Figure 9S. FT-IR spectrum of compound 3b (in KBr). 71

Figure 10S. FT-IR spectrum of compound 3c (in KBr). 72

Figure 11S. FT-IR spectrum of compound 3d (in KBr). 73

Figure 12S. FT-IR spectrum of compound 3e (in KBr). 74

Figure 13S. FT-IR spectrum of compound 3f (in CH_2Cl_2). 75

Figure 14S. FT-IR spectrum of compound 3g (in KBr). 76

Figure 15S. FT-IR spectrum of compound 3h (in KBr). 77

Figure 16S. FT-IR spectrum of compound 3i (in KBr). 78

Figure 17S. FT-IR spectrum of compound 3j (in KBr). 79

Figure 18S. FT-IR spectrum of compound 4a (in KBr). 80

Figure 19S. FT-IR spectrum of compound 4b (in KBr). 81

Figure 20S. FT-IR spectrum of compound 4c (in KBr). 82

Figure 21S. FT-IR spectrum of compound 4d (in KBr). 83

Figure 22S. FT-IR spectrum of compound 4e (in KBr). 84

Figure 23S. FT-IR spectrum of compound 4f (in CH_2Cl_2). 85

Figure 24S. FT-IR spectrum of compound 4g (in KBr). 86

Figure 25S. FT-IR spectrum of compound 4h (in KBr). 87

Figure 26S. FT-IR spectrum of compound 4i (in KBr). 88

Figure 27S. FT-IR spectrum of compound 4j (in KBr). 89
Figure 28S. FT-IR spectrum of compound 5a (in KBr). 90
Figure 29S. FT-IR spectrum of compound 5b (in KBr). 91
Figure 30S. FT-IR spectrum of compound 5c (in KBr). 92
Figure 31S. FT-IR spectrum of compound 5d (in KBr). 93
Figure 32S. FT-IR spectrum of compound 5e (in KBr). 94
Figure 33S. FT-IR spectrum of compound 5f (in KBr). 95
Figure 34S. FT-IR spectrum of compound 6a (in KBr). 96
Figure 35S. FT-IR spectrum of compound 6b (in KBr). 97
Figure 36S. FT-IR spectrum of compound 6c (in KBr). 98
Figure 37S. FT-IR spectrum of compound 6d (in KBr). 99
Figure 38S. FT-IR spectrum of compound 6e (in KBr). 100
Figure 39S. FT-IR spectrum of compound 6f (in KBr). 101
Figure 40S. FT-IR spectrum of compound 7a (in KBr). 102
Figure 41S. FT-IR spectrum of compound 7b (in KBr). 103
Figure 42S. FT-IR spectrum of compound 7c (in KBr). 104
Figure 43S. FT-IR spectrum of compound 7d (in KBr). 105
Figure 44S. FT-IR spectrum of compound 8a (in KBr). 106
Figure 45S. FT-IR spectrum of compound 8b (in KBr). 107
Figure 46S. FT-IR spectrum of compound 8c (in KBr). 108
Figure 47S. FT-IR spectrum of compound 8d (in KBr). 109
Figure 48S. FT-IR spectrum of compound 9 (in KBr). 110
Figure 49S. FT-IR spectrum of compound 10 (in KBr). 111
NMR spectra 112
Figure 50S. ^{1}H NMR spectrum of compound 3a in $CDCl_3$. 113
Figure 51S. ^{13}C NMR spectrum of compound 3a in $CDCl_3$. 114
Figure 52S. ^{1}H NMR spectrum of compound 3b in $CDCl_3$. 115
Figure 53S. ^{13}C NMR spectrum of compound 3b in $CDCl_3$ 116
Figure 54S. ^{1}H NMR spectrum of compound 3c in $CDCl_3$. 117
Figure 55S. ^{1}H NMR spectrum of compound 3c in $CDCl_3$. 118
Figure 56S. ^{1}H NMR spectrum of compound 3d in $CDCl_3$. 119
Figure 57S. ^{1}H NMR spectrum of compound 3d in $CDCl_3$. 120
Figure 58S. ^{1}H NMR spectrum of compound 3e in $CDCl_3$. 121
Figure 59S. ^{13}C NMR spectrum of compound 3e in $CDCl_3$. 122

Figure 60S. ^{1}H NMR spectrum of compound 3f in $CDCl_3$. 123
Figure 61S. ^{13}C NMR spectrum of compound 3f in $CDCl_3$. 124
Figure 62S. ^{1}H NMR spectrum of compound 3g in $CDCl_3$. 125
Figure 63S. ^{13}C NMR spectrum of compound 3g in $CDCl_3$. 126
Figure 64S. ^{1}H NMR spectrum of compound 3h in $CDCl_3$. 127
Figure 65S. ^{13}C NMR spectrum of compound 3h in $CDCl_3$. 128
Figure 66S. ^{1}H NMR spectrum of compound 3i in $CDCl_3$. 129
Figure 67S. ^{13}C NMR spectrum of compound 3i in $CDCl_3$. 130
Figure 68S. ^{1}H NMR spectrum of compound 3j in $CDCl_3$. 131
Figure 69S. ^{13}C NMR spectrum of compound 3j in $CDCl_3$. 132
Figure 70S. ^{1}H NMR spectrum of compound 4a in $CDCl_3$ 133
Figure 71S. ^{13}C NMR spectrum of compound 4a in $CDCl_3$ 134
Figure 72S. ^{1}H NMR spectrum of compound 4b in $CDCl_3$. 135
Figure 73S. ^{13}C NMR spectrum of compound 4b in $CDCl_3$. 136
Figure 74S. ^{1}H NMR spectrum of compound 4c in $CDCl_3$ 137
Figure 75S. ^{13}C NMR spectrum of compound 4c in $CDCl_3$ 138
Figure 76S. ^{1}H NMR spectrum of compound 4d in $CDCl_3$. 139
Figure 77S. ^{13}C NMR spectrum of compound 4d in $CDCl_3$. 140
Figure 78S. ^{1}H NMR spectrum of compound 4e in $CDCl_3$ 141
Figure 79S. ^{13}C NMR spectrum of compound 4e in $CDCl_3$ 142
Figure 80S. ^{1}H NMR spectrum of compound 4f in $CDCl_3$. 143
Figure 81S. ^{13}C NMR spectrum of compound 4f in $CDCl_3$. 144
Figure 82S. ^{1}H NMR spectrum of compound 4g in $CDCl_3$. 145
Figure 83S. ^{13}C NMR spectrum of compound 4g in $CDCl_3$. 146
Figure 84S. ^{1}H NMR spectrum of compound 4h in $CDCl_3$. 147
Figure 85S. ^{13}C NMR spectrum of compound 4h in $CDCl_3$. 148
Figure 86S. ^{1}H NMR spectrum of compound 4i in $CDCl_3$. 149
Figure 87S. ^{13}C NMR spectrum of compound 4i in $CDCl_3$. 150
Figure 88S. ^{1}H NMR spectrum of compound 4j in $CDCl_3$. 151
Figure 89S. ^{13}C NMR spectrum of compound 4j in $CDCl_3$. 152
Figure 90S. ^{1}H NMR spectrum of compound 5a in $CDCl_3$ 153
Figure 91S. ^{13}C NMR spectrum of compound 5a in $CDCl_3$ 154
Figure 92S. ^{1}H NMR spectrum of compound 5b in $CDCl_3$. 155
Figure 93S. ^{13}C NMR spectrum of compound 5b in $CDCl_3$. 156

Figure 94S. ^{1}H NMR spectrum of compound 5c in $CDCl_3$. 157
Figure 95S. ^{13}C NMR spectrum of compound 5c in $CDCl_3$. 158
Figure 96S. ^{1}H NMR spectrum of compound 5d in $CDCl_3$. 159
Figure 97S. ^{13}C NMR spectrum of compound 5d in $CDCl_3$. 160
Figure 98S. ^{1}H NMR spectrum of compound 5e in $CDCl_3$. 161
Figure 99S. ^{13}C NMR spectrum of compound 5e in $CDCl_3$. 162
Figure 100S. ESI MS^+ spectrum of 5e. 163
Figure 101S. ^{1}H NMR spectrum of compound 5f in $CDCl_3$. 164
Figure 102S. ^{13}C NMR spectrum of compound 5f in $CDCl_3$. 165
Figure 103S. ^{1}H NMR spectrum of compound 6a in $CDCl_3$. 166
Figure 104S. ^{13}C NMR spectrum of compound 6a in $CDCl_3$. 167
Figure 105S. ^{1}H NMR spectrum of compound 6b in $CDCl_3$. 168
Figure 106S. ^{13}C NMR spectrum of compound 6b in $CDCl_3$. 169
Figure 107S. ^{1}H NMR spectrum of compound 6c in $CDCl_3$. 170
Figure 108S. ^{13}C NMR spectrum of compound 6c in $CDCl_3$. 171
Figure 109S. ^{1}H NMR spectrum of compound 6d in $CDCl_3$. 172
Figure 110S. ^{13}C NMR spectrum of compound 6d in $CDCl_3$. 173
Figure 111S. ^{1}H NMR spectrum of compound 6e in $CDCl_3$. 174
Figure 112S. ^{13}C NMR spectrum of compound 6e in $CDCl_3$. 175
Figure 113S. ESI MS^+ spectrum of 6e. 176
Figure 114S. ^{1}H NMR spectrum of compound 6f in $CDCl_3$. 177
Figure 115S. ^{13}C NMR spectrum of compound 6f in $CDCl_3$. 178
Figure 116S. ^{1}H NMR spectrum of compound 7a in $CDCl_3$. 179
Figure 117S. ^{13}C NMR spectrum of compound 7a in $CDCl_3$. 180
Figure 118S. ^{1}H NMR spectrum of compound 7b in $CDCl_3$. 181
Figure 119S. ^{13}C NMR spectrum of compound 7b in $CDCl_3$. 182
Figure 120S. ^{1}H NMR spectrum of compound 7c in $CDCl_3$. 183
Figure 121S. ^{13}C NMR spectrum of compound 7c in $CDCl_3$. 184
Figure 122S. ^{1}H NMR spectrum of compound 7d in $CDCl_3$. 185
Figure 123S. ^{13}C NMR spectrum of compound 7d in $CDCl_3$. 186
Figure 124S. ^{1}H NMR spectrum of compound 8a in $CDCl_3$. 187
Figure 125S. ^{13}C NMR spectrum of compound 8a in $CDCl_3$. 188
Figure 126S. ^{1}H NMR spectrum of compound 8b in $CDCl_3$. 189
Figure 127S. ^{13}C NMR spectrum of compound 8b in $CDCl_3$. 190

Figure 128S. ^{1}H NMR spectrum of compound 8c in $CDCl_3$.......... 191
Figure 129S. ^{13}C NMR spectrum of compound 8c in $CDCl_3$.......... 192
Figure 130S. ^{1}H NMR spectrum of compound 8d in $CDCl_3$. 193
Figure 131S. ^{13}C NMR spectrum of compound 8d in $CDCl_3$. 194
Figure 132S. ^{1}H NMR spectrum of compound 9 in $CDCl_3$. 195
Figure 133S. ^{13}C NMR spectrum of compound 9 in $CDCl_3$. 196
Figure 134S. ^{1}H NMR spectrum of compound 10 in $CDCl_3$. 197
Figure 135S. ^{13}C NMR spectrum of compound 10 in $CDCl_3$. 198
Figure 136S. Structures of all novel chemical entities **3a-3j** presented in ChemDraw 12.0 format. 199
Figure 137S. Structures of all novel chemical entities **4a-4j** presented in ChemDraw 12.0 format. 200
Figure 138S. Structures of all novel chemical entities **5a-5f** presented in ChemDraw 12.0 format. 201
Figure 139S. Structures of all novel chemical entities **6a-6f** presented in ChemDraw 12.0 format. 202
Figure 140S. Structures of all novel chemical entities **7a-7d** presented in ChemDraw 12.0 format. 203
Figure 141S. Structures of all novel chemical entities **8a-8d** presented in ChemDraw 12.0 format. 204

Table 1S. Comparison of S_N2 reaction of **2** with different bromides, performed in different reaction times and solvents, and in the presence of two types bases (inorganic NaH and organic MTBD).

S_N2 reactant	Base	Solvent	Reaction time (h)	T (°C)	Convers. of **1** (%)	Yield (%) of (**3** + **4**)	Ratio (%) of (**3** : **4**)
BnBr	NaH	DMF	4	70	91.3	87.3(**3a** + **4a**)	59.8:40.2 (**3a** : **4a**)
BnBr	NaH	THF/DMF (1:0.5)	4	70	95.1	95.1(**3a** + **4a**)	68.3:31.7 (**3a** : **4a**)
4-iodo-BnBr	NaH	THF/DMF (1:0.5)	4	70	84.1	83.6(**3b** + **4b**)	71.5:28.5 (**3b** : **4b**)
Cinnamyl-Br	NaH	THF/DMF (1:0.5)	4	70	93.7	91.3(**3c** + **4c**)	77.2:22.8 (**3c** : **4c**)
Allyl-Br	NaH	THF/DMF (1:0.5)	4	70	95.1	94.8(**3d** + **4d**)	69.3:30.7 (**3d** : **4d**)
Crotyl-Br	NaH	THF/DMF (1:0.5)	4	70	95.7	95.0(**3e** + **4e**)	68.7:31.3 (**3e** : **4e**)
Propargyl-Br	NaH	THF/DMF (1:0.5)	4	70	66.3	65.5(**3f** + **4f**)	64.4:35.6 (**3f** : **4f**)
Ethyl-Br acetate	NaH	THF/DMF (1:0.5)	4	70	97.3	92.6(**3g** + **4g**)	66.8:33.2 (**3g** : **4g**)
BnBr	MTBD	Toluene	1	70	95.5	99.5(**3a** + **4a**)	28.6:71.4 (**3a** : **4a**)
4-iodo-BnBr	MTBD	Toluene	1	70	98.7	98.1(**3b** + **4b**)	33.6:66.4 (**3b** : **4b**)
Cinnamyl-Br	MTBD	Toluene	1	70	99.4	99.3(**3c** + **4c**)	34.6:65.4 (**3c** : **4c**)
Allyl-Br	MTBD	Toluene	1	70	98.6	97.8(**3d** + **4d**)	29.6:70.4 (**3d** : **4d**)
Crotyl-Br	MTBD	Toluene	1	70	90.0	96.6(**3e** + **4e**)	35.7:64.3 (**3e** : **4e**)
Propargyl-Br	MTBD	Toluene	1	70	95.6	95.5(**3f** + **4f**)	29.8:70.2 (**3f** : **4f**)
Ethyl-Br acetate	MTBD	Toluene	1	70	90.1	89.6(**3g** + **4g**)	27.6:72.4 (**3g** : **4g**)

Table 2S. Binding energies [$\Delta H°_f$ (kcal/mol)] of colchicine (**1**), simple ether derivatives of types **3** and **4** and hybrids of types **7** and **8** to dimeric α_{GTP}/β tubulins (PDB 1SA0)[1], calculated by MOG-PM6 method (*Scigress* package FJ 2.6, EU 3.1.9).[2]

**Compound** [substitution]	$\Delta H°_f$ [kcal/mol]
**1** (C10) (Fig1a)	-149.72
**3a** (C9) (Fig S1a)	-123.82
**4a** (C10) (Fig S1b)	-121.95
**3e** (C9) (Fig1b)	-159.18
**4e** (C10) (Fig1c)	-146.11
**3f** (C9) (Fig1d)	-150.11
**4f** (C10) (Fig1e)	-147.80
**3j** (C9) (Fig S1c)	-112.92
**4j** (C10) (Fig S1d)	-119.37
**5b** (C9) (Fig S1e)	-122.79
**6b** (C10) (Fig S1f)	-119.01
**7a** (C9) (Fig 2a)	-102.64
**8a** (C10) (Fig 2b)	-91.53
**7b** (C9) (Fig S1g)	-120.27
**8b** (C10) (Fig S1h)	-118.98
**7c** (C9) (Fig 2c)	-142.75
**8c** (C10) (Fig 2d)	-139.16

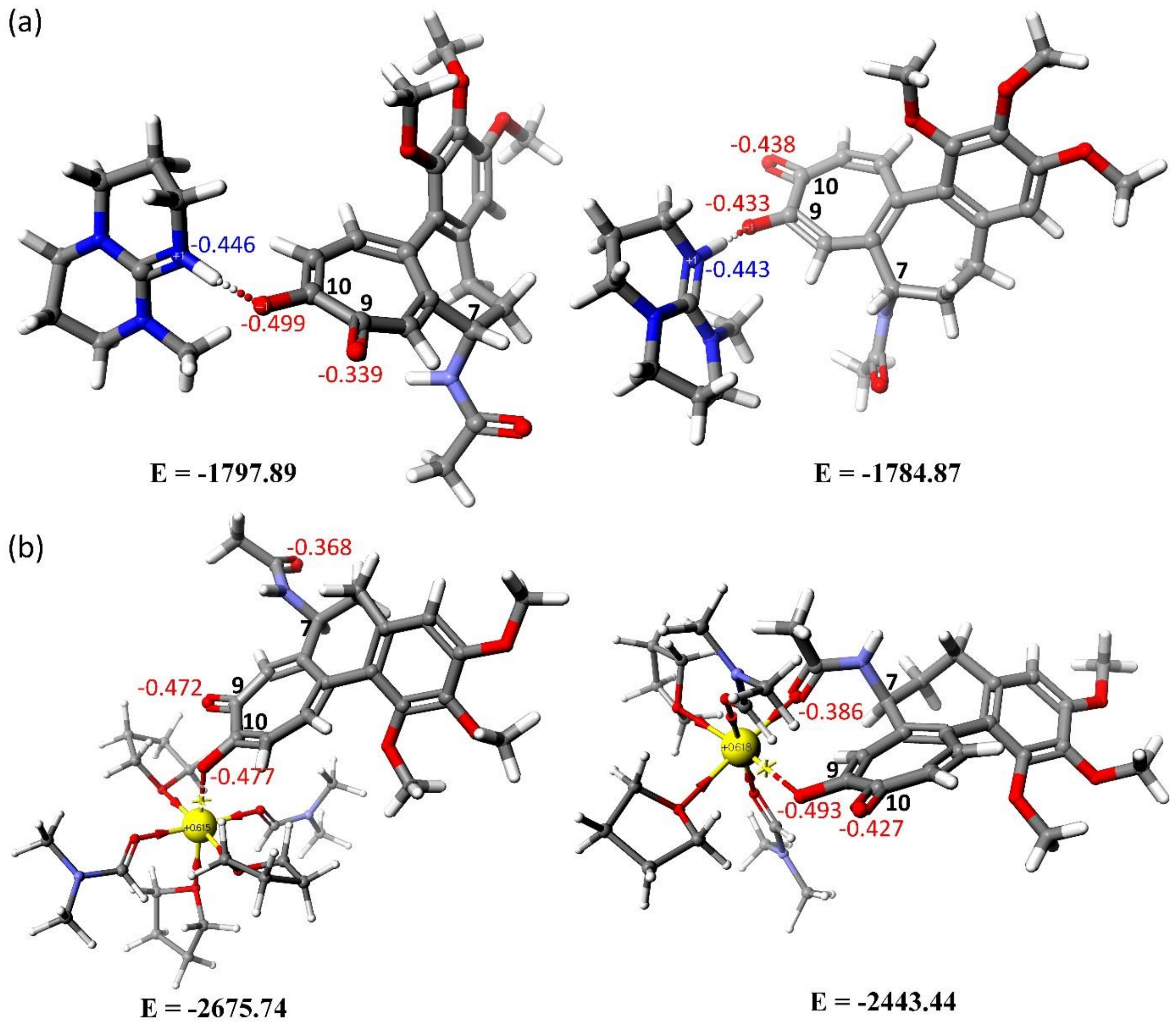

Figure 1S. Structures and the partial charges of: (a) salts formed between **2** and MTBD *via* C(10) alkoxylate (left) or *via* C(9) alkoxylate (right), and (b) salts with Na^+ cation where coordination sphere is fulfilled with DMF and THF solvent molecules; formed *via* C(10) alkoxylates (left) or C(9) alkoxylates (right); calculated by B88-LYP (GGA) DFT method (*Scigress* F.J. 2.6, EU 3.1.9., 2008-2019 Fujitsu); E units are expressed in a.u. [2]

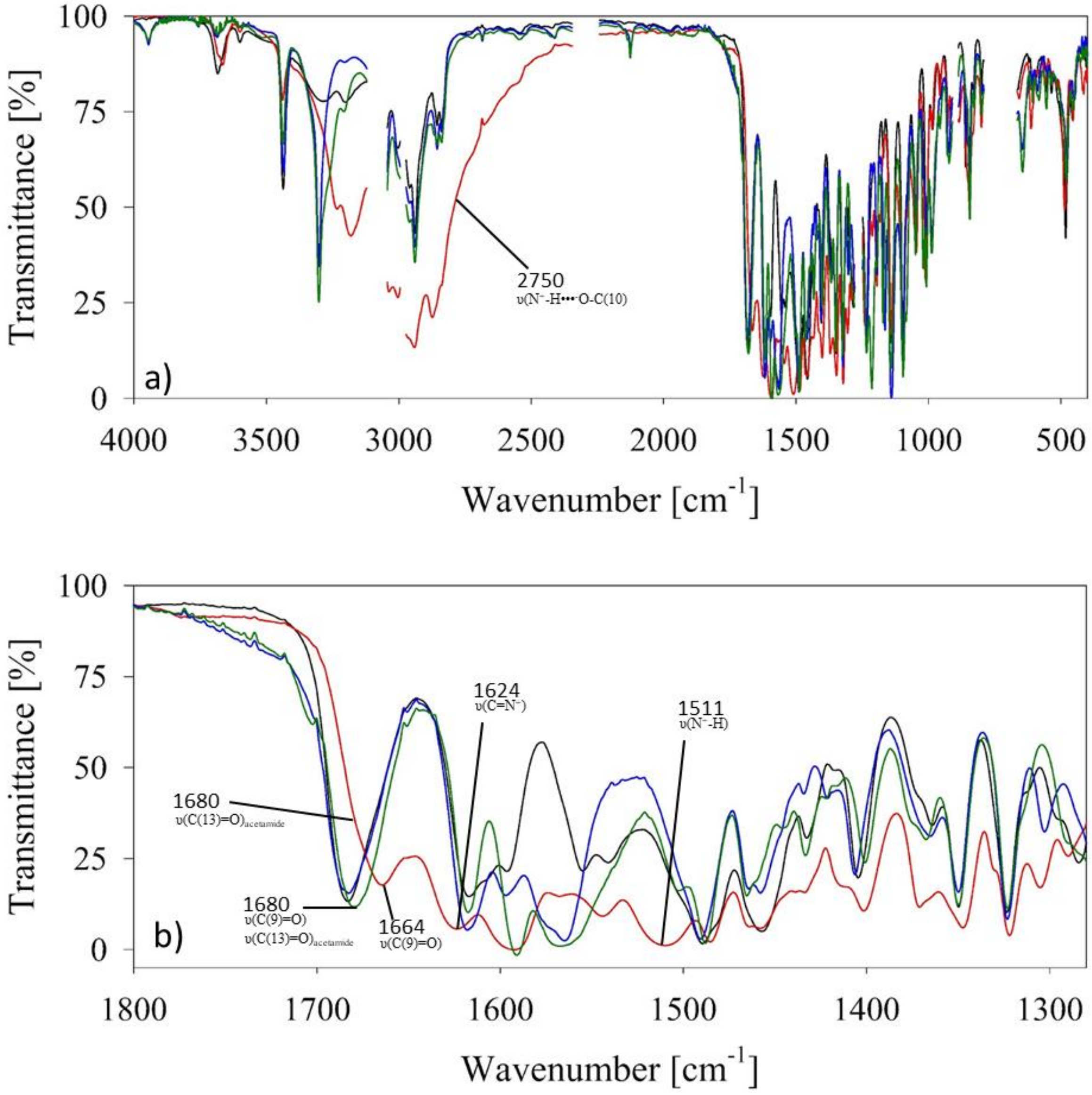

Figure 2S. Comparison FT-IR spectra of: **2** (black), 1:1 complex of **2**-MTBD (red), C(9)-ether derivative **3f** (blue) and C(10)-derivative **4f** (green); in ranges: (a) 4000-400 cm^{-1}, (b) 1800-1250 cm^{-1}, all recorded in CH_2Cl_2.

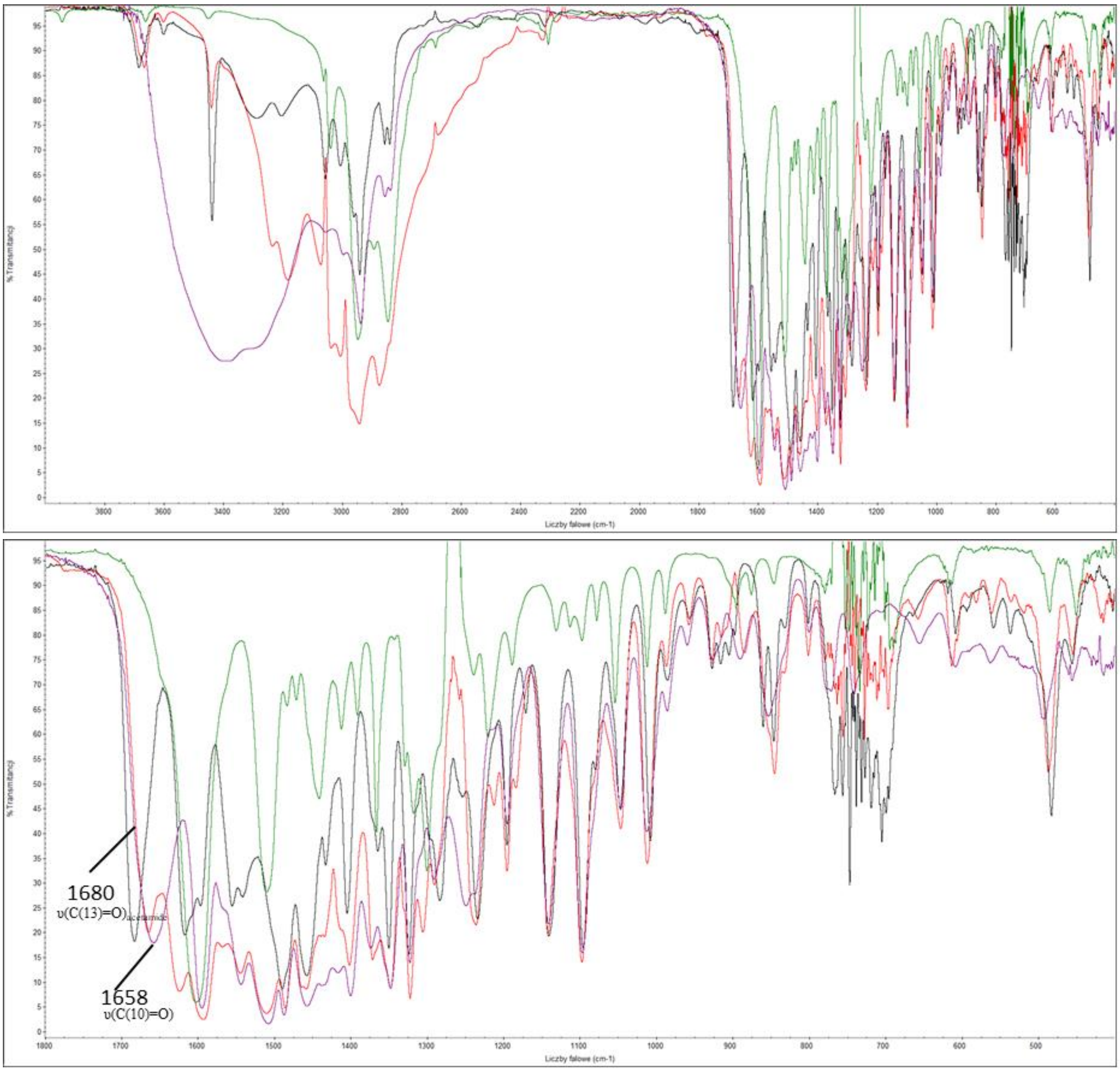

Figure 3S. Comparison FT-IR spectra of: **2** (black), 1:1 complex of **2-**MTBD (red), **2**-NaH (pink) and MTBD (green); in ranges: 4000-400 cm^{-1} and 1800-1250 cm^{-1}, **2**, **2-**MTBD, MTBD recorded in CH_2Cl_2, **2-**NaH recorded in KBr pellet.

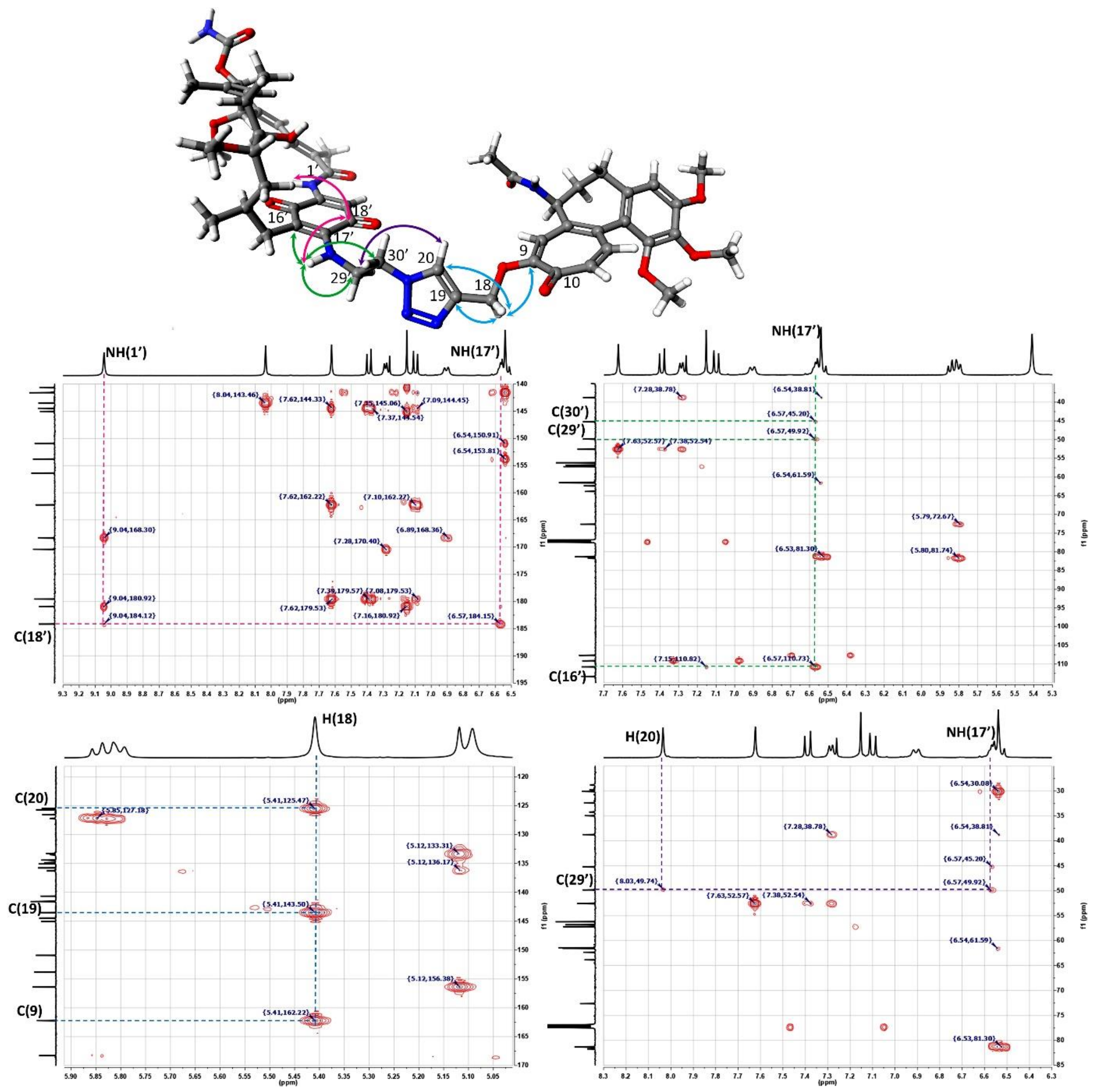

Figure 4S. Experimentally recorded ^{1}H-^{13}C HMBC long-range couplings within structure of hybrid **5f**, optimized by B88 LYP (GGA) DFT method (*Scigress* package).[2]

Figure 5S. Docking models for colchiceine and its derivatives containing arms attached to C(9) or C(10) positions, at the binding pocket of tubulin dimer αGTP/β (PDB 1SA0)[1]: (a) **3a** – pink, C(9)-substitution pattern; (b) **4a** – pink, C(10)-substitution pattern; (c) 3j – rose, C(9)-substitution pattern; (d) **4j** - rose, C(10)-substitution pattern; (e) **5b** – violet, C(9)-substitution pattern; (f) **6b** – violet, C(10)-substitution pattern; (g) **7b** – brown, C(9)-substitution pattern; (h) **8b** – brown, C(10)-substitution pattern; optimized *via* MO-G PM6 semi-empirical method using MOZYME algorithm for huge molecules (Scigress package 3.1.9, 2008-2019)[2]. Tubulin units are distinguished by different colors: α-tubulin and its key amino acids (bold green sticks) and β-tubulin and its key amino acids (blue bold sticks).

a) **3a** - α_{GTP}/β

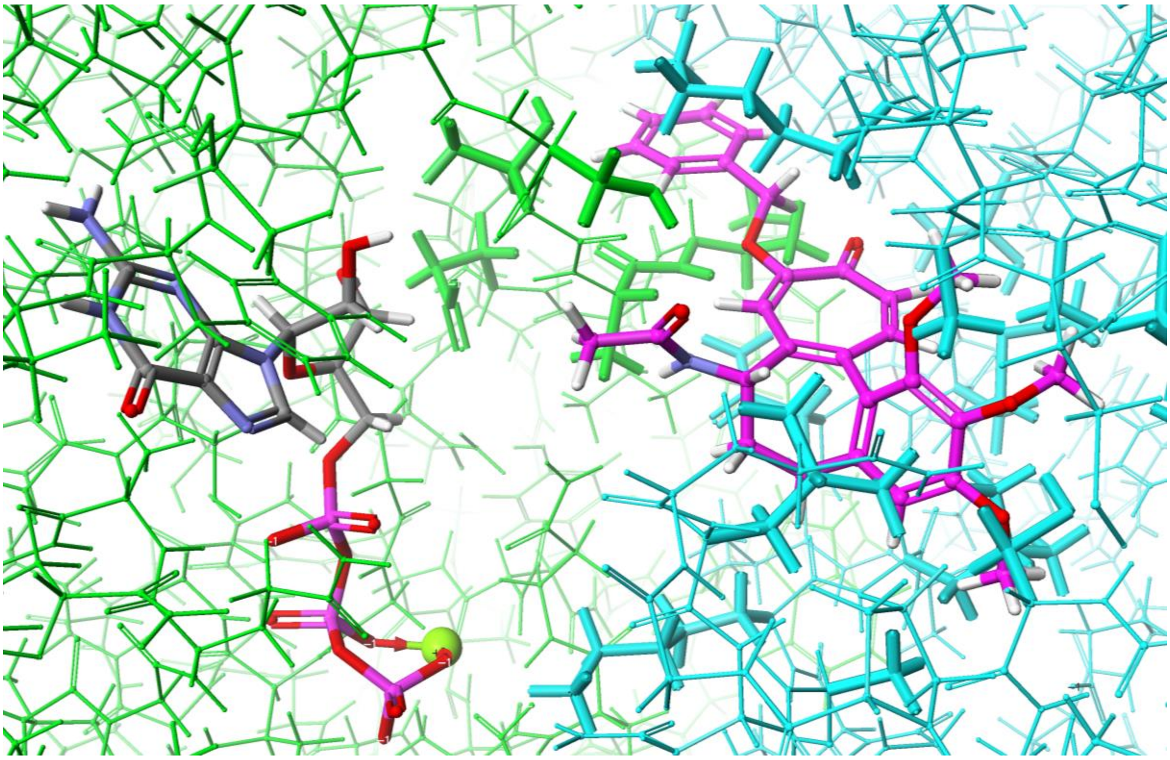

b) **4a** - α_{GTP}/β

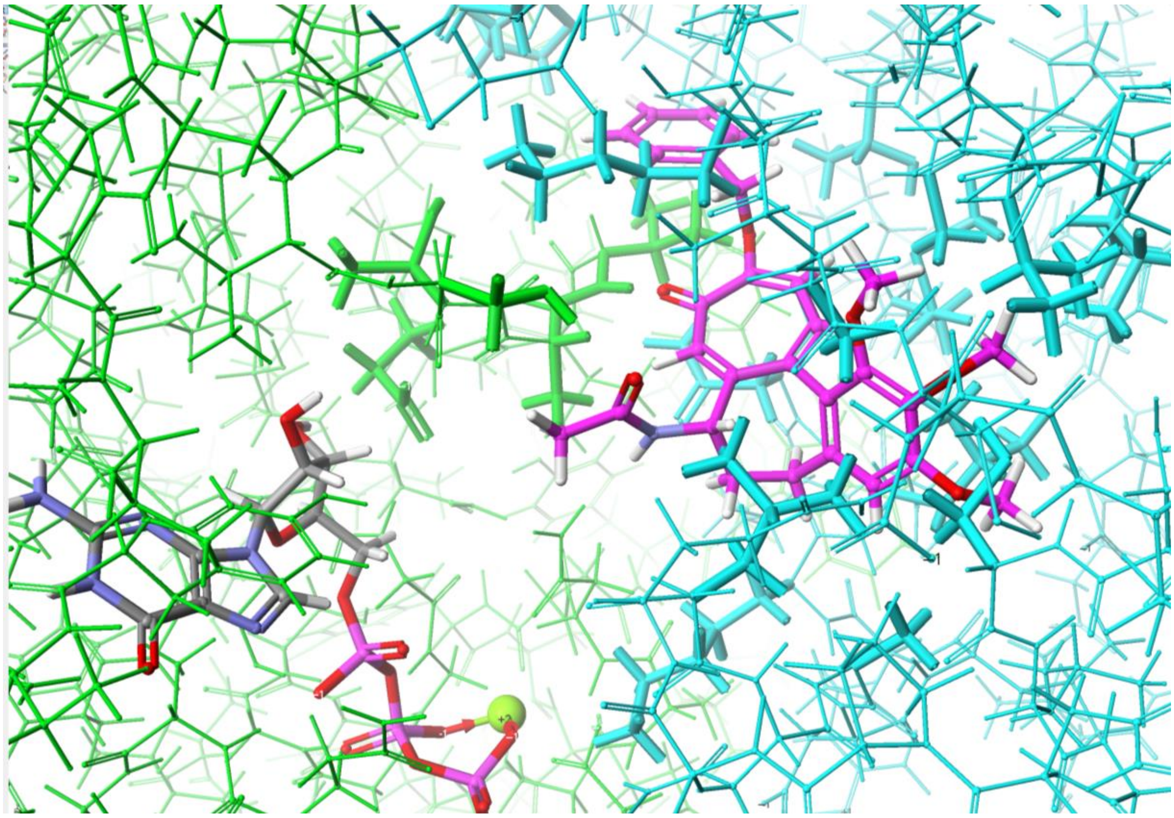

c) **3j** - α_{GTP}/β

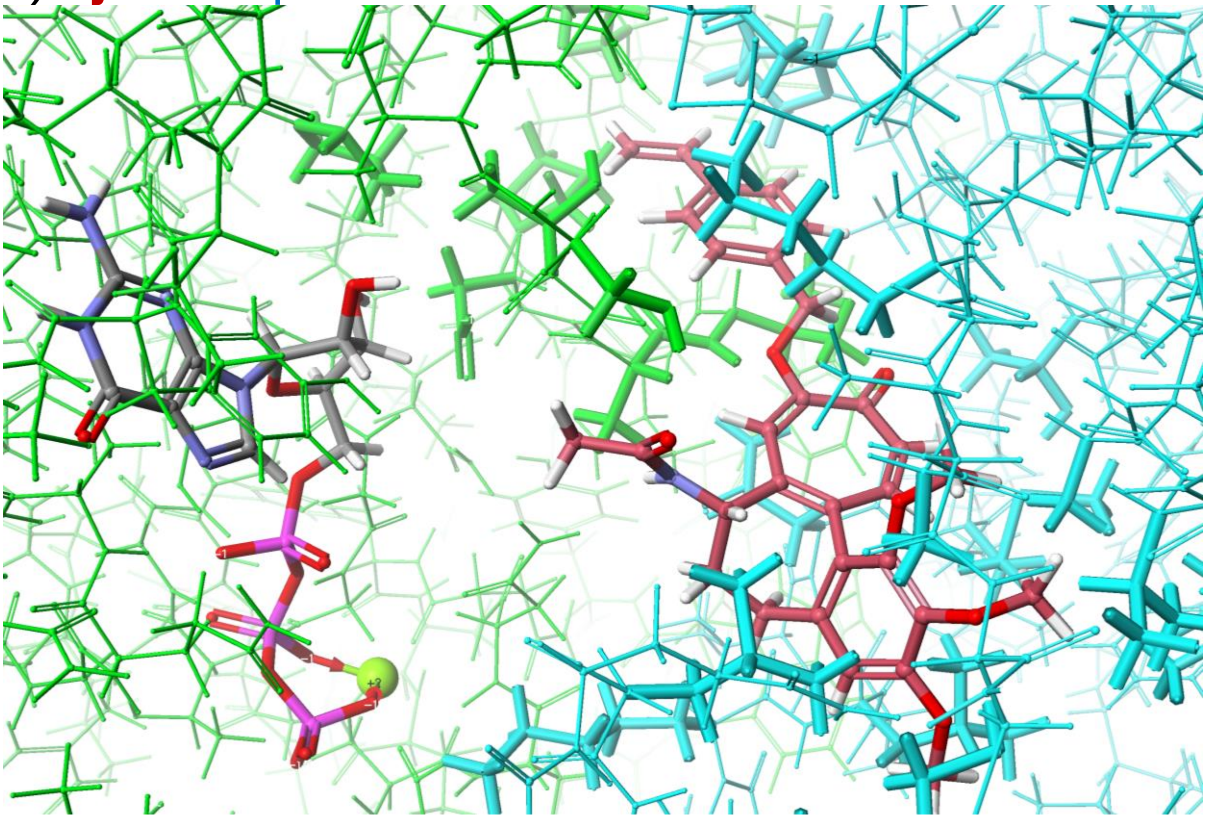

d) **4j** - α_{GTP}/β

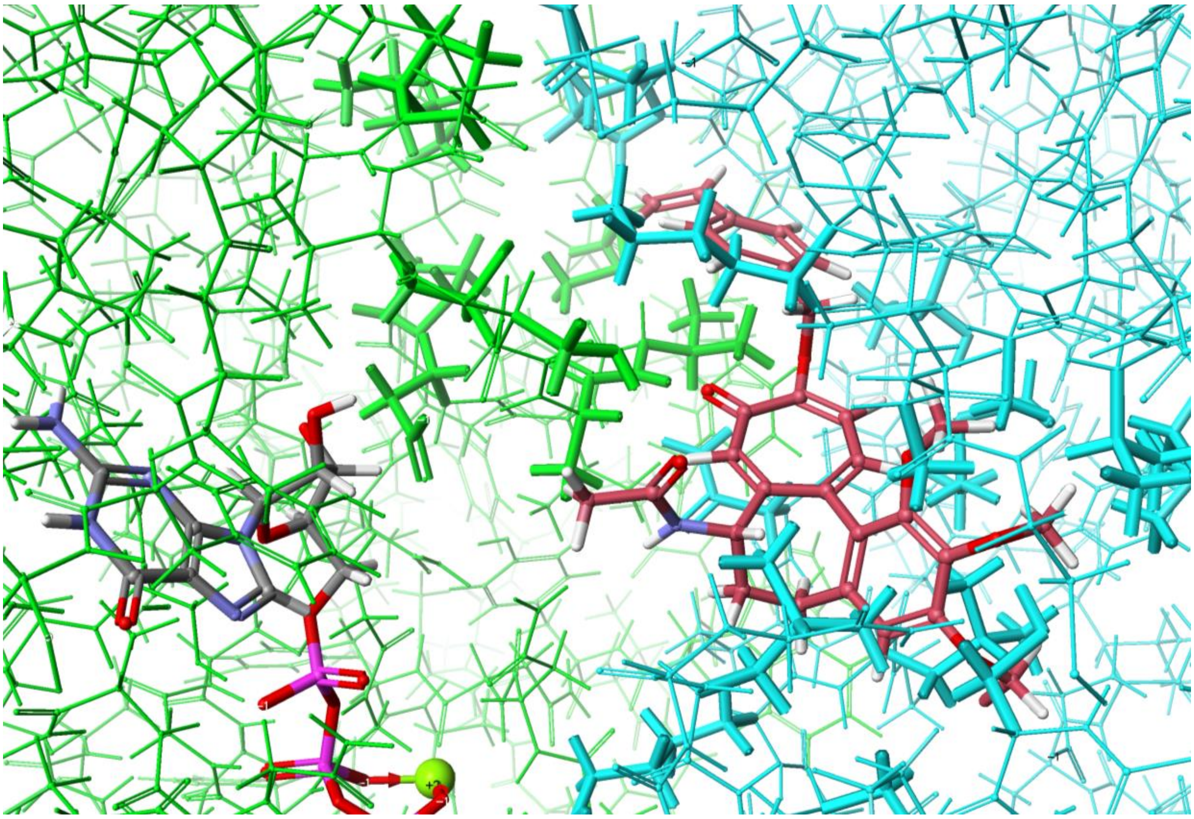

e) **5b** - α_{GTP}/β

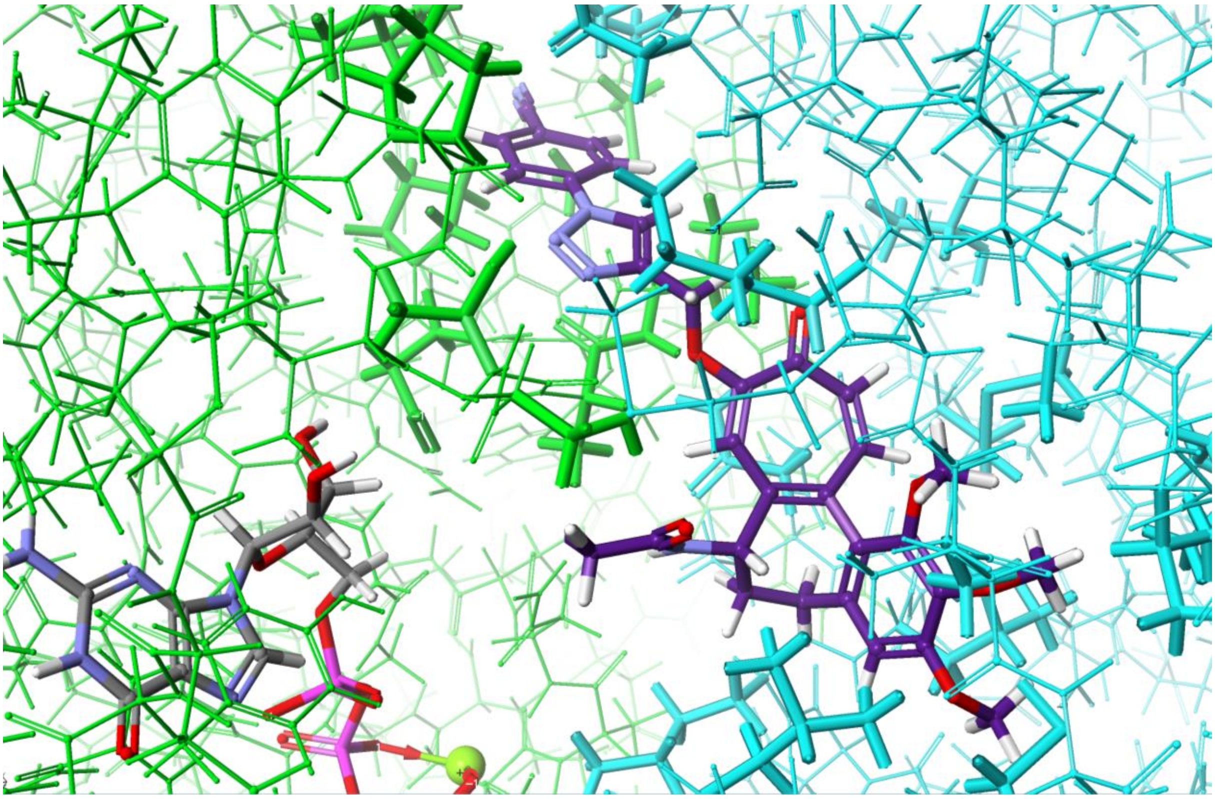

f) **6b** - α_{GTP}/β

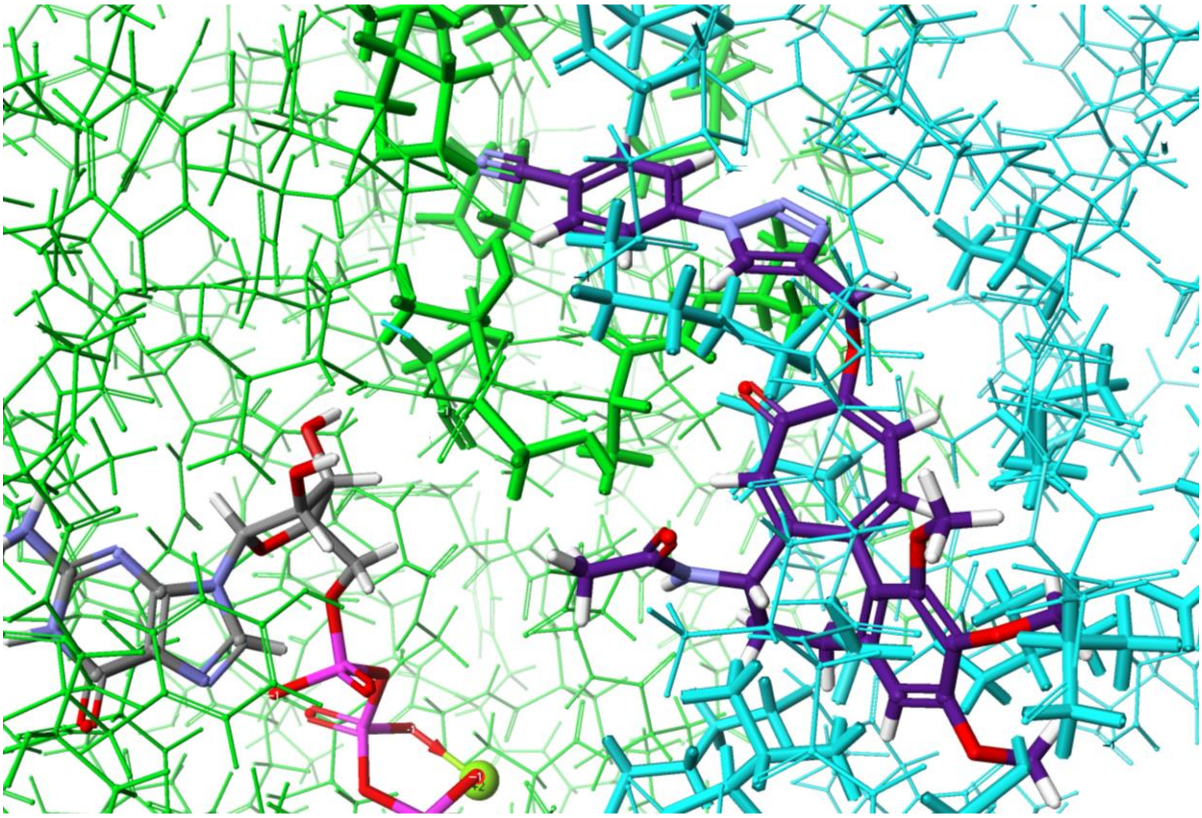

g) **7b** - α_{GTP}/β

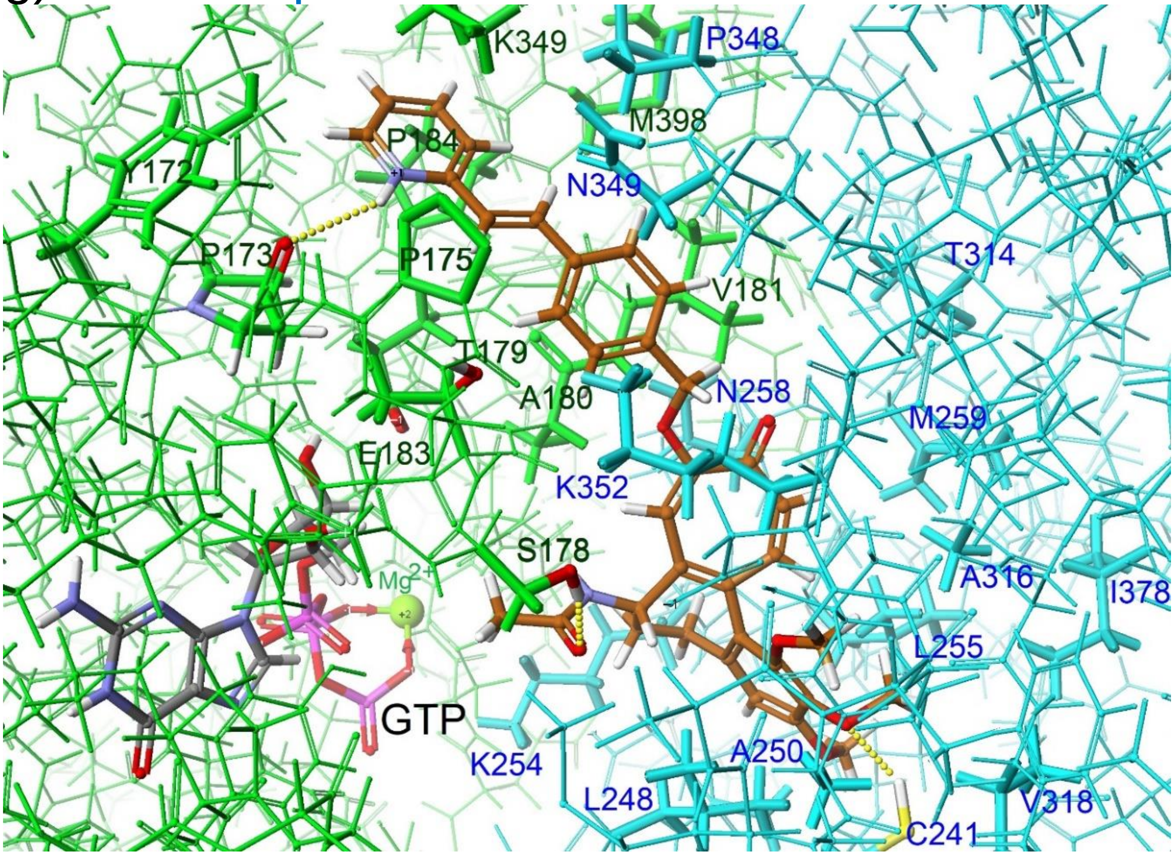

h) **8b** - α_{GTP}/β

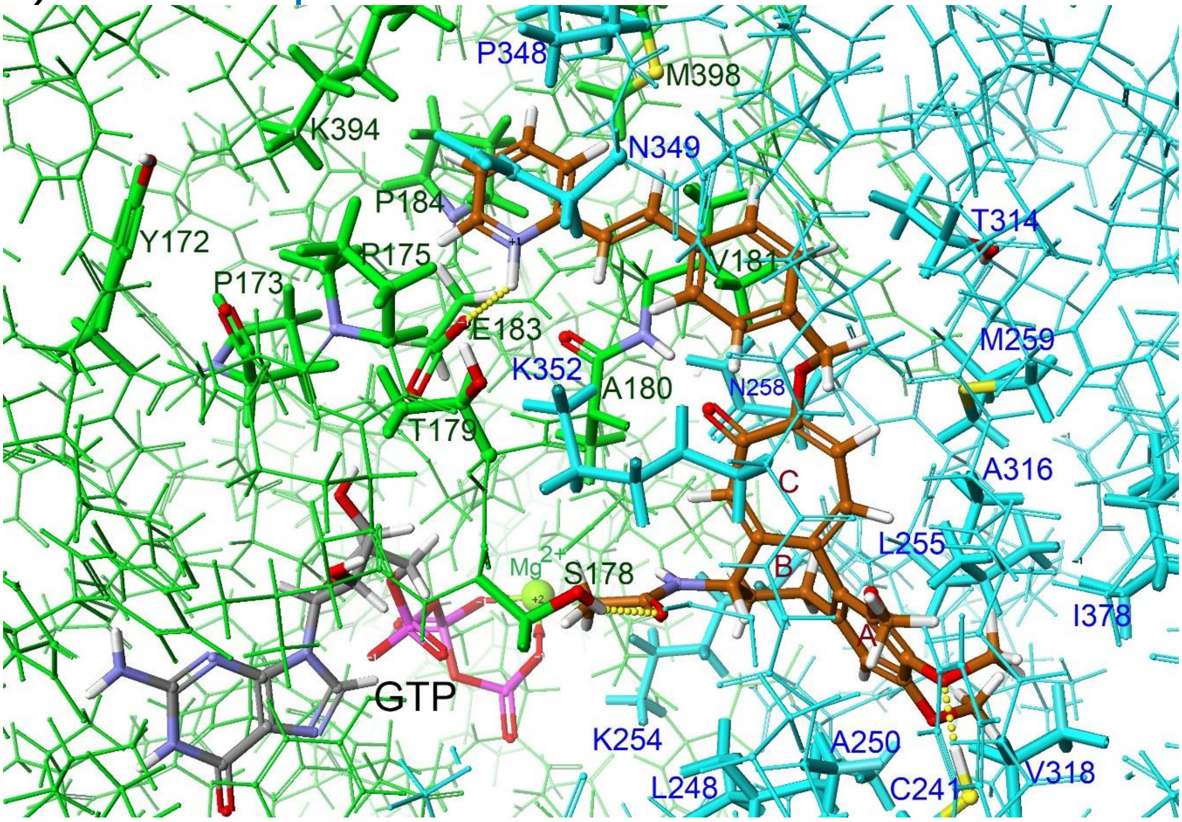

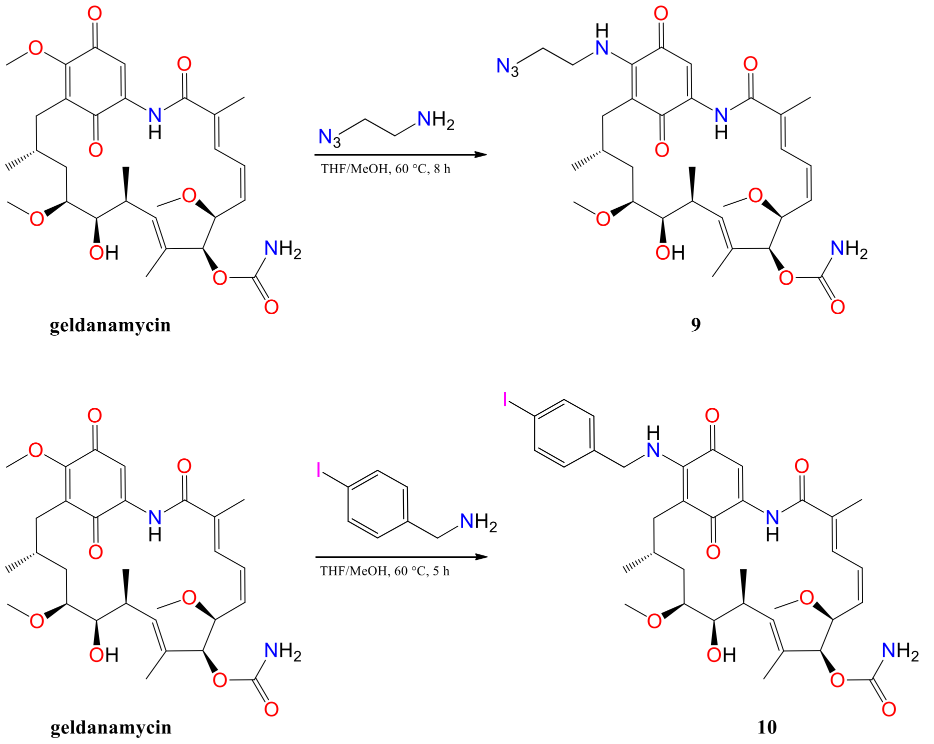

Scheme 1S. Synthesis and structure new geldanamycin derivatives (**9** and **10**).

Figure 6S. DFT calculated geometries (xyz coordinates) and partial charges for complexes of **2** with Na^+ and MTBD.

2(10-**O**¯)→Na^+(2DMF+3THF)

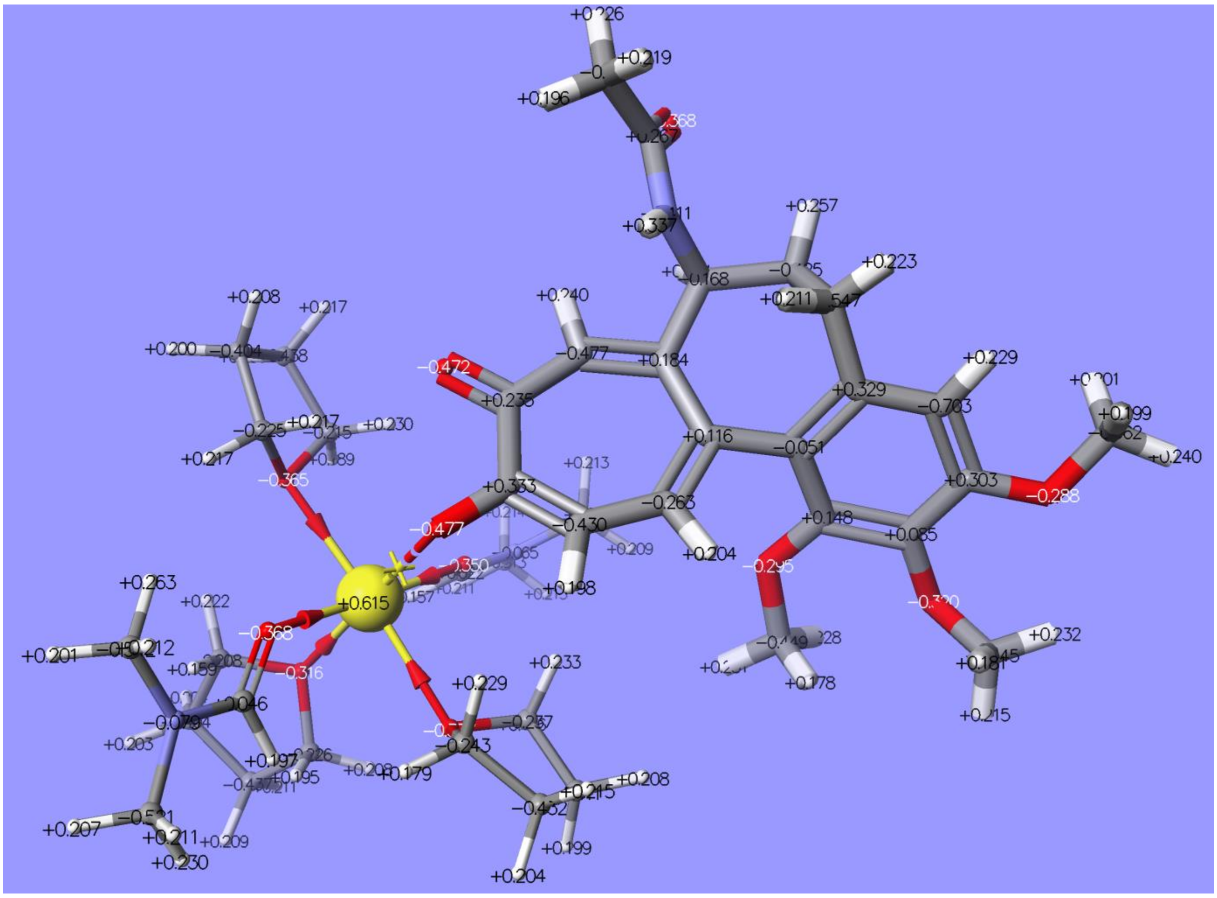

E= -2675.74 au

XYZ

C -10.820851 -1.806566 -15.467258

C -9.573829 -2.258604 -15.023389

C -9.528284 -3.127549 -13.908794

C -10.720803 -3.630823 -13.344962

C -11.965085 -3.220906 -13.847483

C -11.993117 -2.283952 -14.879593

C -10.928345 -0.631996 -16.397675

C -8.281607 -1.861704 -15.694293

O -8.270844 -3.478026 -13.478997

O -10.642868 -4.51585 -12.304157

O -13.106632 -3.671565 -13.240234

H -12.953891 -1.879334 -15.232326

C -8.000591 -4.530955 -12.567724

C -11.378204 -5.729428 -12.421157

C -14.351667 -3.050027 -13.52489

H -8.41592 -5.493421 -12.941782

H -8.394199 -4.292684 -11.554977

H -6.898284 -4.648555 -12.480539

H -11.330967 -6.12606 -13.460776

H -12.43677 -5.591899 -12.112266

H -10.927499 -6.486088 -11.742062

H -14.645811 -3.217558 -14.585164

H -14.311546 -1.960386 -13.299613

H -15.133784 -3.50473 -12.878122

C -7.909035 -0.459835 -16.03348

C -10.323281 0.547978 -15.618234

C -8.84988 0.762038 -15.972531

H -10.398774 -0.824684 -17.357464

H -11.991184 -0.415895 -16.651989

C -7.483831 -2.900364 -15.986391

C -6.706795 -0.106528 -16.518608

H -10.896427 1.4761 -15.84759

H -10.435868 0.392992 -14.519645

H -8.424495 1.453459 -15.206618

N -8.810137 1.389479 -17.302077

H -8.705716 0.787693 -18.131979

C -8.884843 2.761359 -17.475587

C -8.742908 3.233515 -18.918342

O -9.036399 3.537039 -16.561611

H -7.758086 2.926065 -19.336393

H -9.546442 2.803271 -19.556887

H -8.809567 4.342483 -18.98389

H -6.611355 0.979409 -16.67751

C -5.425386 -0.698449 -16.92025

C -6.120015 -3.031119 -16.518282

H -7.935573 -3.888249 -15.800114

C -5.179176 -2.152683 -16.858263

O -4.573411 0.0506426 -17.34525

O -4.019656 -2.708875 -17.343966

H -5.803519 -4.074878 -16.647662

Na -2.113666 -2.9958 -16.148297

O 0.0419037 -3.314152 -14.759798

O -1.153915 -0.697791 -16.829806

C 0.233024 -4.684378 -14.419518

C 1.70699 -4.733065 -13.969406

C 2.360567 -3.565904 -14.750371

C 1.18218 -2.953489 -15.536133

H -0.474913 -4.996942 -13.61899

H 0.0788361 -5.331446 -15.311511

H 1.789114 -4.558722 -12.871414

H 2.177735 -5.715343 -14.205897

H 2.796936 -2.82089 -14.045161

H 3.167491 -3.917466 -15.43435

H 1.259064 -1.847292 -15.63728

H 1.118145 -3.404957 -16.551831

C -1.526689 -0.398866 -18.17319

C -1.702233 0.333652 -16.014842

H -2.588162 -0.668692 -18.355955

H -0.892907 -0.96791 -18.890668

C -1.319726 1.126715 -18.281844

H -1.212893 0.343025 -15.014501

H -2.792218 0.180218 -15.86929

C -1.42671 1.620617 -16.818485

H -2.08766 1.596414 -18.938999

H -0.312368 1.359957 -18.69814

H -2.243131 2.367552 -16.685282

H -0.467556 2.08788 -16.49541

O -1.141608 -3.822606 -18.031507

C -0.67522 -4.918885 -18.226455

N -0.148584 -5.363927 -19.427771

H -0.648612 -5.681903 -17.409156

C 0.320275 -6.736576 -19.562929

C -0.221389 -4.519171 -20.615059

H -0.965373 -4.915122 -21.341776

H -0.511908 -3.470502 -20.383372

H 0.761255 -4.474729 -21.135042

H 0.449062 -7.243848 -18.580996

H -0.393314 -7.346297 -20.160518

H 1.305537 -6.774229 -20.078333

O -3.154743 -2.129361 -14.322527

C -2.652862 -1.791955 -13.277711

N -3.343991 -1.269879 -12.196643

H -1.548052 -1.885853 -13.11917

C -2.637925 -0.864937 -10.987912

C -4.786359 -1.062158 -12.279372

H -5.227428 -1.443801 -13.226877

H -5.312491 -1.578281 -11.445653

H -5.037201 0.0198577 -12.213065

H -3.045375 -1.386125 -10.093114

H -1.548853 -1.089297 -11.028423

H -2.741977 0.228883 -10.812158

O -2.906711 -5.352772 -15.443094

C -3.883172 -5.235864 -14.411881

C -3.562612 -6.023203 -16.520393

H -4.648593 -4.474962 -14.681472

H -3.409271 -4.943475 -13.447202

C -4.50889 -6.640047 -14.36367

H -2.822329 -6.447379 -17.234043

H -4.222655 -5.324007 -17.075491

C -4.395453 -7.127234 -15.827004

H -5.563145 -6.614854 -14.003393

H -3.920744 -7.303825 -13.688167

H -5.39635 -7.235892 -16.305009

H -3.874651 -8.111704 -15.874648

2(9-**O**$^{-}$)→Na^{+}(2DMF+2THF)

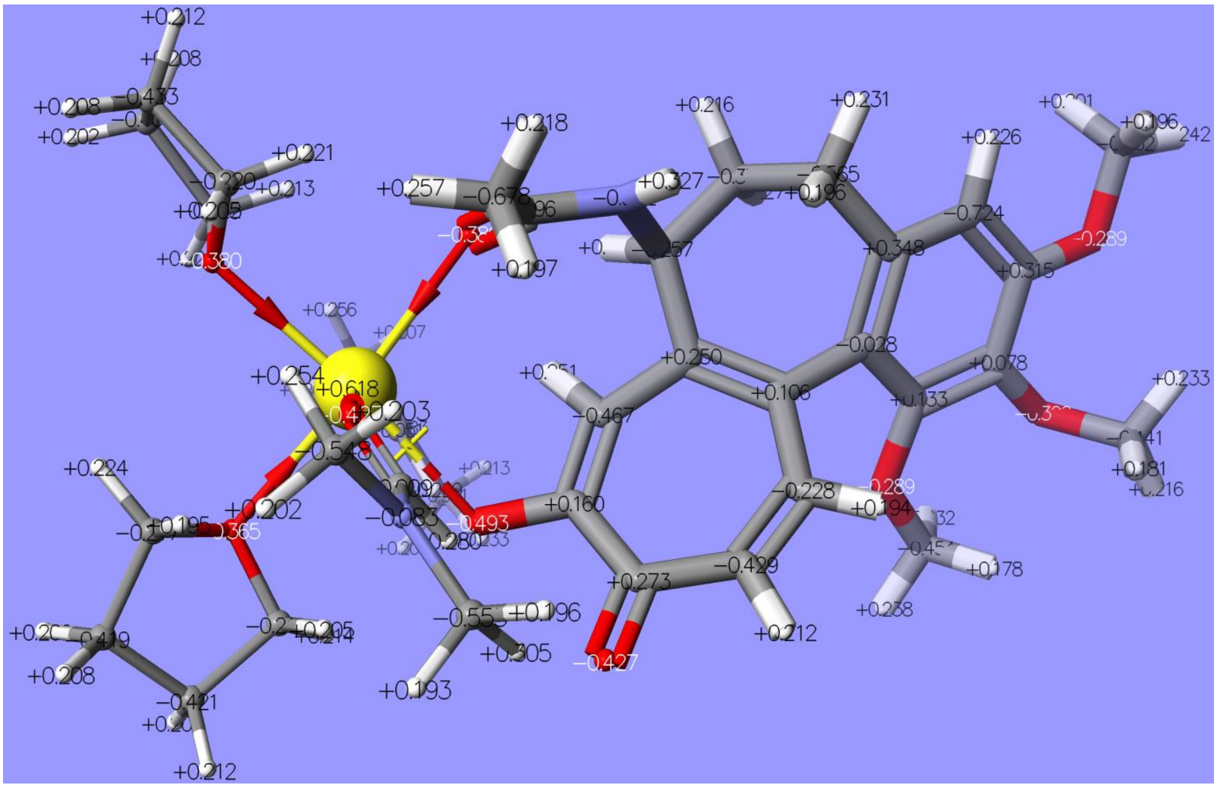

E= -2443.44 au

XYZ

C -10.724985 -1.605311 -15.861662

C -9.452475 -1.973988 -15.413875

C -9.362619 -2.862128 -14.315811

C -10.523209 -3.449635 -13.769411

C -11.788013 -3.088789 -14.248057

C -11.868792 -2.16732 -15.2904

C -10.917644 -0.458518 -16.807672

C -8.160267 -1.464438 -16.005013

O -8.092084 -3.113505 -13.858558

O -10.436647 -4.310873 -12.712047

O -12.892997 -3.629906 -13.645745

H -12.853033 -1.83647 -15.654729

C -7.770423 -3.994868 -12.795154

C -10.824582 -5.65012 -12.996645

C -14.190585 -3.238519 -14.069015

H -8.064555 -5.037934 -13.044913

H -8.245499 -3.663061 -11.845689

H -6.668559 -3.981979 -12.646264

H -10.335033 -6.015102 -13.927518

H -11.92751 -5.740242 -13.09952

H -10.506687 -6.302827 -12.154344

H -14.353714 -3.498859 -15.138891

H -14.348889 -2.148937 -13.905552

H -14.944611 -3.789112 -13.465017

C -7.8563 -0.156735 -16.172295

C -10.335127 0.786664 -16.118539

C -8.850127 1.001014 -16.424598

H -10.441135 -0.691322 -17.784011

H -11.999599 -0.293584 -17.016089

C -7.191838 -2.542481 -16.297447

C -6.47974 0.37385 -16.326709

H -10.897843 1.68611 -16.46216

H -10.497408 0.731454 -15.016698

H -8.533314 1.874255 -15.806817

N -8.972404 1.4404764 -17.8373582

H -9.4018156 0.8237568 -18.5348895

C -8.0420769 2.3418125 -18.3414022

C -7.8186274 2.203117 -19.8461189

O -7.401677 3.067574 -17.6169093

H -7.2643496 3.0707942 -20.2610156

H -7.2476701 1.2754177 -20.074881

H -8.7900925 2.1553931 -20.3869461

H -6.5558538 1.4511833 -16.1209927

C -5.251972 -0.0685233 -16.583223

C -5.913992 -2.532561 -16.665599

H -7.608423 -3.559543 -16.207755

C -4.905846 -1.480749 -16.833734

O -4.259791 0.875566 -16.746555

O -3.782233 -1.79832 -17.164575

H -5.476137 -3.526669 -16.854963

Na -4.777936 3.0887384 -17.812497

O -2.226292 3.4554274 -17.672018

O -5.058547 5.4405514 -18.842023

C -1.572346 2.2151254 -17.410653

C -0.096392 2.5167754 -17.745846

C -0.1901778 3.6718244 -18.773654

C -1.709421 3.8900094 -18.926219

H -1.719836 1.9011134 -16.35242

H -1.962837 1.4203924 -18.085818

H 0.456596 2.8475944 -16.836211

H 0.418694 1.6217414 -18.165036

H 0.302093 4.5888884 -18.374108

H 0.287257 3.4114874 -19.746463

H -1.977675 4.9535514 -19.1194

H -2.109979 3.2601354 -19.752607

C -5.994709 5.4112994 -19.91531

C -5.630743 6.3262134 -17.882333

H -6.91602 4.8654734 -19.61037

H -5.557719 4.9185274 -20.813344

C -6.312323 6.9022114 -20.140083

H -4.88124 6.6132884 -17.110149

H -6.500005 5.8411024 -17.380997

C -6.100223 7.5234984 -18.737313

H -7.350466 7.0507184 -20.517413

H -5.602588 7.3483004 -20.875003

H -7.035864 7.9728124 -18.33137

H -5.31682 8.3155934 -18.775934

O -4.507855 2.1635584 -19.871453

C -4.143472 1.0210654 -20.02225

N -3.942999 0.3803824 -21.23389

H -3.931055 0.3716864 -19.133911

C -3.483531 -1.0028456 -21.264724

C -4.149766 1.0911024 -22.488362

H -4.891642 0.5636654 -23.12823

H -4.525359 2.1279074 -22.341119

H -3.201974 1.1644334 -23.066561

H -3.390265 -1.4515046 -20.250867

H -4.049362 -1.80735 -21.704121

H -2.485655 -1.0810376 -21.750818

O -4.956594 3.9458474 -15.71566

C -4.614048 3.3124594 -14.745147

N -4.653877 3.7441914 -13.429444

H -4.212544 2.2714764 -14.861278

C -4.158102 2.8844254 -12.362291

C -5.08881 5.0973554 -13.110286

H -4.242925 5.7065994 -12.720996

H -5.506147 5.6362164 -13.989535

H -5.880856 5.0886694 -12.328832

H -3.968585 1.8415474 -12.701539

H -3.065694 3.112273 -11.809116

H -4.887745 2.8247624 -11.524377

2(10-**O**¯)→[H-MTBD]$^+$

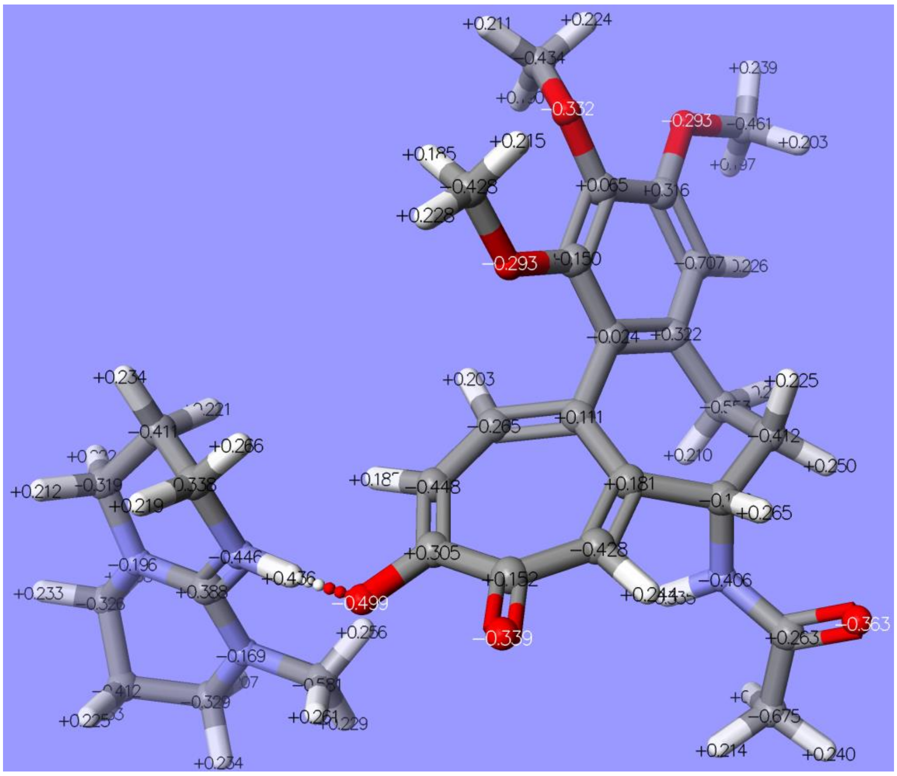

E= -1797.89 au

XYZ

C 16.513147 -0.004187 -1.141128

C 17.468232 -0.455708 -0.201528

C 17.409228 -1.840298 0.208433

C 16.433595 -2.723629 -0.332158

C 15.498137 -2.227033 -1.280923

C 15.555236 -0.884315 -1.683539

C 16.367217 1.476865 -1.438086

C 18.574153 0.425213 0.35539

O 18.352457 -2.20788 1.151795

O 16.341286 -4.025349 0.165389

O 14.549093 -3.135118 -1.732341

H 14.82123 -0.488104 -2.387777

C 18.326579 -3.459129 1.89442

C 16.29756 -5.128684 -0.784446

C 13.53719 -2.643851 -2.640334

H 18.623048 -4.313352 1.261847

H 17.340196 -3.657545 2.339458

H 19.077167 -3.308459 2.686987

H 16.84868 -4.891978 -1.71148

H 15.253892 -5.381292 -1.031207

H 16.778049 -5.98225 -0.277024

H 13.982355 -2.233448 -3.565702

H 12.907421 -1.871706 -2.159859

H 12.918222 -3.518282 -2.89011

C 18.365984 1.706574 1.023701

C 15.971905 2.203823 -0.125591

C 17.178206 2.676786 0.715481

H 17.291739 1.921554 -1.851168

H 15.582812 1.622727 -2.199097

C 19.859194 -0.111952 0.198393

C 19.2468 2.299821 1.927971

H 15.377485 3.105648 -0.343376

H 15.331351 1.540431 0.480895

H 16.775577 3.053996 1.669776

N 17.78759 3.865881 0.037231

H 18.721289 3.751099 -0.359083

C 17.297497 5.147076 0.200351

C 18.158473 6.245851 -0.445697

O 16.24215 5.390954 0.815198

H 19.164095 6.273768 0.011035

H 18.283674 6.070484 -1.529108

H 17.6692 7.218216 -0.292715

H 18.884412 3.232406 2.381646

C 20.583473 2.016917 2.466773

C 21.143475 0.268597 0.671545

H 19.896227 -1.00527 -0.435534

C 21.520505 1.145843 1.692224

O 20.990309 2.63701 3.480936

O 22.8124 1.278219 2.057116

H 21.97578 -0.284263 0.21925

C 24.783153 -2.717163 0.151468

C 24.444709 -1.61024 1.165301

N 24.501304 -0.270172 0.55097

C 25.470467 -0.05111 -0.326645

N 26.405417 -1.01947 -0.710151

C 26.188735 -2.45699 -0.413008

N 25.57321 1.212474 -0.903587

C 26.712587 1.63957 -1.740223

C 27.92987 0.728252 -1.538179

C 27.491418 -0.734824 -1.66291

C 24.563233 2.234625 -0.571759

H 24.693398 3.083309 -1.260586

H 23.544913 1.837559 -0.7055

H 24.661079 2.600545 0.466272

H 24.03492 -2.70481 -0.660786

H 24.754607 -3.714447 0.623887

H 23.4315 -1.759771 1.576505

H 25.147056 -1.668682 2.022485

H 26.338035 -3.032767 -1.346434

H 26.961741 -2.794331 0.305708

H 26.421068 1.646284 -2.810979

H 26.977528 2.677136 -1.471691

H 28.700013 0.962678 -2.292714

H 28.368161 0.895078 -0.539044

H 27.175496 -0.957399 -2.705489

H 28.336006 -1.407803 -1.428182

H 23.801102 0.440074 1.122042

2(9-**O**$^-$)→[H-MTBD]$^+$

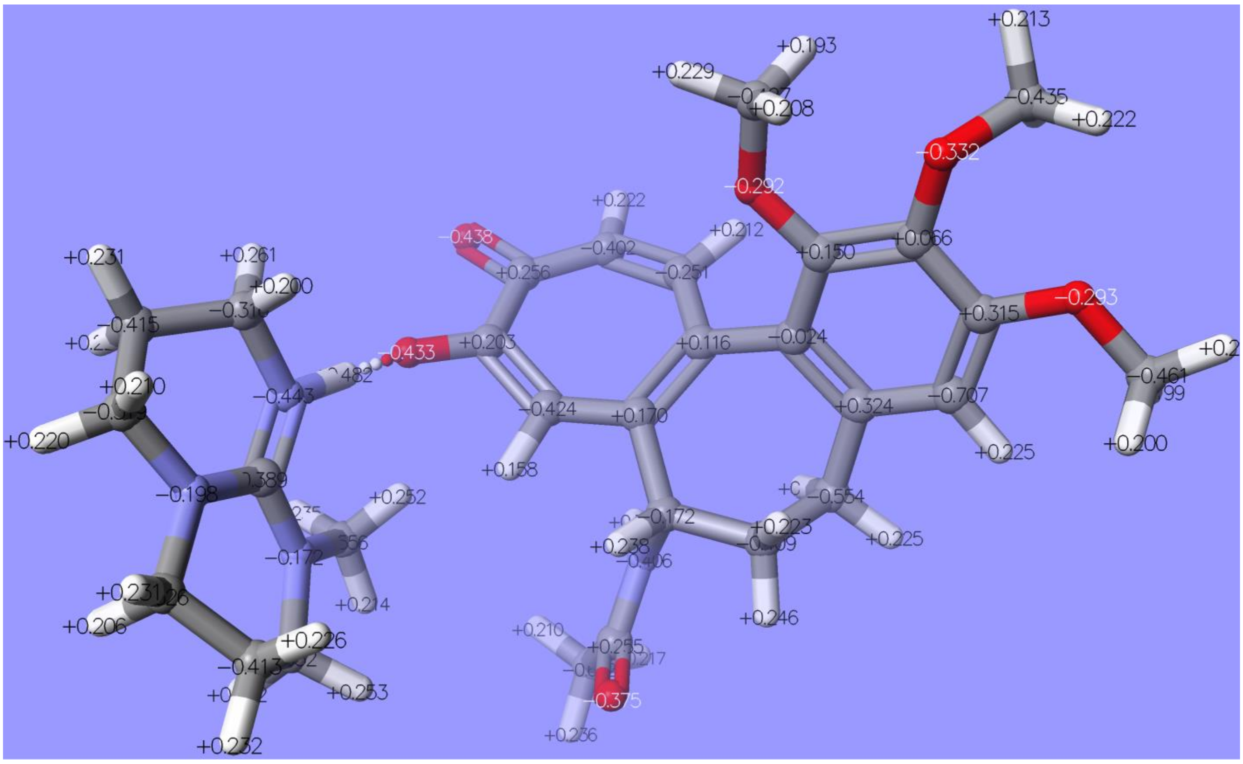

E= -1784.87 au

XYZ

C 16.5196046 -0.0050532 -1.1528828

C 17.4746896 -0.4565742 -0.2132828

C 17.4156856 -1.8411642 0.1966782

C 16.4400526 -2.7244952 -0.3439128

C 15.5045946 -2.2278992 -1.2926778

C 15.5616936 -0.8851812 -1.6952938

C 16.3736746 1.4759988 -1.4498408

C 18.5806106 0.4243468 0.3436352

O 18.3589146 -2.2087462 1.1400402

O 16.3477436 -4.0262152 0.1536342

O 14.5555506 -3.1359842 -1.7440958

H 14.8276876 -0.4889702 -2.3995318

C 18.3330366 -3.4599952 1.8826652

C 16.3040176 -5.1295502 -0.7962008

C 13.5436476 -2.6447172 -2.6520888

H 18.6295056 -4.3142182 1.2500922

H 17.3466536 -3.6584112 2.3277032

H 19.0836246 -3.3093252 2.6752322

H 16.8551376 -4.8928442 -1.7232348

H 15.2603496 -5.3821582 -1.0429618

H 16.7845066 -5.9831162 -0.2887788

H 13.9888126 -2.2343142 -3.5774568

H 12.9138786 -1.8725722 -2.1716138

H 12.9246796 -3.5191482 -2.9018648

C 18.3724416 1.7057078 1.0119462

C 15.9783626 2.2029568 -0.1373458

C 17.1846636 2.6759198 0.7037262

H 17.2981966 1.9206878 -1.8629228

H 15.5892696 1.6218608 -2.2108518

C 19.8656516 -0.1128182 0.1866382

C 19.2532576 2.2989548 1.9162162

H 15.3839426 3.1047818 -0.3551308

H 15.3378086 1.5395648 0.4691402

H 16.7820346 3.0531298 1.6580212

N 17.7940476 3.8650148 0.0254762

H 18.7277466 3.7502328 -0.3708378

C 17.3039546 5.1462098 0.1885962

C 18.1649306 6.2449848 -0.4574518

O 16.2486076 5.3900878 0.8034432

H 19.1705526 6.2729018 -7.198E-4

H 18.2901316 6.0696178 -1.5408628

H 17.6756576 7.2173498 -0.3044698

H 18.8908696 3.2315398 2.3698912

C 20.5899306 2.0160508 2.4550182

C 21.1499326 0.2677308 0.6597902

H 19.9026846 -1.0061362 -0.4472888

C 21.5269626 1.1449768 1.6804692

O 20.9967666 2.6361438 3.4691812

O 22.8188576 1.2773528 2.0453612

H 21.9822376 -0.2851292 0.2074952

C 20.6921566 2.8456228 8.1408002

C 20.5410746 2.2460248 6.7317802

N 20.1343036 3.2643688 5.7449162

C 19.2551556 4.1724188 6.1449362

N 18.7273106 4.2257308 7.4401072

C 19.3526906 3.4742518 8.5563002

N 18.8959764 5.1625387 5.261038

C 17.7147246 6.0692248 5.4902102

C 16.8347186 5.5897038 6.6515672

C 17.7261656 5.2270548 7.8441262

C 19.7248129 5.3500741 4.000776

H 20.7539581 5.5830382 4.2740669

H 19.7039005 4.4328111 3.4123109

H 19.3109446 6.1688561 3.4122107

H 21.4872246 3.6120178 8.1244282

H 20.9803266 2.0734678 8.8751732

H 21.4940596 1.8012148 6.3971082

H 19.7985706 1.4217898 6.7599862

H 19.5038506 4.1697808 9.4039342

H 18.6483516 2.6904138 8.8989302

H 18.1119416 7.0814318 5.7120262

H 17.1048036 6.1558538 4.5740562

H 16.1236266 6.3849858 6.9331212

H 16.2526676 4.7028808 6.3466692

H 18.2158156 6.1376378 8.2532702

H 17.1174626 4.7917088 8.6573182

H 20.5012326 2.9512418 4.7017622

Figure 7S. DFT calculated geometries (xyz coordinates) of **5f**.

XYZ data of hybrid 5f

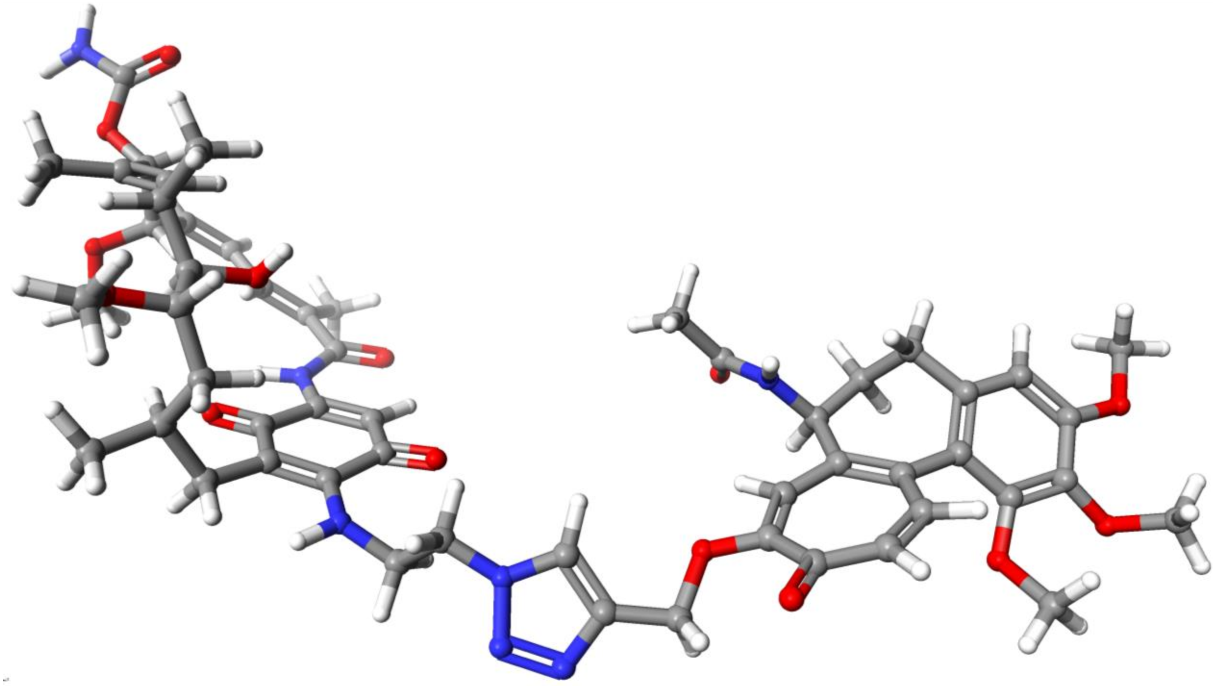

E = -3531.9480140 au

O -5.511875833279024 12.889565763030651 9.000084161491957

O -11.493981833279024 13.146701763030652 4.631696161491957

O -11.315877833279025 10.856826763030652 3.038279161491958

O -10.114992833279025 8.947796763030652 2.437590161491958

O -6.032961833279025 14.331057763030651 3.395927161491958

H -5.292856833279025 13.949565763030652 2.875826161491958

O -7.243699833279025 16.669185763030654 0.786374161491958

N -3.5080278332790242 18.606699763030655 5.985130161491957

O -2.4083338332790243 16.320161763030654 7.181249161491959

O -7.718695833279025 16.32451376303065 6.259848161491957

N -6.771876833279024 14.353932763030652 7.710460161491959

H -7.711013833279025 14.585345763030652 7.360744161491958

N -12.558445808252404 8.970226215348285 2.3456932376128306

C -6.577307833279025 13.148296763030652 8.415020161491958

C -7.7479678332790245 12.197531763030652 8.405829161491958

C -8.577584833279024 12.17205776303065 7.314367161491958

H -8.339992833279025 12.864894763030652 6.5025091614919575

C -9.718793833279024 11.298095763030652 7.058513161491959

H -9.985120833279025 10.560199763030651 7.826698161491958

C -10.485182833279024 11.33008576303065 5.9270791614919585

H -11.324862833279024 10.628170763030651 5.841186161491958

C -10.325778833279024 12.29163676303065 4.758757161491959

H -9.432285833279025 12.923274763030651 4.897023161491958

C -10.083416833279024 11.567363763030652 3.399925161491958

H -9.295816833279025 10.814805763030652 3.555219161491958

C -9.635982833279025 12.551129763030652 2.305580161491958

C -8.309763833279025 12.821789763030651 2.2550921614919583

H -7.655324833279025 12.252143763030652 2.930758161491958

C -7.564939833279024 13.875236763030651 1.4498451614919579

H -8.257101833279025 14.44028176303065 0.8085141614919581

C -6.966608833279024 14.927093763030651 2.4499281614919584

H -7.797150833279025 15.271758763030652 3.0840711614919583

C -6.3052958332790245 16.170822763030653 1.7766501614919579

H -5.392276833279024 15.821249763030652 1.2448091614919579

C -5.840655833279024 17.244766763030654 2.809872161491958

H -5.262581833279024 18.023120763030654 2.274365161491958

H -5.123452833279025 16.723990763030653 3.466400161491958

C -6.937363833279025 17.920850763030653 3.6889861614919583

H -7.678347833279025 17.161958763030654 3.9815591614919583

C -6.354467833279025 18.479784763030654 5.035960161491959

H -5.695730833279025 19.334481763030652 4.793630161491958

H -7.203190833279025 18.904496763030654 5.601373161491958

C -5.648046833279024 17.456559763030654 5.924574161491957

C -4.308860833279025 17.536992763030653 6.320879161491957

C -3.656391833279024 16.375913763030653 7.083279161491959

C -4.506621833279024 15.334158763030652 7.638766161491958

H -4.0312388332790245 14.552618763030651 8.233245161491958

C -5.844279833279025 15.309852763030651 7.339032161491959

C -6.473609833279024 16.390505763030653 6.461862161491958

C -7.807460833279024 11.256620763030652 9.593862161491957

H -6.836034833279024 10.749476763030652 9.716638161491957

H -8.596289833279025 10.49805376303065 9.490164161491958

H -7.985504833279025 11.820765763030652 10.527328161491958

C -11.504903833279025 14.234223763030652 5.584337161491959

H -12.424149833279024 14.806675763030652 5.386337161491959

H -10.626265833279025 14.89708576303065 5.450934161491958

H -11.526302833279024 13.868703763030652 6.628911161491958

C -11.176158833279025 9.55672276303065 2.5817111614919583

C -10.715432833279024 13.176856763030651 1.440000161491958

H -11.496051833279024 13.64728276303065 2.0607321614919583

H -11.219946833279025 12.399456763030651 0.8371351614919581

H -10.303271833279025 13.927650763030652 0.7500011614919581

C -6.499995833279025 13.20445176303065 0.5353211614919581

H -5.757210833279024 12.626721763030652 1.116271161491958

H -5.959900833279025 13.939150763030652 -0.08664083850804202

H -6.991355833279025 12.487064763030652 -0.142407838508042

C -6.630213833279025 17.432841763030652 -0.268226838508042

H -7.434135833279025 17.696311763030653 -0.974860838508042

H -5.860333833279024 16.837285763030653 -0.802568838508042

H -6.162807833279024 18.366527763030653 0.10265716149195797

C -7.684080833279024 19.060778763030655 2.951124161491958

H -6.989448833279025 19.868808763030653 2.649943161491958

H -8.456469833279025 19.510259763030653 3.601115161491958

H -8.180276833279025 18.682557763030655 2.0437031614919583

C -2.239951833279024 19.048135763030654 6.612488161491958

H -2.311172833279024 20.133722763030654 6.787302161491958

C -1.033888833279024 18.751303763030652 5.685885161491958

H -2.094555833279024 18.548128763030654 7.576087161491959

H -13.389232885169697 9.53339380862684 2.5274488281955128

H -12.655248350706154 8.013493311352637 2.005558825671697

H -3.9862648332790247 19.340205763030653 5.463672161491958

H -1.132347833279024 19.301495763030655 4.733170161491959

H -0.993196833279024 17.674206763030654 5.478677161491959

N 0.24027216672097612 19.131271763030654 6.325784161491958

N 0.3963561667209761 20.363944763030652 6.914305161491958

N 1.6509331667209761 20.430976763030653 7.357256161491959

C 2.299717166720976 19.257079763030653 7.043743161491959

C 1.4084221667209762 18.416662763030654 6.383996161491957

C 3.756006166720976 19.033352763030653 7.373190161491959

H 1.5111951667209762 17.411142763030654 5.982909161491959

H 3.951137166720976 19.249749763030653 8.439604161491959

H 4.409755166720975 19.655614763030652 6.7468411614919575

C 8.822969166720975 12.671317763030652 8.085419161491957

C 8.827419166720976 14.073599763030652 8.283385161491958

C 9.963760166720975 14.64794976303065 8.950379161491957

C 11.068371166720976 13.857915763030652 9.360470161491957

C 11.047757166720976 12.457332763030651 9.102451161491958

C 9.928361166720975 11.887030763030651 8.469797161491957

C 7.553778166720975 11.970327763030651 7.644135161491958

C 7.741314166720976 14.998979763030652 7.786040161491957

O 9.854325166720976 15.986728763030651 9.286843161491957

O 12.055370166720975 14.456558763030651 10.139837161491958

O 12.133418166720976 11.731618763030651 9.563400161491957

H 9.878611166720976 10.80794276303065 8.307597161491957

C 11.004815166720975 16.872410763030654 9.162649161491958

C 13.430666166720975 14.353159763030652 9.664348161491958

C 12.088399166720976 10.290660763030651 9.426607161491958

H 11.648439166720975 16.583211763030654 8.312504161491958

H 11.596874166720976 16.878747763030653 10.089933161491958

H 10.578891166720975 17.871315763030655 8.973208161491957

H 13.508090166720976 14.665581763030652 8.605505161491958

H 13.812696166720976 13.32652776303065 9.774971161491958

H 14.012375166720975 15.043380763030651 10.295434161491958

H 12.042458166720975 9.986211763030651 8.364635161491957

H 11.228212166720976 9.862083763030652 9.973229161491957

H 13.025663166720975 9.925365763030651 9.872081161491957

C 6.354447166720975 14.818280763030652 7.9555781614919585

C 6.471578166720976 12.242688763030651 8.725359161491959

C 5.644418166720976 13.51993776303065 8.453560161491957

H 7.202497166720976 12.323564763030651 6.656611161491957

H 7.738340166720976 10.888376763030651 7.546914161491959

C 8.284413166720975 16.130558763030653 7.049164161491959

C 5.354228166720976 15.832544763030652 7.715251161491958

H 5.750288166720975 11.41250676303065 8.790147161491957

H 6.955012166720976 12.315404763030651 9.714177161491957

H 5.096347166720975 13.756568763030652 9.382038161491957

N 4.5809701667209755 13.200808763030652 7.456788161491959

H 4.761736166720976 13.401287763030652 6.472644161491958

C 3.382866166720976 12.606816763030652 7.812807161491959

C 2.417911166720976 12.331437763030651 6.651454161491959

O 3.1142171667209757 12.305975763030652 8.989857161491958

H 2.538854166720976 13.037913763030652 5.811766161491958

H 2.582998166720976 11.30805676303065 6.270096161491958

H 1.386890166720976 12.388548763030652 7.0311291614919575

H 4.357764166720975 15.53942676303065 8.065421161491958

C 5.303939166720975 17.10053476303065 7.150774161491958

C 7.744403166720976 17.256960763030655 6.468977161491958

H 9.356147166720975 16.02691376303065 6.852452161491959

C 6.391352166720975 17.81014676303065 6.430645161491958

O 4.0178861667209755 17.609618763030653 7.120893161491958

O 6.163440166720975 18.855031763030652 5.762198161491957

H 8.439755166720975 17.880182763030653 5.891270161491958

EXPERIMENTAL SECTION:

General Experimental:

Colchicine, geldanamycin and $CDCl_3$, DMSO-d_6 for spectroscopic measurements, NaH, K_2CO_3, *N*,*N*,*N'*,*N'*-Tetramethylguanidine (TMG), 1,5,7-triazabicyclo[4.4.0]dec-5-ene (TBD), 7-Methyl-1,5,7-triazabicyclo[4.4.0]dec-5-ene (MTBD), *N,N,N',N',N'',N''*-Hexamethylphosphorimidic triamide (Phosphazene base P_1-H), *N'',N''''*-1,8-Naphthalenediylbis*(N,N,N',N'*-tetramethyl-guanidine) (TMGN), benzyl bromide, 4-iodobenzyl bromide, 3-bromo-1-phenyl-1-propene, allyl bromide, crotyl bromide, propargyl bromide, ethyl bromoacetate, 4-bromo-1-butene, 1-[(3-azidopropane)sulfonyl]-4-methylbenzene, 4-vinylbenzyl chloride, 4-iodobenzylamine, propargyl chloroformate, 4-nitrobenzyl azide, 4-cyanobenzyl azide, benzyl azide, 2-acetamido-2-deoxy-β-D-glucopyranosyl azide 3,4,6-triacetate, methanol, THF, DMF, toluene, acetone, xylene, CuOAc, ascorbic acid, TBA, $Pd(OAc)_2$, used for the syntheses of new colchiceine derivatives and performed test reactions, $CH_3COOC_2H_5$, DCM, NaCl, H_2O HPLC gradient grade, CH_3CN HPLC gradient grade were purchased.

Colchiceine was synthesized from colchicine by acidic hydrolysis in the mild conditions.[3]

To obtain new derivative of colchiceine **5f**, **6f**, **7d**, **8d**, new derivatives of geldanamycin (**9** and **10**), were synthesized Scheme. 1S.

HPLC separations

For HPLC separations the following settings were used:

<u>Method HPLC 1</u>: C18 250×4.6 mm (5 µm, carbon load 17%, pH 2 to 13) column at 25 ºC was used. The flow rates was 0.75 mL/min with injection volumes of 5 µL. Mixtures of water, acetonitrile and 0.05% NH_3(aq) at 30:45:25 H2O/CH3CN/buffer was used as the mobile phase. The analytical wavelengths was λ_{max} = 220 and 341 nm. Used for compounds: **3a-3j** and **4a-4j**.

<u>Method HPLC 2</u>: C18 150×4.6 mm (5 µm, carbon load 11%, pH 1 to 11) column at 25 ºC was used. The flow rate was 1 mL/min with injection volumes of 5 µL. Mixtures of water and acetonitrile in gradient ratio: 90:10 to 60:40 by 10 min and 60:40 system solvent was continue to 25 min. The analytical wavelengths was λmax= 220 and 341 nm. Used for compounds: **3a-3j** and **4a-4j** as well as **7d** and **8d**.

<u>Method HPLC 3</u>: C18 150×4.6 mm (5 µm, carbon load 11%, pH 1 to 11) column at 25 ºC was used. The flow rate was 0.5 mL/min with injection volumes of 5 µL. Mixtures of water and acetonitrile in 60:40 solvent system was used as a mobile phase. The analytical wavelengths was λmax= 220 and 260 nm. Used for compounds: **5a-5c**, **5f**, **6a-6c**, **6f**, **9** and **10**.

Method HPLC 4: C18 150×4.6 mm (5 µm, carbon load 11%, pH 1 to 11) column at 25 ºC was used. The flow rate was 1 mL/min with injection volumes of 5 µL. Mixtures of water and acetonitrile in gradient ratio: 90:10 to 50:40 by 5 min and 50:50 solvent system was continue to 9 min. Then solvent system was changed to 20:80 by 3 min and 20:80 system solvent was continue to 20 min. The analytical wavelengths was λmax= 220 and 341 nm. Used for compounds: **7a-7c** and **8a-8c.**

All retention times (Rt) are given in minutes.

FT-IR measurements

The FT-IR spectra of colchiceine and its new derivatives as well as geldanamycin derivatives were recorded in KBr pellet or CH_2Cl_2 solution. FT-IR measurements were performed at spectrometer equipped with a DTGS detector and two-columnar purge gas generator at resolution 1 cm^{-1}, NSS = 150, range 4000-400 cm^{-1}. The Happ-Genzel apodization function was used.

NMR measurements

The ^1H and ^{13}C measurements of colchiceine and its new derivatives as well as geldanamycin derivatives were performed in $CDCl_3$ or DMSO-d_6 at frequency 600 MHz, 500 MHz and 400 MHz spectrometers.

For 600 MHz spectrometer: the operating frequencies for ^1H measurements was 600.08 MHz; pulse width corresponding to the flip angle of 45^0; spectral width swh = 9842.5 Hz; acquisition time at= 0.2 sec; relaxation delay d_1=1.0 s; T = 293.0 K, TMS was used as the internal standard. No window function or zero filling were used. Digital resolution was 0.2 Hz/point. ^{13}C NMR spectra were recorded at the operating frequency 150.454 MHz; pulse width corresponding to the flip angle of 60^0; sw = 19000 Hz; at = 1.8 s; d_1=1.0 s; T = 293.0 K and TMS as the internal standard. Line broadening parameters of 0.5 or 1 Hz were applied

For 500 MHz spectrometer: the operating frequencies for ^1H measurements was 500.25 MHz; pulse width corresponding to the flip angle of 45^0; spectral width swh = 11029.4 Hz; acquisition time at= 0.2 sec; relaxation delay d_1=1.0 s; T = 257.0 K, TMS was used as the internal standard. No window function or zero filling were used. Digital resolution was 0.2 Hz/point. ^{13}C NMR spectra were recorded at the operating frequency 125.79 MHz; pulse width corresponding to the flip angle of 60^0; sw = 35714.3 Hz; at = 2.0 s; d_1=0.92 s; T = 257.0 K and TMS as the internal standard. Line broadening parameters of 0.5 or 1 Hz were applied.

For 400 MHz spectrometer: the operating frequencies for ^1H measurements was 402.65 MHz; pulse width corresponding to the flip angle of 45^0; spectral width swh = 8064.5 Hz; acquisition time at= 5 sec; relaxation delay d_1=1.0 s; T = 293.0 K, TMS was used as the internal standard. No window function or zero filling were used. Digital resolution was 0.2 Hz/point. ^{13}C NMR spectra were recorded at the operating frequency 101.26 MHz; pulse

width corresponding to the flip angle of 60^0; sw = 26041.7 Hz; at = 1.3 s; d_1=1.0 s; T = 293.0 K and TMS as the internal standard. Line broadening parameters of 0.5 or 1 Hz were applied.

The ^{1}H and ^{13}C NMR resonances in solution were unambiguously assigned on the basis of the HMBC, HSQC, COSY and NOESY correlation spectra.

DFT calculations

DFT calculations with DGauss using the B88-LYP GGA energy functional with the DZVP basis sets of energetically the most favorable structures of new hybrid derivatives of **2** were performed in the gas phase with the use of *Scigress* F.J. 2.4 package (version EU 3.1.8, 2008-2014)[2]. Initial structures of analysed derivatives were assumed on ^{1}H-^{1}H NOESY contacts, after initial structural optimization (MM3 and MO-G PM6 semiempirical methods of *Scigress* package) the local minimum searches were performed with the use of B88-LYP DFT method at the gradient not exciding 5 a.u. at one step. Assumed structures of complexes between **2** and MTBD as well as Na^+ were calculated *via* initial optimization with semi-empirical PM6 method[2] and next with B88-LYP GGA[2] energy functional with the DZVP basis sets, at the gradient not exciding 1 a.u. All of geometries were verified as local minima (possessing no imaginary frequencies). The lowest energy structures of local-minimum energy, which are in total agreement with ^{1}H-^{1}H NOESY data were shown in Figs. 2, 4-6 and their xyz coordinates were placed in SI section.

Biological assay

Human cancer cells SKBR-3 (human breast cancer cell line) and SKOV-3 (ovarian cancer cell line) were cultured in McCoy's Modified Medium. Human cancer cells PC-3 (human prostate cancer cell line) were cultured in F-12K medium. U-87MG cells (glioblastoma cell line) were cultured in Eagle's Minimal Essential Medium. Human Dermal Fibroblasts cell line (HDF) was cultured in Fibroblast Basal Medium. Each medium was supplemented with 10% fetal bovine serum, 1% L-glutamine, and 1% penicillin/streptomycin solution. The cell lines were kept in the incubator at 37°C. The optimal plating density of cell lines was determined to be 5×10^4. All the cell lines and mediums were obtained from American Type Culture Collection (ATCC) supplied by LGC-Standards. The protein-staining SRB (Sigma-Aldrich) microculture colorimetric assay, developed by the National Cancer Institute (USA) for in vitro antitumor screening was used in this study, to estimate the cell number by providing a sensitive index of total cellular protein content, being linear to cell density. The monolayer cell culture was trypsinized and the cell count was adjusted to 5×10^4 cells. To each well of the 96 well microtiter plate, 0.1 mL of the diluted cell suspension (approximately 10,000 cells) was added. After 24 hours, when a partial monolayer was formed, the supernatant was washed out and 100 μL of different concentrations of tested compounds (from 10nM to 2 x 10^5nM) were added to the cells in microtitre plates. The tested compounds were dissolved in DMSO (containing 10 % of water) (100 μL) and the content of DMSO did not exceed 0.1%; this concentration was found to be nontoxic to the cell lines. The cells were exposed to compounds for 72 hours at 37 °C in a humidified atmosphere (90% RH) containing 5% CO_2.

After that, 25 μL of 50 % trichloroacetic acid was added to the wells and the plates were incubated for 1 hour at 4 °C. The plates were then washed out with the distilled water to remove traces of medium and next dried by the air. The air-dried plates were stained with 100 μL of 0.4% sulforhodamine B (prepared in 1 % acetic acid) and kept for 30 minutes at room temperature. The unbound dye was removed by rapidly washing with 1% acetic acid and then air dried overnight. The protein-bound dye was dissolved in 100 μL of 10 mM unbuffered Tris base (pH 10.5) for optical density determination at 490 nm. All cytotoxicity experiments were performed three times. Cell survival was measured as the percentage absorbance compared to the control (nontreated cells). Cytarabine (**ara-C**), actinomycin D and mitomycin C were used as the internal standards. Additionally biological assays were performed in Human Dermal Fibroblasts cell line (HDF) in aim to evaluate cytotoxicity of **1- 8** analogs in healthy cells. Results of anticancer studies of 1 -8 analogs are shown in Table 2. SI indexes were calculated from equation SI = IC_{50} normal cell line HDF/IC_{50} respective cancerous cell line. A beneficial SI > 1.0 indicates a compound with efficacy against tumor cell greater than the toxicity against normal cells.

S_N2 reaction tests:

Method 1. Compound 2 (10 mg, 0.026 mmol) was dissolved in the mixture of 1 mL THF with 0.5 mL DMF or in the mixture of 0.75 mL THF with 0.75 mL DMF or in 1.5 mL DMF. To this mixture 0.078 mmol one of the listed bromide: benzyl bromide, 4-iodobenzyl bromide, 3-bromo-1-phenyl-1-propene, allyl bromide, crotyl bromide, propargyl bromide or ethyl bromoacetate was added. After cooling the reaction mixture to 0 ºC NaH (0.62 mg, 0.026 mmol) was added . The mixture was stirred at 70 ºC for 240 minutes. Every 60 minutes 5 μL sample of reaction mixture was taken to check the reaction progress (before injection extraction in the solvent system EtOAc:$HCl_{(0.1\ M)}$ was performed, the organic layer was evaporated to dryness and the residue was dissolved in acetonitrile). All HPLC analysis were performed with the use of methods HPLC 1 and HPLC 2.

Method 2. Compound 2 (10 mg, 0.026 mmol) was dissolved in toluene (1.5 ml). To this mixture benzyl bromide (22.2 mg, 0.078 mmol) and K_2CO_3 (10.5 mg, 0.078 mmol) were added. The mixture was stirred at 70 ºC for 240 minutes. Every 60 minutes 5 μL sample of reaction mixture was taken to check the reaction progress (before injection extraction in the solvent system EtOAc:$HCl_{(0.1\ M)}$ was performed, the organic layer was evaporated to dryness and the residue was dissolved in acetonitrile). THF (66 ºC), acetone (56 ºC) or DMF (115 ºC) were also used as a solvent. All HPLC analysis were performed with the use of methods HPLC 1 and HPLC 2.

Method 3. Compound 2 (10 mg, 0.026 mmol) was dissolved in toluene (1.5 ml). To this mixture 0.078 mmol one of the listed bromide: benzyl bromide, 4-iodobenzyl bromide, 3-bromo-1-phenyl-1-propene, allyl bromide, crotyl bromide, propargyl bromide or ethyl bromoacetate and MTBD (8 mg, 0.052 mmol) were added. The mixture was stirred at 70 ºC for 240 minutes. Every 60 minutes 5 μL sample of reaction mixture was taken to check the reaction progress (before injection extraction in the solvent system EtOAc:$HCl_{(0.1\ M)}$ was

performed, the organic layer was evaporated to dryness and the residue was dissolved in acetonitrile). For BnBr also another bases were used: TMG, Phosphazene base P1-H, DMAN or TBD. In the BnBr case THF (66 ºC), acetone (56 ºC), DMF (115 ºC), ACN (70 ºC) or xylene (115 ºC) were also used as a solvent in the reaction performed with MTBD as a base. All HPLC analysis were performed with the use of methods HPLC 1 and HPLC 2.

To obtain 3-type and 4-type compounds in the larger scale the same conditions basis on the method 2 were used.

Synthesis method and spectroscopic data of colchiceine derivatives

Colchiceine 150 mg (0.39 mmol) was dissolved in a 15 mL toluene and 1.17 mmol one of the listed bromide: benzyl bromide (**3a**, **4a**), 4-iodobenzyl bromide (**3b**, **4b**), 3-bromo-1-phenyl-1-propene (**3c**, **4c**), allyl bromide (**3d**, **4d**), crotyl bromide (**3e**, **4e**), propargyl bromide (**3f**, **4f**), ethyl bromoacetate (**3g**, **4g**), 4-bromo-1-butene (**3h**, **4h**) or 1-[(3-azidopropane)sulfonyl]-4-methylbenzene (**3i**, **4i**) and 4-vinylbenzyl chloride (**3j**, **4j**) were added. To each mixture, 157.5 mg (1.17 mmol) of K_2CO_3 was added. The solution was stirred at 70 ºC for a 4 h (for compounds **3h**-**3j** and **4h-4j** for 24 h) and after this time, diethyl ether was added and extracted twice with 25 mL of water. The organic layer was evaporated and the synthesized derivative **3a-3j** and **4a-4j** was purified by column chromatography with silica gel with dichloromethane/acetone (4:1 to 1:1) as an eluent. The products were obtained as a cream-coloured to little yellow powders. Yields are given after isolation.

(*S*)-*N*-(9-(benzyloxy)-1,2,3-trimethoxy-10-oxo-5,6,7,10-tetrahydrobenzo[*a*]heptalen-7-yl)acetamide (compound **3a**):Yield: 50%; HPLC Rt = 6.612 (HPLC method 1) and 13.118 (HPLC method 2), Anal. Calcd $C_{28}H_{29}NO_6$: C, 70.72; H, 6.15; N, 2.95. Found: C, 70.71; H, 6.17; N, 2.94; HRMS (ESI-TOF) m/z: $[M + Na]^+$ Calcd for $C_{28}H_{29}NNaO_6^+$ 498.1887; Found 498.1889. ^{1}H NMR (500 MHz, $CDCl_3$, T = 298 K) δ 7.42 (d, $^3J_{H11,H12}$ = 12.8 Hz, 1H, H-12), 7.40 – 7.35 (m, 2H, H-20 + H-24), 7.34 – 7.29 (m, 2H, H-21 + H-23), 7.29 – 7.23 (m, 1H, H-22), 7.19 (s, 1H, H-8), 7.15 (d, $^3J_{H11,H12}$ = 12.8 Hz, 1H, H-11), 6.95 (d, $^3J_{H7,NH}$ = 6.4 Hz, 1H, 7-NH), 6.54 (s, 1H, H-4), 5.28 (s, 2H, H-18), 4.51 (dt, $^3J_{H6b, H7}$ = 12.7 Hz, $^3J_{H6a, H7}$ = 6.4 Hz, $^3J_{H7,NH}$ = 6.4 Hz, 1H, H-7), 3.93 (s, 3H, H-16), 3.89 (s, 3H, H-17), 3.66 (s, 3H, H-15), 2.43 (dd, ^2J = 13.4 Hz, $^3J_{H5b,H6a}$ = 6.4 Hz, 1H, H-5b), 2.27 (td, ^2J = 13.1 Hz, $^3J_{H5a, H6a}$ = 13.1 Hz, $^3J_{H5a, H6b}$ = 7.3 Hz, 1H, H-5a), 2.16 (tt, ^2J = 12.8, $^3J_{H5a,H6a}$ = 12.8 Hz, $^3J_{H5b,H6a}$ = 6.3 Hz, $^3J_{H6a, H7}$ = 6.3 Hz, 1H, H-6a), 1.92 – 1.81 (m, 1H, H-6b), 1.81 (s, 3H, H-14), ^{13}C NMR (126 MHz, $CDCl_3$, T = 298 K) δ 179.6 (C-10), 169.9 (C-13), 163.1 (C-9), 153.7 (C-3), 151.1 (C-1), 144.4 (C-7a), 141.7 (C-2), 141.3 (C-12), 135.9 (C-19), 135.2 (C-12a), 134.9 (C-4a), 134.3 (C-11), 128.9 (C-21 + C-23), 128.3 (C-22), 127.2 (C-20 + C-24), 125.9 (C-1a), 112.9 (C-8), 107.6 (C-4), 71.1 (C-18), 61.6 (C-15), 61.5 (C-16), 56.2 (C-17), 52.7 (C-7), 38.3 (C-6), 30.0 (C-5), 22.8 (C-14).

(*S*)-*N*-(9-((4-iodobenzyl)oxy)-1,2,3-trimethoxy-10-oxo-5,6,7,10-tetrahydrobenzo[*a*]heptalen-7-yl)acetamide (compound **3b**): Yield: 49%; HPLC Rt = 11.111 (HPLC method 1) and 17.018 (HPLC method 2), Anal. Calcd $C_{28}H_{28}INO_6$: C, 55.92; H, 4.69; I, 21.10; N, 2.33. Found C, 55.94; H, 4.68; I, 20.11; N, 2.32; HRMS (ESI-TOF) m/z: $[M + Na]^+$ Calcd for

$C_{28}H_{28}INNaO_6^+$ 624.0854; Found 624.0856. ^{1}H NMR (401 MHz, $CDCl_3$, T = 298 K) δ 7.66 (d, ^3J $_{H20,H21 \text{ and } H23,H24}$ = 8.3 Hz, 2H, H-21 + H-23), 7.42 (d, ^3J $_{H11,H12}$ = 12.8 Hz, 1H, H-12), 7.15 (d, ^3J $_{H11,H12}$ = 12.8 Hz, 1H, H-11), 7.13 (d, ^3J $_{H20,H21 \text{ and } H23,H24}$ = 8.3 Hz, 2H, H-20 + H-24), 7.07 (s, 1H, H-8), 6.59 (d, $^3J_{H7,NH}$ = 6.3 Hz, 1H, 7-NH), 6.54 (s, 1H, H-4), 5.32 – 5.04 (m, 2H, H-18), 4.51 (dt, $^3J_{H6b, H7}$ = 12.4 Hz, $^3J_{H6a, H7}$ = 6.2 Hz, ^3J $_{H7,NH}$ = 6.2 Hz, 1H, H-7), 3.92 (s, 3H, H-16), 3.89 (s, 3H, H-17), 3.65 (s, 3H, H-15), 2.45 (dd, ^2J = 13.0 Hz, $^3J_{H5b,H6a}$ = 6.1 Hz, 1H, H-5b), 2.28 (td, ^2J = 12.7 Hz, $^3J_{H5a, H6a}$ = 12.7 Hz, $^3J_{H5a, H6b}$ = 6.8 Hz, 1H, H-5a), 2.19 (tt, ^2J = 12.7, $^3J_{H5a,H6a}$ = 12.7 Hz, $^3J_{H5b,H6a}$ = 6.2 Hz, $^3J_{H6a, H7}$ = 6.2 Hz, 1H, H-6a), 1.92 – 1.83 (m, 1H, H-6b), 1.83 (s, 3H, H-14), ^{13}C NMR (101 MHz, $CDCl_3$, T = 298 K) δ 179.6 (C-10), 169.8 (C-13), 162.8 (C-9), 153.9 (C-3), 151.1 (C-1), 143.9 (C-7a), 141.8 (C-2), 141.4 (C-12), 138.0 (C-21 + C-23), 135.8 (C-19), 135.6 (C-12a), 134.8 (C-4a), 134. 7 (C-11), 129.0 (C-20 + C-24), 125.9 (C-1a), 113.3 (C-8), 107.6 (C-4), 93.9 (C-22), 70.5 (C-18), 61.6 (C-15), 61.5 (C-16), 56.3 (C-17), 52.8 (C-7), 38.5 (C-6), 30.0 (C-5), 22.9 (C-14).

(*S,E*)-*N*-(9-(cinnamyloxy)-1,2,3-trimethoxy-10-oxo-5,6,7,10-tetrahydrobenzo[*a*]heptalen-7-yl)acetamide (compound **3c**): Yield: 46%; HPLC Rt = 8.865 (HPLC method 1) and 15.618 (HPLC method 2), Anal. Calcd $C_{30}H_{31}NO_6$: C, 71.84; H, 6.23; N, 2.79. Found C, C, 71.83; H, 6.24; N, 2.81; HRMS (ESI-TOF) m/z: $[M + Na]^+$ Calcd for $C_{30}H_{31}NNaO_6^+$ 524.2044; Found 524.2041. ^{1}H NMR (500 MHz, Chloroform-*d*) δ 7.42 (d, ^3J $_{H11,H12}$ = 12.8 Hz, 1H, H-12), 7.37 (d, ^3J $_{H22,H23 \text{ and } H25,H26}$ = 7.1 Hz, 2H, H-22 + H-26), 7.30 (t, ^3J $_{H22,H23 \text{ and } H25,H26}$ = 7.4 Hz, ^3J $_{HH23,24 \text{ and } H24,H26}$ = 7.4 Hz, 2H, H-23 + H-25), 7.25 (t, ^3J $_{HH23,24 \text{ and } H24,H26}$ = 7.4 Hz, 1H, H-24), 7.18 (s, 1H, H-8), 7.16 (d, ^3J $_{H11,H12}$ = 13.1 Hz, 1H, H-11), 6.72 (d, $^3J_{H19, H20}$ = 16.0 Hz, 1H, H-20), 6.52 (s, 1H, H-4), 6.46 (d, $^3J_{H7,NH}$ = 6.1 Hz, 1H, 7-NH), 6.44 (dt, $^3J_{H19, H20}$ = 16.0 Hz, $^3J_{H18, H19}$ = 5.7 Hz, 1H, H-19), 4.99 – 4.83 (m, 2H, H-18), 4.58 (dt, $^3J_{H6b, H7}$ = 12.4 Hz, $^3J_{H6a, H7}$ = 6.2 Hz, ^3J $_{H7,NH}$ = 6.2 Hz, 1H, H-7), 3.93 (s, 3H, H-16), 3.89 (s, 3H, H-17), 3.67 (s, 3H, H-15), 2.47 (dd, ^2J = 13.3 Hz, $^3J_{H5b,H6a}$ = 6.3 Hz, 1H, H-5b), 2.35 (td, ^2J = 12.8 Hz, $^3J_{H5a, H6a}$ = 12.8 Hz, $^3J_{H5a, H6b}$ = 6.9 Hz, 1H, H-5a), 2.26 (tt, ^2J = 12.6, $^3J_{H5a,H6a}$ = 12.6 Hz, $^3J_{H5b,H6a}$ = 6.2 Hz, $^3J_{H6a, H7}$ = 6.2 Hz, 1H, H-6a), 2.00 – 1.91 (m, 1H, H-6b), 1.90 (s, 3H, H-14), ^{13}C NMR (126 MHz, $CDCl_3$) δ 179.7 (C-10), 169.8 (C-13), 163.2 (C-9), 153.8 (C-3), 151.2 (C-1), 143.8 (C-7a), 141.8 (C-2), 141.2 (C-12), 136.1 (C-12a), 135.1 (C-4a), 134.8 (C-11), 134.5 (C-21), 133.9 (C-20), 128.8 (C-22 + C-26), 128.4 (C-24), 126.7 (C-23 + C-25), 126.0 (C-1a), 123.3 (C-19), 112.3 (C-8), 107.5 (C-4), 70.1 (C-18), 61.63 (C-15), 61.56 (C-16), 56.2 (C-17), 53.0 (C-7), 38.6 (C-6), 30.0 (C-5), 23.0(C-14).

(*S*)-*N*-(9-(allyloxy)-1,2,3-trimethoxy-10-oxo-5,6,7,10-tetrahydrobenzo[*a*]heptalen-7-yl)acetamide (compound **3d**): Yield: 53%; HPLC Rt = HPLC Rt = 4.454 (HPLC method 1) and 10.725 (HPLC method 2), Anal. Calcd $C_{24}H_{27}NO_6$: C, 67.75; H, 6.40; N, 3.29. Found: C, 67.76; H, 6.38; N, 3.30; HRMS (ESI-TOF) m/z: $[M + Na]^+$ Calcd for $C_{24}H_{27}NNaO_6^+$ 448.1731; Found 448.1729. ^{1}H NMR (500 MHz $CDCl_3$, T = 298 K) δ 7.41 (d, ^3J $_{H11,H12}$ = 12.7 Hz, 1H, H-12), 7.17 (s, 1H, H-8), 7.13 (d, ^3J $_{H11,H12}$ = 12.8 Hz, 1H, H-11), 7.04 (d, $^3J_{H7,NH}$ = 6.3 Hz, 1H, 7-NH), 6.55 (s, 1H, H-4), 6.04 (ddt, $^3J_{H19, H20trans}$ = 17.3 Hz, $^3J_{H19, H20cis}$ = 10.5 Hz, ^3J $_{H18,H19}$ = 5.2 Hz, 1H, H-19), 5.40 (dq, $^3J_{H19, H20trans}$ = 17.3 Hz, ^2J = 1.5 Hz, ^4J $_{H20trans,H18}$ = 1.5 Hz, 1H, 20_{trans}-H), 5.40 (dq, $^3J_{H19, H20cis}$ = 10.5 Hz, ^2J = 1.3 Hz, ^4J $_{H20cis,H18}$ = 1.3 Hz, 1H, 20_{cis}-H), 4.76 (m, 2H, H-18), 4.58 (dt, $^3J_{H6b, H7}$ = 12.5 Hz, $^3J_{H6a, H7}$ = 6.3 Hz, ^3J $_{H7,NH}$ = 6.3 Hz, 1H,

H-7), 3.92 (s, 3H, H-16), 3.89 (s, 3H, H-17), 3.65 (s, 3H, H-15), 2.49 (dd, ^{2}J = 13.2 Hz, $^{3}J_{H5b,H6a}$ = 6.3 Hz, 1H, H-5b), 2.35 (td, ^{2}J = 12.8 Hz, $^{3}J_{H5a, H6a}$ = 12.8 Hz, $^{3}J_{H5a, H6b}$ = 6.9 Hz, 1H, H-5a), 2.26 (tt, ^{2}J = 12.6, $^{3}J_{H5a,H6a}$ = 12.6 Hz, $^{3}J_{H5b,H6a}$ = 6.6 Hz, $^{3}J_{H6a, H7}$ = 6.6 Hz, 1H, H-6a), 2.03 – 1.97 (m, 1H, H-6b), 2.02 (s, 3H, H-14), ^{13}C NMR (126 MHz, $CDCl_3$, T = 298 K) δ 179.6 (C-10), 170.0 (C-13), 162.9 (C-9), 153.8 (C-3), 151.1 (C-1), 144.4 (C-7a), 141.7 (C-2), 141.3 (C-12), 135.0 (C-12a), 134.9 (C-4a), 134.2 (C-11), 132.3 (C-19), 126.0 (C-1a), 118.7 (C-20), 112.3 (C-8), 107.6 (C-4), 69.9 (C-18), 61.6 (C-15), 61.5 (C-16), 56.2 (C-17), 53.0 (C-7), 38.4 (C-6), 30.1 (C-5), 23.1 (C-14).

(*S*,*E*)-*N*-(9-(but-2-en-1-yloxy)-1,2,3-trimethoxy-10-oxo-5,6,7,10-tetrahydrobenzo[*a*]heptalen-7-yl)acetamide (compound **3e**): Yield: 52%; HPLC Rt = HPLC Rt = 5.265 (HPLC method 1) and 11.878 (HPLC method 2), Anal. Calcd $C_{25}H_{29}NO_6$: C, 68.32; H, 6.65; N, 3.19. Found: C, 68.34; H, 6.64; N, 3.18; HRMS (ESI-TOF) m/z: $[M + Na]^+$ Calcd for $C_{25}H_{29}NNaO_6^+$ 462.1888; Found 462.1889. ^{1}H NMR (500 MHz, $CDCl_3$, T = 298 K) δ 7.40 (d, $^{3}J_{H11,H12}$ = 12.8 Hz, 1H, H-12), 7.14 (s, 1H, H-8), 7.12 (d, $^{3}J_{H11,H12}$ = 12.5 Hz, 1H, H-11), 6.89 (d, $^{3}J_{H7,NH}$ = 6.4 Hz, 1H, 7-NH), 6.55 (s, 1H, H-4), 5.87 (m, 1H, H-19), 5.73 (m, 1H, H-20), 4.75 (m, 2H, H-18), 4.58 (dt, $^{3}J_{H6b, H7}$ = 12.4 Hz, $^{3}J_{H6a, H7}$ = 6.1 Hz, $^{3}J_{H7,NH}$ = 6.1 Hz, 1H, H-7), 3.92 (s, 3H, H-16), 3.89 (s, 3H, H-17), 3.66 (s, 3H, H-15), 2.49 (dd, ^{2}J = 13.2 Hz, $^{3}J_{H5b,H6a}$ = 6.2 Hz, 1H, H-5b), 2.35 (td, ^{2}J = 12.8 Hz, $^{3}J_{H5a, H6a}$ = 12.8 Hz, $^{3}J_{H5a, H6b}$ = 6.7 Hz, 1H, H-5a), 2.26 (tt, ^{2}J = 12.6, $^{3}J_{H5a,H6a}$ = 12.6 Hz, $^{3}J_{H5b,H6a}$ = 6.2 Hz, $^{3}J_{H6a, H7}$ = 6.2 Hz, 1H, H-6a), 2.03 (s, 3H, H-14), 2.02 – 1.94 (m, 1H, H-6b), 1.71 (dd, $^{3}J_{H20,H21}$ = 6.5 Hz, $^{4}J_{H219,H21}$ = 1.5 Hz, 3H, H-21), ^{13}C NMR (126 MHz, $CDCl_3$, T = 298 K) δ 179.6 (C-10), 169.9 (C-13), 163.1 (C-9), 153.7 (C-3), 151.1 (C-1), 144.1 (C-7a), 141.8 (C-2), 141.2 (C-12), 134.8 (C-12a), 134.7 (C-4a), 134.2 (C-11), 131.6 (C-19), 126.1 (C-1a), 124.9 (C-20), 111.8(C-8), 107.5 (C-4), 69.8 (C-18), 61.6 (C-15), 61.5 (C-16), 56.3 (C-17), 53.0 (C-7), 38.5 (C-6), 30.0 (C-5), 23.0 (C-14), 18.0 (C-21).

(*S*)-*N*-(1,2,3-trimethoxy-10-oxo-9-(prop-2-yn-1-yloxy)-5,6,7,10-tetrahydrobenzo[*a*]heptalen-7-yl)acetamide (compound **3f**): Yield: 52%; HPLC Rt = 6.591 (HPLC method 1) and 10.418 (HPLC method 2), Anal. Calcd $C_{24}H_{25}NO_6$: C, 68.07; H, 5.95; N, 3.31. Found: C, 68.09; H, 5.94; N, 3.30; HRMS (ESI-TOF) m/z: $[M + Na]^+$ Calcd for $C_{24}H_{25}NNaO_6^+$ 446.1574; Found 446.1575. ^{1}H NMR (600 MHz, $CDCl_3$, T = 298 K) δ 7.46 (d, $^{3}J_{H11,H12}$ = 12.8 Hz, 1H, H-12), 7.44 (s, 1H, H-8), 7.31 (m, 1H, 7-NH), 7.16 (d, $^{3}J_{H11,H12}$ = 12.8 Hz, 1H, H-11), 6.59 (s, 1H, H-4), 4.98 (m, 2H, H-18), 4.65 (m, 1H, H-7), 3.94 (s, 3H, H-16), 3.91 (s, 3H, H-17), 3.68 (s, 3H, H-15), 2.57 (m, 1H, H-20), 2.51 (m, 1H, H-5b), 2.34 (m, 1H, H-5a), 2.32 (m, 1H, H-6a), 2.06 (s, 3H, H-14), 2.06 – 2.01 (m, 1H, H-6b), ^{13}C NMR (151 MHz, $CDCl_3$, T = 298 K) δ 179.6 (C-10), 170.2 (C-13), 161.5 (C-9), 153.9 (C-3), 151.0 (C-1), 144.5 (C-7a), 141.7 (C-2), 141.6 (C-12), 136.1 (C-12a), 135.0 (C-4a), 134.6 (C-11), 125.8 (C-1a), 114.1 (C-8), 107.6 (C-4), 77.6 (C-19), 76.9 (C-20), 61.6 (C-15), 61.5 (C-16), 56.7 (C-18), 56.2 (C-17), 52.8 (C-7), 38.4 (C-6), 30.0 (C-5), 23.2 (C-14).

(*S*)-ethyl 2-((7-acetamido-1,2,3-trimethoxy-10-oxo-5,6,7,10-tetrahydrobenzo[*a*]heptalen-9-yl)oxy)acetate (compound **3g**): Yield: 44%; HPLC Rt = 4.838 (HPLC method 1) and 11.258 (HPLC method 2), Anal. Calcd $C_{25}H_{29}NO_8$: C, 63.68; H, 6.20; N, 2.97. Found: C, 63.69; H, 6.18; N, 2.98; HRMS (ESI-TOF) m/z: $[M + Na]^+$ Calcd for $C_{25}H_{29}NNaO_8^+$ 494.1785; Found

494.1784. ^{1}H NMR (400 MHz, $CDCl_3$, T = 298 K) δ 7.41 (d, $^{3}J_{H11,H12}$ = 12.8 Hz, 1H, H-12), 7.28 (s, 1H, H-8), 7.13 (d, $^{3}J_{H11,H12}$ = 12.8 Hz, 1H, H-11), 6.74 (d, $^{3}J_{H7,NH}$ = 6.5 Hz, 1H, 7-NH), 6.54 (s, 1H, H-4), 4.94 (d, ^{2}J=16.3 Hz, 1H, H-18a), 4.81 (d, ^{2}J=16.3 Hz, 1H, H-18b), 4.55 (dt, $^{3}J_{H6b, H7}$ = 12.2 Hz, $^{3}J_{H6a, H7}$ = 6.0 Hz, $^{3}J_{H7,NH}$ = 6.0 Hz, 1H, H-7), 4.20 (q, $^{3}J_{H20, H21}$ = 7.1 Hz, 1H, H-20a), 4.20 (q, $^{3}J_{H20, H21}$ = 7.1 Hz, 1H, H-20b),3.90 (s, 3H, H-16), 3.88 (s, 3H, H-17), 3.64 (s, 3H, H-15), 2.46 (m, 1H, H-5b), 2.28 (m, 1H, H-5a), 2.23 (m, 1H, H-6a), 2.03(s, 3H, H-14), 1.94 (m, 1H, H-6b), 1.23 (t, $^{3}J_{H20, H21}$ = 7.2 Hz, 3H, H-21), ^{13}C NMR (101 MHz, $CDCl_3$, T = 298 K) δ 179.7 (C-10), 170.1 (C-13), 169.1 (C-19), 162.2 (C-9), 153.9 (C-3), 151.1 (C-1), 143.9 (C-7a), 141.7 (C-2), 141.4 (C-12), 136.9 (C-12a), 135.6 (C-4a), 134.9 (C-11), 125.7 (C-1a), 116.8 (C-8), 107.6 (C-4), 66.8 (C-18), 61.6 (C-20), 61.6 (C-15), 61.5 (C-16), 56.2 (C-17), 52.6 (C-7), 38.5 (C-6), 30.0 (C-5), 23.0 (C-14), 14.2 (C-21).

(*S*)-*N*-(9-(but-3-en-1-yloxy)-1,2,3-trimethoxy-10-oxo-5,6,7,10-tetrahydrobenzo[*a*]heptalen-7-yl)acetamide (compound **3h**): Yield: 49%; HPLC Rt = 5.272 (HPLC method 1) and 11.891 (HPLC method 2), Anal. Calcd $C_{25}H_{29}NO_6$: C, 68.32; H, 6.65; N, 3.19. Found: C, 68.31; H, 6.67; N, 3.17; HRMS (ESI-TOF) m/z: $[M + Na]^+$ Calcd for $C_{25}H_{29}NNaO_6^+$ 462.1887; Found 462.1885. ^{1}H NMR (400 MHz, $CDCl_3$, T = 298 K) δ 7.39 (d, $^{3}J_{H11,H12}$ = 12.8 Hz, 1H, H-12), 7.13 (d, $^{3}J_{H11,H12}$ = 12.8 Hz, 1H, H-11), 7.05 (s, 1H, H-8), 6.56 (s, 1H, H-4), 6.38 (d, $^{3}J_{H7,NH}$ = 6.2 Hz, 1H, 7-NH), 5.90 (ddt, $^{3}J_{H20, H21trans}$ = 17.1 Hz, $^{3}J_{H20, H21cis}$ = 10.3 Hz, $^{3}J_{H19, H20}$ = 6.7 Hz, 1H, H-20), 5.17 (dq, ^{2}J = 1.6 Hz, $^{3}J_{H20, H21}$ = 7.2 Hz, $^{4}J_{H20, H21}$ = 1.6 Hz, 1H, H-21a), 5.11 (dq, ^{2}J = 1.2 Hz, $^{3}J_{H20, H21}$ = 10.2 Hz, $^{4}J_{H20, H21}$ = 1.2 Hz, 1H, H-21b), 4.58 (dt, $^{3}J_{H6b, H7}$ = 12.2 Hz, $^{3}J_{H6a, H7}$ = 6.1 Hz, $^{3}J_{H7,NH}$ = 6.1 Hz, 1H, H-7), 4.15 (m, 2H, H-18), 3.93 (s, 3H, H-16), 3.90 (s, 3H, H-17), 3.66 (s, 3H, H-15), 2.65 (m, 2H, H-19), 2.51 (dd, ^{2}J = 12.1 Hz, $^{3}J_{H5, H6}$ = 5.3 Hz, 1H, H-5b), 2.37 (td, ^{2}J = 12.6 Hz, $^{3}J_{H5, H6}$ = 12.6 Hz, $^{3}J_{H5, H6}$ = 6.9 Hz, 1H, H-5a), 2.30 (tt, ^{2}J = 12.6 Hz, $^{3}J_{H5, H6}$ = 12.6 Hz, $^{3}J_{H5, H6}$ = 6.5 Hz, $^{3}J_{H6, H7}$ = 6.5 Hz, 1H, H-6a), 2.04(s, 3H, H-14), 1.98 (m, 1H, H-6b), ^{13}C NMR (101 MHz, $CDCl_3$, T = 298 K) δ 179.6 (C-10), 169.8 (C-13), 163.4 (C-9), 153.7 (C-3), 151.2 (C-1), 143.5 (C-7a), 141.8 (C-2), 141.1 (C-12), 134.8 (C-12a), 134.7 (C-4a), 134.5 (C-11), 133.8 (C-20), 126.1 (C-1a), 117.8 (C-21), 111.6 (C-8), 107.5 (C-4), 66.5 (C-18), 61.6 (C-15), 61.6 (C-16), 56.3 (C-17), 53.0 (C-7), 38.5 (C-6), 33.3 (C-19), 30.0 (C-5), 23.1 (C-14).

(*S*)-*N*-(9-(3-azidopropoxy)-1,2,3-trimethoxy-10-oxo-5,6,7,10-tetrahydrobenzo[*a*]heptalen-7-yl)acetamide (compound **3i**): Yield: 51%; HPLC Rt = 5.332 (HPLC method 1) and 12.171 (HPLC method 2), Anal. Calcd $C_{24}H_{28}N_4O_6$: C, 61.53; H, 6.02; N, 11.96. Found: C, 61.55; H, 6.02; N, 11.95; HRMS (ESI-TOF) m/z: $[M + Na]^+$ Calcd for $C_{24}H_{28}N_4NaO_6^+$ 491.1901; Found 491.1900. ^{1}H NMR (400 MHz, $CDCl_3$, T = 298 K) δ 7.40 (d, $^{3}J_{H11,H12}$ = 12.8 Hz, 1H, H-12), 7.12 (d, $^{3}J_{H11,H12}$ = 12.9 Hz, 1H, H-11), 7.10 (s, 1H, H-8), 6.68 (d, $^{3}J_{H7,NH}$ = 6.3 Hz, 1H, 7-NH), 6.56 (s, 1H, H-4), 4.59 (dt, $^{3}J_{H6b, H7}$ = 12.3 Hz, $^{3}J_{H6a, H7}$ = 6.2 Hz, $^{3}J_{H7,NH}$ = 6.2 Hz, 1H, H-7), 4.22 (dt, ^{2}J = 9.9 Hz, $^{3}J_{H18, H19}$ = 6.3 Hz, 1H, H-18a), 4.14 (dt, ^{2}J = 9.8 Hz, $^{3}J_{H18, H19}$ = 5.9 Hz, 1H, H-18b), 3.92 (s, 3H, H-16), 3.89 (s, 3H, H-17), 3.65 (s, 3H, H-15), 3.57 (m, 2H, H-20), 2.51 (dd, ^{2}J = 12.7 Hz, $^{3}J_{H5, H6}$ = 5.7 Hz, 1H, H-5b), 2.37 (td, ^{2}J = 12.7 Hz, $^{3}J_{H5, H6}$ = 12.7 Hz, $^{3}J_{H5, H6}$ = 6.8 Hz, 1H, H-5a), 2.29 (tt, ^{2}J = 12.6 Hz, $^{3}J_{H5, H6}$ = 12.6 Hz, $^{3}J_{H5, H6}$ = 6.3 Hz, $^{3}J_{H6, H7}$ = 6.3 Hz, 1H, H-6a), 2.13 (m, 2H, H-19), 2.04(s, 3H, H-14), 2.0 (m, 1H, H-6b), ^{13}C NMR (101 MHz, $CDCl_3$, T = 298 K) δ 179.5 (C-10), 169.9 (C-13), 163.1 (C-9), 153.8 (C-

3), 151.1 (C-1), 143.9 (C-7a), 141.7 (C-2), 141.3 (C-12), 135.1 (C-12a), 134.8 (C-4a), 134.4 (C-11), 125.9 (C-1a), 111.8 (C-8), 107.5 (C-4), 66.8 (C-18), 61.6 (C-15), 61.5 (C-16), 56.2 (C-17), 53.0 (C-7), 48.2 (C-20), 38.5 (C-6), 30.0 (C-5), 28.5 (C-19), 23.0 (C-14).

(*S*)-*N*-(1,2,3-trimethoxy-10-oxo-9-((4-vinylbenzyl)oxy)-5,6,7,10-tetrahydrobenzo[*a*]heptalen-7-yl)acetamide (compound **3j**): Yield: 51%; HPLC Rt = 8.872 (HPLC method 1) and 15.745 (HPLC method 2), Anal. Calcd $C_{30}H_{31}NO_6$: C, 71.84; H, 6.23; N, 2.79. Found: C, 71.85; H, 6.21; N, 2.80; HRMS (ESI-TOF) m/z: $[M + Na]^+$ Calcd for $C_{30}H_{31}NNaO_6^+$ 524.2044; Found 524.2042. ^{1}H NMR (401 MHz, $CDCl_3$, T = 298 K) δ 7.41 (d, $^3J_{H11,H12}$ = 12.8 Hz, 1H, H-12), 7.34 (d, $^3J_{H20, H21}$ = 8.6 Hz, 1H, H-21), 7.34 (d, $^3J_{H23, H24}$ = 8.6 Hz, 1H, H-23), 7.31 (d, $^3J_{H20, H21}$ = 8.8 Hz, 1H, H-20), 7.31 (d, $^3J_{H23, H24}$ = 8.8 Hz, 1H, H-24),7.19 (s, 1H, H-8), 7.13 (d, $^3J_{H11,H12}$ = 12.7 Hz, 1H, H-11), 7.08 (d, $^3J_{H7,NH}$ = 6.5 Hz, 1H, 7-NH), 6.63 (dd, $^3J_{H25, H26}$ = 17.5 Hz, $^3J_{H25, H26}$ = 10.9 Hz, 1H, H-25), 6.52 (s, 1H, H-4), 4.50 (dt, $^3J_{H6b, H7}$ = 12.6 Hz, $^3J_{H6a, H7}$ = 6.4 Hz, $^3J_{H7,NH}$ = 6.4 Hz, 1H, H-7), 5.69 (dd, ^2J = 1.0 Hz, $^3J_{H25, H26}$ = 17.7 Hz, 1H, H-26a), 5.28 (d, ^2J = 13.0 Hz, 1H, H-18a), 5.23 (d, ^2J = 12.9 Hz, 1H, H-18b), 5.21 (dd, ^2J = 0.9 Hz, $^3J_{H25, H26}$ = 10.9 Hz, 1H, H-26b), 3.91 (s, 3H, H-16), 3.88 (s, 3H, H-17), 3.64 (s, 3H, H-15), 2.40 (dd, ^2J = 13.3 Hz, $^3J_{H5, H6}$ = 6.3 Hz, 1H, H-5b), 2.37 (td, ^2J = 13.0 Hz, $^3J_{H5, H6}$ = 13.0 Hz, $^3J_{H5, H6}$ = 7.2 Hz, 1H, H-5a), 2.12 (tt, ^2J = 12.6 Hz, $^3J_{H5, H6}$ = 12.6 Hz, $^3J_{H5, H6}$ = 6.0 Hz, $^3J_{H6, H7}$ = 6.0 Hz, 1H, H-6a), 1.87 (m, 1H, H-6b), 1.78(s, 3H, H-14), ^{13}C NMR (101 MHz, $CDCl_3$, T = 298 K) δ 179.6 (C-10), 169.9 (C-13), 163.0 (C-9), 153.7 (C-3), 151.1 (C-1), 144.4 (C-7a), 141.7 (C-2), 141.3 (C-12), 137.7 (C-22), 136.2 (C-25), 135.3 (C-12a), 135.2 (C-19), 134.9 (C-4a), 134.3 (C-11), 127.4 (C-21), 127.4 (C-23), 126.6 (C-20), 126.6 (C-26), 125.9 (C-1a), 114.6 (C-26), 112.9 (C-8), 107.6 (C-4), 70.8 (C-18), 61.6 (C-15), 61.5 (C-16), 56.2 (C-17), 52.8 (C-7), 38.3 (C-6), 30.0 (C-5), 22.8 (C-14).

(*S*)-*N*-(10-(benzyloxy)-1,2,3-trimethoxy-9-oxo-5,6,7,9-tetrahydrobenzo[*a*]heptalen-7-yl)acetamide (compound **4a**): Yield: 40%; HPLC Rt = HPLC Rt = 7.698 (HPLC method 1) and 14.598 (HPLC method 2), Anal. Calcd $C_{28}H_{29}NO_6$: C, 70.72; H, 6.15; N, 2.95. Found: C, 70.70; H, 6.14; N, 2.97; HRMS (ESI-TOF) m/z: $[M + Na]^+$ Calcd for $C_{28}H_{29}NNaO_6^+$ 498.1887; Found 498.1886. ^{1}H NMR (500 MHz, $CDCl_3$, T = 298 K) δ 8.0 (d, $^3J_{H7,NH}$ = 6.4 Hz, 1H, 7-NH), 7.60 (s, 1H, H-8), 7.42 (d, $^3J_{H23,H24}$ = 6.9 Hz, 1H, H-24), 7.42 (d, $^3J_{H20,H21}$ = 6.9 Hz, 1H, H-20), 7.36 (t, $^3J_{H20,H21}$ = 7.4 Hz, $^3J_{H21,H20jj}$ = 7.4 Hz, 1H, H-21), 7.36 (t, $^3J_{H22,H23}$ = 7.4 Hz, $^3J_{H23,H24}$ = 7.4 Hz, 1H, H-23), 7.30 (m, 1H, H-22), 7.21 (d, $^3J_{H11,H12}$ = 10.8 Hz, 1H, H-12), 6.89 (d, $^3J_{H11,H12}$ = 10.8 Hz, 1H, H-11), 6.50 (s, 1H, H-4), 5.35 (d, ^2J = 13.0 Hz, 1H, H-18a), 5.28 (d, ^2J = 12.9 Hz, 1H, H-18b), 4.62 (dt, $^3J_{H6b, H7}$ = 12.1 Hz, $^3J_{H6a, H7}$ = 6.4 Hz, $^3J_{H7,NH}$ = 6.4 Hz, 1H, H-7), 3.91 (s, 3H, H-16), 3.89 (s, 3H, H-17), 3.61 (s, 3H, H-15), 2.48 (dd, ^2J = 13.0 Hz, $^3J_{H5b,H6a}$ = 5.9 Hz, 1H, H-5b), 2.36 (td, ^2J = 12.8 Hz, $^3J_{H5a, H6a}$ = 12.8 Hz, $^3J_{H5a, H6b}$ = 6.2 Hz, 1H, H-5a), 2.28 (tt, ^2J = 12.8, $^3J_{H5a,H6a}$ = 12.8 Hz, $^3J_{H5b,H6a}$ = 6.3 Hz, $^3J_{H6a, H7}$ = 6.3 Hz, 1H, H-6a), 1.91 (m, 1H, H-6b), 1.97 (s, 3H, H-14), ^{13}C NMR (126 MHz, $CDCl_3$, T = 298 K) δ 179.8 (C-9), 170.2 (C-13), 163.1 (C-10), 153.6 (C-3), 152.2 (C-7a), 151.3 (C-1), 141.7 (C-2), 137.2 (C-12a), 135.4 (C-12), 135.4 (C-19), 134.4 (C-4a), 131.0 (C-8), 128.9 (C-21 + C-23), 128.4 (C-22), 127.2 (C-20 + C-24), 125.7 (C-1a), 115.1 (C-11), 107.4 (C-4), 71.1 (C-18), 61.7 (C-15), 61.5 (C-16), 56.2 (C-17), 52.6 (C-7), 36.5 (C-6), 30.0 (C-5), 22.9 (C-14).

(*S*)-*N*-(10-((4-iodobenzyl)oxy)-1,2,3-trimethoxy-9-oxo-5,6,7,9-tetrahydrobenzo[*a*]heptalen-7-yl)acetamide (compound **4b**): Yield: 42%; HPLC Rt = 14.351 (HPLC method 1) and 20.845 (HPLC method 2), Anal. Calcd $C_{28}H_{28}INO_6$: C, 55.92; H, 4.69; I, 21.10; N, 2.33. Found C, 55.91; H, 4.70; I, 20.12; N, 2.34; HRMS (ESI-TOF) m/z: $[M + Na]^+$ Calcd for $C_{28}H_{28}INNaO_6^+$ 624.0854; Found 624.0853. ^{1}H NMR (401 MHz, $CDCl_3$, T = 298 K) δ 7.70 (d, $^3J_{H20,H21}$ = 8.3 Hz, 1H, H-21), 7.70 (d, $^3J_{H22,H23}$ = 8.3 Hz, 1H, H-23),7.63 (d, $^3J_{H7,NH}$ = 6.4 Hz, 1H, 7-NH), 7.55 (s, 1H, H-8), 7.21 (d, $^3J_{H11,H12}$ = 11.2 Hz, 1H, H-12),7.18 (d, $^3J_{H23,H24}$ = 8.4 Hz, 1H, H-24), 7.18 (d, $^3J_{H20,H21}$ = 8.4 Hz, 1H, H-20), 6.84 (d, $^3J_{H11,H12}$ = 10.8 Hz, 1H, H-11), 6.51 (s, 1H, H-4), 5.28 (d, ^2J = 13.1 Hz, 1H, H-18a), 5.20 (d, ^2J = 13.0 Hz, 1H, H-18b), 4.62 (dt, $^3J_{H6b, H7}$ = 11.9 Hz, $^3J_{H6a, H7}$ = 6.3 Hz, $^3J_{H7,NH}$ = 6.3 Hz, 1H, H-7), 3.92 (s, 3H, H-16), 3.89 (s, 3H, H-17), 3.61 (s, 3H, H-15), 2.50 (dd, ^2J = 12.9 Hz, $^3J_{H5b,H6a}$ = 5.8 Hz, 1H, H-5b), 2.36 (td, ^2J = 12.8 Hz, $^3J_{H5a, H6a}$ = 12.8 Hz, $^3J_{H5a, H6b}$ = 6.2 Hz, 1H, H-5a), 2.27 (tt, ^2J = 12.4, $^3J_{H5a,H6a}$ = 12.4 Hz, $^3J_{H5b,H6a}$ = 6.1 Hz, $^3J_{H6a, H7}$ = 6.1 Hz, 1H, H-6a), 1.97 (s, 3H, H-14), 1.89 (m, 1H, H-6b), ^{13}C NMR (101 MHz, $CDCl_3$, T = 298 K) δ 179.7 (C-9), 170.0 (C-13), 162.8 (C-10), 153.7 (C-3), 152.0 (C-7a), 151.3 (C-1), 141.8 (C-2), 138.0 (C-21 + C-23), 137.4 (C-12a), 135.2 (C-12), 135.2 (C-19), 134.3 (C-4a), 131.2 (C-8), 129.1 (C-20 + C-24), 125.6 (C-1a), 115.1 (C-11), 107.5 (C-4), 93.9 (C-22), 70.4 (C-18), 61.7 (C-15), 61.5 (C-16), 56.2 (C-17), 52.6 (C-7), 36.6 (C-6), 30.0 (C-5), 23.0 (C-14).

(*S*,*E*)-*N*-(10-(cinnamyloxy)-1,2,3-trimethoxy-9-oxo-5,6,7,9-tetrahydrobenzo[*a*]heptalen-7-yl)acetamide (compound **4c**): Yield: 39%; HPLC Rt = 10.505 (HPLC method 1) and 17.945 (HPLC method 2), Anal. Calcd $C_{30}H_{31}NO_6$: C, 71.84; H, 6.23; N, 2.79. Found C, C, 71.85; H, 6.21; N, 2.80; HRMS (ESI-TOF) m/z: $[M + Na]^+$ Calcd for $C_{30}H_{31}NNaO_6^+$ 524.2044; Found 524.2046. ^{1}H NMR (500 MHz, $CDCl_3$, T = 298 K) δ 7.54 (s, 1H, H-8), 7.41 (m, 1H, H-26), 7.41 (m, 1H, H-22), 7.38 (d, $^3J_{H7,NH}$ = 6.9 Hz, 1H, 7-NH), 7.33 (m, 1H, H-25), 7.33 (m, 1H, H-23), 7.29 (d, $^3J_{H11,H12}$ = 10.7 Hz, 1H, H-12),7.27 (m, 1H, H-24), 6.95 (d, $^3J_{H11,H12}$ = 10.8 Hz, 1H, H-11), 6.76 (m, 1H, H-20), 6.51 (s, 1H, H-4), 6.44 (dt, $^3J_{H19,H20}$ = 16.0 Hz, $^3J_{H18,H19}$ = 5.9 Hz, 1H, H-19), 4.95 (ddd, ^2J = 13.1 Hz, $^3J_{H18,H19}$ = 5.8 Hz, , $^4J_{H18,H20}$ = 1.7 Hz, 1H, H-18a), 4.91 (ddd, ^2J = 13.1 Hz, $^3J_{H18,H19}$ = 6.1 Hz, , $^4J_{H18,H20}$ = 1.4 Hz, 1H, H-18b), 4.65 (dt, $^3J_{H6b, H7}$ = 12.2 Hz, $^3J_{H6a, H7}$ = 6.4 Hz, $^3J_{H7,NH}$ = 6.4 Hz, 1H, H-7), 3.94 (s, 3H, H-16), 3.90 (s, 3H, H-17), 3.65 (s, 3H, H-15), 2.51 (dd, ^2J = 13.3 Hz, $^3J_{H5b,H6a}$ = 6.2 Hz, 1H, H-5b), 2.40 (td, ^2J = 13.1 Hz, $^3J_{H5a, H6a}$ = 13.1 Hz, $^3J_{H5a, H6b}$ = 6.6 Hz, 1H, H-5a), 2.27 (tt, ^2J = 12.4, $^3J_{H5a,H6a}$ = 12.4 Hz, $^3J_{H5b,H6a}$ = 6.1 Hz, $^3J_{H6a, H7}$ = 6.1 Hz, 1H, H-6a), 1.99 (s, 3H, H-14), 1.89 (td, ^2J = 11.7, $^3J_{H5a,H6a}$ = 11.7 Hz, $^3J_{H5b,H6a}$ = 6.3 Hz, 1H, H-6b), ^{13}C NMR (126 MHz, $CDCl_3$, T = 298 K) δ 179.8 (C-9), 170.0 (C-13), 163.2 (C-10), 153.6 (C-3), 151.7 (C-7a), 151.3 (C-1), 141.8 (C-2), 136.9 (C-12a), 136.1 (C-21), 135.4 (C-12), 134.5 (C-20), 134.4 (C-4a), 131.0 (C-8), 128.8 (C-22 + 26), 128.4 (C-24), 126.8 (C-23 + C-25), 125.8 (C-1a), 122.8 (C-19), 114.4 (C-11), 107.5 (C-4), 70.2 (C-18), 61.7 (C-15), 61.5 (C-16), 56.3 (C-17), 52.6 (C-7), 36.8 (C-6), 30.0 (C-5), 23.1 (C-14).

(*S*)-*N*-(10-(allyloxy)-1,2,3-trimethoxy-9-oxo-5,6,7,9-tetrahydrobenzo[*a*]heptalen-7-yl)acetamide (compound **4d**): Yield: 41%; HPLC Rt = HPLC Rt = 4.858 (HPLC method 1) and 11.645 (HPLC method 2), Anal. Calcd $C_{24}H_{27}NO_6$: C, 67.75; H, 6.40; N, 3.29. Found: C, 67.77; H, 6.39; N, 3.30; HRMS (ESI-TOF) m/z: $[M + Na]^+$ Calcd for $C_{24}H_{27}NNaO_6^+$

448.1731; Found 448.1732. ^{1}H NMR (500 MHz, $CDCl_3$, T = 298 K) δ 7.55 (s, 1H, H-8), 7.78 (d, $^3J_{H7,NH}$ = 6.5 Hz, 1H, 7-NH), 7.28 (d, $^3J_{H11,H12}$ = 10.7 Hz, 1H, H-12), 6.88 (d, $^3J_{H11,H12}$ = 10.8 Hz, 1H, H-11), 6.52 (s, 1H, H-4), 6.04 (ddt, $^3J_{H19,H20}$ = 17.2 Hz, $^3J_{H19,H20}$ = 10.6 Hz, $^3J_{H18,H19}$ = 5.3 Hz, 1H, H-19), 5.44 (dq, ^2J = 1.5 Hz, $^3J_{H19,H20}$ = 10.0 Hz, $^4J_{H18,H20}$ = 1.5 Hz, 1H, H-20a), 5.35 (dq, ^2J = 1.3 Hz, $^3J_{H19,H20}$ = 17.3 Hz, $^4J_{H18,H20}$ = 1.3 Hz, 1H, H-20b), 4.79 (m, 1H, H-18a), 4.75 (m, 1H, H-18b), 4.63 (dt, $^3J_{H6b, H7}$ = 12.2 Hz, $^3J_{H6a, H7}$ = 6.4 Hz, $^3J_{H7,NH}$ = 6.4 Hz, 1H, H-7), 3.93 (s, 3H, H-16), 3.89 (s, 3H, H-17), 3.64 (s, 3H, H-15), 2.51 (dd, ^2J = 12.9 Hz, $^3J_{H5b,H6a}$ = 5.7 Hz, 1H, H-5b), 2.38 (td, ^2J = 13.0 Hz, $^3J_{H5a, H6a}$ = 13.0 Hz, $^3J_{H5a, H6b}$ = 6.4 Hz, 1H, H-5a), 2.30 (tt, ^2J = 12.7, $^3J_{H5a,H6a}$ = 12.7 Hz, $^3J_{H5b,H6a}$ = 6.3 Hz, $^3J_{H6a, H7}$ = 6.3 Hz, 1H, H-6a), 1.96 (s, 3H, H-14), 1.91 (m, 1H, H-6b), ^{13}C NMR (126 MHz, $CDCl_3$, T = 298 K) δ 179.7 (C-9), 170.1 (C-13), 163.1 (C-10), 153.6 (C-3), 152.1 (C-7a), 151.3 (C-1), 141.8 (C-2), 137.1 (C-12a), 135.4 (C-12), 134.4 (C-4a), 131.8 (C-19), 130.9 (C-8), 125.8 (C-1a), 119.2 (C-20), 114.5 (C-11), 107.4 (C-4), 70.2 (C-18), 61.7 (C-15), 61.5 (C-16), 56.2 (C-17), 52.7 (C-7), 36.6 (C-6), 30.0 (C-5), 23.0 (C-14).

(*S*,*E*)-*N*-(10-(but-2-en-1-yloxy)-1,2,3-trimethoxy-9-oxo-5,6,7,9-tetrahydrobenzo[*a*]heptalen-7-yl)acetamide (compound **4e**): Yield: 39%; HPLC Rt = 5.845 (HPLC method 1) and 13.025 (HPLC method 2), Anal. Calcd $C_{25}H_{29}NO_6$: C, 68.32; H, 6.65; N, 3.19. Found: C, 68.31; H, 6.67; N, 3.20; HRMS (ESI-TOF) m/z: $[M + Na]^+$ Calcd for $C_{25}H_{29}NNaO_6^+$ 462.1888; Found 462.1886. ^{1}H NMR (500 MHz, $CDCl_3$, T = 298 K) δ 7.76 (d, $^3J_{H7,NH}$ = 6.6 Hz, 1H, 7-NH), 7.54 (s, 1H, H-8), 7.28 (d, $^3J_{H11,H12}$ = 10.8 Hz, 1H, H-12), 6.88 (d, $^3J_{H11,H12}$ = 11.0 Hz, 1H, H-11), 6.52 (s, 1H, H-4), 5.87 (m, 1H, H-19), 5.75 (m, 1H, H-20), 4.63 (dt, $^3J_{H6b, H7}$ = 12.1 Hz, $^3J_{H6a, H7}$ = 6.4 Hz, $^3J_{H7,NH}$ = 6.4 Hz, 1H, H-7), 4.22 (d, $^3J_{H18, H19}$ = 6.1 Hz, 2H, H-18), 3.93 (s, 3H, H-16), 3.89 (s, 3H, H-17), 3.64 (s, 3H, H-15), 2.50 (dd, ^2J = 13.3 Hz, $^3J_{H5b,H6a}$ = 6.0 Hz, 1H, H-5b), 2.38 (td, ^2J = 13.0 Hz, $^3J_{H5a, H6a}$ = 13.0 Hz, $^3J_{H5a, H6b}$ = 6.4 Hz, 1H, H-5a), 2.30 (tt, ^2J = 12.8, $^3J_{H5a,H6a}$ = 12.8 Hz, $^3J_{H5b,H6a}$ = 6.3 Hz, $^3J_{H6a, H7}$ = 6.3 Hz, 1H, H-6a), 1.96 (s, 3H, H-14), 1.89 (m, 1H, H-6b), 1.75 (m, 3H, H-21), ^{13}C NMR (126 MHz, $CDCl_3$, T = 298 K) δ 179.8 (C-9), 170.1 (C-13), 163.4 (C-10), 153.6 (C-3), 151.9 (C-7a), 151.3 (C-1), 141.8 (C-2), 136.7 (C-12a), 135.5 (C-12), 134.4 (C-4a), 132.2 (C-19), 130.7 (C-8), 125.8 (C-1a), 124.6 (C-20), 114.2 (C-11), 107.4 (C-4), 70.2 (C-18), 61.7 (C-15), 61.5 (C-16), 56.2 (C-17), 52.7 (C-7), 36.7 (C-6), 30.0 (C-5), 23.0 (C-14), 18.0 (C-21).

(*S*)-*N*-(1,2,3-trimethoxy-9-oxo-10-(prop-2-yn-1-yloxy)-5,6,7,9-tetrahydrobenzo[*a*]heptalen-7-yl)acetamide (compound **4f**): Yield: 41%; HPLC Rt = 5.591 (HPLC method 1) and 11.065 (HPLC method 2), Anal. Calcd $C_{24}H_{25}NO_6$: C, 68.07; H, 5.95; N, 3.31. Found: C, 68.06; H, 5.97; N, 3.32; HRMS (ESI-TOF) m/z: $[M + Na]^+$ Calcd for $C_{24}H_{25}NNaO_6^+$ 446.1574; Found 446.1572. ^{1}H NMR (600 MHz, $CDCl_3$, T = 298 K) δ 7.90 (d, $^3J_{H7,NH}$ = 6.4 Hz, 1H, 7-NH), 7.58 (s, 1H, H-8), 7.34 (d, $^3J_{H11,H12}$ = 10.6 Hz, 1H, H-12), 7.11 (d, $^3J_{H11,H12}$ = 10.6 Hz, 1H, H-11), 6.53 (s, 1H, H-4), 4.97 (dd, ^2J = 16.2 Hz, $^4J_{H18, H20}$ = 2.4 Hz, 1H, H-18a), 4.91 (dd, ^2J = 16.2 Hz, $^4J_{H18, H20}$ = 2.4 Hz, 1H, H-18b), 4.64 (dt, $^3J_{H6b, H7}$ = 11.9 Hz, $^3J_{H6a, H7}$ = 6.4 Hz, $^3J_{H7,NH}$ = 6.4 Hz, 1H, H-7),3.94 (s, 3H, H-16), 3.91 (s, 3H, H-17), 3.66 (s, 3H, H-15), 2.58 (t,$^4J_{H18, H20}$ = 2.4 Hz, 1H, H-20), 2.53 (dd, ^2J = 13.6 Hz, $^3J_{H5b,H6a}$ = 5.5 Hz, 1H, H-5b), 2.39 (td, ^2J = 13.1 Hz, $^3J_{H5a, H6a}$ = 13.1 Hz, $^3J_{H5a, H6b}$ = 6.5 Hz, 1H, H-5a), 2.32 (tt, ^2J = 12.9, $^3J_{H5a,H6a}$ = 12.9 Hz, $^3J_{H5b,H6a}$ = 6.3 Hz, $^3J_{H6a, H7}$ = 6.3 Hz, 1H, H-6a), 1.97 (s, 3H, H-14), 1.93 (m, 1H, H-

6b), ^{13}C NMR (151 MHz, $CDCl_3$, T = 299 K) δ 179.7 (C-9), 170.2 (C-13), 161.7 (C-10), 153.8 (C-3), 152.5 (C-7a), 151.3 (C-1), 141.8 (C-2), 138.1 (C-12a), 135.3 (C-12), 134.3 (C-4a), 77.4 (C-19), 131.4 (C-8), 125.6 (C-1a), 76.9 (C-20), 115.6 (C-11), 107.5 (C-4), 56.8 (C-18), 61.7 (C-15), 61.5 (C-16), 56.3 (C-17), 52.7 (C-7), 36.5 (C-6), 30.0 (C-5), 23.0 (C-14).

(*S*)-ethyl 2-((7-acetamido-1,2,3-trimethoxy-9-oxo-5,6,7,9-tetrahydrobenzo[*a*]heptalen-10-yl)oxy)acetate (compound **4g**): Yield: 37%; HPLC Rt = 4.838 (HPLC method 1) and 11.785 (HPLC method 2), Anal. Calcd $C_{25}H_{29}NO_8$: C, 63.68; H, 6.20; N, 2.97. Found: C, 63.66; H, 6.22; N, 2.96; HRMS (ESI-TOF) m/z: $[M + Na]^+$ Calcd for $C_{25}H_{29}NNaO_8^+$ 494.1785; Found 494.1787. ^{1}H NMR (400 MHz, $CDCl_3$, T = 298 K) δ 7.67 (d, $^3J_{H7,NH}$ = 6.6 Hz, 1H, 7-NH), 7.55 (s, 1H, H-8), 7.26 (d, $^3J_{H11,H12}$ = 10.8 Hz, 1H, H-12), 6.82 (d, $^3J_{H11,H12}$ = 10.8 Hz, 1H, H-11), 6.52 (s, 1H, H-4), 4.90 (dd, ^2J = 16.2 Hz, 1H, H-18a), 4.83 (dd, ^2J = 16.4 Hz, 1H, H-18b), 4.63 (dt, $^3J_{H6b, H7}$ = 12.1 Hz, $^3J_{H6a, H7}$ = 6.4 Hz, $^3J_{H7,NH}$ = 6.4 Hz, 1H, H-7),4.27 (q, $^3J_{H20, H21}$ = 7.2 Hz, 1H, H-20a), 4.26 (q, $^3J_{H20, H21}$ = 7.1 Hz, 1H, H-20b), 3.92 (s, 3H, H-16), 3.89 (s, 3H, H-17), 3.63 (s, 3H, H-15), 2.52 (dd, ^2J = 13.0 Hz, $^3J_{H5b,H6a}$ = 5.8 Hz, 1H, H-5b), 2.39 (td, ^2J = 12.9 Hz, $^3J_{H5a, H6a}$ = 12.9 Hz, $^3J_{H5a, H6b}$ = 6.3 Hz, 1H, H-5a), 2.28 (tt, ^2J = 12.8, $^3J_{H5a,H6a}$ = 12.8 Hz, $^3J_{H5b,H6a}$ = 6.1 Hz, $^3J_{H6a, H7}$ = 6.1 Hz, 1H, H-6a), 1.97 (s, 3H, H-14), 1.89 (m, 1H, H-6b), 1.28 (t, $^3J_{H20, H21}$ = 7.1 Hz, 3H, H-21), ^{13}C NMR (101 MHz, $CDCl_3$, T = 298 K) δ 179.5 (C-9), 170.1 (C-13), 167.8 (C-19), 162.2 (C-10), 153.7 (C-3), 152.5 (C-7a), 151.3 (C-1), 141.7 (C-2), 138.5 (C-12a), 135.1 (C-12), 134.3 (C-4a), 131.9 (C-8), 125.5 (C-1a), 116.3 (C-11), 107.4 (C-4), 66.1 (C-18), 61.8 (C-20), 61.7 (C-15), 61.5 (C-16), 52.6 (C-7), 56.2 (C-17), 36.6 (C-6), 30.0 (C-5), 23.0 (C-14), 14 .3 (C-21).

(*S*)-*N*-(10-(but-3-en-1-yloxy)-1,2,3-trimethoxy-9-oxo-5,6,7,9-tetrahydrobenzo[*a*]heptalen-7-yl)acetamide (compound **4h**): Yield: 40%; HPLC Rt = 5.658 (HPLC method 1) and 12.731 (HPLC method 2), Anal. Calcd $C_{25}H_{29}NO_6$: C, 68.32; H, 6.65; N, 3.19. Found: C, 68.30; H, 6.66; N, 3.18; HRMS (ESI-TOF) m/z: $[M + Na]^+$ Calcd for $C_{25}H_{29}NNaO_6^+$ 462.1887; Found 462.1883. ^{1}H NMR (401 MHz, $CDCl_3$, T = 298 K) δ 7.76 (d, $^3J_{H7,NH}$ = 6.6 Hz, 1H, 7-NH), 7.54 (s, 1H, H-8), 7.28 (d, $^3J_{H11,H12}$ = 10.8 Hz, 1H, H-12), 6.85 (d, $^3J_{H11,H12}$ = 10.8 Hz, 1H, H-11), 6.52 (s, 1H, H-4), 5.89 (ddt, $^3J_{H20, H21}$ = 17.1 Hz, $^3J_{H20, H21}$ = 10.3 Hz, $^3J_{H19, H20}$ = 6.7 Hz, 1H, H-20), 5.19 (dq, ^2J = 1.5 Hz, $^3J_{H20, H21}$ = 17.2 Hz, $^4J_{H20, H21}$ = 1.5 Hz, 1H, H-21a), 5.13 (dq, ^2J = 1.2 Hz, $^3J_{H20, H21}$ = 10.2 Hz, $^4J_{H20, H21}$ = 1.2 Hz, 1H, H-21b), 4.64 (dt, $^3J_{H6b, H7}$ = 12.2 Hz, $^3J_{H6a, H7}$ = 6.4 Hz, $^3J_{H7,NH}$ = 6.4 Hz, 1H, H-7), 4.17 (t, $^3J_{H18, H19}$ = 7.1 Hz, 2H, H-18), 3.93 (s, 3H, H-16), 3.89 (s, 3H, H-17), 3.64 (s, 3H, H-15), 2.68 (m, 2H, H-19),2.51 (dd, ^2J = 13.1 Hz, $^3J_{H5b,H6a}$ = 6.0 Hz, 1H, H-5b), 2.38 (td, ^2J = 13.1 Hz, $^3J_{H5a, H6a}$ = 13.1 Hz, $^3J_{H5a, H6b}$ = 6.0 Hz, 1H, H-5a), 2.29 (tt, ^2J = 12.8, $^3J_{H5a,H6a}$ = 12.8 Hz, $^3J_{H5b,H6a}$ = 6.3 Hz, $^3J_{H6a, H7}$ = 6.3 Hz, 1H, H-6a), 1.96 (s, 3H, H-14), 1.90 (m, 1H, H-6b), ^{13}C NMR (101 MHz, $CDCl_3$, T = 298 K) δ 179.7 (C-9), 170.1 (C-13), 163.5 (C-10), 153.6 (C-3), 151.8 (C-7a), 151.3 (C-1), 141.8 (C-2), 136.8 (C-12a), 135.4 (C-12), 134.4 (C-4a), 133.5 (C-20),130.9 (C-8), 125.8 (C-1a), 117.9 (C-21), 113.9 (C-11), 107.5 (C-4), 68.7 (C-18), 61.7 (C-15), 61.5 (C-16), 56.2 (C-17), 52.5 (C-7), 36.7 (C-6), 33.1 (C-19), 30.1 (C-5), 23.0 (C-14).

(*S*)-*N*-(10-(3-azidopropoxy)-1,2,3-trimethoxy-9-oxo-5,6,7,9-tetrahydrobenzo[*a*]heptalen-7-yl)acetamide (compound **4i**): Yield: 41%; HPLC Rt = 5.512 (HPLC method 1) and 12.705

(HPLC method 2), Anal. Calcd $C_{24}H_{28}N_4O_6$: C, 61.53; H, 6.02; N, 11.96. Found: C, 61.51; H, 6.03; N, 11.98; HRMS (ESI-TOF) m/z: $[M + Na]^+$ Calcd for $C_{24}H_{28}N_4NaO_6^+$ 491.1901; Found 491.1899. ^{1}H NMR (401 MHz, $CDCl_3$, T = 298 K) δ 7.44 (s, 1H, H-8), 7.28 (d, $^3J_{H11,H12}$ = 10.7 Hz, 1H, H-12), 6.94 (d, $^3J_{H7,NH}$ = 6.9 Hz, 1H, 7-NH),6.84 (d, $^3J_{H11,H12}$ = 10.8 Hz, 1H, H-11), 6.53 (s, 1H, H-4), 4.63 (dt, $^3J_{H6b, H7}$ = 12.4 Hz, $^3J_{H6a, H7}$ = 6.5 Hz, $^3J_{H7,NH}$ = 6.5 Hz, 1H, H-7), 4.19 (m, 2H, H-18), 3.94 (s, 3H, H-16), 3.90 (s, 3H, H-17), 3.64 (s, 3H, H-15), 3.60 (m, 2H, H-20), 2.52 (dd, ^2J = 13.4 Hz, $^3J_{H5b,H6a}$ = 6.3 Hz, 1H, H-5b), 2.40 (td, ^2J = 13.1 Hz, $^3J_{H5a, H6a}$ = 13.1 Hz, $^3J_{H5a, H6b}$ = 6.6 Hz, 1H, H-5a), 2.27 (m, 1H, H-6a), 2.19 (m, 2H, H-19), 1.99 (s, 3H, H-14), 1.84 (m, 1H, H-6b), 1.75 (m, 3H, H-21), ^{13}C NMR (101 MHz, $CDCl_3$, T = 298 K) δ 179.6 (C-9), 169.8 (C-13), 163.4 (C-10), 153.6 (C-3), 151.4 (C-7a), 151.3 (C-1), 141.8 (C-2), 136.8 (C-12a), 135.2 (C-12), 134.3 (C-4a), 131.1 (C-8), 125.8 (C-1a), 113.8 (C-11), 107.5 (C-4), 66.0 (C-18), 61.7 (C-15), 61.5 (C-16), 56.3 (C-17), 52.4 (C-7), 48.2 (C-20), 36.9 (C-6), 30.0 (C-5), 28.5 (C-19), 23.2 (C-14).

(*S*)-*N*-(1,2,3-trimethoxy-9-oxo-10-((4-vinylbenzyl)oxy)-5,6,7,9-tetrahydrobenzo[*a*]heptalen-7-yl)acetamide (compound **4j**): Yield: 40%; HPLC Rt = 11.105 (HPLC method 1) and 18.491 (HPLC method 2), Anal. Calcd $C_{30}H_{31}NO_6$: C, 71.84; H, 6.23; N, 2.79. Found: C, 71.82; H, 6.24; N, 2.80; HRMS (ESI-TOF) m/z: $[M + Na]^+$ Calcd for $C_{30}H_{31}NNaO_6^+$ 524.2044; Found 524.2043. ^{1}H NMR (500 MHz, $CDCl_3$, T = 298 K) δ 7.60 (d, $^3J_{H7,NH}$ = 6.5 Hz, 1H, 7-NH), 7.54 (s, 1H, H-8), 7.41 (d, $^3J_{H20, H21}$ = 8.6 Hz, 1H, H-21), 7.41 (d, $^3J_{H23, H24}$ = 8.6 Hz, 1H, H-23), 7.38 (d, $^3J_{H20, H21}$ = 8.8 Hz, 1H, H-20), 7.38 (d, $^3J_{H23, H24}$ = 8.8 Hz, 1H, H-24), 7.21 (d, $^3J_{H11,H12}$ = 10.7 Hz, 1H, H-12), 6.88 (d, $^3J_{H11,H12}$ = 10.9 Hz, 1H, H-11), 6.69 (dd, $^3J_{H25, H26}$ = 17.6 Hz, $^3J_{H25, H26}$ = 10.5 Hz, 1H, H-25), 6.50 (s, 1H, H-4), 5.74 (dd, ^2J = 0.9 Hz, $^3J_{H25, H26}$ = 17.6 Hz, 1H, H-26a), 5.36 (d, ^2J = 13.1 Hz, 1H, H-18a), 5.28 (d, ^2J = 12.9 Hz, 1H, H-18b), 5.21 (dd, ^2J = 0.9 Hz, $^3J_{H25, H26}$ = 10.9 Hz, 1H, H-26b), 4.63 (dt, $^3J_{H6b, H7}$ = 11.7 Hz, $^3J_{H6a, H7}$ = 6.1 Hz, $^3J_{H7,NH}$ = 6.1 Hz, 1H, H-7), 3.91 (s, 3H, H-16), 3.88 (s, 3H, H-17), 3.61 (s, 3H, H-15), 2.52 (dd, ^2J = 12.7 Hz, $^3J_{H5b,H6a}$ = 5.2 Hz, 1H, H-5b), 2.36 (td, ^2J = 12.9 Hz, $^3J_{H5a, H6a}$ = 12.9 Hz, $^3J_{H5a, H6b}$ = 6.2 Hz, 1H, H-5a), 2.30 (m, 1H, H-6a), 1.98 (s, 3H, H-14), 1.89 (m, 1H, H-6b), ^{13}C NMR (101 MHz, $CDCl_3$, T = 298 K) δ 179.8 (C-9), 170.1 (C-13), 163.1 (C-10), 153.6 (C-3), 152.1 (C-7a), 151.3 (C-1), 141.7 (C-2), 137.8 (C-22), 137.2 (C-12a), 136.4 (C-25), 135.3 (C-19), 134.9 (C-12), 134.3 (C-4a), 131.1 (C-8), 127.4 (C-21), 127.4 (C-23), 126.7 (C-20), 126.7 (C-24), 125.7 (C-1a), 115.1 (C-11), 114.5 (C-26), 107.4 (C-4), 70.9 (C-18), 61.7 (C-15), 61.5 (C-16), 52.6 (C-7), 56.2 (C-17), 36.6 (C-6), 30.0 (C-5), 23.0 (C-14).

(*S*)-*N*-(9-((1-benzyl-1*H*-1,2,3-triazol-4-yl)methoxy)-1,2,3-trimethoxy-10-oxo-5,6,7,10-tetrahydrobenzo[*a*]heptalen-7-yl)acetamide (compound **5a**) Derivative **3f** 70 mg (0.17 mmol) was dissolved in a 6 mL mixture of THF/MeOH (3:1) and benzyl azide 22 mg (0.17 mmol) was added. Then, to each mixture, 6.5 mg (0.05 mmol) of $CH_3COOCu(I)$ and 45 mg (0.25 mmol) of ascorbic acid was added. The solution was stirred at room temperature for a three hours and after this time, dichloromethane was added and extracted twice with 25 mL of ammonium hydroxide, twice with 25mL of water and finally twice with 25 mL of brine. The organic layer was evaporated and the synthesized derivative **5a** was purified by column chromatography with silica gel with dichloromethane/acetone (2:1) as an eluent. The product was obtained as a yellow powder. (24 mg, 27%) mp 121-125 °C. HPLC R_t= 9.680 min. Anal.

Calcd for $C_{31}H_{32}N_4O_6$: C, 66.89; H, 5.79; N, 10.07. Found: C, 66.91; H, 5.78; N, 10.05; HRMS (ESI-TOF) m/z: $[M + H]^+$ Calcd 556.2322; Found 556.2320. FT-IR (KBr): ν$(N\text{-}H)_{amide}$ - 3287 cm^{-1}; ν(=CH) - 3063 cm^{-1}; ν(C-H) - 2934 cm^{-1} ; 2854 cm^{-1} ; ν$(C{=}O)_{amide}$ - 1662 cm^{-1}; ν$(C{=}C)_{ar}$ - 1611 cm^{-1}; δ $(N\text{-}H)_{amide}$ - 1559 cm^{-1}; ν(C=C) - 1489 cm^{-1}; ν$(C\text{-}O)_{as}$.- 1253 cm^{-1}; ν$(C\text{-}O)_{sym}$ -1166, 1096 cm^{-1}. ^1H NMR (500 MHz, $CDCl_3$, 25ºC) δ 7.72 (s, 1H, H-20), 7.72 (s, 1H, H-8), 7.40 (s, 1H, H-12), 7.40 – 7.38 (m, 2H, H-24,26), 7.38 – 7.37 (m, 2H, H-23,27), 7.27 (s, 1H, H-25), 7.12 (d, $^3J_{H11,H12}$ = 12.8 Hz, 1H, H-11), 6.92 (d, $^3J_{H7,NH7}$ = 8.0 Hz, 1H, NH-7), 6.54 (s, 1H, H-4), 5.51 (d, ^2J = 1.8 Hz, 2H, H-21), 5.47 (d, ^2J = 13.9 Hz, 1H, H-18), 5.40 (d, ^2J = 13.8 Hz, 1H, H-18), 4.69 (dt, $^3J_{H6b,H7}$ = 13.5 Hz, $^3J_{H6a,H7}$ = 6.9 Hz, $^3J_{H7,NH7}$ = 6.9 Hz, 1H, H-7), 3.92 (s, 3H, H-16), 3.89 (s, 3H, H-17), 3.66 (s, 3H, H-15), 2.43 (dd, ^2J = 13.0 Hz, $^3J_{H5b,H6a}$ = 6.1 Hz, 1H, H-5b), 2.31 (td, ^2J = 12.3 Hz, $^3J_{H5a,H6a}$ = 12.3 Hz, $^3J_{H5a,H6b}$ = 6.2 Hz, 1H, H-5a), 2.22 (tt, ^2J = 12.9 Hz, $^3J_{H5a,H6a}$ = 12.9 Hz, $^3J_{H5b,H6a}$ = 7.2 Hz, $^3J_{H6a,H7}$ = 7.2 Hz, 1H, H-6a), 2.12 (s, 3H, H-14), 1.85 – 1.83 (m, 1H, H-6b). ^{13}C NMR (126 MHz, $CDCl_3$, 25ºC) δ 179.6 (C-10), 170.2 (C-13), 162.2 (C-9), 153.8 (C-3), 151.1 (C-1), 143.9 (C-7a), 143.6 (C-19), 141.7 (C-2), 141.2 (C-12), 135.5 (C-12a), 135.0 (C-4a), 134.7 (C-11), 134.0 (C-22), 129.4* (C-23,27), 129.3 (C-25), 128.3* (C-24,26), 125.8 (C-1a), 124.8 (C-20), 113.0 (C-8), 107.7 (C-4), 62.3 (C-18), 61.6 (C-15), 61.5 (C-16), 56.2 (C-17), 54.7 (C-21), 52.3 (C-7), 39.1 (C-6), 29.8 (C-5), 23.4 (C-14). (*-overlapped)

(*S*)-*N*-(9-((1-(4-cyanobenzyl)-1*H*-1,2,3-triazol-4-yl)methoxy)-1,2,3-trimethoxy-10-oxo-5,6,7,10-tetrahydrobenzo[*a*]heptalen-7-yl)acetamide (compound **5b**) Derivative **3f** 70 mg (0.17 mmol) was dissolved in a 6 mL mixture of THF/MeOH (3:1) and 4-(Azidomethyl)benzonitrile 0.331 mL (0.17 mmol) was added. Then, to each mixture, 6.5 mg (0.05 mmol) of CH_3COOCu(I) and 45 mg (0.25 mmol) of ascorbic acid was added. The solution was stirred at room temperature for a three hours and after this time, dichloromethane was added and extracted twice with 25 mL of ammonium hydroxide, twice with 25mL of water and finally twice with 25 mL of brine. The organic layer was evaporated and the synthesized derivative **5b** was purified by column chromatography with silica gel with dichloromethane/acetone (2:1) as an eluent. The product was obtained as a yellow powder. (23 mg, 24%) mp 124-128 °C. HPLC R_t= 8.467 min. Anal. Calcd for $C_{32}H_{31}N_5O_6$: C, 66.08; H, 5.37; N, 12.04. Found: C, 66.06; H, 5.38; N, 12.03; HRMS (ESI-TOF) m/z: $[M + H]^+$ Calcd 581.2274; Found 581.2272. FT-IR (KBr): ν$(N\text{-}H)_{amide}$ - 3287 cm^{-1}; ν(=CH) - 3058 cm^{-1}; ν(C-H) - 2934 cm^{-1}, 2853 cm^{-1}; ν(C≡N) - 2229 cm^{-1}; ν$(C{=}O)_{amide}$ - 1661 cm^{-1}; ν$(C{=}C)_{ar}$ - 1611 cm^{-1}.; δ$(N\text{-}H)_{amide}$ - 1560 cm^{-1}; ν(C=C) - 1489 cm^{-1}; ν$(C\text{-}O)_{as}$ - 1253 cm^{-1}; ν$(C\text{-}O)_s$ - 1166, 1096 cm^{-1}. ^1H NMR (500 MHz, $CDCl_3$, 25ºC) δ 7.83 (s, 1H, H-20), 7.66 (d, $^3J_{H24,26,H23,27}$ = 8.0 Hz, 2H, H-24,26), 7.63 (s, 1H, H-8), 7.39 (d, $^3J_{H11,H12}$ = 12.8 Hz, 1H, H-12), 7.34 (d, $^3J_{H24,26,H23,27}$ = 8.0 Hz, 2H, H-23,27), 7.12 (d, $^3J_{H11,H12}$ = 12.8 Hz, 1H, H-11), 6.81 (d, $^3J_{H7,NH7}$ = 7.7 Hz, 1H, NH-7), 6.54 (s, 1H, H-4), 5.59 (d, ^2J = 2.3 Hz, 2H, H-21), 5.47 (d, ^2J = 13.7 Hz, 1H, H-18), 5.43 (d, ^2J = 13.7 Hz, 1H, H-18), 4.69 (dt, $^3J_{H6b,H7}$ = 13.5 Hz, $^3J_{H6a,H7}$ = 6.9 Hz, $^3J_{H7,NH7}$ = 6.9 Hz, 1H, H-7), 3.92 (s, 3H, H-16), 3.89 (s, 3H, H-17), 3.66 (s, 3H, H-15), 2.45 (dd, ^2J = 12.9 Hz, $^3J_{H5b,H6a}$ = 6.0 Hz, 1H, H-5b), 2.31 (td, ^2J = 11.9 Hz, $^3J_{H5a,H6a}$ = 11.9 Hz, $^3J_{H5a,H6b}$ = 6.1 Hz, 1H, H-5a), 2.23 (tt, ^2J = 12.8 Hz, $^3J_{H5a,H6a}$ = 12.8 Hz, $^3J_{H5b,H6a}$ = 7.0 Hz, $^3J_{H6a,H7}$ = 7.0 Hz, 1H, H-6a), 2.09 (s, 3H, H-14), 1.88 – 1.82 (m, 1H, H-6b). ^{13}C NMR (126 MHz, $CDCl_3$, 25ºC) δ 179.6 (C-10), 170.1 (C-13), 162.1 (C-9), 153.9 (C-3), 151.0 (C-1),

144.0 (C-7a), 143.8 (C-19), 141.8 (C-2), 141.4 (C-12), 139.3 (C-22), 135.7 (C-12a), 134.9 (C-4a), 134.7 (C-11), 133.1* (C-24,26), 128.7* (C-23,27), 125.7 (C-1a), 125.0 (C-20), 118.1 (C-28), 113.2 (C-25), 113.1 (C-8), 107.7 (C-4), 62.2 (C-18), 61.6 (C-15), 61.5 (C-16), 56.2 (C-17), 53.9 (C-21), 52.4 (C-7), 39.1 (C-6), 29.8 (C-5), 23.3 (C-14). (*-overlapped)

(*S*)-*N*-(1,2,3-trimethoxy-9-((1-(4-nitrobenzyl)-1*H*-1,2,3-triazol-4-yl)methoxy)-10-oxo-5,6,7,10-tetrahydrobenzo[*a*]heptalen-7-yl)acetamide (compound **5c**) Derivative **3f** 70 mg (0.17 mmol) was dissolved in a 6 mL mixture of THF/MeOH (3:1) and 1-(azidomethyl)-4-nitrobenzene 30 mg (0.17 mmol) was added. Then, to each mixture, 6.5 mg (0.05 mmol) of $CH_3COOCu(I)$ and 45 mg (0.25 mmol) of ascorbic acid was added. The solution was stirred at room temperature for a three hours and after this time, dichloromethane was added and extracted twice with 25 mL of ammonium hydroxide, twice with 25mL of water and finally twice with 25 mL of brine. The organic layer was evaporated and the synthesized derivative **5c** was purified by column chromatography with silica gel with dichloromethane/acetone (2:1) as an eluent. The product was obtained as a yellow powder. (19 mg, 19%) mp 123-126 °C. HPLC R_t= 9.547 min. Anal. Calcd for $C_{31}H_{31}N_5O_8$: C, 61.89; H, 5.19; N, 11.64. Found: C, 61.88; H, 5.21; N, 11.62; HRMS (ESI-TOF) m/z: $[M + H]^+$ Calcd 601.2173; Found 601.2172. FT-IR (KBr): ν(N-H)$_{amide}$ - 3284 cm^{-1}; ν(=CH) - 3077 cm^{-1}; ν(C-H) - 2933 cm^{-1} , 2850 cm^{-1}; ν(C=O)$_{amide}$ - 1661 cm^{-1}; ν(C=C)$_{ar}$ - 1610 cm^{-1} ; δ(N-H)$_{amide}$ - 1560 cm^{-1}; ν(NO_2)$_{as}$ - 1522 cm^{-1}; ν(C=C) - 1489 cm^{-1}; ν(NO_2)$_s$ - 1348 cm^{-1}; ν(C-O)$_{as}$ - 1253 cm^{-1}.; ν(C-O)$_s$ - 1166, 1095 cm^{-1}. ^{1}H NMR (500 MHz, $CDCl_3$, 25ºC) δ 8.22 (d, $^3J_{H24,26,H23,27}$ = 8.2 Hz, 2H, H-24,26), 7.87 (s, 1H, H-20), 7.64 (s, 1H, H-8), 7.41 (d, $^3J_{H24,26,H23,27}$ = 8.5 Hz, 2H, H-23,27), 7.39 (d, $^3J_{H11,H12}$ = 12.3 Hz, 1H, H-12), 7.13 (d, $^3J_{H11,H12}$ = 12.8 Hz, 1H, H-11), 6.80 (d, $^3J_{H7,NH7}$ = 7.6 Hz, 1H, NH-7), 6.54 (s, 1H, H-4), 5.64 (s, 2H, H-21), 5.47 (d, ^2J = 14.2 Hz, 1H, H-18), 5.43 (d, ^2J = 13.8 Hz, 1H, H-18), 4.69 (dt, $^3J_{H6b,H7}$ = 13.3 Hz, $^3J_{H6a,H7}$ = 6.9 Hz, $^3J_{H7,NH7}$ = 6.9 Hz, 1H, H-7), 3.92 (s, 3H, H-16), 3.89 (s, 3H, H-17), 3.66 (s, 3H, H-15), 2.45 (dd, ^2J = 12.9 Hz, $^3J_{H5b,H6a}$ = 6.0 Hz, 1H, H-5b), 2.31 (dq, ^2J = 12.0 Hz, $^3J_{H5a,H6a}$ = 12.0 Hz, $^3J_{H5a,H6b}$ = 6.1 Hz, 1H, H-5a), 2.23 (tt, ^2J = 12.8 Hz, $^3J_{H5a,H6a}$ = 12.8 Hz, $^3J_{H5b,H6a}$ = 6.9 Hz, $^3J_{H6a,H7}$ = 6.9 Hz, 1H, H,-6a), 2.10 (s, 3H, H-14), 1.88 – 1.77 (m, 1H, H-6b). ^{13}C NMR (126 MHz, $CDCl_3$, 25ºC) δ 179.6 (C-10), 170.1 (C-13), 162.1 (C-9), 153.9 (C-3), 151.0 (C-1), 148.3 (C-25), 144.1 (C-7a), 143.9 (C-19), 141.8 (C-2), 141.4 (C-12), 141.1 (C-22), 135.7 (C-12a), 134.9 (C-4a), 134.7 (C-11), 128.9* (C-23,27), 125.7 (C-1a), 125.0 (C-20), 124.5* (C-24,26), 113.1 (C-8), 107.7 (C-4), 62.2 (C-18), 61.6 (C-15), 61.5 (C-16), 56.2 (C-17), 53.5 (C-21), 52.5 (C-7), 39.1 (C-6), 29.8 (C-5), 23.4 (C-14). (*-overlapped)

(2*R*,3*S*,4*R*,5*R*,6*R*)-5-acetamido-6-(4-((((*S*)-7-acetamido-1,2,3-trimethoxy-10-oxo-5,6,7,10-tetrahydrobenzo[*a*]heptalen-9-yl)oxy)methyl)-1*H*-1,2,3-triazol-1-yl)-2-(acetoxymethyl)tetrahydro-2*H*-pyran-3,4-diyl diacetate (compound **5d**) Derivative **3f** 70 mg (0.17 mmol) was dissolved in a 6 mL mixture of THF/MeOH (3:1) and 2-acetamido-2-deoxy-β-D-glucopyranosyl azide 3,4,6-triacetate 61.5 mg (0.17 mmol) was added. Then, to each mixture, 6.5 mg (0.05 mmol) of $CH_3COOCu(I)$ and 45 mg (0.25 mmol) of ascorbic acid was added. The solution was stirred at room temperature for a three hours and after this time, the

mixture was evaporated. Synthesized derivative **5d** was purified by column chromatography with silica gel with dichloromethane/acetone (2:1) as an eluent. The product was obtained as a yellow powder. (34 mg, 26%) mp 137-140 °C. Anal. Calcd for $C_{38}H_{45}N_5O_{14}$: C, 57.35; H, 5.70; N, 8.80. Found: C, 57.33; H, 5.69; N, 8.81; HRMS (ESI-TOF) m/z: $[M + H]^+$ Calcd 795.2963; Found 795.2961. FT-IR (KBr): ν(N-H)$_{amide}$ - 3276 cm^{-1}; ν(=CH) - 3061 cm^{-1}; ν(C-H) - 2932 cm^{-1} , 2855 cm^{-1}, ν(C=O)$_{ester}$ - 1750 cm^{-1}; ν(C=O)$_{amide}$ - 1663 cm^{-1}; ν(C=C)$_{ar.}$- 1612 cm^{-1}; δ(N-H)$_{amide}$ - 1560 cm^{-1}; ν(C=C) - 1490 cm^{-1}; ν(C-O)$_{as}$ - 1237 cm^{-1}; ν(C-O)$_s$ - 1167, 1096 cm^{-1}. ^{1}H NMR (500 MHz, $CDCl_3$, 25ºC) δ 8.17 (s, 1H, H-20), 7.58 (s, 1H, H-8), 7.42 (d, $^3J_{H11,H12}$ = 12.6 Hz, 1H, H-12), 7.16 (d, $^3J_{H11,H12}$ = 12.8 Hz, 1H, H-11), 6.88 (bs, 1H, H-21), 6.54 (s, 1H, H-4), 6.15 – 6.08 (m, 2H, NH-7,22), 5.51 (t, $^3J_{H22,H23}$ = 9.9 Hz, 1H, H-23), 5.46 (bs, 2H, H-18), 5.26 (t, $^3J_{H22,H23}$ = 9.9 Hz, 1H, H-23), 4.65 (bs, 1H, H-7), 4.50 (d, $^3J_{H24,H25}$ = 9.2 Hz, 1H, H-25), 4.29 (dd, ^2J = 12.6 Hz, $^3J_{H24,H32}$= 4.4 Hz, 1H, H-32), 4.16 (d, ^2J = 12.4 Hz, 1H, H-32), 4.04 (d, $^3J_{H24,H25}$ = 9.7 Hz, 1H, H-24), 3.91 (s, 3H, H-16), 3.89 (s, 3H, H-17), 3.67 (s, 3H, H-15), 2.45 (d, $^3J_{H5b,H6a}$ = 7.6 Hz, 1H, H-5b), 2.30 – 2.26 (m, 2H, H-5a,6a), 2.11 (s, 3H, H-14), 2.07 (s, 3H, H-29), 2.05 (s, 3H, H-31), 2.04 (s, 3H, H-34), 1.98 (bs, 1H, H-6b), 1.65 (s, 3H, H-27). ^{13}C NMR (126 MHz, $CDCl_3$, 25ºC) δ 179.6 (C-10), 170.8* (C-28,30,33), 170.5 (C-13), 169.5 (C-26), 162.2 (C-9), 153.9 (C-3), 150.9 (C-1), 144.3 (C-7a), 144.3 (C-19), 141.7 (C-2), 141.7 (C-12), 136.0 (C-12a), 135.1 (C-4a), 135.1 (C-11), 125.7* (C-1a,20), 113.8 (C-8), 107.6 (C-4), 86.2 (C-21), 75.1 (C-24), 72.3 (C-23), 68.0 (C-25), 62.5 (C-18), 61.8 (C-32), 61.6 (C-15), 61.5 (C-16), 56.3 (C-17), 54.1 (C-22), 52.6 (C-7), 38.8 (C-6), 30.1 (C-5), 23.3 (C-14), 22.9 (C-27), 20.9 (C-34), 20.8 (C-31), 20.7 (C-29). (*-overlapped)

N-((*S*)-9-((1-((2*S*,3*R*,5*R*)-2-(hydroxymethyl)-5-(5-methyl-2,4-dioxo-3,4-dihydropyrimidin-1(2*H*)-yl)tetrahydrofuran-3-yl)-1*H*-1,2,3-triazol-4-yl)methoxy)-1,2,3-trimethoxy-10-oxo-5,6,7,10-tetrahydrobenzo[*a*]heptalen-7-yl)acetamide (compound **5e**) Derivative **3f** 70 mg (0.17 mmol) was dissolved in a 6 mL mixture of THF/MeOH (3:1) and AZT 31.5 mg (0.17 mmol) was added. Then, to each mixture, 6.5 mg (0.05 mmol) of $CH_3COOCu(I)$ and 45 mg (0.25 mmol) of ascorbic acid was added. The solution was stirred at 60ºC for a three hours and after this time, the mixture was evaporated. Synthesized derivative **5e** was purified by column chromatography with silica gel with dichloromethane/methanol (25:1) as an eluent. The product was obtained as a yellow powder. (31 mg, 27%) mp 201-203 °C. HPLC R_t= 4.652 min. Anal. Calcd for $C_{34}H_{38}N_6O_{10}$: C, 59.12; H, 5.55; N, 12.17. Found: C, 59.13; H, 5.56; N, 12.18; HRMS (ESI-TOF) m/z: $[M + H]^+$ Calcd 690.2649; Found 690.2644. FT-IR (KBr): ν(N-H)*$_{amide, substituent,}$ ν(O-H)* - 3287 cm^{-1}; ν(=CH) - 3064 cm^{-1}; ν(C-H) - 2934 cm^{-1} ; ν(C=O)*$_{amide, substituent}$ - 1692 cm^{-1}; ν(C=C)$_{ar.}$- 1610 cm^{-1}; δ(N-H)$_{amide}$ - 1558 cm^{-1}; ν(C=C) - 1489 cm^{-1}; ν(C=C)$_{substituent}$ - 1457 cm^{-1} , ν(C-O)$_{as}$ - 1267 cm^{-1}; ν(C-O)$_s$ - 1140, 1096 cm^{-1}. ^{1}H NMR (500 MHz, $CDCl_3$, 25ºC) δ 10.07 (vbs, 1H, NH-28), 9.53 (vbs, 1H, OH-25), 8.09 (bs, 1H, H-20), 7.72 – 7.49 (m, 2H, H-8,26), 7.42 (d, $^3J_{H11,H12}$ = 12.2 Hz, 1H, H-12), 7.14 (d, $^3J_{H11,H12}$ = 12.3 Hz, 1H, H-11), 6.55 (s, 1H, H-4), 5.44 (bs, 3H, NH-7, H-18), 5.38 – 5.08 (m, 1H, H-23), 4.65 (bs, 1H, H-21), 4.48 (bs, 1H, H-7), 3.90 (s, 3H, H-16), 3.88 (s, 5H, H-17,25), 3.83 – 3.73 (m, 1H, H-22), 3.64 (bs, 2H, H-24), 3.63 (s, 3H, H-15), 2.45 (bs, 1H, H-5b), 2.33 – 2.21 (m, 2H, H-5a,6a), 2.16 (d, $^4J_{H14,NH7}$ = 3.3 Hz, 3H, H-14), 2.10 – 2.04 (m, 1H, H-6b), 1.97 – 1.86 (m, 3H, H-30). ^{13}C NMR (126 MHz, $CDCl_3$, 25ºC) δ 179.6 (C-10), 170.5 (C-13), 164.0 (C-28), 162.3 (C-9), 153.9 (C-3), 151.0 (C-1), 150.5 (C-29), 144.7 (C-7a), 144.7 (C-

19), 141.6 (C-2), 141.6 (C-12), 136.0 (C-12a), 135.7 (C-26), 135.1 (C-4a), 135.1 (C-11), 125.7 (C-1a), 125.6 (C-20), 113.5 (C-8), 111.1 (C-27), 107.7 (C-4), 85.8 (C-23), 83.6 (C-22), 69.7 (C-21), 66.7 (C-25), 62.4 (C-18), 61.6 (C-15), 61.5 (C-16), 56.2 (C-17), 52.6 (C-7), 38.8 (C-6), 31.9 (C-24), 29.8 (C-5), 23.3 (C-14), 12.6 (C-30). (*-overlapped)

(4*E*,6*Z*,8*S*,9*S*,10*E*,12*S*,13*R*,14*S*,16*R*)-19-((2-(4-((((*S*)-7-acetamido-1,2,3-trimethoxy-10-oxo-5,6,7,10-tetrahydrobenzo[*a*]heptalen-9-yl)oxy)methyl)-1*H*-1,2,3-triazol-1-yl)ethyl)amino)-13-hydroxy-8,14-dimethoxy-4,10,12,16-tetramethyl-3,20,22-trioxo-2-azabicyclo[16.3.1]docosa-1(21),4,6,10,18-pentaen-9-yl carbamate (compound **5f**) Derivative **3f** 70 mg (0.17 mmol) was in a 4 mL mixture of TBA/H_2O (1:1) and **9** 70 mg (0.11 mmol) was added. Then, to each mixture, 2 mg (0.02 mmol) of $CH_3COOCu(I)$ and 10 mg (0.06 mmol) of ascorbic acid was added. The solution was stirred at room temperature for a 72 hours and after this time, the mixture was evaporated. Synthesized derivative **5f** was purified by column chromatography with silica gel with dichloromethane/methanol (25:1) as an eluent. The product was obtained as a violet powder. (41 mg, 25%) mp 179-181 °C. HPLC R_t= 4.461 min. Anal. Calcd for $C_{54}H_{67}N_7O_{14}$: C, 62.47; H, 6.51; N, 9.44. Found: C, 62.46; H, 6.50; N, 9.43; HRMS (ESI-TOF) m/z: $[M + H]^+$ Calcd 1037.4746; Found 1037.4744. FT-IR (KBr): $\nu_s(N\text{-}H)_{carbamate}$ - 3449 cm^{-1}; $\nu_s(N\text{-}H)_{lactam}$ - 3335cm^{-1}; $\nu(N\text{-}H)^*_{amide}$, ν(O-H) - 3205 cm^{-1}; ν(=CH) - 3064 cm^{-1}; ν(C-H) - 2933 cm^{-1} ; $\nu(C{=}O)_{carbamate}$ - 1721 cm^{-1}; $\nu(C{=}O)^*_{lactam,\ amide}$ - 1685 cm^{-1}; $\nu(C{=}O)_{quinone}$ - 1650 cm^{-1}; ν(C=C) - 1610 cm^{-1}; $\delta(N\text{-}H)^*_{lactam,amide}$ - 1563 cm^{-1}; $\delta(N\text{-}H)_{carbamate}$ - 1489 cm^{-1}; $\nu(C\text{-}N)_{carbamate}$ - 1322 cm^{-1}; $\nu(C\text{-}O)_{as}$ - 1252 cm^{-1}; $\nu(C\text{- }O\text{-}C)_{carbamate}$ - 1193 cm^{-1}; $\nu(C\text{-}O)_s$ - 1140, 1096 cm^{-1} , $\nu(C\text{-}O)_{methoxy}$ - 1053 cm^{-1}. ^1H NMR (500 MHz, $CDCl_3$, 25ºC) δ 9.04 (s, 1H, 1'-NH), 8.04 (s, 1H, H-20), 7.62 (s, 1H, H-8), 7.39 (d, $^3J_{H11,H12}$ = 12.8 Hz, 1H, H-12), 7.29 (d, $^3J_{H7,NH7}$ = 7.6 Hz, 1H, NH-7), 7.15 (s, 1H, H-19'), 7.10 (d, $^3J_{H-11,H-12}$ = 12.7 Hz, 1H, H-11), 6.91 (d, $^3J_{H3',H4'}$ = 11.6 Hz, 1H, H-3'), 6.59 – 6.55 (m, 1H, NH-17'), 6.55 – 6.50 (m, 2H, H-4',4), 5.85 (t, $J^3_{H4',H5'}$ = 10.3 Hz, 1H, H-5'), 5.82 – 5.78 (m, 1H, H-9'), 5.41 (s, 2H, H-18), 5.12 (s, 1H, H-7'), 5.09 (s, 2H, NH_2-24'), 4.69 – 4.61 (m, 3H, H-7,29'), 4.30 – 4.25 (m, 1H, H-6'), 4.11 – 4.01 (m, 2H, 30'-H), 3.90 (s, 3H, H-16), 3.88 (s, 3H, H-17), 3.65 (s, 3H, H-15), 3.58 – 3.52 (m, 1H, H-11'), 3.41 – 3.36 (m, 1H, H-12'), 3.31 (s, 3H, H-27'), 3.24 (s, 3H, H-23'), 2.75 – 2.68 (m, 1H, H-10'), 2.64 (d, ^2J =13.6 Hz, 1H, H-15'), 2.47 – 2.40 (m, 1H, H-5b), 2.34 – 2.18 (m, 4H, H-5a,6a,15', OH-11'), 2.08 (s, 3H, H-14), 1.98 (s, 3H, H-22'), 1.96 – 1.88 (m, 1H, H-6b), 1.78 – 1.75 (m, 3H, H-25'), 1.75 – 1.71 (m, 2H, H-13'), 1.69 (s, 1H, H-14'), 0.95 (d, $^3J_{H10,H26}$= 6.9 Hz, 3H, H-26'), 0.90 (d, $J^3_{H14,H28}$= 6.4 Hz, 3H, H-28'). ^{13}C NMR (126 MHz, $CDCl_3$, 25ºC) δ 184.1 (C-18'), 180.9 (C-21'), 179.5 (C-10), 170.4 (C-13), 168.3 (C-1'), 162.2 (C-9), 156.4 (C-24'), 153.8 (C-3), 150.9 (C-1), 145.0 (C-17'), 144.5 (C-7a), 143.5 (C-19'), 141.6* (C-2,12), 140.7 (C-20'), 136.3 (C-5'), 135.8 (C-12a), 135.1 (C-4a), 134.9 (C-2'), 134.4 (C-11), 133.5 (C-9'), 133.3 (C-8'), 127.2 (C-3'), 126.5 (C-4'), 125.7 (C-1a), 125.5 (C-20), 113.3 (C-8), 110.8 (C-16'), 109.1 (C-19'), 107.7 (C-4), 81.7 (C-7'), 81.3 (C-6'), 81.3 (C-12'), 72.6 (C-11'), 62.3 (C-18), 61.6 (C-15), 61.5 (C-16), 57.2 (C-23'), 56.8 (C-27'), 56.2 (C-17), 52.6 (C-7), 49.8 (C-29'), 45.2 (C-30'), 38.8 (C-6), 35.0 (C-13'), 34.2 (C-15'), 32.4 (C-10'), 30.1 (C-5), 28.8 (C-14'), 23.2 (C-28'), 23.0 (C-14), 12.9 (C-25'), 12.6 (C-22'), 12.6 (C-26'). (*-overlapped)

(S)-N-(10-((1-benzyl-1H-1,2,3-triazol-4-yl)methoxy)-1,2,3-trimethoxy-9-oxo-5,6,7,9-tetrahydrobenzo[a]heptalen-7-yl)acetamide (compound **6a**) Derivative **4f** 70 mg (0.17 mmol) was dissolved in a 6 mL mixture of THF/MeOH (3:1) and benzyl azide 22 mg (0.17 mmol) was added. Then, to each mixture, 6.5 mg (0.05 mmol) of $CH_3COOCu(I)$ and 45 mg (0.25 mmol) of ascorbic acid was added. The solution was stirred at room temperature for a three hours and after this time, dichloromethane was added and extracted twice with 25 mL of ammonium hydroxide, twice with 25mL of water and finally twice with 25 mL of brine. The organic layer was evaporated and the synthesized derivative **6a** was purified by column chromatography with silica gel with dichloromethane/acetone (2:1) as an eluent. The product was obtained as a yellow powder. (23 mg, 25%) mp 116-119 °C. HPLC R_t= 9.187min. Anal. Calcd for $C_{31}H_{32}N_4O_6$: C, 66.89; H, 5.79; N, 10.07. Found: C, 66.90; H, 5.78; N, 10.06; HRMS (ESI-TOF) m/z: $[M + H]^+$ Calcd 556.2322; Found 556.2321. FT-IR (KBr): ν(N-H)$_{amide}$ - 3281 cm^{-1}; ν(=CH) - 3061 cm^{-1}; ν(C-H) - 2933 cm^{-1} , 2853 cm^{-1}; ν(C=O)$_{amide}$ - 1661 cm^{-1}; ν(C=C)$_{ar.}$- 1614 cm^{-1}; δ(N-H)$_{amide}$ - 1558 cm^{-1}; ν(C=C) - 1487 cm^{-1}; ν(C-O)$_{as}$.- 1237 cm^{-1}; ν(C-O)$_{sym}$ - 1171, 1094 cm^{-1}. ^{1}H NMR (500 MHz, $CDCl_3$, 25ºC) δ 7.64 (s, 1H, H-20), 7.43 (s, 1H, H-8), 7.36 (s, 1H, H-12), 7.36 – 7.34 (m, 2H, H-24,26), 7.31 – 7.26 (m, 1H, H-25), 7.27 – 7.25 (m, 2H, H-23,27), 7.19 (d, $^3J_{H11,H12}$ = 10.8 Hz, 1H, H-11), 7.07 (d, $^3J_{H7,NH7}$ = 6.7 Hz, 1H, NH-7), 6.51 (s, 1H, H-4), 5.52 (s, 2H, H-21), 5.41 (d, ^2J = 13.1 Hz, 1H, H-18), 5.38 (d, ^2J = 13.1 Hz, 1H, H-18), 4.60 (dt, $^3J_{H6b,H7}$ = 12.4 Hz, $^3J_{H6a,H7}$ = 6.6 Hz, $^3J_{H7,NH7}$ = 6.6 Hz, 1H, H-7), 3.93 (s, 3H, H-16), 3.89 (s, 3H, H-17), 3.62 (s, 3H, H-15), 2.49 (dd, ^2J = 13.5 Hz, $^3J_{H5b,H6a}$ = 6.3 Hz, 1H, H-5b), 2.35 (td, ^2J = 13.2 Hz, $^3J_{H5a,H6a}$ =13.2 Hz, $^3J_{H5a,H6b}$ = 6.7 Hz, 1H, H-5a), 2.23 (tt, ^2J = 12.6 Hz, $^3J_{H5a,H6a}$ = 12.6 Hz, $^3J_{H5b,H6a}$ = 6.2 Hz, $^3J_{H6a,H7}$ = 6.2 Hz, 1H, H-6a), 1.96 (s, 3H, H-14), 1.83 – 1.80 (m, 1H, H-6b). ^{13}C NMR (126 MHz, $CDCl_3$, 25ºC) δ 179.7 (C-9), 169.9 (C-13), 162.7 (C-10), 153.7 (C-3), 151.7 (C-7a), 151.4 (C-1), 143.2 (C-19), 141.9 (C-2), 137.5 (C-12a), 135.5 (C-12), 134.3 (C-22), 134.2 (C-4a), 131.2 (C-8), 129.3* (C-23,27), 129.0 (C-25), 128.4* (C-24,26), 125.7 (C-1a), 123.7 (C-20), 115.2 (C-11), 107.4 (C-4), 63.2 (C-18), 61.6 (C-15), 61.5 (C-16), 56.3 (C-17), 53.9 (C-21), 52.5 (C-7), 36.8 (C-6), 30.0 (C-5), 23.1 (C-14). (*-overlapped)

(S)-N-(10-((1-(4-cyanobenzyl)-1H-1,2,3-triazol-4-yl)methoxy)-1,2,3-trimethoxy-9-oxo-5,6,7,9-tetrahydrobenzo[a]heptalen-7-yl)acetamide (compound **6b**) Derivative **4f** 70 mg (0.17 mmol) was dissolved in a 6 mL mixture of THF/MeOH (3:1) and 4-(Azidomethyl)benzonitrile 0.331 mL (0.17 mmol) was added. Then, to each mixture, 6.5 mg (0.05 mmol) of $CH_3COOCu(I)$ and 45 mg (0.25 mmol) of ascorbic acid was added. The solution was stirred at room temperature for a three hours and after this time, dichloromethane was added and extracted twice with 25 mL of ammonium hydroxide, twice with 25mL of water and finally twice with 25 mL of brine. The organic layer was evaporated and the synthesized derivative **6b** was purified by column chromatography with silica gel with dichloromethane/acetone (2:1) as an eluent. The product was obtained as a yellow powder. (22 mg, 24%) mp 128-132 °C. HPLC R_t= 8.047 min. Anal. Calcd for $C_{32}H_{31}N_5O_6$: C, 66.08; H, 5.37; N, 12.04. Found: C, 66.09; H, 5.38; N, 12.05; HRMS (ESI-TOF) m/z: $[M + H]^+$ Calcd 581.2274; Found 581.2275. FT-IR (KBr): ν(N-H)$_{amide}$ - 3280 cm^{-1}; ν(=CH) - 3057 cm^{-1}; ν(C-H) - 2934 cm^{-1}, 2850 cm^{-1}; ν(C≡N) - 2229 cm^{-1}; ν(C=O)$_{amide}$ - 1661 cm^{-1}; ν(C=C)$_{ar}$ - 1611 cm^{-1}.; δ(N-H)$_{amide}$ - 1556 cm^{-1}; ν(C=C) - 1487 cm^{-1}; ν(C-O)$_{as}$ - 1237 cm^{-1}; ν(C-O)$_s$ - 1171,

1094 cm^{-1}. ^{1}H NMR (500 MHz, $CDCl_3$, 25ºC) δ 7.75 (s, 1H, H-20), 7.66 (d, $^3J_{H24,26,H23,27}$ = 8.3 Hz, 2H, H-24,26), 7.40 (s, 1H, H-8), 7.36 (d, $^3J_{H24,26,H23,27}$ = 8.3 Hz, 2H, H-23,27), 7.27 (d, $^3J_{H11,H12}$ = 12.2 Hz, 1H, H-12), 7.15 (d, $^3J_{H11,H12}$ = 10.8 Hz, 1H, H-11), 6.78 (d, $^3J_{H7,NH7}$ = 6.7 Hz, 1H, NH-7), 6.52 (s, 1H, H-4), 5.61 (d, ^2J = 15.5 Hz, 1H, H-21), 5.57 (d, ^2J = 15.5 Hz, 1H, H-21), 5.43 (d, ^2J = 13.0 Hz, 1H, H-18), 5.39 (d, ^2J = 12.9 Hz, 1H, H-18), 4.59 (dt, $^3J_{H6,H7}$ = 12.6 Hz, $^3J_{H6a,H7}$ = 6.6 Hz, $^3J_{H7,NH7}$ = 6.6 Hz, 1H, H-7), 3.93 (s, 3H, H-16), 3.89 (s, 3H, H-17), 3.63 (s, 3H, H-15), 2.51 (dd, ^2J = 13.6 Hz, $^3J_{H5b,H6a}$ = 6.3 Hz, 1H, H-5b), 2.36 (td, ^2J = 13.2 Hz, $^3J_{H5a,H6a}$ = 13.2 Hz, $^3J_{H5a,H6b}$ = 6.8 Hz, 1H, H-5a), 2.23 (tt, ^2J = 12.6 Hz, $^3J_{H5a,H6a}$ = 12.6 Hz, $^3J_{H5b,H6a}$ = 6.1 Hz, $^3J_{H6a,H7}$ = 6.1 Hz, 1H, H-6a), 1.96 (s, 3H, H-14), 1.80 (dt, ^2J = 11.8 Hz, $^3J_{H5a,H6b}$ = 5.3 Hz, 1H, H-6b). ^{13}C NMR (126 MHz, $CDCl_3$, 25ºC) δ 179.6 (C-9), 169.8 (C-13), 162.6 (C-10), 153.7 (C-3), 151.6 (C-7a), 151.4 (C-1), 143.8 (C-19), 141.9 (C-2), 139.6 (C-22), 137.5 (C-12a), 135.3 (C-12), 134.1 (C-4a), 133.1* (C-24,26), 131.3 (C-8), 128.7* (C-23,27), 125.6 (C-1a), 123.9 (C-20), 118.2 (C-28), 115.1 (C-11), 113.0 (C-25), 107.5 (C-4), 63.1 (C-18), 61.6 (C-15), 61.5 (C-16), 56.3 (C-17), 53.9 (C-21), 52.5 (C-7), 36.9 (C-6), 29.9 (C-5), 23.1 (C-14). (*-overlapped)

(*S*)-*N*-(1,2,3-trimethoxy-10-((1-(4-nitrobenzyl)-1*H*-1,2,3-triazol-4-yl)methoxy)-9-oxo-5,6,7,9-tetrahydrobenzo[*a*]heptalen-7-yl)acetamide (compound **6c**) Derivative **4f** 70 mg (0.17 mmol) was dissolved in a 6 mL mixture of THF/MeOH (3:1) and 1-(azidomethyl)-4-nitrobenzene 30 mg (0.17 mmol) was added. Then, to each mixture, 6.5 mg (0.05 mmol) of $CH_3COOCu(I)$ and 45 mg (0.25 mmol) of ascorbic acid was added. The solution was stirred at room temperature for a three hours and after this time, dichloromethane was added and extracted twice with 25 mL of ammonium hydroxide, twice with 25mL of water and finally twice with 25 mL of brine. The organic layer was evaporated and the synthesized derivative **6c** was purified by column chromatography with silica gel with dichloromethane/acetone (2:1) as an eluent. The product was obtained as a yellow powder. (21 mg, 21%) mp 120-123 °C. HPLC R_t= 9.480 min. Anal. Calcd for $C_{31}H_{31}N_5O_8$: C, 61.89; H, 5.19; N, 11.64. Found: C, 61.88; H, 5.20; N, 11.63; HRMS (ESI-TOF) m/z: $[M + H]^+$ Calcd 601.2173; Found 601.2171. FT-IR (KBr): $\nu(N\text{-}H)_{amide}$ - 3280 cm^{-1}; ν(=CH) - 3075 cm^{-1}; ν(C-H) - 2933 cm^{-1} , 2852 cm^{-1}; $\nu(C=O)_{amide}$ - 1661 cm^{-1}; $\nu(C=C)_{ar}$ - 1612 cm^{-1} ; $\delta(N\text{-}H)_{amide}$ - 1556 cm^{-1}; $\nu(NO_2)_{as}$ - 1523 cm^{-1}; ν(C=C) - 1487 cm^{-1}; $\nu(NO_2)_s$ - 1348 cm^{-1}; $\nu(C\text{-}O)_{as}$ - 1237 cm^{-1}.; $\nu(C\text{-}O)_s$ - 1171, 1094 cm^{-1}. ^{1}H NMR (500 MHz, $CDCl_3$, 25ºC) δ 8.22 (d, $^3J_{H24,26,H23,27}$ = 8.7 Hz, 2H, H-24,26), 7.79 (s, 1H, H-20), 7.43 (d, $^3J_{H24,26,H23,27}$ = 8.5 Hz, 2H, H-23,27), 7.40 (s, 1H, H-8) 7.27 (d, $^3J_{H11,H12}$ = 11.0 Hz, 1H, H-12), 7.16 (s, 1H, H-11), 6.81 (d, $^3J_{H7,NH7}$ = 6.7 Hz, 1H, NH-7), 6.51 (s, 1H, H-4), 5.64 (d, ^2J = 15.5 Hz, 1H, H-21), 5.62 (d, ^2J = 15.5 Hz, 1H, H-21), 5.43 (d, ^2J = 12.9 Hz, 1H, H-18), 5.39 (d, ^2J = 12.8 Hz, 1H, H-18), 4.59 (dt, $^3J_{H6b,H7}$ = 12.6 Hz, $^3J_{H6a,H7}$ = 6.5 Hz, $^3J_{H7,NH7}$ = 6.5 Hz, 1H, H-7), 3.93 (s, 3H, H-16), 3.89 (s, 3H, H-17), 3.62 (s, 3H, H-15), 2.50 (dd, ^2J = 13.5 Hz, $^3J_{H5b,H6a}$= 6.3 Hz, 1H, H-5b), 2.36 (td, ^2J = 13.3 Hz, $^3J_{H5a,H6a}$ = 13.3 Hz, $^3J_{H5a,H6b}$ = 6.8 Hz, 1H, H-5a), 2.22 (tt, ^2J = 13.0 Hz, $^3J_{H5a,H6a}$ = 13.0 Hz, $^3J_{H5b,H6a}$ = 6.6 Hz, $^3J_{H6a,H7}$ = 6.6 Hz, 1H, 6a), 1.96 (s, 3H, H-14), 1.79 (td, ^2J = 11.8 Hz, $^3J_{H5a,H6b}$ = 6.6 Hz, 1H, H-6b). ^{13}C NMR (126 MHz, $CDCl_3$, 25ºC) δ 179.6 (C-9), 169.8 (C-13), 162.6 (C-10), 153.7 (C-3), 151.6 (C-7a), 151.4 (C-1), 148.3 (C-25), 143.9 (C-19), 141.9 (C-2), 141.4 (C-22), 137.6 (C-12a), 135.3 (C-12), 134.1 (C-4a), 131.3 (C-8), 129.0* (C-23,27), 125.6 (C-1a),

124.5* (C-24,26), 124.0 (C-20), 115.1 (C-11), 107.5 (C-4), 63.1 (C-18), 61.6 (C-15), 61.5 (C-16), 56.3 (C-17), 53.4 (C-21), 52.5 (C-7), 36.8 (C-6), 29.9 (C-5), 23.1 (C-14). (*-overlapped)

(2*R*,3*S*,4*R*,5*R*,6*R*)-5-acetamido-6-(4-((((*S*)-7-acetamido-1,2,3-trimethoxy-9-oxo-5,6,7,9-tetrahydrobenzo[*a*]heptalen-10-yl)oxy)methyl)-1*H*-1,2,3-triazol-1-yl)-2-(acetoxymethyl)tetrahydro-2*H*-pyran-3,4-diyl diacetate (compound **6d**) Derivative **4f** 70 mg (0.17 mmol) was dissolved in a 6 mL mixture of THF/MeOH (3:1) and 2-Acetamido-2-deoxy-β-D-glucopyranosyl azide 3,4,6-triacetate 61.5 mg (0.17 mmol) was added. Then, to each mixture, 6.5 mg (0.05 mmol) of $CH_3COOCu(I)$ and 45 mg (0.25 mmol) of ascorbic acid was added. The solution was stirred at room temperature for a three hours and after this time, the mixture was evaporated. Synthesized derivative **6d** was purified by column chromatography with silica gel with dichloromethane/acetone (2:1) as an eluent. The product was obtained as a yellow powder. (25 mg, 19%) mp 108-110 °C. Anal. Calcd for $C_{38}H_{45}N_5O_{14}$: C, 57.35; H, 5.70; N, 8.80. Found: C, 57.34; H, 5.71; N, 8.81; HRMS (ESI-TOF) m/z: $[M + H]^+$ Calcd 795.2963; Found 795.2964. FT-IR (KBr): $\nu(N\text{-}H)_{amide}$ - 3279 cm^{-1}; ν(=CH) - 3060 cm^{-1}; ν(C-H) - 2934 cm^{-1} , 2855 cm^{-1}; $\nu(C{=}O)_{ester}$ - 1750 cm^{-1}; $\nu(C{=}O)_{amide}$ - 1666 cm^{-1}; $\nu(C{=}C)_{ar.}$- 1615 cm^{-1}; $\delta(N\text{-}H)_{amide}$ - 1554 cm^{-1}; ν(C=C) - 1488 cm^{-1}; $\nu(C\text{-}O)_{as}$ - 1238 cm^{-1}; $\nu(C\text{-}O)_{s}$ - 1171, 1045 cm^{-1}.^{1}H NMR (500 MHz, $CDCl_3$, 25ºC) δ 8.15 (s, 1H, H-20), 7.44 (s, 1H, H-8), 7.31 (d, $^3J_{H11,H12}$ = 10.6 Hz, 1H, H-12), 7.14 (d, $^3J_{H11,H12}$ = 10.7 Hz, 1H, H-11), 6.81 (bs, 2H, H-21, NH-22), 6.52 (s, 1H, H-4), 6.19 (d, $^3J_{H7,NH7}$ = 9.8 Hz, 1H, NH-7), 5.55 (t, $^3J_{H22,H23}$ = 9.9 Hz, 1H, H-23), 5.36 (d, ^2J = 12.2 Hz, 1H, H-18), 5.35 (d, ^2J = 12.2 Hz, 1H, H-18), 5.23 (t, $^3J_{H22,H23}$ = 9.5 Hz, 1H, H-22), 4.65 (s, 1H, H-7), 4.52 (d, $^3J_{H24,H25}$ = 9.6 Hz, 1H, H-25), 4.28 (dd, ^2J = 12.6 Hz, $^4J_{H24,H32}$ = 4.7 Hz, 1H, H-32), 4.14 (d, ^2J = 11.6 Hz, 1H, H-32), 4.03 (dd, $^3J_{H24,H25}$ = 9.2 Hz, $^4J_{H24,H32}$ = 4.6 Hz, 1H, 24), 3.93 (s, 3H, H-16), 3.89 (s, 3H, H-17), 3.64 (s, 3H, H-15), 2.54 – 2.49 (m, 1H, H-5b), 2.40 (s, 1H, H-5a), 2.24 (s, 1H, H-6a), 2.06 (s, 3H, H-34), 2.05 – 2.04 (m, 6H, H-29,31), 1.97 (s, 3H, H-14), 1.91 (s, 1H, H-6b), 1.74 (s, 3H, H-27).^{13}C NMR (126 MHz, $CDCl_3$, 25ºC) δ 179.6 (C-9), 170.9* (C-28,30), 170.8 (C-33), 169.8 (C-13), 169.5 (C-26), 162.8 (C-10), 153.7 (C-3), 151.7 (C-7a), 151.3 (C-1), 144.6 (C-19), 141.8 (C-2), 137.8 (C-12a), 135.4 (C-12), 134.2 (C-4a), 131.5 (C-8), 125.7 (C-1a), 123.4 (C-20), 115.2 (C-11), 107.5 (C-4), 85.9 (C-21), 75.0 (C-24), 72.5 (C-23), 68.2 (C-25), 62.9 (C-18), 61.9 (C-32), 61.6 (C-15), 61.5 (C-16), 56.3 (C-17), 54.0 (C-22), 52.3 (C-7), 36.9 (C-6), 30.0 (C-5), 23.1 (C-14), 23.0 (C-27), 20.9 (C-34), 20.8 (C-31), 20.7 (C-29). (*-overlapped)

N-((7*S*)-10-((1-((2*S*,5*R*)-2-(hydroxymethyl)-5-(5-methyl-2,4-dioxo-3,4-dihydropyrimidin-1(2*H*)-yl)tetrahydrofuran-3-yl)-1*H*-1,2,3-triazol-4-yl)methoxy)-1,2,3-trimethoxy-9-oxo-5,6,7,9-tetrahydrobenzo[*a*]heptalen-7-yl)acetamide (compound **6e**) Derivative **4f** 70 mg (0.17 mmol) was dissolved in a 6 mL mixture of THF/MeOH (3:1) and AZT 31.5 mg (0.17 mmol) was added. Then, to each mixture, 6.5 mg (0.05 mmol) of $CH_3COOCu(I)$ and 45 mg (0.25 mmol) of ascorbic acid was added. The solution was stirred at 60ºC for a three hours and after this time, the mixture was evaporated. Synthesized derivative **6e** was purified by column chromatography with silica gel with dichloromethane/methanol (25:1) as an eluent. The product was obtained as a yellow powder. (11 mg, 10%) mp 209-212 °C. HPLC R_t= 4.365 min. Anal. Calcd for $C_{34}H_{38}N_6O_{10}$: C, 59.12; H, 5.55; N, 12.17. Found: C, 59.13; H, 5.54; N, 12.16; HRMS (ESI-TOF) m/z: $[M + H]^+$ Calcd 690.2649; Found 690.2647. FT-IR (KBr):

ν(N-H)*$_{amide, substituent,}$ ν(O-H)* - 3415 cm^{-1}; ν(=CH) - 3062 cm^{-1}; ν(C-H) - 2934 cm^{-1} ; ν(C=O)*$_{amide, substituent}$ - 1689 cm^{-1}; ν(C=C)$_{ar.-}$ 1613 cm^{-1}; δ(N-H)$_{amide}$ - 1550 cm^{-1}; ν(C=C) - 1487 cm^{-1}; ν(C=C)$_{substituent}$ - 1463 cm^{-1} , ν(C-O)$_{as}$ - 1272 cm^{-1}; ν(C-O)$_{s}$ - 1142, 1095 cm^{-1}. ^{1}H NMR (500 MHz, Chloroform-d) δ 10.08 (vbs, 1H, 28-NH), 8.27 (bs, 1H, H-20), 7.71 (s, 1H, H-8), 7.55 – 7.28 (m, 2H, H-12,26), 7.16 (s, 1H, H-11), 6.52 (s, 1H, H-4), 6.29 (s, 1H, NH-7), 5.63 – 5.14 (m, 2H, H-18), 5.11 – 4.98 (m, 1H, H-23), 4.59 (bs, 1H, H-21), 4.39 (s, 1H, H-7), 3.90 (s, 5H, H-16,25), 3.89 (s, 4H, H-17,22), 3.83 – 3.77 (m, 2H, H-24), 3.62 (s, 3H, H-15), 2.88 (s, 1H, OH-25), 2.58 – 2.38 (m, 1H, H-5b), 2.38 – 2.24 (m, 1H, H-5a), 2.20 – 2.00 (m, 1H, H-6a), 1.95 (bs, 4H, H-6b,14), 1.81 (s, 3H, H-30). ^{13}C NMR (126 MHz, $CDCl_3$, 25ºC) δ 179.6 (C-9), 170.4 (C-13), 164.5 (C-28), 162.2 (C-10), 153.8 (C-3), 151.2 (C-7a), 150.9 (C-1), 150.4 (C-29), 144.6 (C-19), 141.7 (C-2), 138.1 (C-12a), 135.6 (C-26), 135.5 (C-12), 134.4* (C-4a,8), 125.5* (C-1a,20), 115.8 (C-11), 110.9 (C-27), 107.6 (C-4), 85.1 (C-23), 83.8 (C-22), 70.5 (C-21), 66.3 (C-25), 62.9 (C-18), 61.6 (C-15), 61.5 (C-16), 56.3 (C-17), 52.4 (C-7), 38.3 (C-6), 32.1 (C-24), 29.8 (C-5), 23.0 (C-14), 12.6 (C-30). (*-overlapped)

(4*E*,6*Z*,8*S*,9*S*,10*E*,12*S*,13*R*,14*S*,16*R*)-19-((2-(4-((((*S*)-7-acetamido-1,2,3-trimethoxy-9-oxo-5,6,7,9-tetrahydrobenzo[*a*]heptalen-10-yl)oxy)methyl)-1*H*-1,2,3-triazol-1-yl)ethyl)amino)-13-hydroxy-8,14-dimethoxy-4,10,12,16-tetramethyl-3,20,22-trioxo-2-azabicyclo[16.3.1]docosa-1(21),4,6,10,18-pentaen-9-yl carbamate (compound **6f**) Derivative **4f** 70 mg (0.17 mmol) was dissolved in a 4 mL mixture of TBA/H_2O (1:1) and **9** 70 mg (0.11 mmol) was added. Then, to each mixture, 6.5 mg (0.05 mmol) of $CH_3COOCu(I)$ and 45 mg (0.25 mmol) of ascorbic acid was added. The solution was stirred at room temperature for a 72 hours and after this time, the mixture was evaporated. Synthesized derivative **6f** was purified by column chromatography with silica gel with dichloromethane/methanol (25:1) as an eluent. The product was obtained as a violet powder. (71 mg, 41%) mp 181-183 °C. HPLC R_t= 4.374 min. Anal. Calcd for $C_{54}H_{67}N_7O_{14}$: C, 62.47; H, 6.51; N, 9.44. Found: C, 62.48; H, 6.50; N, 9.45; HRMS (ESI-TOF) m/z: $[M + H]^+$ Calcd 1037.4746; Found 1037.4748. FT-IR (KBr): $ν_s$(N-H)$_{carbamate}$ - 3463 cm^{-1}; $ν_s$(N-H)$_{lactam}$ - 3334cm^{-1}; ν(N-H)*$_{amide,}$ ν(O-H) - 3201 cm^{-1}; ν(=CH) - 3068 cm^{-1}; ν(C-H) - 2934 cm^{-1} ; ν(C=O)$_{carbamate}$ - 1720 cm^{-1}; ν(C=O)*$_{lactam, amide}$ - 1685 cm^{-1}; ν(C=O)$_{quinone}$ - 1651 cm^{-1}; ν(C=C) - 1613 cm^{-1}; δ(N-H)*$_{lactam,amide}$ - 1586 cm^{-1}; δ(N-H)$_{carbamate}$ - 1488 cm^{-1}; ν(C-N)$_{carbamate}$ - 1322 cm^{-1}; ν(C-O)$_{as}$ - 1241 cm^{-1}; ν(C- O-C)$_{carbamate}$ - 1193 cm^{-1}; ν(C-O)$_{s}$ - 1141, 1096 cm^{-1} ; ν(C-O)$_{methoxy}$ - 1050 cm^{-1} .^{1}H NMR (500 MHz, $CDCl_3$, 25ºC) δ 9.07 (s, 1H, NH-1'), 8.02 (s, 1H, H-20), 7.47 (d, $^{3}J_{H7,NH7}$ = 6.5 Hz, 1H, NH-7), 7.44 (s, 1H, H-8), 7.30 – 7.27 (m, 1H, H-12), 7.15 (s, 1H, H-19'), 7.12 (s, 1H, H-11), 6.91 (d, $J^{3}_{H3',H4'}$ = 11.7 Hz, 1H, H-3'), 6.54 (d, $J^{3}_{H3',H4'}$ = 11.2 Hz, 1H, H-4'), 6.50 (s, 2H, H-4, NH-17'), 5.85 – 5.81 (m, 1H, H-5'), 5.81 – 5.78 (m, 1H, H-9'), 5.32 (d, ^{2}J = 12.5 Hz, 1H, H-18), 5.28 (d, ^{2}J = 12.5 Hz, 1H, H-18), 5.25 (s, 2H, NH_2-24'), 5.10 (s, 1H, H-7'), 4.69 – 4.62 (m, 1H, H-29'), 4.61 – 4.53 (m, 2H, H-7,29'), 4.30 – 4.24 (m, 1H, H-6'), 4.16 – 4.07 (m, 1H, 30'-H), 4.08 – 3.99 (m, 1H, H-30'), 3.89 (s, 3H, H-16), 3.87 (s, 3H, H-17), 3.60 (s, 3H, H-15), 3.59 – 3.52 (m, 1H, H-11'), 3.40 – 3.33 (m, 1H, H-12'), 3.30 (s, 3H, H-27'), 3.23 (s, 3H, H-23'), 2.75 – 2.67 (m, 1H, H-10'), 2.63 (d, ^{2}J =13.3 Hz, 1H, H-15'), 2.60 – 2.52 (m, 1H, OH-11'), 2.48 (dd, ^{2}J = 13.4 Hz, $^{3}J_{H5b,H6a}$ = 6.1 Hz, 1H, H-5b), 2.34 (dq, $^{3}J_{H5a,H6a}$ = 13.0 Hz, $^{3}J_{H5a,H6b}$ = 7.0 Hz, 1H, H-5a), 2.28 – 2.22 (m, 1H, H-15'), 2.20 – 2.15 (m, 1H, H-6a), 1.97 (s, 3H, H-22'), 1.94 (s, 3H, H-14), 1.86 – 1.78 (m, 1H, H-6b), 1.75 (s, 3H, H-25'), 1.73 (s, 2H, H-13'), 1.67 (s, 1H, H-14'), 0.93

(d, $^{3}J_{H10,H26}$= 6.8 Hz, 3H, H-26'), 0.88 (d, $^{3}J_{H14,H28}$= 6.3 Hz, 3H, H-28'). ^{13}C NMR (126 MHz, $CDCl_3$, 25ºC) δ 184.3 (C-18'), 180.6 (C-21'), 179.5 (C-9), 170.1 (C-13), 168.3 (C-1'), 162.6 (C-10), 156.5 (C-24'), 153.6 (C-3), 152.1 (C-7a), 151.1 (C-1), 145.3 (C-17'), 142.4 (C-19), 141.6 (C-2), 140.5 (C-20'), 137.6 (C-12a),136.1 (C-5'), 135.3 (C-12), 134.9 (C-2'), 134.2 (C-4a), 133.5 (C-9'), 133.2 (C-8'), 131.3 (C-8), 127.1 (C-3'), 126.5 (C-4'), 125.5 (C-1a), 125.2 (C-20), 114.8 (C-11), 110.9 (C-16'), 109.2 (C-19'), 107.4 (C-4), 81.6 (C-7'), 81.3 (C-6'), 81.3 (C-12'), 72.6 (C-11'), 62.7 (C-18), 61.5 (C-15), 61.4 (C-16), 57.1 (C-23'), 56.8 (C-27'), 56.1 (C-17), 52.3 (C-7), 49.8 (C-29'), 45.2 (C-30'), 36.5 (C-6), 34.9 (C-13'), 34.2 (C-15'), 32.3 (C-10'), 29.9 (C-5), 28.7 (C-14'), 22.9* (C-14,28'), 12.9 (C-25'), 12.6 (C-22'), 12.5 (C-26'). (*-overlapped)

(*S*,*E*)-*N*-(1,2,3-trimethoxy-9-((4-(2-(naphthalen-2-yl)vinyl)benzyl)oxy)-10-oxo-5,6,7,10-tetrahydrobenzo[*a*]heptalen-7-yl)acetamide (compound **7a**). Derivative **3b** 60 mg (0.12 mmol) was dissolved in a 1 mL DMF and 2-vinylnaphthalene 37 mg (0.24 mmol) was added. Then 1.42 mg (0.006 mmol) of $Pd(OCOCH_3)_2$ and 49.7 mg (0.36 mmol) of K_2CO_3 was added. The solution was stirred at 90 ºC for a 4 h after this time, the reaction mixture was extracted in the solvent system EtOAc:H_2O. The organic layer was evaporated and the synthesized derivative **7a** was purified by column chromatography with silica gel with dichloromethane/acetone (from 4:1 to 2:1) as an eluent. The product was obtained as a cream-colored powder. (24 mg, 40%): HPLC Rt = 13.858, Anal. Calcd $C_{40}H_{37}NO_6$: C, 76.53; H, 5.94; N, 2.23. Found C, 76.51; H, 5.93; N, 2.25; HRMS (ESI-TOF) m/z: $[M + Na]^+$ Calcd for $C_{40}H_{37}NNaO_6^+$ 650.2519; Found 650.2523. ^{1}H NMR (500 MHz, $CDCl_3$, T = 298 K) δ 7.82 (d, $^{4}J_{H28, H36}$ = 1.9 Hz, 1H, H-36), 7.79 (d, $^{3}J_{H28, H29}$ = 8.8 Hz, 1H, H-29), 7.79 (m, 1H, H-34), 7.78 (m, 1H, H-31), 7.69 (dd, $^{3}J_{H28, H29}$ = 8.7 Hz, $^{4}J_{H28, H36}$ = 1.9 Hz, 1H, H-28), 7.53 (d, $^{3}J_{H20,H21 \text{ and } H23,H24}$ = 8.3 Hz, 2H, H-21 + H-23), 7.46 (m, 1H, H-32), 7.44 (m, 1H, H-33), 7.42 (d, $^{3}J_{H11,H12}$ = 12.7 Hz, 1H, H-12), 7.40 (d, $^{3}J_{H20,H21 \text{ and } H23,H24}$ = 8.2 Hz, 2H, H-20 + H-24), 7.24 (d, $^{3}J_{H25, H26}$ = 16.5 Hz, 1H, H-26), 7.17 (d, $^{3}J_{H25, H26}$ = 16.5 Hz, 1H, H-25), 7.15 (d, $^{3}J_{H11,H12}$ = 12.8 Hz, 1H, H-11), 7.13 (s, 1H, H-8), 6.51 (s, 1H, H-4), 6.45 (d, $^{3}J_{H7,NH}$ = 6.4 Hz, 1H, 7-NH), 5.32 (m, 2H, H-18), 4.53 (dt, $^{3}J_{H6b, H7}$ = 12.7 Hz, $^{3}J_{H6a, H7}$ = 6.4 Hz, $^{3}J_{H7,NH}$ = 6.4 Hz, 1H, H-7), 3.92 (s, 3H, H-16), 3.85 (s, 3H, H-17), 3.66 (s, 3H, H-15), 2.43 (dd, ^{2}J = 13.1 Hz, $^{3}J_{H5b,H6a}$ = 6.2 Hz, 1H, H-5b), 2.28 (td, ^{2}J = 13.0 Hz, $^{3}J_{H5a, H6a}$ = 13.0 Hz, $^{3}J_{H5a, H6b}$ = 7.2 Hz, 1H, H-5a), 2.18 (tt, ^{2}J = 13.1 Hz, $^{3}J_{H5a,H6a}$ = 13.1 Hz, $^{3}J_{H5b,H6a}$ = 6.3 Hz, $^{3}J_{H6a, H7}$ = 6.3 Hz, 1H, H-6a), 1.88 (m, 1H, H-6b), 1.84 (s, 3H, H-14), ^{13}C NMR (126 MHz, $CDCl_3$, T = 298 K) δ 179.7 (C-10), 169.9 (C-13), 163.1 (C-9), 153.8 (C-3), 151.1 (C-1), 144.1 (C-7a), 141.8 (C-2), 141.3 (C-12), 137.5 (C-22), 135.3 (C-12a + C-19), 134.8 (C-4a), 134.6 (C-27), 134.5 (C-11), 133.8 (C-30), 133.2 (C-35), 129.5 (C-26), 128.5 (C-25), 128.3 (C-29), 128.1 (C-34), 127.8 (C-31), 127.5 (C-20 + C-24), 127.0 (C-21 + C-23 + C-36), 126.5 (C-32), 126.2 (C-33), 125.9 (C-1a), 123.5 (C28), 113.1 (C-8), 107.6 (C-4), 71.0 (C-18), 61.6 (C-15), 61.5 (C-16), 56.2 (C-17), 52.8 (C-7), 38.5 (C-6), 30.0 (C-5), 22.9 (C-14).

(*S*,*E*)-*N*-(1,2,3-trimethoxy-10-oxo-9-((4-(2-(pyridin-2-yl)vinyl)benzyl)oxy)-5,6,7,10-tetrahydrobenzo[*a*]heptalen-7-yl)acetamide (compound **7b**). Derivative **3b** 60 mg (0.12 mmol) was dissolved in a 1 mL DMF and 2-vinylpyridine 25 mg (0.24 mmol) was added. Then 1.42 mg (0.006 mmol) of $Pd(OCOCH_3)_2$ and 49.7 mg (0.36 mmol) of K_2CO_3 was

added. The solution was stirred at 90 ºC for a 4 h after this time, the reaction mixture was extracted in the solvent system EtOAc:H_2O. The organic layer was evaporated and the synthesized derivative **7b** was purified by column chromatography with silica gel with dichloromethane/acetone (from 4:1 to 1:1) as an eluent. The product was obtained as a cream-colored powder. (21.2 mg, 38%), HPLC Rt = 8.398, Anal. Calcd $C_{35}H_{34}N_2O_6$: C, 72.65; H, 5.92; N, 4.84. Found C, 72.64; H, 5.95; N, 4.81; HRMS (ESI-TOF) m/z: $[M + H]^+$ Calcd for $C_{35}H_{35}N_2O_6^+$ 579.2490; Found 579.2493. ^1H NMR (500 MHz, $CDCl_3$, T = 298 K) δ 8.56 (ddd, $^3J_{H30, H31}$ = 4.8 Hz, $^4J_{H29, H31}$ = 1.8 Hz, $^4J_{H28, H31}$ = 0.8 Hz, 1H, H-31), 7.63 (dt, $^3J_{H28, H29}$ = 7.7 Hz, $^3J_{H29, H30}$ = 7.7 Hz, $^4J_{H29, H31}$ = 1.9 Hz, 1H, H-29), 7.56 (d, $^3J_{H25, H26}$ = 16.1 Hz, 1H, H-26), 7.51 (d, $^3J_{H20,H21 \text{ and } H23,H24}$ = 8.2 Hz, 2H, H-21 + H-23), 7.42 (d, $^3J_{H11,H12}$ = 12.7 Hz, 1H, H-12), 7.36 (d, $^3J_{H20,H21 \text{ and } H23,H24}$ = 8.2 Hz, 2H, H-20 + H-24), 7.36 (m, 1H, H-28), 7.17 (s, 1H, H-8), 7.14 (d, $^3J_{H11,H12}$ = 12.6 Hz, 1H, H-11), 7.12 (m, 1H, H-30),7.11 (d, $^3J_{H25, H26}$ = 16.1 Hz, 1H, H-25), 6.94 (d, $^3J_{H7,NH}$ = 6.3 Hz, 1H, 7-NH), 6.50 (s, 1H, H-4), 5.27 (m, 2H, H-18), 4.51 (dt, $^3J_{H6b, H7}$ = 12.7 Hz, $^3J_{H6a, H7}$ = 6.4 Hz, $^3J_{H7,NH}$ = 6.4 Hz, 1H, H-7), 3.89 (s, 3H, H-16), 3.84 (s, 3H, H-17), 3.63 (s, 3H, H-15), 2.45 (dd, ^2J = 13.4 Hz, $^3J_{H5b,H6a}$ = 6.4 Hz, 1H, H-5b), 2.24 (td, ^2J = 13.1 Hz, $^3J_{H5a, H6a}$ = 13.1 Hz, $^3J_{H5a, H6b}$ = 7.2 Hz, 1H, H-5a), 2.14 (m, 1H, H-6a), 1.90 (td, ^2J = 12.4 Hz, $^3J_{H6a,H7}$ = 12.4 Hz, $^3J_{H5b,H6a}$ = 7.2 Hz, 1H, H-6b), 1.80 (s, 3H, H-14), ^{13}C NMR (126 MHz, $CDCl_3$, T = 298 K) δ 179.6 (C-10), 170.0 (C-13), 163.0 (C-9), 155.5(C-27), 153.7 (C-3), 151.0 (C-1), 149.7 (C-31), 144.4 (C-7a), 141.6 (C-2), 141.4 (C-12), 136.7 (C-22 + C-29), 136.0 (C-19), 135.3 (C-12a), 134.9 (C-4a), 134.3 (C-11), 132.0 (C-26), 128.5 (C-25), 127.5 (C-20 +C-21 + C-23 + C-24), 125.9 (C-1a), 122.4 (C28), 122.3 (C-30), 113.1 (C-8), 107.6 (C-4), 70.8 (C-18), 61.6 (C-15), 61.5 (C-16), 56.2 (C-17), 52.7 (C-7), 38.3 (C-6), 30.0 (C-5), 22.8 (C-14).

(*S*,*E*)-*N*-(1,2,3-trimethoxy-10-oxo-9-((4-(2-(quinolin-3-yl)vinyl)benzyl)oxy)-5,6,7,10-tetrahydrobenzo[*a*]heptalen-7-yl)acetamide (compound **7c**). Derivative **3j** 50 mg (0.1 mmol) was dissolved in a 1 mL DMF and 3-bromoquinoline 41.6 mg (0.2 mmol) was added. Then 1.18 mg (0.005 mmol) of $Pd(OCOCH_3)_2$ and 41.4 mg (0.3 mmol) of K_2CO_3 was added. The solution was stirred at 90 ºC for a 24 h after this time, the reaction mixture was extracted in the solvent system EtOAc:H_2O. The organic layer was evaporated and the synthesized derivative **7c** was purified by column chromatography with silica gel with dichloromethane/acetone (from 4:1 to 1:1) as an eluent. The product was obtained as a cream-colored powder. (22.5 mg, 36%), HPLC Rt = 9.931, Anal. Calcd $C_{39}H_{36}N_2O_6$: C, 74.50; H, 5.77; N, 4.46; N, 2.33. Found C, 74.47; H, 5.79; N, 4.43; HRMS (ESI-TOF) m/z: $[M + H]^+$ Calcd for $C_{39}H_{37}N_2O_6^+$ 629.2646; Found 629.2644. ^1H NMR (500 MHz, $CDCl_3$, T = 298 K) δ 9.06 (d, $^4J_{H28, H35}$ = 2.3 Hz, 1H, H-35), 8.11 (d, $^4J_{H28, H35}$ = 2.5 Hz, 1H, H-28), 8.04 (d, $^3J_{H32-H33}$=8.4 Hz, 1H, H-33), 7.65 (ddd, $^3J_{H32,H33}$=8.4 Hz, $^3J_{H31,H32}$=6.9 Hz, $^4J_{H30,H32}$=1.5 Hz, 1H, H-32), 7.50 (d, $^3J_{H20,H21 \text{ and } H23,H24}$ = 8.2 Hz, 2H, H-21 + H-23), 7.42 (d, $^3J_{H11,H12}$ = 12.8 Hz, 1H, H-12), 7.40 (d, $^3J_{H20,H21 \text{ and } H23,H24}$ = 8.2 Hz, 2H, H-20 + H-24), 7.24 (d, $^3J_{H25, H26}$ = 16.3 Hz, 1H, H-25), 7.17 (d, $^3J_{H11,H12}$ = 12.9 Hz, 1H, H-11), 7.17 (s, 1H, H-8), 7.16 (d, $^3J_{H25, H26}$ = 16.6 Hz, 1H, H-26), 6.78 (d, $^3J_{H7,NH}$ = 6.4 Hz, 1H, 7-NH), 6.52 (s, 1H, H-4), 5.30 (s, 2H, H-18), 4.53 (dt, $^3J_{H6b, H7}$ = 12.4 Hz, $^3J_{H6a, H7}$ = 6.2 Hz, $^3J_{H7,NH}$ = 6.2 Hz, 1H, H-7), 3.91 (s, 3H, H-16), 3.85 (s, 3H, H-17), 3.65 (s, 3H, H-15), 2.44 (dd, ^2J = 13.0 Hz, $^3J_{H5b,H6a}$ = 6.3 Hz, 1H, H-5b), 2.28 (td, ^2J = 13.0 Hz, $^3J_{H5a, H6a}$ = 13.0 Hz, $^3J_{H5a, H6b}$ = 7.1 Hz, 1H, H-5a), 2.20 (tt, ^2J = 13.1,

$^{3}J_{H5a,H6a}$ = 13.1 Hz, $^{3}J_{H5b,H6a}$ = 6.5 Hz, $^{3}J_{H6a, H7}$ = 6.5 Hz, 1H, H-6a), 1.89 (m, 1H, H-6b), 1.85 (s, 3H, H-14), ^{13}C NMR (126 MHz, $CDCl_3$, T = 298 K) δ 179.7 (C-10), 169.9 (C-13), 163.0 (C-9), 153.8 (C-3), 151.1 (C-1), 149.3 (C-35), 147.5 (C-34), 144.2 (C-7a), 141.7 (C-2), 141.4 (C-12), 136.8 (C-22), 135.9 (C-19), 135.4 (C-12a), 134.8 (C-4a), 134.5 (C-11), 132.7 (C28), 130.13 (C-25), 130.11 (C-29), 129.5 (C-32), 129.2 (C-33), 128.2 (C-27), 128.0 (C-30), 127.6 (C-20 + C-24), 127.2 (C-31), 127.1 (C-21 + C-23), 125.9 (C-1a), 125.8 (C-26), 113.1 (C-8), 107.6 (C-4), 70.8 (C-18), 61.6 (C-15), 61.5 (C-16), 56.2 (C-17), 52.8 (C-7), 38.4 (C-6), 30.0 (C-5), 22.9 (C-14).

(4*E*,6*Z*,8*S*,9*S*,10*E*,12*S*,13*R*,14*S*,16*R*)-19-((4-((*E*)-4-((((*S*)-7-acetamido-1,2,3-trimethoxy-10-oxo-5,6,7,10-tetrahydrobenzo[*a*]heptalen-9-yl)oxy)methyl)styryl)benzyl)amino)-13-hydroxy-8,14-dimethoxy-4,10,12,16-tetramethyl-3,20,22-trioxo-2-azabicyclo[16.3.1]docosa-1(21),4,6,10,18-pentaen-9-yl carbamate (compound **7d**). Derivative **3j** 30 mg (0.06 mmol) was dissolved in a 0.5 mL DMF and **10** 30 mg (0.04 mmol) was added. Then 0.71 mg (0.003 mmol) of $Pd(OCOCH_3)_2$ and 50 µL of TEA was added. The solution was stirred at room temperature for a week and after this time, ethyl acetate was added and extracted twice with 25 mL of water. The organic layer was evaporated and the synthesized derivative **7d** was purified by column chromatography with silica gel with dichloromethane/methanol (75:1) as an eluent. The product was obtained as a violet powder. (26 mg, 59%), HPLC R_t= 8.115 min. Anal. Calcd for $C_{65}H_{74}N_4O_{14}$: C, 68.77; H, 6.57; N, 4.93. Found: C, 68.76; H, 6.58; N, 4.93; HRMS (ESI-TOF) m/z: $[M + H]^+$ Calcd 1134.5202; Found 1134.5204. FT-IR (KBr): ν(N-H)*$_{amide, substituent,}$ ν(O-H)* - 3480cm^{-1};; $ν_s$(N-H)$_{lactam}$ - 3320cm^{-1}; ν(=CH) - 3191 cm^{-1}; ν(C-H) - 2931 cm^{-1} ; ν(C=O)$_{carbamate}$ - 1722 cm^{-1}; ν(C=O)* $_{lactam, amide}$ - 1685 cm^{-1}; ν(C=O)$_{quinone}$ - 1651cm^{-1}; ν(C=C) - 1610 cm^{-1}; δ(N-H)*$_{lactam,amide}$ - 1562 cm^{-1}; δ(N-H)$_{carbamate}$ - 1487 cm^{-1}; ν(C-N)$_{carbamate}$ - 1321 cm^{-1}; ν(C-O)$_{as}$ - 1264 cm^{-1}; ν(C- O-C)$_{carbamate}$ - 1193 cm^{-1}; ν(C-O)$_s$ - 1139, 1096 cm^{-1} , ν(C-O)$_{methoxy}$ - 1047 cm^{-1}. ^{1}H NMR (500 MHz, $CDCl_3$, 25ºC) δ 9.15 (s, 1H, NH-1’), 7.56 – 7.46 (m, 3H, H-20,24, NH-7), 7.44 – 7.38 (m, 3H, H-12,32’,34’), 7.37 – 7.32 (m, 1H, H-26), 7.30 (s, 1H, H-19’), 7.28 – 7.24 (m, 3H, H-21,23,25), 7.22 – 7.14 (m, 1H, H-11), 7.10 (d, $^{3}J_{H31'/H35',H32'/34'}$ = 8.0 Hz, 2H, H-31’,35’), 7.00 (s, 1H, H-8), 6.95 (d, $^{3}J_{H3',H4'}$ = 11.4 Hz, 1H, H-3’), 6.58 (t, $^{3}J_{H3',H4'}$ = 11.4 Hz, 1H, H-4’), 6.53 (s, 1H, H-4), 6.50 – 6.42 (m, 1H, NH-17’), 5.92 – 5.85 (m, 1H, H-5’), 5.84 – 5.75 (m, 1H, H-9’), 5.32 (d, ^{2}J= 7.5 Hz, 2H, H-18), 5.18 (s, 1H, H-7’), 4.90 (s, 2H, NH_2-24’), 4.80 – 4.72 (m, 1H, H-29’), 4.67 – 4.58 (m, 1H, H-29’), 4.55 – 4.44 (m, 1H, H-7), 4.34 – 4.28 (m, 1H, H-6’), 4.18 (bs, 1H, OH-11’), 3.93 (s, 3H, H-16), 3.88 (s, 3H, H-17), 3.67 (s, 3H, H-15), 3.61 – 3.55 (m, 1H, H-11’), 3.47 – 3.42 (m, 1H, H-12’), 3.36 (s, 3H, H-27’), 3.27 (s, 3H, H-23’), 2.78 – 2.71 (m, 1H, H-10’), 2.67 (d, ^{2}J =13.8 Hz, 1H, H-15’), 2.51 – 2.40 (m, 2H, H-5b,15’), 2.34 – 2.27 (m, 1H, H-5a), 2.27 – 2.17 (m, 1H, H-6a), 2.03 (s, 3H, H-22’), 1.83 (s, 3H, H-14), 1.80 (s, 4H, H-6b,25’), 1.78 (s, 3H, H-13’,14’), 1.03 (d, $^{3}J_{H10,H26}$= 6.4 Hz, 3H, H-26’), 1.00 (d, $^{3}J_{H14,H28}$= 6.8 Hz, 3H, H-28’).^{13}C NMR (126 MHz, $CDCl_3$, 25ºC) δ 183.9 (C-18’), 181.2 (C-21’), 179.8 (C-10), 169.7 (C-13), 168.5 (C-1’), 163.1 (C-9), 156.2 (C-24’), 153.8 (C-3), 151.2 (C-1), 144.8 (C-17’), 143.4 (C-7a), 141.8 (C-2), 141.4 (C-20’), 141.1 (C-12), 137.2 (C-33’), 136.3 (C-5’), 136.0 (C-30’), 135.3* (C-12a,19), 135.1* (C-22, 2’), 134.8 (C-4a), 134.7 (C-11), 133.9 (C-9’), 133.0 (C-8’), 128.8 (C-26), 128.5 (C-25), 128.3* (C-31’,35’), 127.4* (C-20,24), 127.3* (C-32’,34’), 127.1 (C-3’), 127.0 (C-21,23), 126.7 (C-4’), 125.9 (C-1a), 113.1 (C-8), 109.2 (C-16’), 109.0

(C-19’), 107.6 (C-4), 81.8 (C-7’), 81.6 (C-6’), 81.3 (C-12’), 72.8 (C-11’), 71.0 (C-18), 61.6 (C-15), 61.6 (C-16), 57.3 (C-23’), 56.9 (C-27’), 56.3 (C-17), 52.8 (C-7), 50.0 (C-29’), 38.6 (C-6), 35.2 (C-13’), 34.6 (C-15’), 32.5 (C-10’), 30.0 (C-5), 28.7 (C-14’), 23.1(C-28’), 23.0 (C-14), 12.9 (C-25’), 12.8 (C-22’), 12.5 (C-26’). (*-overlapped)

(*S*,*E*)-*N*-(1,2,3-trimethoxy-10-((4-(2-(naphthalen-2-yl)vinyl)benzyl)oxy)-9-oxo-5,6,7,9-tetrahydrobenzo[*a*]heptalen-7-yl)acetamide (compound **8a**). Derivative **4b** 60 mg (0.12 mmol) was dissolved in a 1 mL DMF and 2-vinylpyridine 25 mg (0.24 mmol) was added. Then 1.42 mg (0.006 mmol) of $Pd(OCOCH_3)_2$ and 49.7 mg (0.36 mmol) of K_2CO_3 was added. The solution was stirred at 90 ºC for a 4h after this time, the reaction mixture was extracted in the solvent system EtOAc:H_2O. The organic layer was evaporated and the synthesized derivative **8a** was purified by column chromatography with silica gel with dichloromethane/acetone (from 4:1 to 1:1) as an eluent. The product was obtained as a cream-colored powder. (23.4 mg, 39%), HPLC Rt = 14.338, Anal. Calcd $C_{40}H_{37}NO_6$: C, 76.53; H, 5.94; N, 2.23. Found C, 76.52; H, 5.96; N, 2.24; HRMS (ESI-TOF) m/z: $[M + Na]^+$ Calcd for $C_{40}H_{37}NNaO_6^+$ 650.2519; Found 650.2517. ^{1}H NMR (500 MHz, $CDCl_3$, T = 298 K) δ 7.85 (d, $^4J_{H28, H36}$ = 2.0 Hz, 1H, H-36), 7.83 (m, 1H, H-31), 7.82 (d, $^3J_{H28, H29}$ = 8.8 Hz, 1H, H-29), 7.80 (m, 1H, H-34), 7.73 (dd, $^3J_{H28, H29}$ = 8.6 Hz, $^4J_{H28, H36}$ = 1.7 Hz, 1H, H-28), 7.57 (s, 1H, H-8), 7.57 (d, $^3J_{H20,H21 \text{ and } H23,H24}$ = 8.3 Hz, 2H, H-21 + H-23), 7.52 (d, $^3J_{H7,NH}$ = 6.8 Hz, 1H, 7-NH), 7.47 (m, 1H, H-32), 7.45 (m, 3H, H-20 + H-24 +H-33), 7.28 (d, $^3J_{H25, H26}$ = 16.3 Hz, 1H, H-26), 7.23 (d, $^3J_{H11,H12}$ = 10.8 Hz, 1H, H-12), 7.21 (d, $^3J_{H25, H26}$ = 16.3 Hz, 1H, H-25), 6.91 (d, $^3J_{H11,H12}$ = 10.9 Hz, 1H, H-11), 6.51 (s, 1H, H-4), 5.37 (d, ^2J = 13.0 Hz, 1H, H-18a), 5.29 (d, ^2J = 13.0 Hz, 1H, H-18b), 4.65 (dt, $^3J_{H6b,H7}$ = 13.0 Hz, $^3J_{H6a,H7}$ = 6.6 Hz, $^3J_{H7,NH}$ = 6.6 Hz, 1H, H-7), 3.92 (s, 3H, H-16), 3.87 (s, 3H, H-17), 3.63 (s, 3H, H-15), 2.50 (dd, ^2J = 13.3 Hz, $^3J_{H5b,H6a}$ = 6.0 Hz, 1H, H-5b), 2.38 (td, ^2J = 13.1 Hz, $^3J_{H5a,H6a}$ = 13.1 Hz, $^3J_{H5a,H6b}$ = 6.6 Hz, 1H, H-5a), 2.29 (tt, ^2J = 12.8 Hz, $^3J_{H5a,H6a}$ = 12.8 Hz, $^3J_{H5b,H6a}$ = 6.3 Hz, $^3J_{H6a,H7}$ = 6.3 Hz, 1H, H-6a), 2.00 (s, 3H, H-14), 1.89 (td, ^2J = 11.7 Hz, $^3J_{H6a,H7}$ = 11.7 Hz, $^3J_{H5b,H6a}$ = 5.9 Hz, 1H, H-6b), ^{13}C NMR (126 MHz, $CDCl_3$, T = 298 K) δ 179.8 (C-9), 170.1 (C-13), 163.1 (C-10), 153.6 (C-3), 151.9 (C-7a), 151.3 (C-1), 141.8 (C-2), 137.6 (C-22), 137.2 (C-12a), 135.3 (C-19), 134.8 (C-12), 134.3 (C-4a), 134.7 (C-27), 133.8 (C-30), 133.2 (C-35), 131.1 (C-8), 129.4 (C-26), 128.5 (C-25 +C-29), 128.1 (C-34), 127.8 (C-31), 127.7 (C-20 + C-24), 127.0 (C-21 + C-23), 129.9 (C-36), 126.5 (C-32), 126.1 (C-33), 125.7 (C-1a), 123.6 (C-28), 115.1 (C-11), 107.5 (C-4), 71.0 (C-18), 61.7 (C-15), 61.5 (C-16), 56.2 (C-17), 52.6 (C-7), 36.7 (C-6), 30.0 (C-5), 23.1(C-14).

(*S*,*E*)-*N*-(1,2,3-trimethoxy-9-oxo-10-((4-(2-(pyridin-2-yl)vinyl)benzyl)oxy)-5,6,7,9-tetrahydrobenzo[*a*]heptalen-7-yl)acetamide (compound **8b**). Derivative **4b** 60 mg (0.12 mmol) was dissolved in a 1 mL DMF and 2-vinylpyridine 25 mg (0.24 mmol) was added. Then 1.42 mg (0.006 mmol) of $Pd(OCOCH_3)_2$ and 49.7 mg (0.36 mmol) of K_2CO_3 was added. The solution was stirred at 90 ºC for a 4h after this time, the reaction mixture was extracted in the solvent system EtOAc:H_2O. The organic layer was evaporated and the synthesized derivative **8b** was purified by column chromatography with silica gel with dichloromethane/acetone (from 4:1 to 1:1) as an eluent. The product was obtained as a cream-colored powder. (19.5 mg, 35%), HPLC Rt = 8.931, Anal. Calcd $C_{35}H_{34}N_2O_6$: C, 72.65; H,

5.92; N, 4.84. Found C, 72.68; H, 5.92; N, 4.80; HRMS (ESI-TOF) m/z: $[M + H]^+$ Calcd for $C_{35}H_{35}N_2O_6^+$ 579.2490; Found 579.2488. ^1H NMR (500 MHz, $CDCl_3$, T = 298 K) δ 8.58 (ddd, $^3J_{H30,H31}$ = 4.8 Hz, $^4J_{H29,H31}$ = 1.8 Hz, $^4J_{H28,H31}$ = 0.9 Hz, 1H, H-31), 7.90 (d, $^3J_{H7,NH}$ = 6.5 Hz, 1H, 7-NH), 7.64 (dt, $^3J_{H28,H29}$ = 7.5 Hz, $^3J_{H29,H30}$ = 7.5 Hz, $^4J_{H29,H31}$ = 1.8 Hz, 1H, H-29), 7.61 (d, $^3J_{H25, H26}$ = 16.0 Hz, 1H, H-26), 7.59 (s, 1H, H-8), 7.58 (d, $^3J_{H20,H21 \text{ and } H23,H24}$ = 8.4 Hz, 2H, H-21 + H-23), 7.43 (d, $^3J_{H20,H21 \text{ and } H23,H24}$ = 8.4 Hz, 2H, H-20 + H-24), 7.36 (dt, $^3J_{H28, H29}$ = 8.0 Hz, $^3J_{H28, H30}$ = 1.1 Hz, $^4J_{H28, H31}$ = 1.1 Hz 1H, H-28), 7.21 (d, $^3J_{H11,H12}$ = 10.7 Hz, 1H, H-12), 7.16 (d, $^3J_{H25, H26}$ = 16.1 Hz, 1H, H-25), 7.13 (ddd, $^3J_{H29,H30}$ = 7.5 Hz, $^3J_{H30,H31}$ = 4.8 Hz, $^4J_{H28,H30}$ = 1.2 Hz, 1H, H-30), 6.90 (d, $^3J_{H11,H12}$ = 10.9 Hz, 1H, H-11), 6.50 (s, 1H, H-4), 5.36 (d, ^2J = 13.1 Hz, 1H, H-18a), 5.28 (d, ^2J = 13.0 Hz, 1H, H-18b), 4.63 (dt, $^3J_{H6b, H7}$ = 12.2 Hz, $^3J_{H6a, H7}$ = 6.4 Hz, $^3J_{H7,NH}$ = 6.4 Hz, 1H, H-7), 3.90 (s, 3H, H-16), 3.86 (s, 3H, H-17), 3.61 (s, 3H, H-15), 2.48 (dd, ^2J = 13.3 Hz, $^3J_{H5b,H6a}$ = 6.5 Hz, 1H, H-5b), 2.36 (td, ^2J = 12.9 Hz, $^3J_{H5a,H6a}$ = 12.9 Hz, $^3J_{H5a,H6b}$ = 6.3 Hz, 1H, H-5a), 2.28 (tt, ^2J = 12.7 Hz, $^3J_{H5a,H6a}$ = 12.7 Hz, $^3J_{H5b,H6a}$ = 6.1 Hz, $^3J_{H6a,H7}$ = 6.1 Hz, 1H, H-6a), 1.98 (s, 3H, H-14), 1.90 (td, ^2J = 11.7 Hz, $^3J_{H6a,H7}$ = 11.7 Hz, $^3J_{H5b,H6a}$ = 5.7 Hz, 1H, H-6b), ^{13}C NMR (126 MHz, $CDCl_3$, T = 298 K) δ 179.7 (C-9), 170.1 (C-13), 163.0 (C-10), 155.5(C-27), 153.6 (C-3), 152.1 (C-7a), 151.2 (C-1), 149.8 (C-31), 141.7 (C-2), 137.3 (C-12a), 136.8 (C-22), 136.7 (C-29), 135.4 (C-19), 135.3 (C-12), 134.3 (C-4a), 132.1 (C-26), 131.8 (C-8), 128.5 (C-25), 127.6 (C-20 +C-21 + C-23 + C-24), 125.7 (C-1a), 122.3 (C28 + C-30), 115.1 (C-11), 107.4 (C-4), 70.9 (C-18), 61.6 (C-15), 61.5 (C-16), 56.2 (C-17), 52.6 (C-7), 36.5 (C-6), 30.0 (C-5), 22.9 (C-14).

(*S*,*E*)-*N*-(1,2,3-trimethoxy-9-oxo-10-((4-(2-(quinolin-3-yl)vinyl)benzyl)oxy)-5,6,7,9-tetrahydrobenzo[*a*]heptalen-7-yl)acetamide (compound **8c**). Derivative **4j** 50 mg (0.1 mmol) was dissolved in a 1 mL DMF and 3-bromoquinoline 41.6 mg (0.2 mmol) was added. Then 1.18 mg (0.005 mmol) of $Pd(OCOCH_3)_2$ and 41.4 mg (0.3 mmol) of K_2CO_3 was added. The solution was stirred at 90 ºC for a 24 h after this time, the reaction mixture was extracted in the solvent system EtOAc:H_2O. The organic layer was evaporated and the synthesized derivative **8c** was purified by column chromatography with silica gel with dichloromethane/acetone (from 4:1 to 1:1) as an eluent. The product was obtained as a cream-colored powder. (20.6 mg, 33%), HPLC Rt = 10.925, Anal. Calcd $C_{39}H_{36}N_2O_6$: C, 74.50; H, 5.77; N, 4.46; N, 2.33. Found C, 74.52; H, 5.74; N, 4.47; HRMS (ESI-TOF) m/z: $[M + H]^+$ Calcd for $C_{39}H_{37}N_2O_6^+$ 629.2646; Found 629.2643. ^1H NMR (500 MHz, $CDCl_3$, T = 298 K) δ 9.11 (d, $^4J_{H28,H35}$ = 2.3 Hz, 1H, H-35), 8.17 (d, $^4J_{H28, H35}$ = 2.5 Hz, 1H, H-28), 8.08 (d, $^3J_{H32,H33}$=8.4 Hz, 1H, H-33), 7.65 (ddd, $^3J_{H32,H33}$=8.4 Hz, $^3J_{H31,H32}$=6.9 Hz, $^4J_{H30,H32}$=1.5 Hz, 1H, H-32), 7.59 (d, $^3J_{H20,H21 \text{ and } H23,H24}$ = 8.3 Hz, 2H, H-21 + H-23), 7.54 (s, 1H, H-8), 7.47 (d, $^3J_{H20,H21 \text{ and } H23,H24}$ = 8.3 Hz, 2H, H-20 + H-24), 7.33(d, $^3J_{H7,NH}$ = 6.0 Hz, 1H, 7-NH), 7.32 (d, $^3J_{H25,H26}$ = 16.4 Hz, 1H, H-25), 7.24 (d, $^3J_{H25,H26}$ = 16.5 Hz, 1H, H-26), 7.22 (d, $^3J_{H11,H12}$ = 10.8 Hz, 1H, H-12), 6.90 (d, $^3J_{H11,H12}$ = 11.0 Hz, 1H, H-11), 6.51 (s, 1H, H-4), 5.37 (d, ^2J = 13.0 Hz, 1H, H-18a), 5.30 (d, ^2J = 13.0 Hz, 1H, H-18b), 4.64 (dt, $^3J_{H6b,H7}$ = 13.0 Hz, $^3J_{H6a,H7}$ = 6.6 Hz, $^3J_{H7,NH}$ = 6.6 Hz, 1H, H-7), 3.91 (s, 3H, H-16), 3.88 (s, 3H, H-17), 3.63 (s, 3H, H-15), 2.48 (dd, ^2J = 13.4 Hz, $^3J_{H5b,H6a}$ = 6.3 Hz, 1H, H-5b), 2.39 (td, ^2J = 13.2 Hz, $^3J_{H5a,H6a}$ = 13.2 Hz, $^3J_{H5a,H6b}$ = 6.3 Hz, 1H, H-5a), 2.27 (tt, ^2J = 13.2 Hz, $^3J_{H5a,H6a}$ = 13.2 Hz, $^3J_{H5b,H6a}$ = 6.5 Hz, $^3J_{H6a,H7}$ = 6.5 Hz, 1H, H-6a), 1.99 (s, 3H, H-14), 1.87 (m, 1H, H-6b), ^{13}C NMR (126 MHz, $CDCl_3$, T = 298 K) δ 179.8 (C-9), 170.0 (C-13), 163.1 (C-10), 153.8 (C-3), 151.7 (C-7a),

151.3 (C-1), 149.5 (C-35), 147.6 (C-34), 141.8 (C-2), 137.2 (C-12a), 137.0 (C-22), 135.4 (C-19), 135.2 (C-12), 134.3 (C-4a), 132.6 (C28), 131.2 (C-8), 130.4 (C-25), 130.3 (C-29), 129.4 (C-32), 129.3 (C-33), 128.2 (C-27), 128.0 (C-30), 127.8 (C-20 + C-24), 127.2 (C-21 + C-23 + C-31), 125.9 (C-26), 125.7 (C-1a), 115.0 (C-11), 107.5 (C-4), 70.9 (C-18), 61.7 (C-15), 61.5 (C-16), 56.2 (C-17), 52.5 (C-7), 36.8 (C-6), 30.0 (C-5), 23.1 (C-14).

(4*E*,6*Z*,8*S*,9*S*,10*E*,12*S*,13*R*,14*S*,16*R*)-19-((4-((*E*)-4-((((*S*)-7-acetamido-1,2,3-trimethoxy-9-oxo-5,6,7,9-tetrahydrobenzo[*a*]heptalen-10-yl)oxy)methyl)styryl)benzyl)amino)-13-hydroxy-8,14-dimethoxy-4,10,12,16-tetramethyl-3,20,22-trioxo-2-azabicyclo[16.3.1]docosa-1(21),4,6,10,18-pentaen-9-yl carbamate (compound **8d**). Derivative **4j** 50 mg (0.1 mmol) was dissolved in a 0.5 mL DMF and **10** 50 mg (0.07 mmol) was added. Then 1.2 mg (0.005 mmol) of $Pd(OCOCH_3)_2$ and 50 μL of TEA was added. The solution was stirred at room temperature for a week and after this time, ethyl acetate was added and extracted twice with 25 mL of water. The organic layer was evaporated and the synthesized derivative **8d** was purified by column chromatography with silica gel with dichloromethane/methanol (60:1) as an eluent. The product was obtained as a violet powder. (19 mg, 25%), HPLC R_t= 9.128 min. Anal. Calcd for $C_{65}H_{74}N_4O_{14}$: C, 68.77; H, 6.57; N, 4.93. Found: C, 68.76; H, 6.55; N, 4.91; HRMS (ESI-TOF) m/z: $[M + H]^+$ Calcd 1134.5202; Found 1134.5200. FT-IR (KBr): ν(N-H)*$_{amide,\ substituent,}$ ν(O-H)* - 3474 cm^{-1}; $ν_s$(N-H)$_{lactam}$ - 3324cm^{-1}; ν(N-H)*$_{amide}$, ν(O-H),ν(=CH)* - 3197 cm^{-1}; ν(C-H) - 2931 cm^{-1} ; ν(C=O)$_{carbamate}$ - 1722 cm^{-1}; ν(C=O)* $_{lactam,\ amide}$ - 1689 cm^{-1}; ν(C=O)$_{quinone}$ - 1651 cm^{-1}; ν(C=C) - 1613 cm^{-1}; δ(N-H)*$_{lactam,amide}$ - 1573 cm^{-1}; δ(N-H)$_{carbamate}$ - 1486 cm^{-1}; ν(C-N)$_{carbamate}$ - 1322 cm^{-1}; ν(C-O)$_{as}$ - 1236 cm^{-1}; ν(C- O-C)$_{carbamate}$ - 1194 cm^{-1}; ν(C-O)$_s$ - 1139, 1095 cm^{-1} , ν(C-O)$_{methoxy}$ - 1049 cm^{-1}. ^{1}H NMR (500 MHz, $CDCl_3$, 25ºC) δ 9.12 (s, 1H, NH-1'), 7.50 – 7.48 (m, 2H, H-20,24), 7.48 – 7.46 (m, 4H, H-31',32',34',35'), 7.41 – 7.37 (m, 2H, H-8,26), 7.23 – 7.20 (m, 3H, H-12,21,23), 7.16 (d, $^3J_{H11,H12}$ = 10.7 Hz, 1H, H-11), 7.06 (s, 2H, H-25,19'), 7.04 – 7.00 (m, 1H, NH-7), 6.92 (d, $^3J_{H3',H4'}$ = 11.5 Hz, 1H, H-3'), 6.54 (t, $^3J_{H3',H4'}$ = 11.3 Hz, 1H, H-4'), 6.46 (s, 1H, H-4), 6.42 (t, $^3J_{NH17',H29'}$ = = 5.5 Hz, 1H, NH-17'), 5.85 (t, $^3J_{H5',H6'}$ = 9.2 Hz, 1H, H-5'), 5.81 (d, $^3J_{H9',H10'}$ = 10.6 Hz, 1H, H-9'), 5.30 (d, ^2J = 13.0 Hz, 1H, H-18), 5.23 (d, ^2J = 13.4 Hz, 1H, H-18), 5.14 (s, 1H, H-7'), 4.87 (vbs, 2H, NH_2-24'), 4.74 – 4.68 (m, 1H, H-29'), 4.62 – 4.55 (m, 2H, H-7, 29'), 4.29 – 4.25 (m, 1H, H-6'), 4.13 (s, 1H, OH-11'), 3.87 (s, 3H, H-16), 3.83 (s, 3H, H-17), 3.57 (s, 3H, H-15), 3.55 – 3.52 (m, 1H, H-11'), 3.42 – 3.37 (m, 1H, H-12'), 3.32 (s, 3H, H-27'), 3.23 (s, 3H, H-23'), 2.74 – 2.66 (m, 1H, H-10'), 2.62 (d, ^2J =13.9 Hz, 1H, H-15'), 2.45 (dt, ^2J =12.0 Hz, $^3J_{H5b,H6a}$ = 6.0 Hz, 1H, H-5b), 2.41 – 2.37 (m, 1H, H-15'), 2.33 (^2J = 13.2 Hz, $^3J_{H5a,H6a}$ = 13.2 Hz, $^3J_{H5a,H6b}$ = 6.5 Hz, 1H, H-5a), 2.21 (dt, ^2J = 12.9 Hz, $^3J_{H5a,H6a}$ = 12.9 Hz, $^3J_{H5b,H6a}$ = 6.3 Hz, $^3J_{H6a,H7}$ = 6.3 Hz, 1H, H-6a), 1.98 (s, 3H, H-22'), 1.95 (s, 3H, H-14), 1.85 – 1.78 (m, 1H, H-6b), 1.76 (d, $^4J_{H10',H25'}$ = 1.3 Hz, 3H, H-25'), 1.74 – 1.72 (m, 1H, H-14'), 1.70 (bs, 2H, H-13'), 0.99 (d, $^3J_{H10,H26}$= 6.1 Hz, 3H, H-26'), 0.95 (d, $^3J_{H14,H28}$= 6.9 Hz, 3H, H-28').^{13}C NMR (126 MHz, $CDCl_3$, 25ºC) δ 183.9 (C-18'), 181.2 (C-21'), 179.8 (C-9), 169.9 (C-13), 168.5 (C-1'), 163.1 (C-10), 156.2 (C-24'), 153.6 (C-3), 151.6 (C-7a), 151.3 (C-1), 144.8 (C-17'), 141.4 (C-2), 141.4 (C-20'), 137.6 (C-12a), 137.3 (C-22), 137.1 (C-33'), 136.1 (C-5'), 136.0 (C-30'), 135.2* (C-12, 2'), 135.0 (C-19), 134.3 (C-4a), 133.8 (C-9'), 133.0 (C-8'), 131.2 (C-8), 129.0 (C-26), 128.4 (C-25), 128.3* (C-31',35'), 127.7* (C-20,24), 127.4* (C-32',34'), 127.1 (C-3'), 127.1 (C-21,23), 126.7 (C-4'), 125.7 (C-1a), 115.0 (C-11),

109.2 (C-16’), 109.0 (C-19’), 107.5 (C-4), 81.8 (C-7’), 81.6 (C-6’), 81.4 (C-12’), 72.8 (C-11’), 70.9 (C-18), 61.7 (C-15), 61.5 (C-16), 57.3 (C-23’), 56.9 (C-27’), 56.2 (C-17), 52.4 (C-7), 50.0 (C-29’), 36.9 (C-6), 35.2 (C-13’), 34.6 (C-15’), 32.4 (C-10’), 30.0 (C-5), 28.6 (C-14’), 23.1 (C-28’), 23.1 (C-14), 12.9 (C-25’), 12.7 (C-22’), 12.5 (C-26’). (*-overlapped)

(4*E*,6*Z*,8*S*,9*S*,10*E*,12*S*,13*R*,14*S*,16*R*)-19-((2-azidoethyl)amino)-13-hydroxy-8,14-dimethoxy-4,10,12,16-tetramethyl-3,20,22-trioxo-2-azabicyclo[16.3.1]docosa-1(21),4,6,10,18-pentaen-9-yl carbamate (compound **9**). First, 200 mg (0.36 mmol) of geldanamycin was dissolved in a 4.4 mL mixture of THF/MeOH (10:1) and then a four-fold excess of 2-azidoethanamine was added (1.44 mmol). Then, to the each mixture, 0.7 ml of TEA was added. The mixtures were stirred at 60 °C for a 8h and after that the solvent was evaporated. After the reaction, solvent was evaporated. All of these products were purified by column chromatography on silica gel with methylene chloride/acetone as an eluent. After evaporation of the solvent, product was obtained as a violet powder, (274 mg, 98%). HPLC R_t= 6.549 min. Anal. Calcd for $C_{30}H_{42}N_6O_8$: C, 58.62; H, 6.89; N, 13.67. Found: C, 58.63; H, 6.90; N, 13.65; HRMS (ESI-TOF) m/z: $[M + H]^+$ Calcd 614.3064; Found 614.3067. FT-IR (KBr): ν_{as}(N-H)$_{carbamate}$=3446.02 cm^{-1}, ν_s(N-H)$_{carbamate}$=3420.24 cm^{-1}, ν_s(N-H)$_{lactam}$=3321.45 cm^{-1}, ν(O-H) =3200.36 cm^{-1}, ν_s(C-H) =2931.03 cm^{-1}, ν(N_3) =2102.72 cm^{-1}, ν(C=O)$_{carbamate}$ =1727.88 cm^{-1} , ν(C=O)$_{lactam}$=1691.24 cm^{-1}, ν(C=O)$_{quinone}$=1650.69 cm^{-1}, ν(C=C) =1616.24 cm^{-1} , δ(N-H)*$_{lactam,substituent}$=1581.51 cm^{-1}, δ(N-H)$_{carbamate}$ =1489.14 cm^{-1}, ν(C-N)$_{carbamate}$ =1323.27 cm^{-1}, ν(C- O-C)$_{carbamate}$ =1190.92 cm^{-1}, ν(C- O-C)$_{substituent}$ =1102.09 cm^{-1}, ν(C- O-C) $_{methoxy}$ =1056.87 cm^{-1} , γ(=C-H)$_{carbamate}$ =784.50 cm^{-1} , γ(N-H)$_{carbamate}$=700.51 cm^{-1}. ^{1}H NMR (500 MHz, $CDCl_3$, 25ºC) δ 9.10 (s, 1H, NH-1), 7.29 (s, 1H, H-19), 6.94 (d, $^3J_{H3,H4}$ = 11.7 Hz, 1H, H-3), 6.61 – 6.54 (m, 1H, H-4), 6.32 (t, $^3J_{H29,NH17}$ = 5.8 Hz, 1H, NH-17), 5.88 (s, 1H, H-9), 5.86 (t, $^3J_{H5,H6}$ = 10.6 Hz, 1H, H-5), 5.18 (s, 1H, H-7), 4.88 (s, 2H, NH_2-24), 4.33 – 4.28 (m, 1H, H-6), 3.76 – 3.64 (m, 2H, H-29), 3.64 – 3.59 (m, 2H, H-30), 3.58 – 3.55 (m, 1H, H-11), 3.46 – 3.41 (m, 1H, H-12), 3.35 (s, 3H, H-27), 3.26 (s, 3H, H-23), 2.92 – 2.85 (m, 1H, OH-11), 2.77 – 2.72 (m, 1H, H-10), 2.73 – 2.67 (m, 1H, H-15), 2.29 (dd, ^2J = 14.1 Hz, $^3J_{H14,H15}$ = 10.5 Hz, 1H, H-15), 2.02 (d, $^4J_{H3,H22}$ = 1.3 Hz, 3H, H-22), 1.82 – 1.76 (m, 5H, H-13,25), 1.76 – 1.67 (m, 1H, H-14), 0.99 (d, $^3J_{H10,H26}$= 7.4 Hz, 3H, H-26), 0.97 (d, $^3J_{H14,H28}$= 6.9 Hz, 3H, H-28). ^{13}C NMR (126 MHz, $CDCl_3$, 25ºC) δ 183.9 (C-18), 181.3 (C-21), 168.5 (C-1), 156.2 (C-24), 144.8 (C-17), 141.0 (C-20), 136.0 (C-5), 135.1 (C-2), 133.7 (C-9), 133.0 (C-8), 127.1 (C-3), 126.7 (C-4), 109.8 (C-16), 109.2 (C-19), 81.7 (C-7), 81.5 (C-6), 81.3 (C-12), 72.8 (C-11), 57.3 (C-23), 56.9 (C-27), 50.5 (C-29), 44.6 (C-30), 35.2 (C-13), 34.5 (C-15), 32.5 (C-10), 28.8 (C-14), 23.1 (C-28), 13.0 (C-25), 12.7 (C-22), 12.6 (C-26). (*-overlapped)

(4*E*,6*Z*,8*S*,9*S*,10*E*,12*S*,13*R*,14*S*,16*R*)-13-hydroxy-19-((4-iodobenzyl)amino)-8,14-dimethoxy-4,10,12,16-tetramethyl-3,20,22-trioxo-2-azabicyclo[16.3.1]docosa-1(21),4,6,10,18-pentaen-9-yl carbamate (compound **10**) First, 50 mg (0.09 mmol) of geldanamycin was dissolved in a 3.3 mL mixture of THF/MeOH (10:1) and then a four-fold excess of (4-iodophenyl)methanamine was added (0.36 mmol). Then, to the each mixture, 0.5 ml of TEA was added. The mixtures were stirred at 60 °C for a 5h and after that the solvent was evaporated. After the reaction, solvent was evaporated. All of these products were purified by column chromatography on silica gel with methylene chloride/acetone as an eluent. After

evaporation of the solvent, product was obtained as a violet powder, (26 mg, 59%). HPLC R_t= 13.911 min. Anal. Calcd for $C_{35}H_{44}IN_3O_8$: C, 55.19 H, 5.82; I, 16.66; N, 5.52. Found: C, 55.20 H, 5.84; I, 16.65; N, 5.51.; HRMS (ESI-TOF) m/z: $[M + H]^+$ Calcd 761.2173; Found 761.2176. FT-IR (KBr): ν_{as}(N-H)$_{carbamate}$=3489.48 cm^{-1}, ν_s(N-H)$_{carbamate}$=3433.25 cm^{-1}, ν_s(N-H)$_{lactam}$=3323.38 cm^{-1}, ν(O-H) =3191.22 cm^{-1}, ν_s(C-H) =2929.43 cm^{-1}, ν(C=O)$_{carbamate}$ =1733.03 cm^{-1} , ν(C=O)$_{lactam}$=1695.73 cm^{-1}, ν(C=O)$_{quinone}$=1646.62 cm^{-1}, ν(C=C) =1615.53 cm^{-1} , δ(N-H)*$_{lactam,substituent}$=1590.95 cm^{-1}, δ(N-H)$_{carbamate}$ =1485.53 cm^{-1}, ν(C-N)$_{carbamate}$ =1326.30 cm^{-1}, ν(C- O-C)$_{carbamate}$ =1190.30 cm^{-1}, ν(C- O-C)$_{substituent}$ =1100.30 cm^{-1}, ν(C- O-C)$_{methoxy}$ =1030.23 cm^{-1} , γ(=C-H)$_{carbamate}$ =786.67 cm^{-1} , γ(N-H)$_{carbamate}$=707.18 cm^{-1}. ^{1}H NMR (500 MHz, $CDCl_3$, 25ºC) δ 9.13 (s, 1H, NH-1), 7.74 – 7.70 (m, 2H, H-32,34), 7.30 (s, 1H, H-19), 7.04 – 7.00 (m, 2H, H-31,35), 6.95 (d, $^3J_{H3,H4}$ = 11.7 Hz, 1H, H-3), 6.62 – 6.54 (m, 1H, H-4), 6.41 (t, $^3J_{H29,NH17}$ = 5.8 Hz, 1H, NH-17), 5.91 – 5.85 (m, 2H, H-5,9), 5.87 – 5.84 (m, 1H, H-5), 5.19 (s, 1H, H-7), 4.84 (s, 2H, NH_2-24), 4.69 (dd, ^2J = 14.8 Hz, $^3J_{H29,NH17}$ = 5.9 Hz, 1H, H-29), 4.58 (dd, ^2J= 14.8 Hz, $^3J_{H29,NH17}$ = 5.7 Hz, 1H, H-29), 4.33 – 4.29 (m, 1H, H-6), 4.05 (s, 1H, OH-11), 3.59 – 3.54 (m, 1H, H-11), 3.45 – 3.41 (m, 1H, H-12), 3.36 (s, 3H, H-27), 3.27 (s, 3H, H-23), 2.78 – 2.70 (m, 1H, H-10), 2.67 – 2.61 (m, 1H, H-15), 2.36 (dd, ^2J = 14.1 Hz, $J^3_{H15,H14}$ = 10.1 Hz, 1H, H-15), 2.02 (d, $^4J_{H3,H22}$ = 1.3 Hz, 3H, H-22), 1.79 (d, $^4J_{H9,H25}$ = 1.4 Hz, 3H, H-25), 1.77 – 1.73 (m, 2H, H-13), 1.65 (s, 1H, H-14), 1.00 (d, $^3J_{H10,H26}$= 7.4 Hz, 3H, H-26), 0.99 (d, $^3J_{H14,H28}$= 6.9 Hz, 3H, H-28). ^{13}C NMR (126 MHz, $CDCl_3$, 25ºC) δ 183.9 (C-18), 181.4 (C-21), 168.5 (C-1), 156.2 (C-24), 144.7 (C-17), 141.3 (C-20), 138.4* (C-32,34), 136.5 (C-30), 136.1 (C-5), 135.1 (C-2), 133.8 (C-9), 133.0 (C-8), 129.6* (C-31,35), 127.1 (C-3), 126.7 (C-4), 109.5 (C-16), 109.0 (C-19), 94.1 (C-33), 81.8 (C-7), 81.5 (C-6), 81.3 (C-12), 72.8 (C-11), 57.3 (C-23), 56.9 (C-27), 49.6 (C-29), 35.1 (C-13), 34.6 (C-15), 32.5 (C-10), 28.7 (C-14), 23.1 (C-28), 12.9 (C-25), 12.7 (C-22), 12.5 (C-26). (*-overlapped)

FT-IR spectra

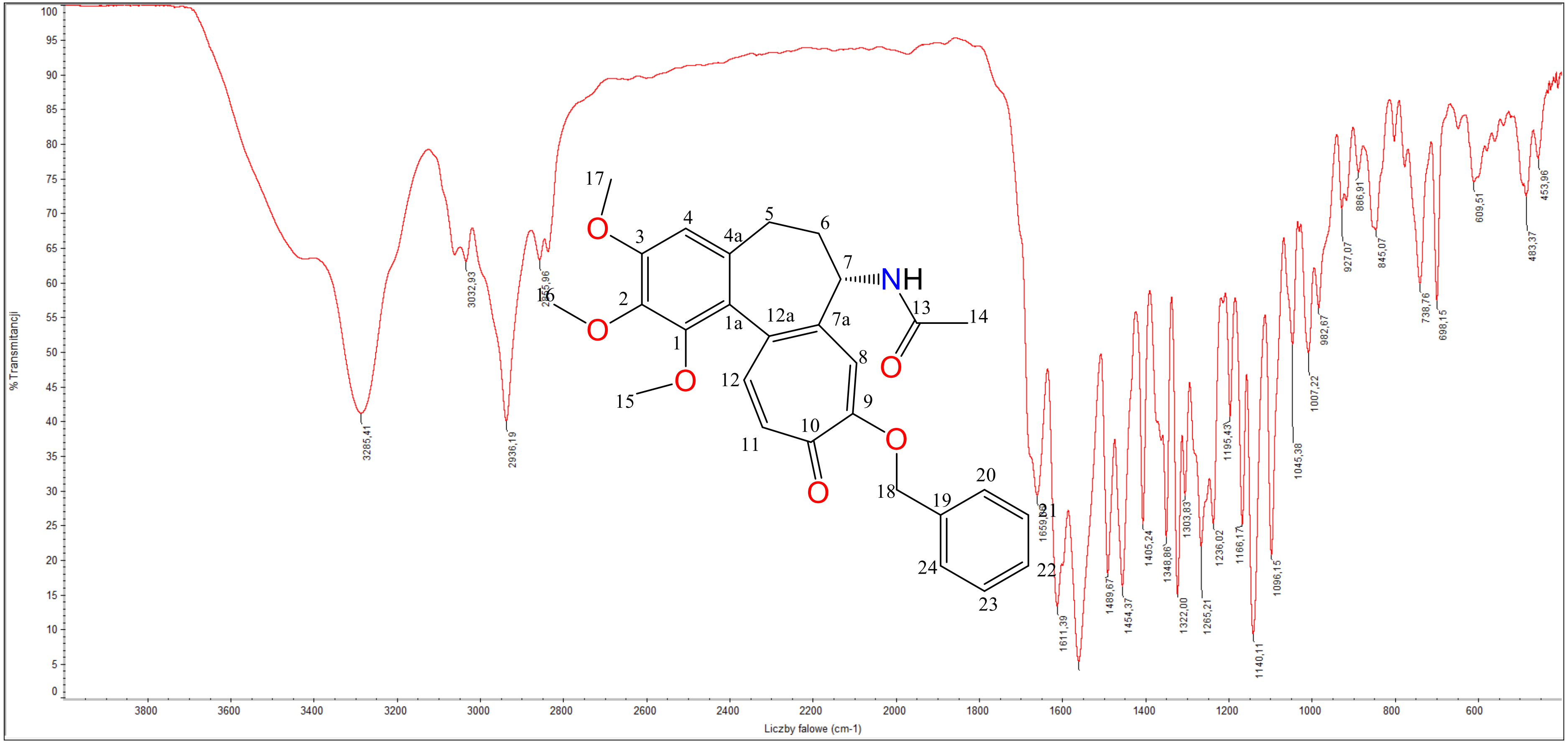

Figure 8S. FT-IR spectrum of compound **3a** (in KBr).

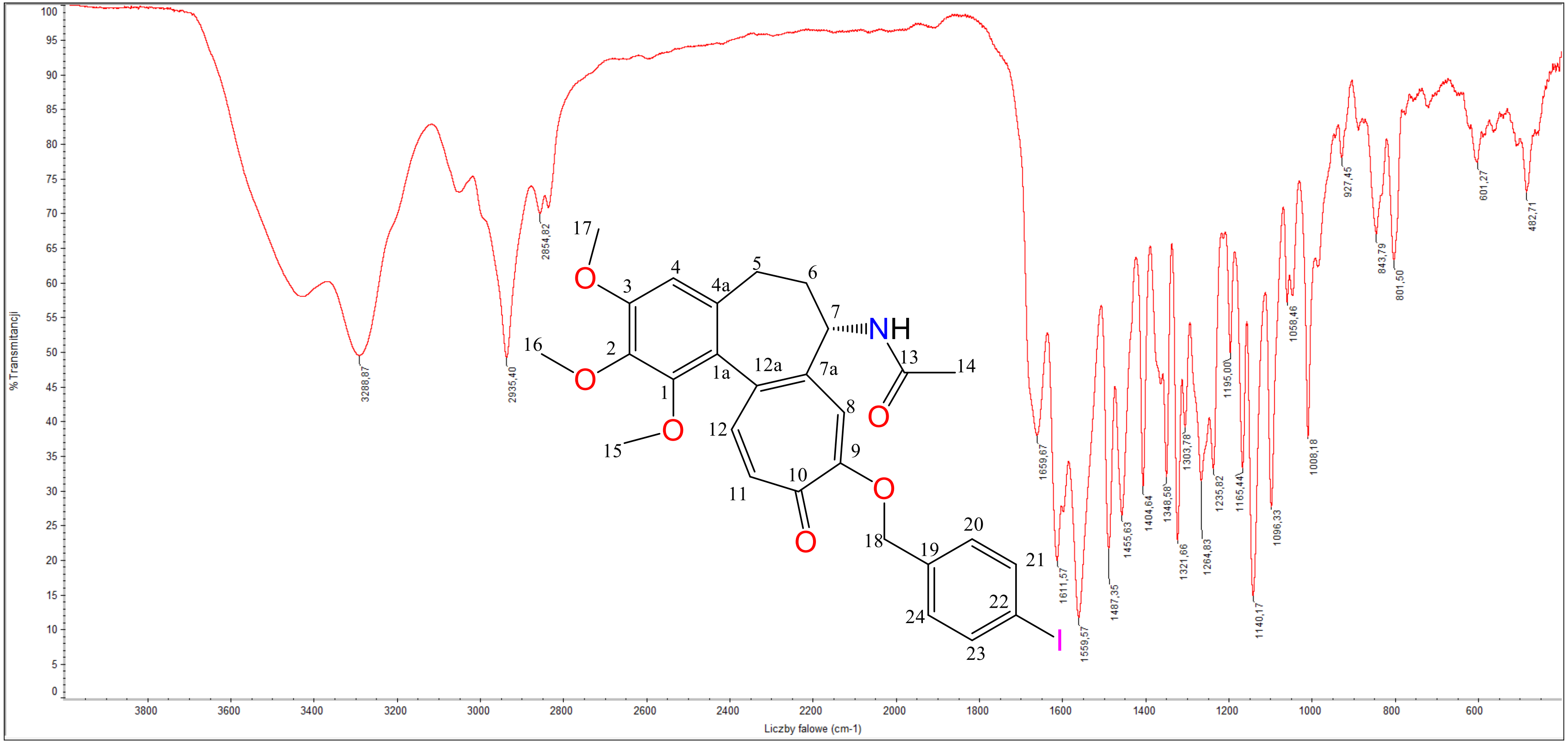

Figure 9S. FT-IR spectrum of compound **3b** (in KBr).

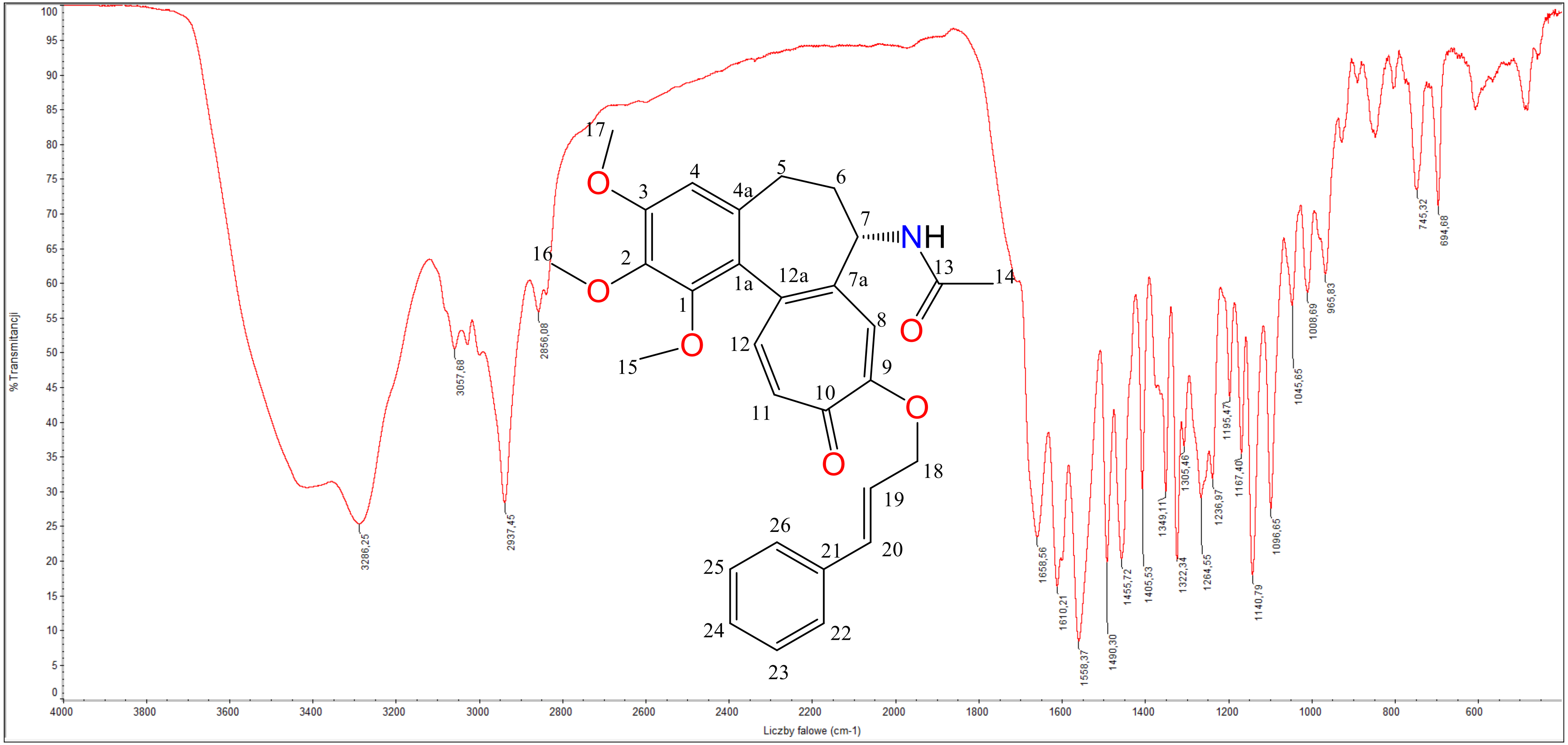

Figure 10S. FT-IR spectrum of compound **3c** (in KBr).

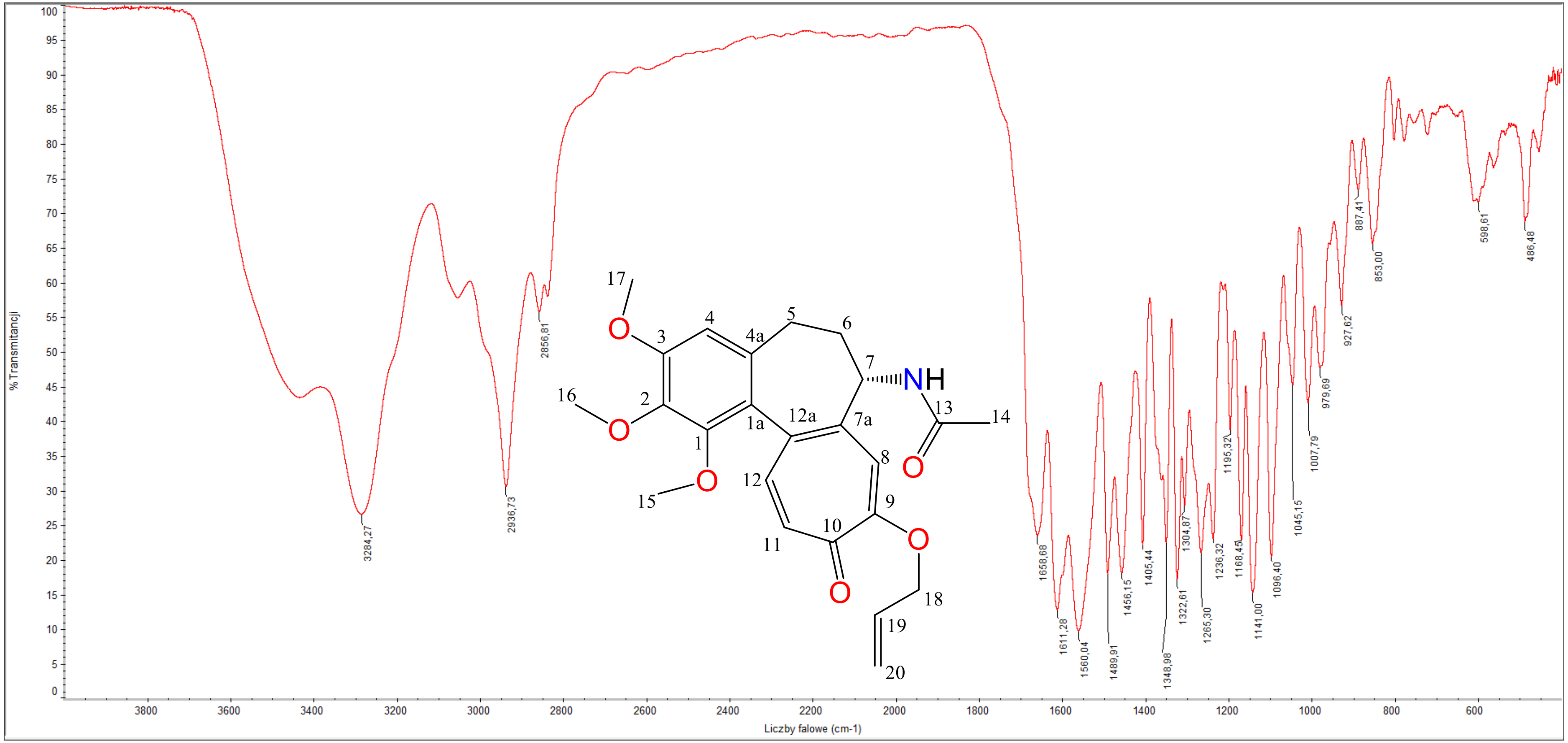

Figure 11S. FT-IR spectrum of compound **3d** (in KBr).

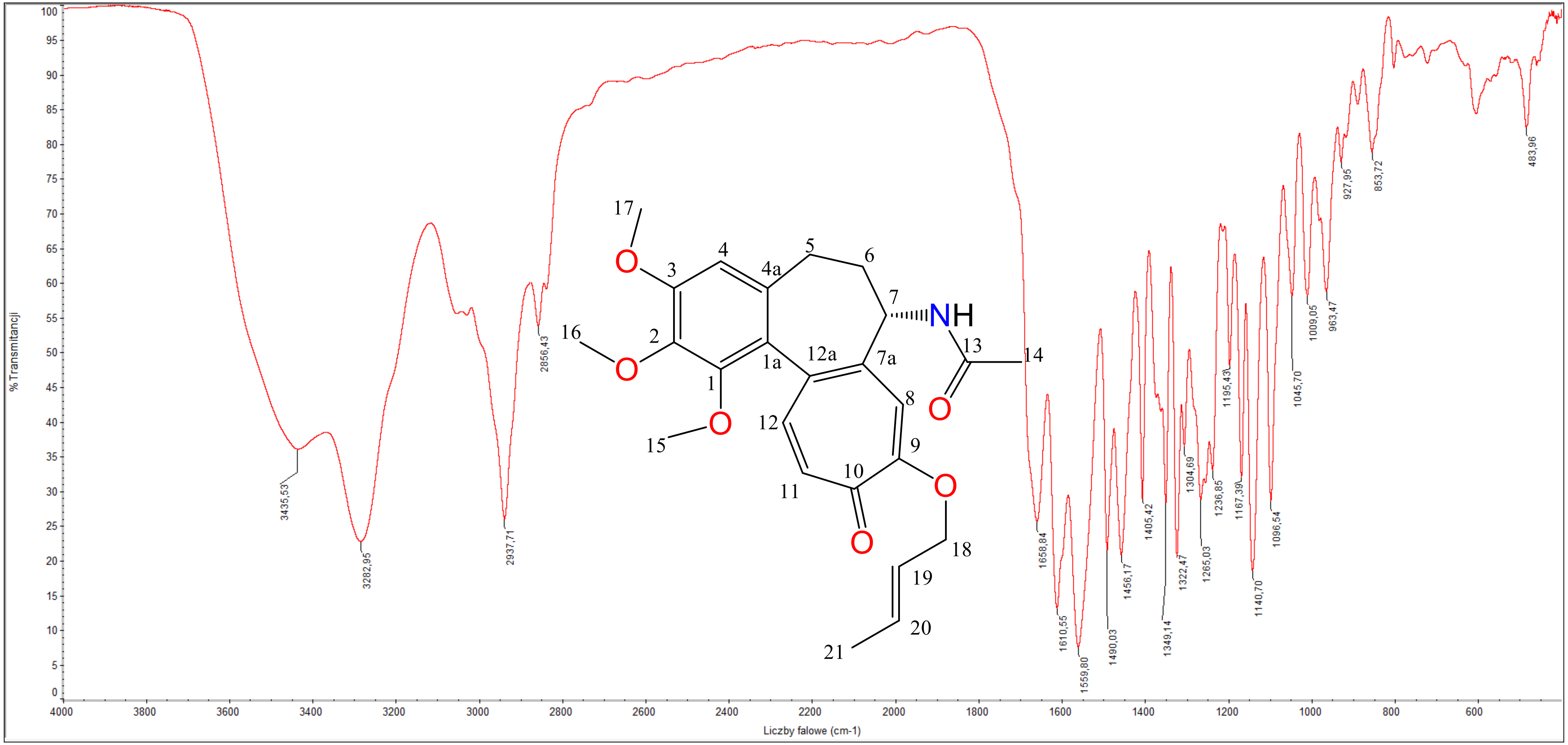

Figure 12S. FT-IR spectrum of compound **3e** (in KBr).

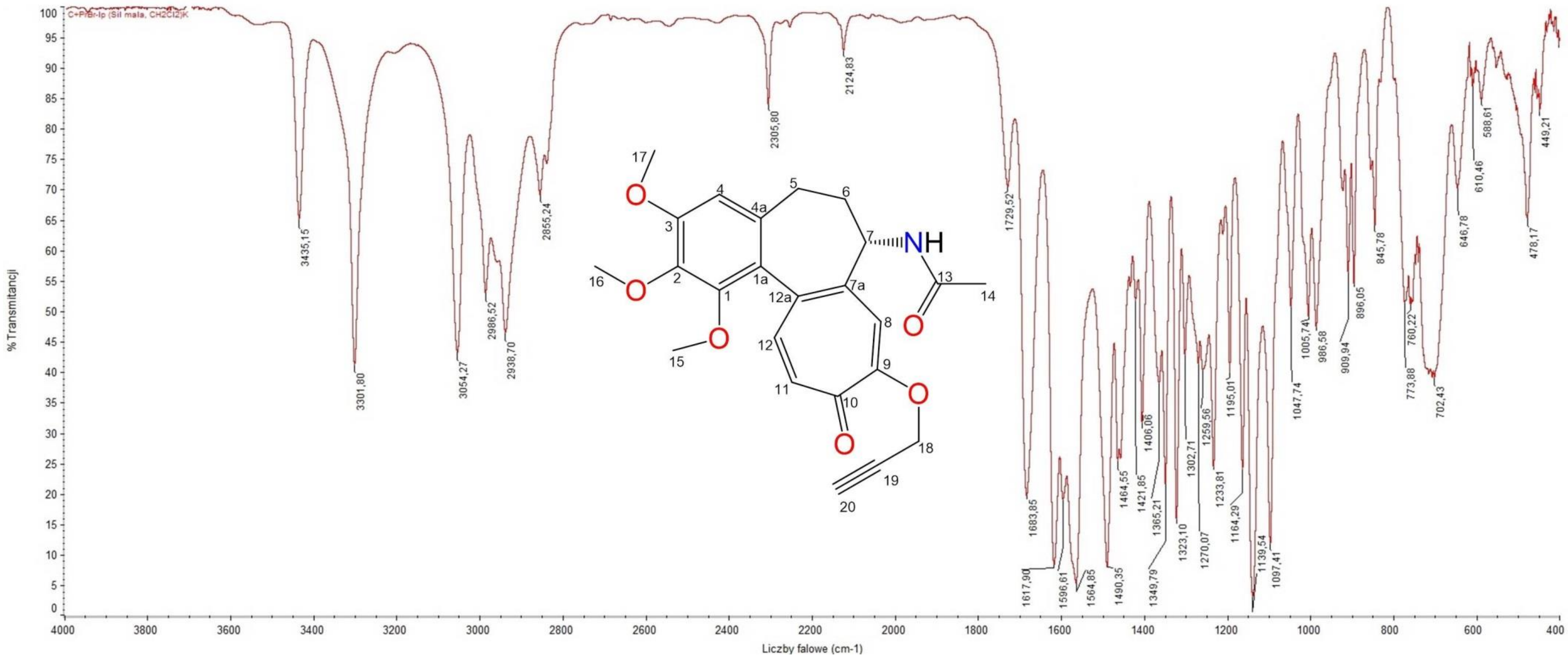

Figure 13S. FT-IR spectrum of compound **3f** (in CH_2Cl_2).

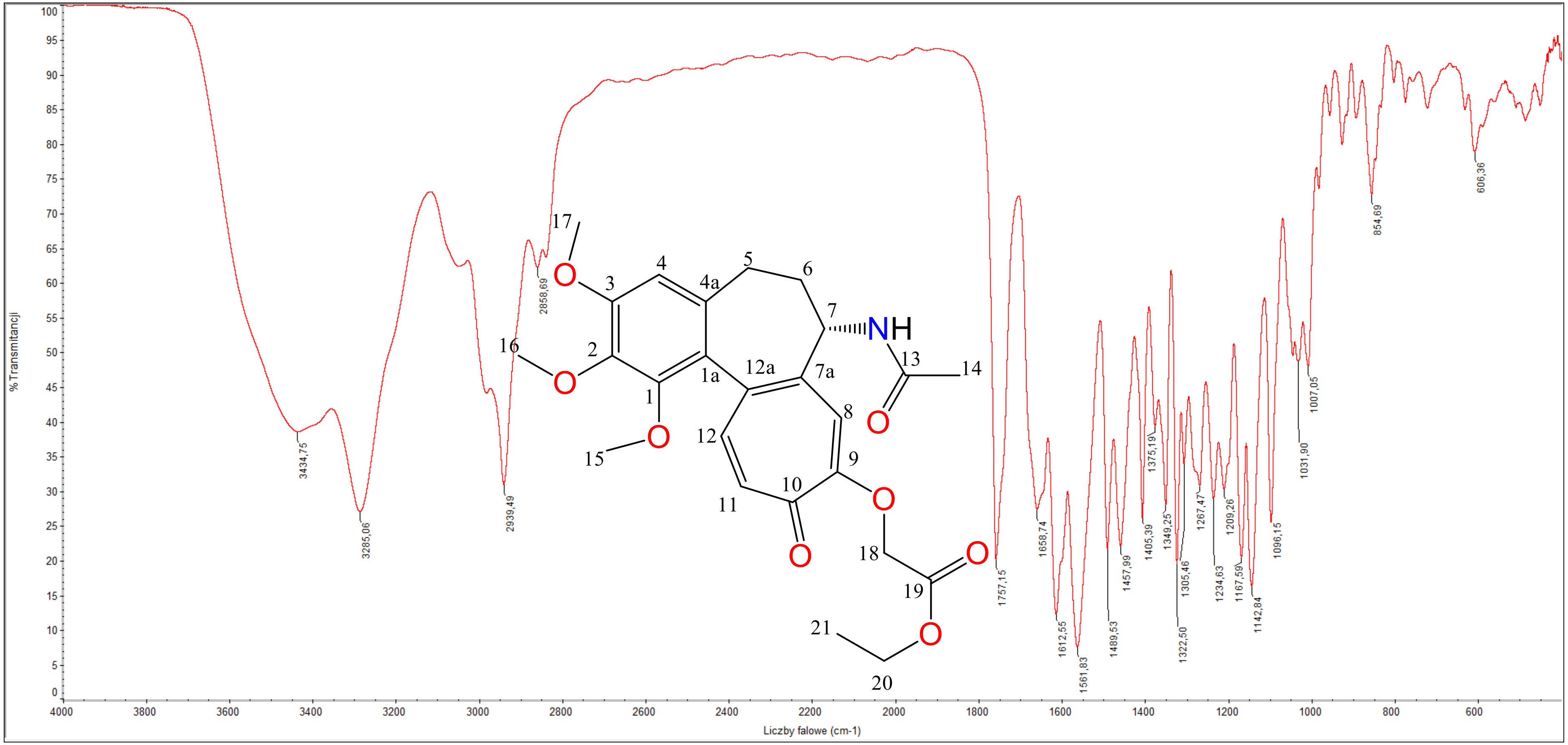

Figure 14S. FT-IR spectrum of compound **3g** (in KBr).

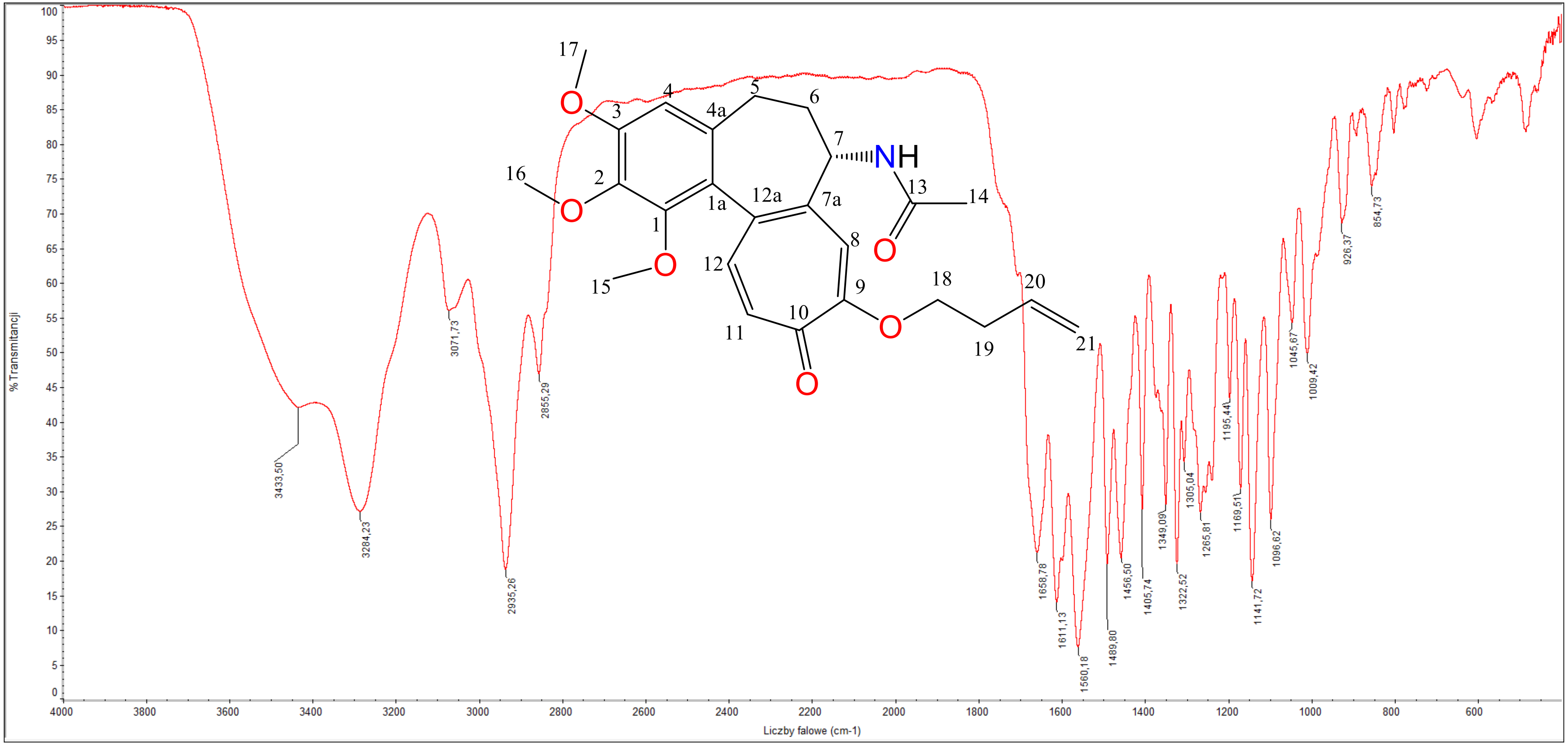

Figure 15S. FT-IR spectrum of compound **3h** (in KBr).

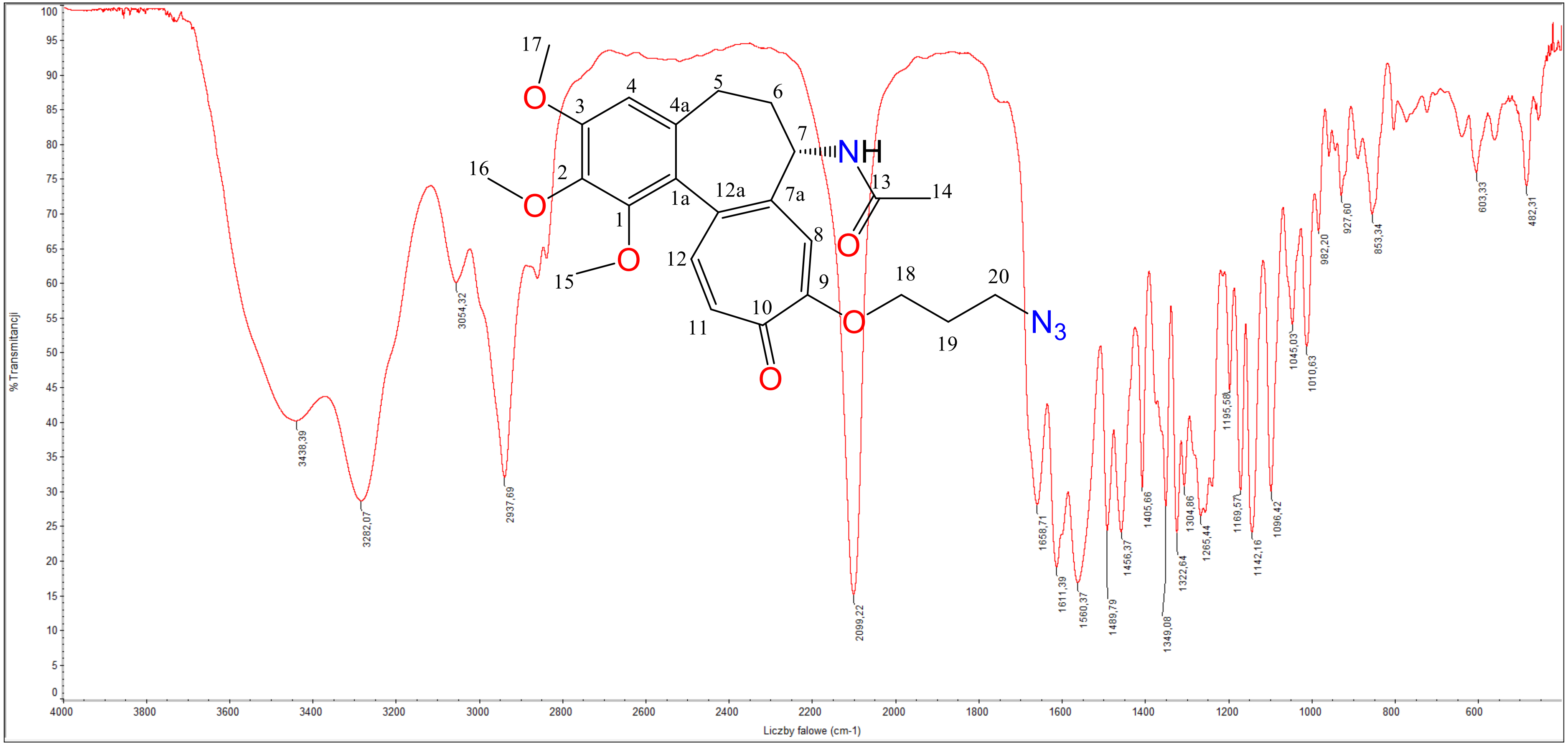

Figure 16S. FT-IR spectrum of compound **3i** (in KBr).

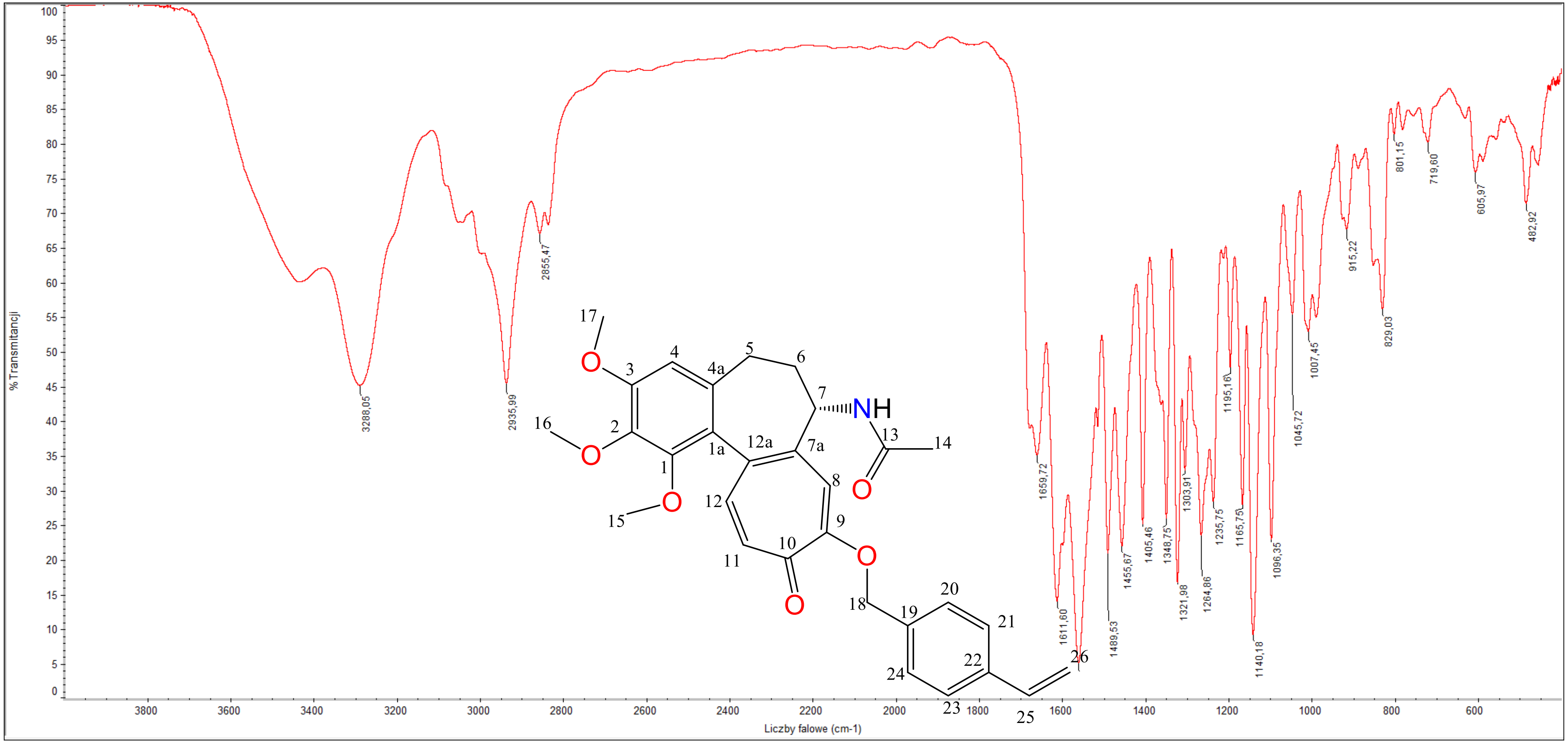

Figure 17S. FT-IR spectrum of compound **3j** (in KBr).

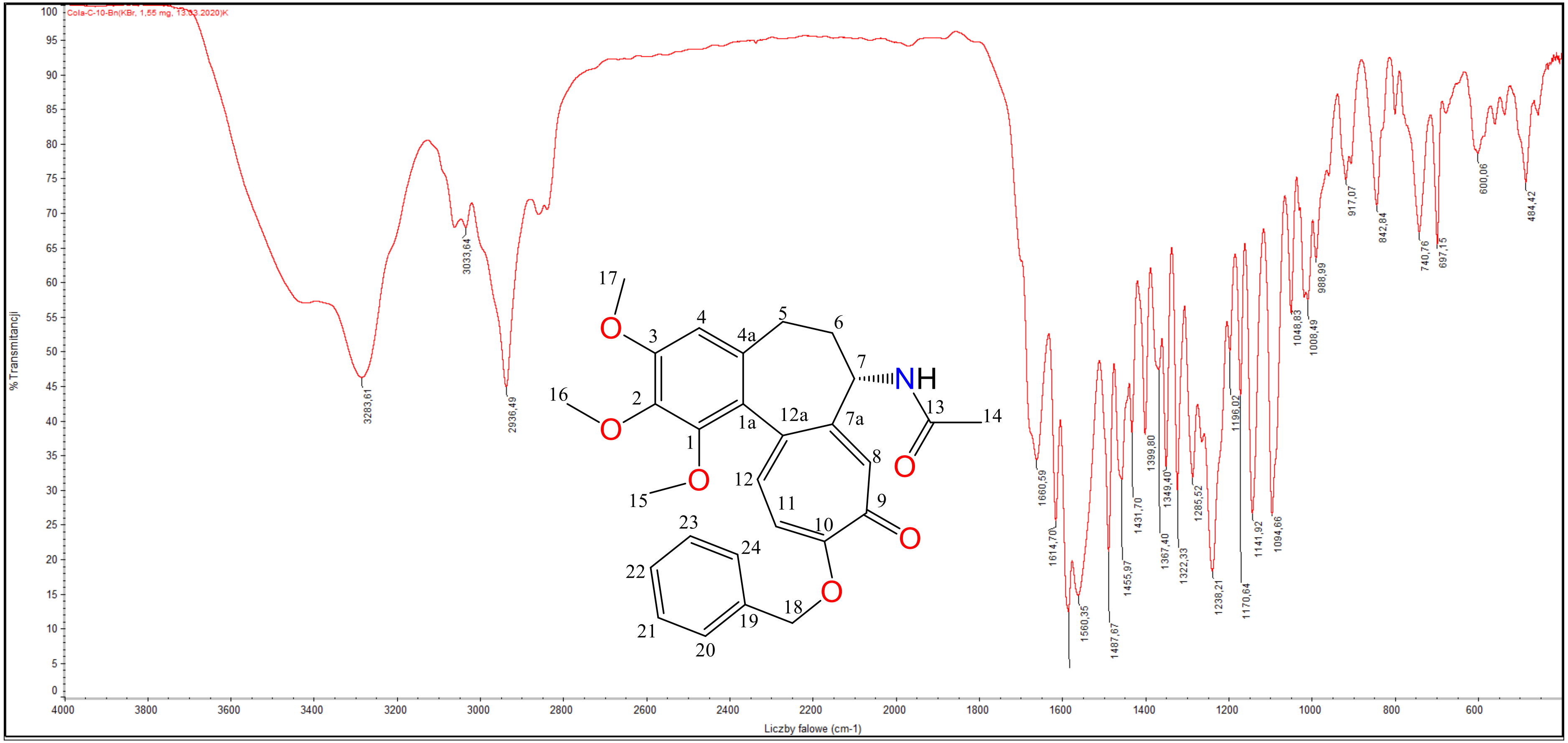

Figure 18S. FT-IR spectrum of compound **4a** (in KBr).

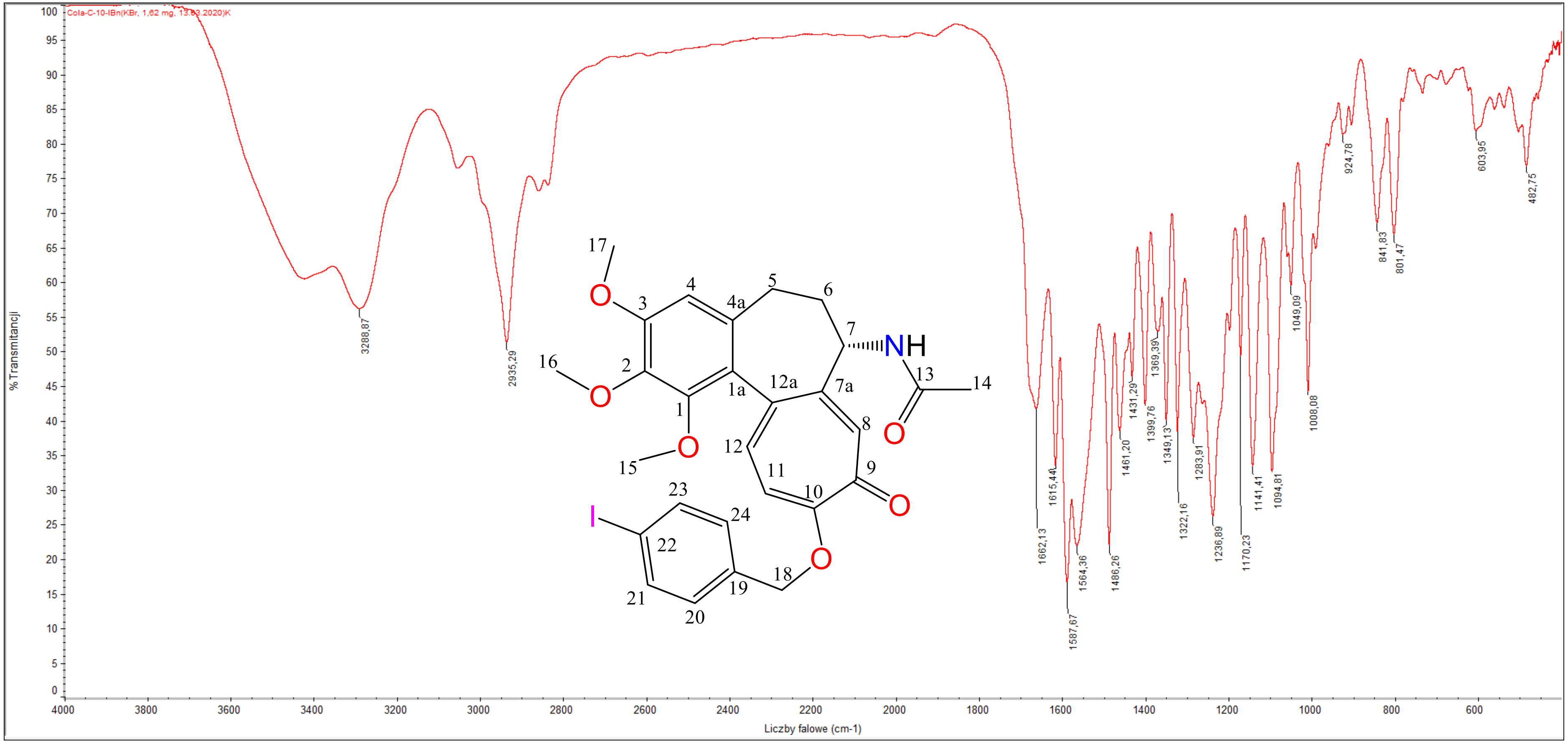

Figure 19S. FT-IR spectrum of compound **4b** (in KBr).

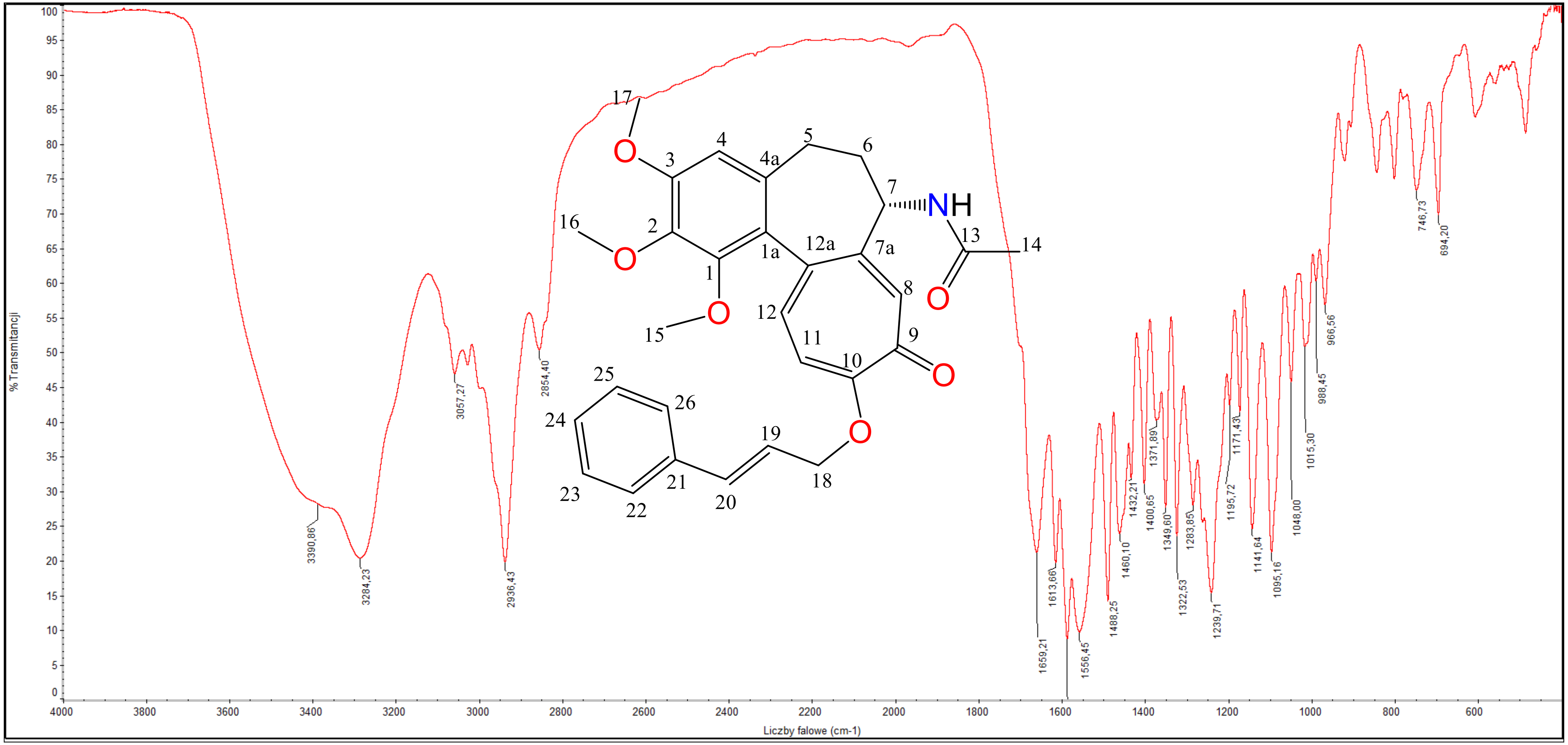

Figure 20S. FT-IR spectrum of compound **4c** (in KBr).

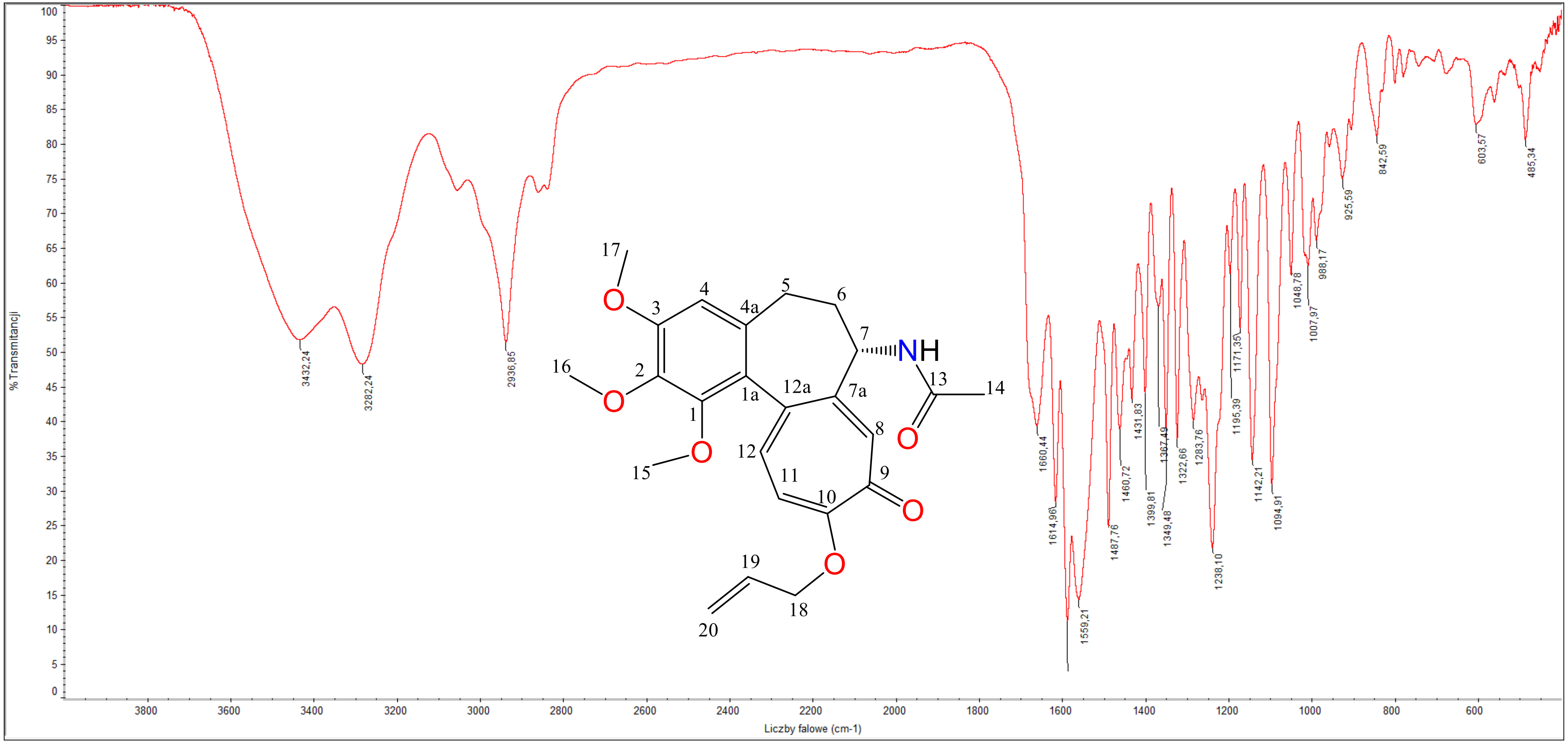

Figure 21S. FT-IR spectrum of compound **4d** (in KBr).

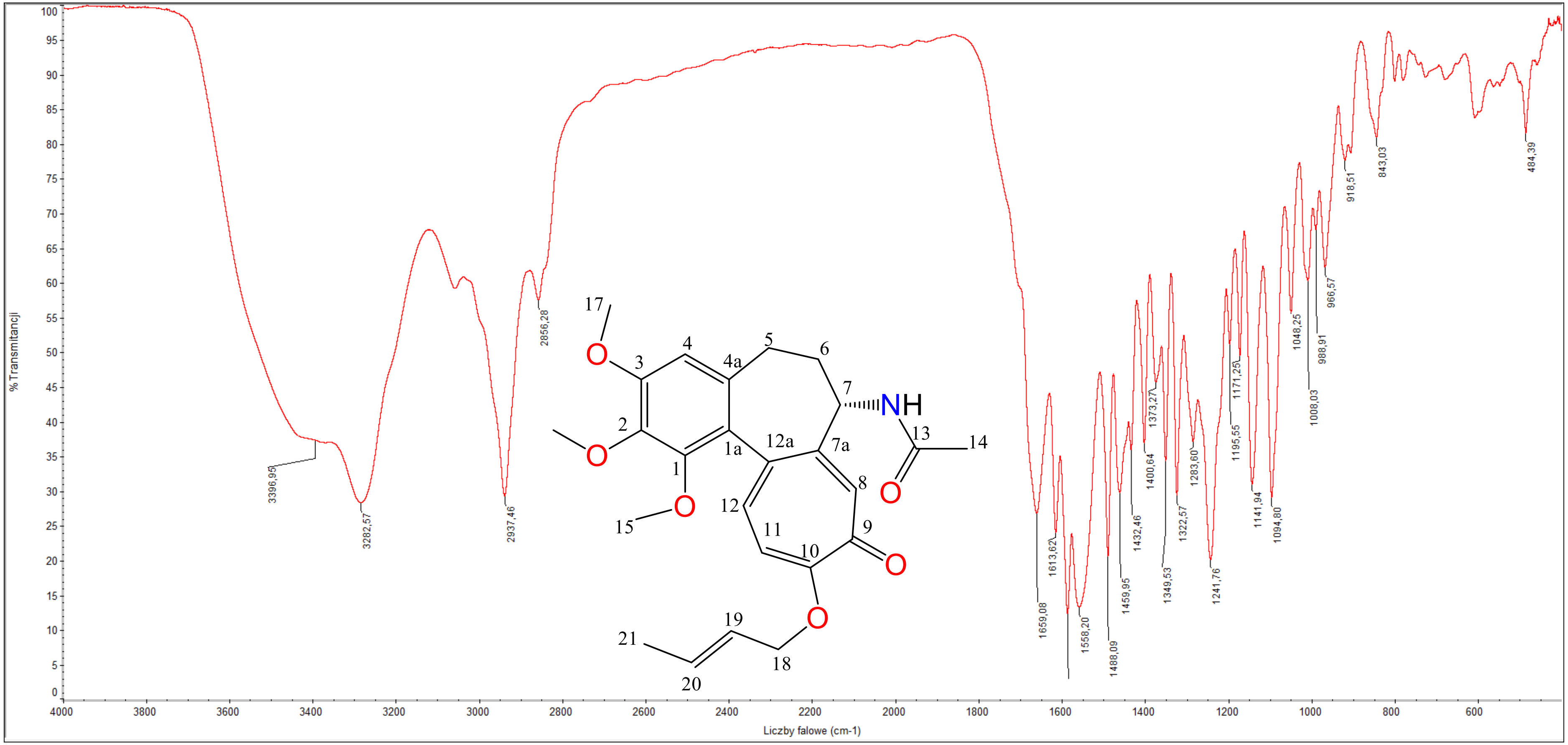

Figure 22S. FT-IR spectrum of compound **4e** (in KBr).

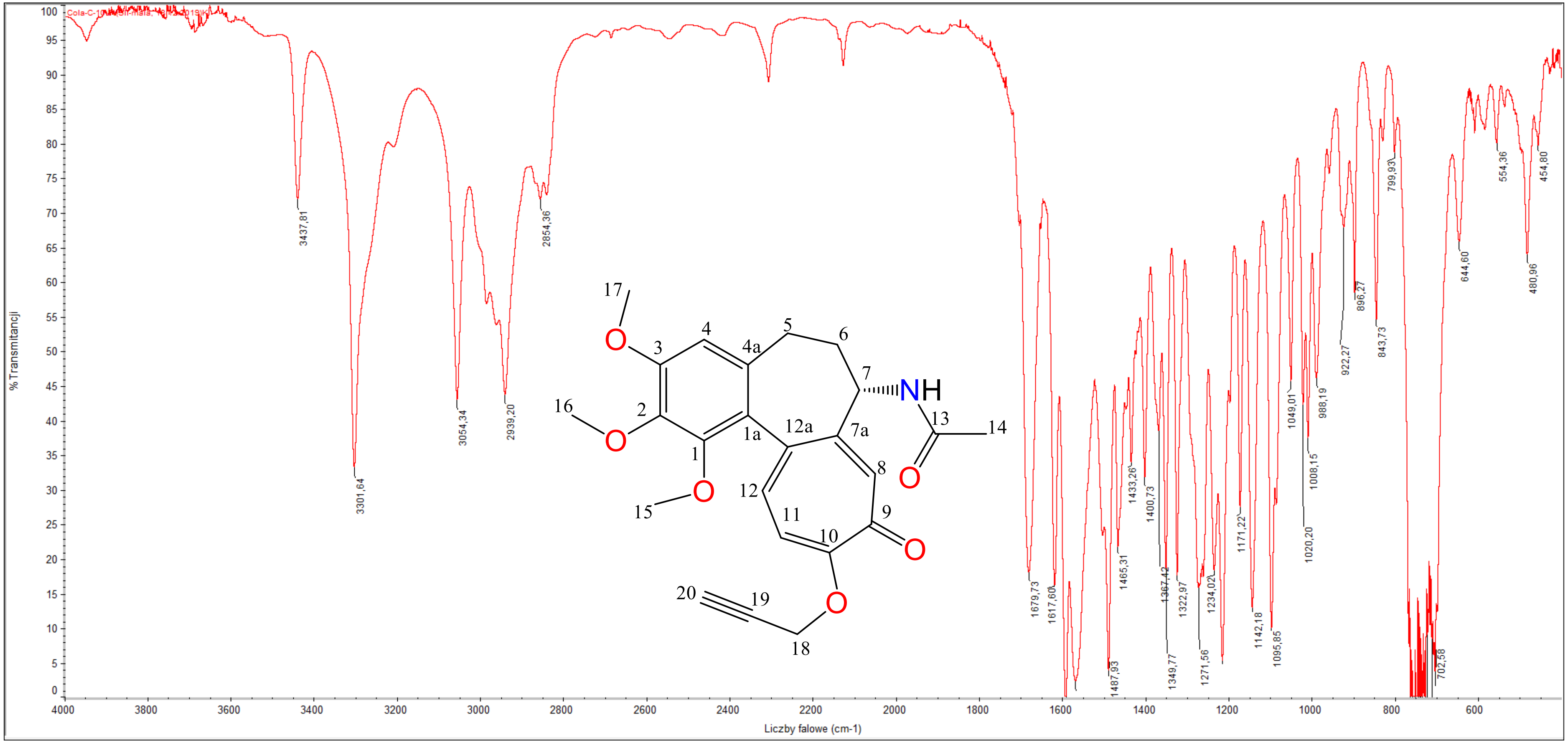

Figure 23S. FT-IR spectrum of compound **4f** (in CH_2Cl_2).

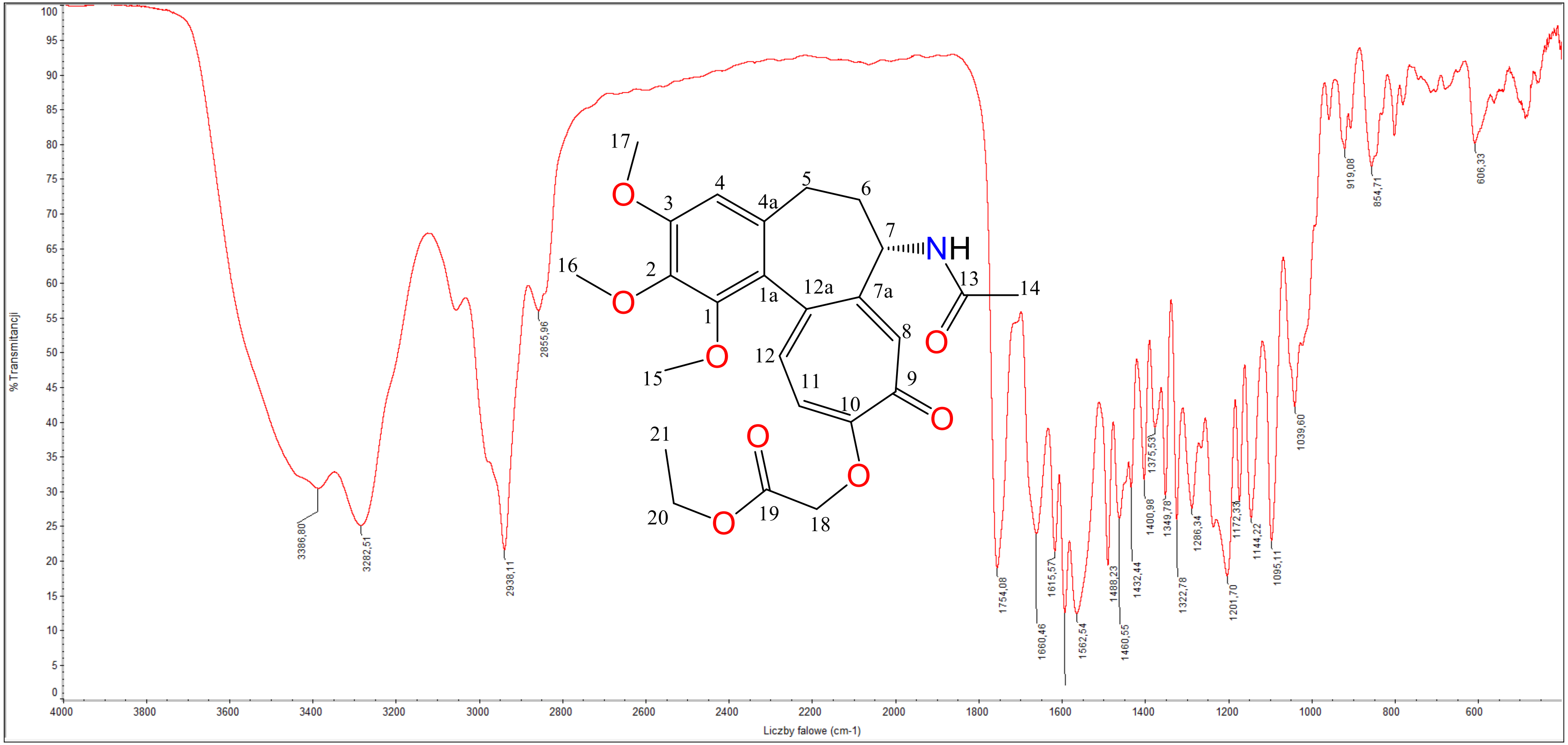

Figure 24S. FT-IR spectrum of compound **4g** (in KBr).

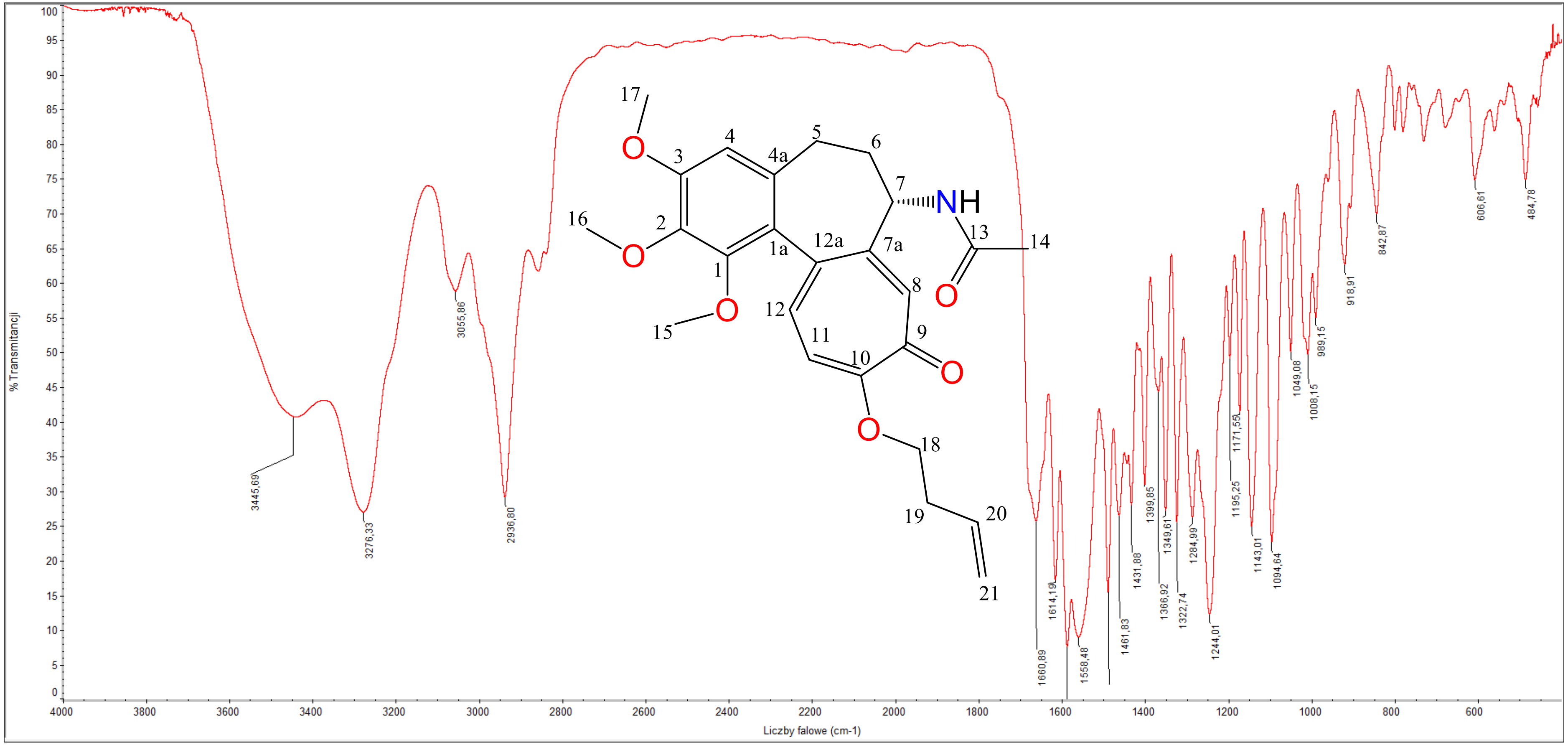

Figure 25S. FT-IR spectrum of compound **4h** (in KBr).

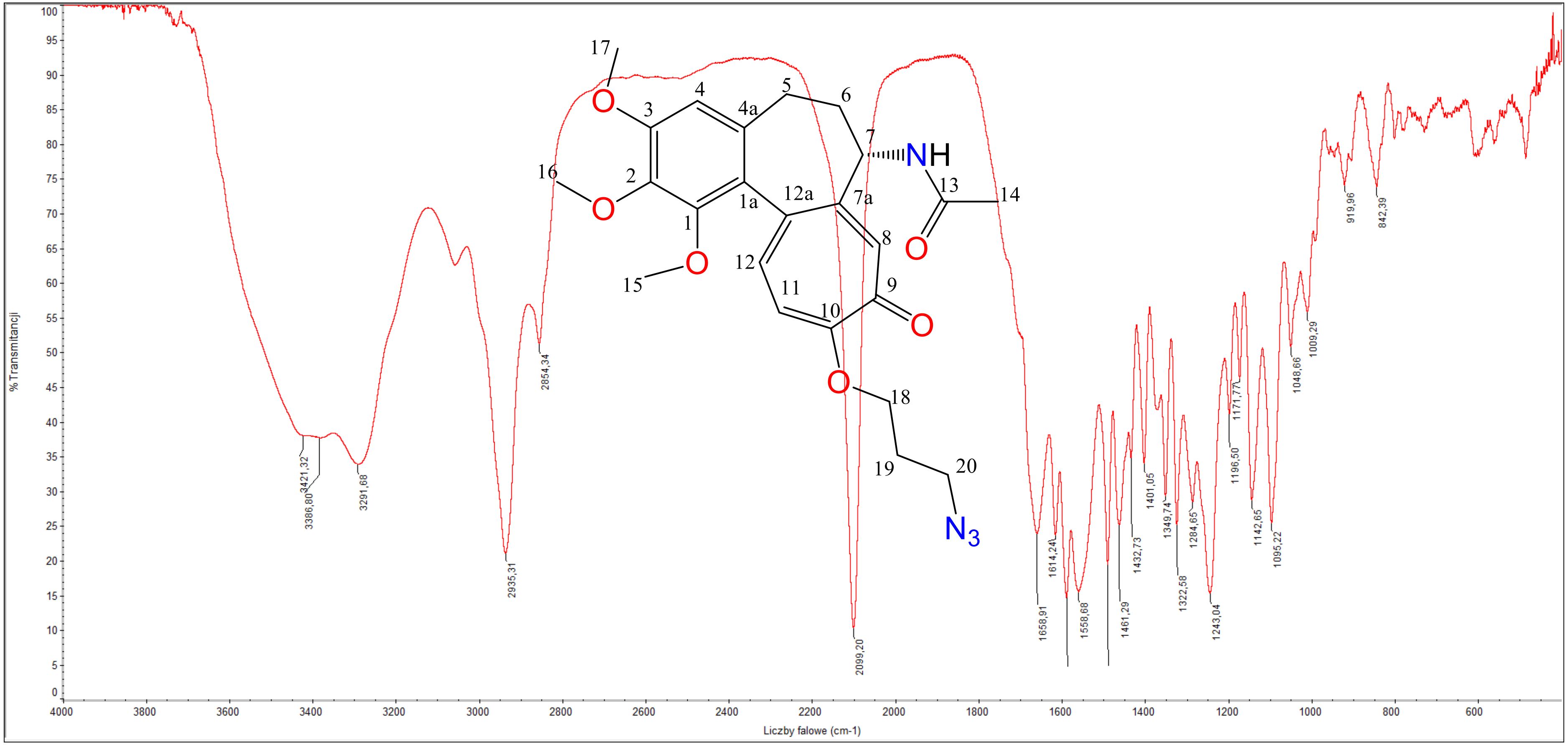

Figure 26S. FT-IR spectrum of compound **4i** (in KBr).

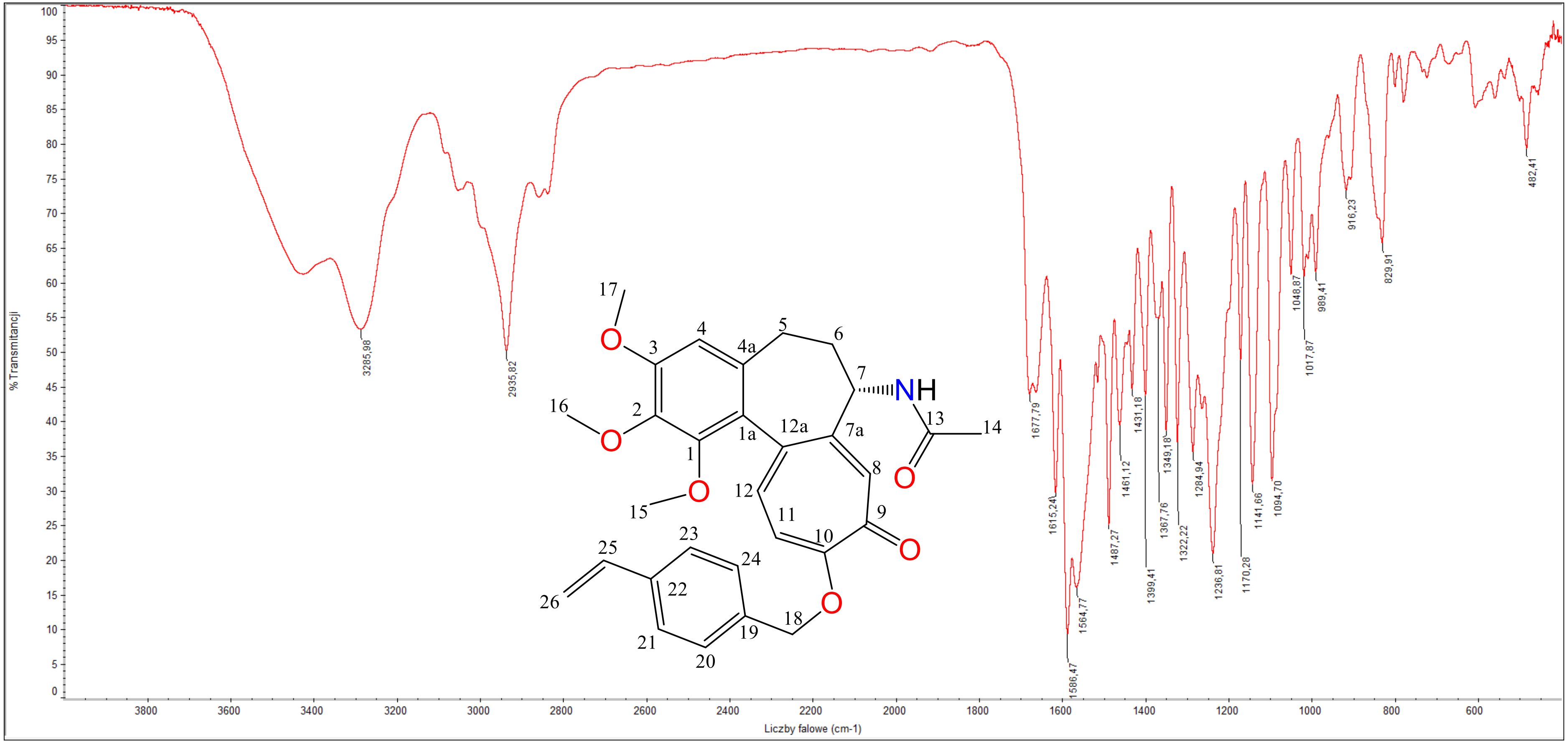

Figure 27S. FT-IR spectrum of compound **4j** (in KBr).

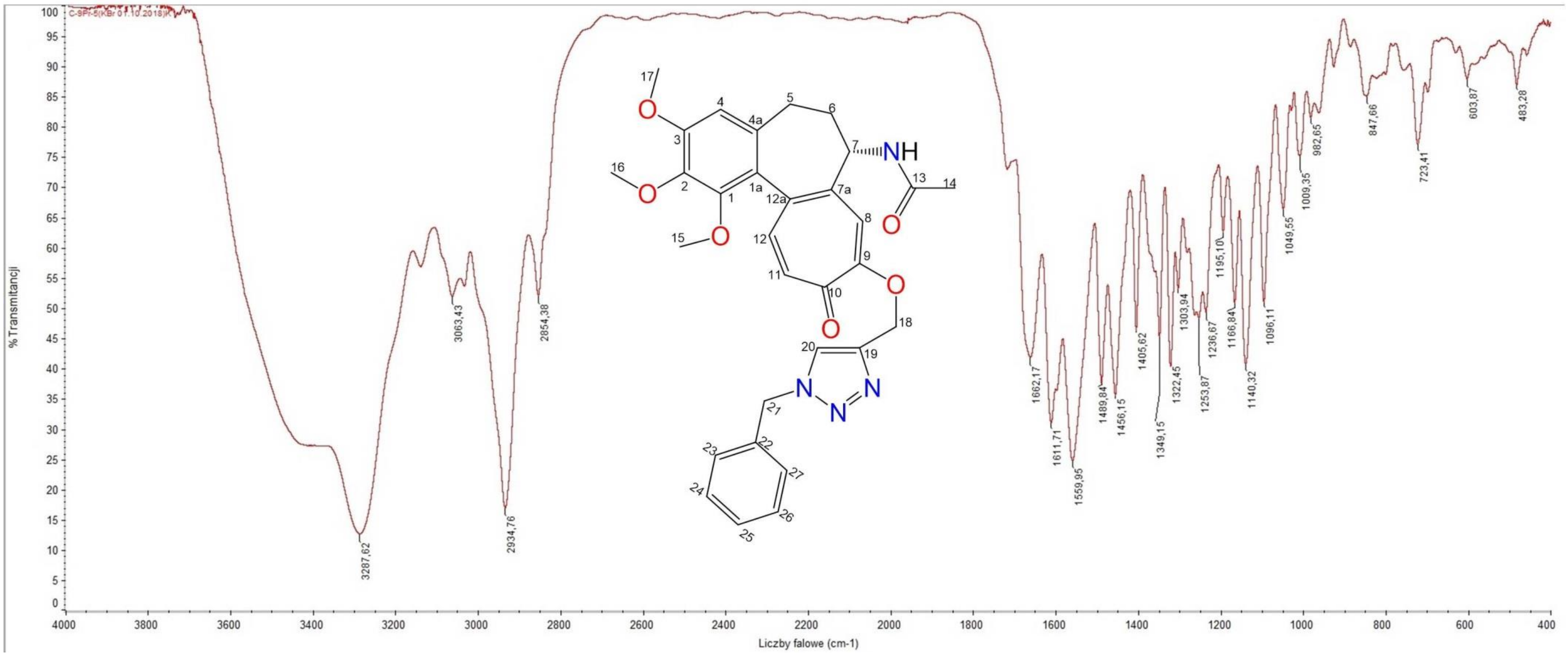

Figure 28S. FT-IR spectrum of compound **5a** (in KBr).

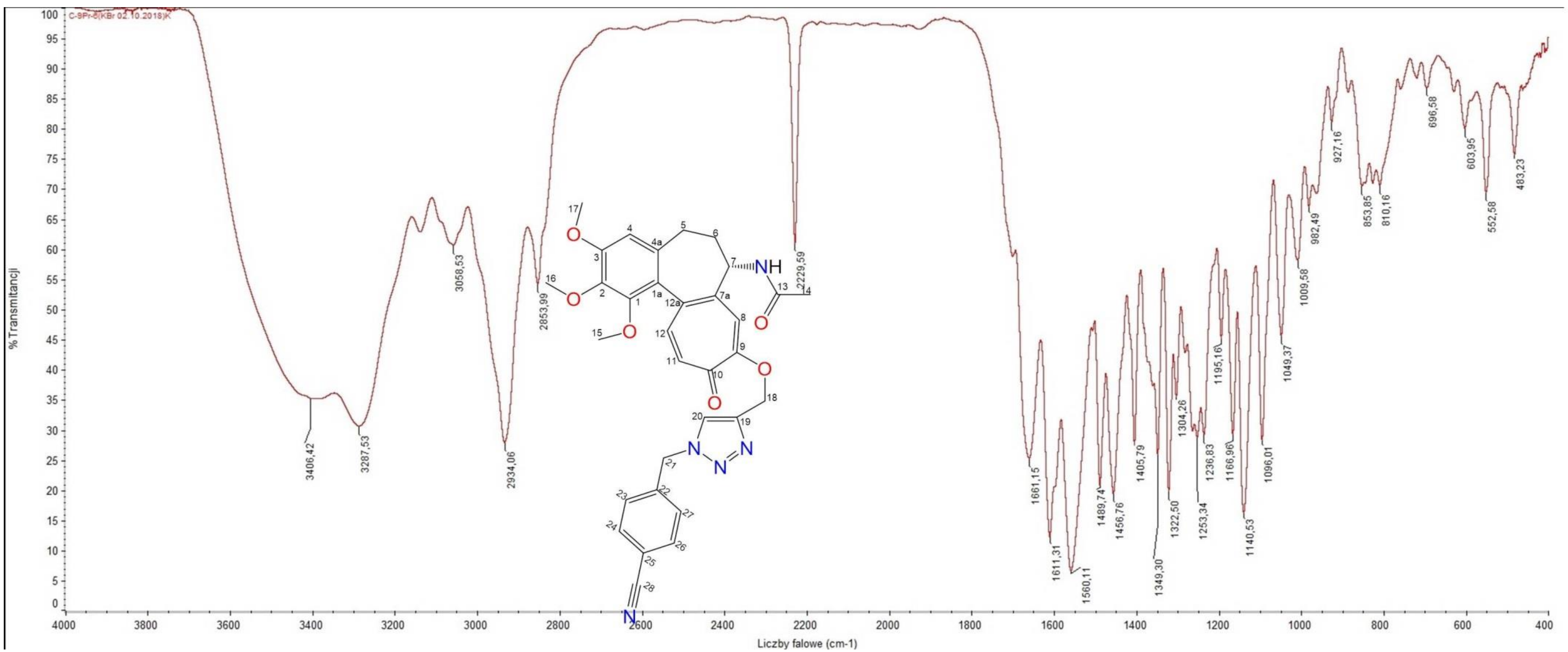

Figure 29S. FT-IR spectrum of compound **5b** (in KBr).

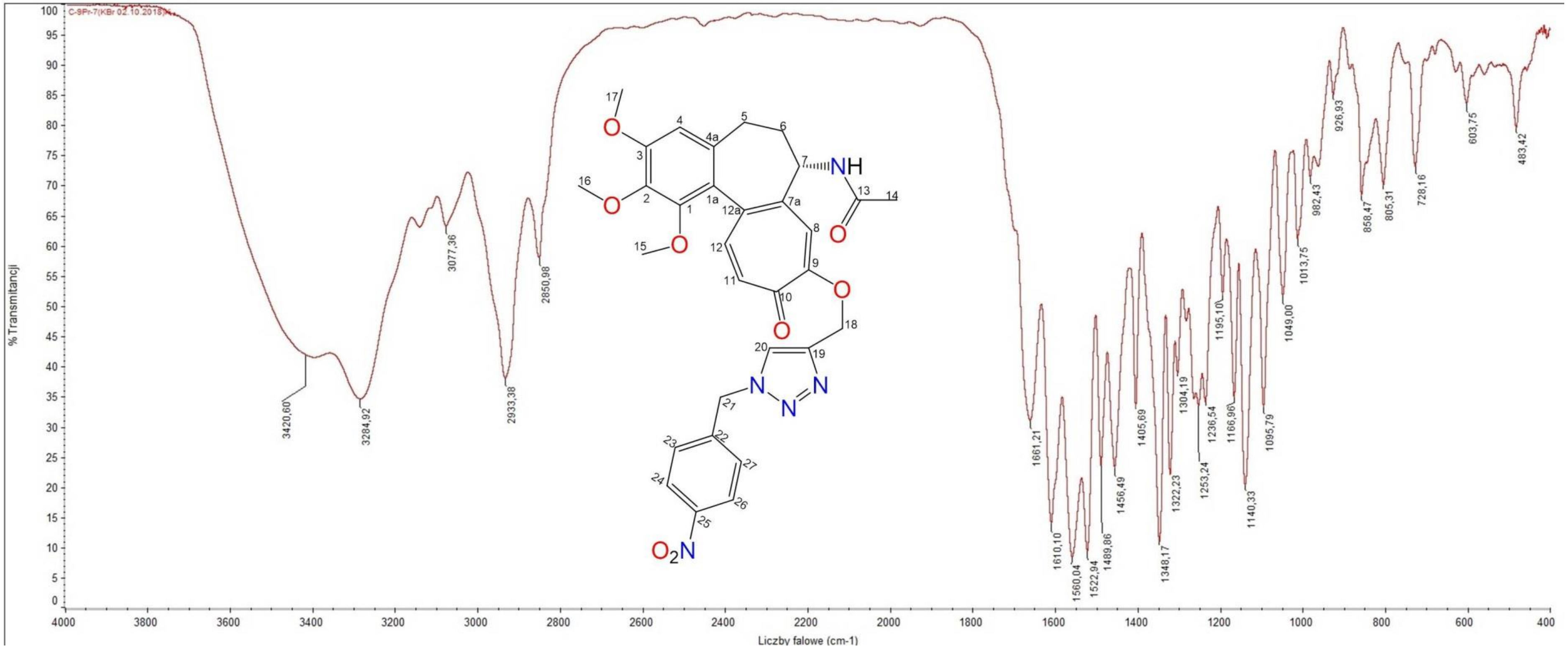

Figure 30S. FT-IR spectrum of compound **5c** (in KBr).

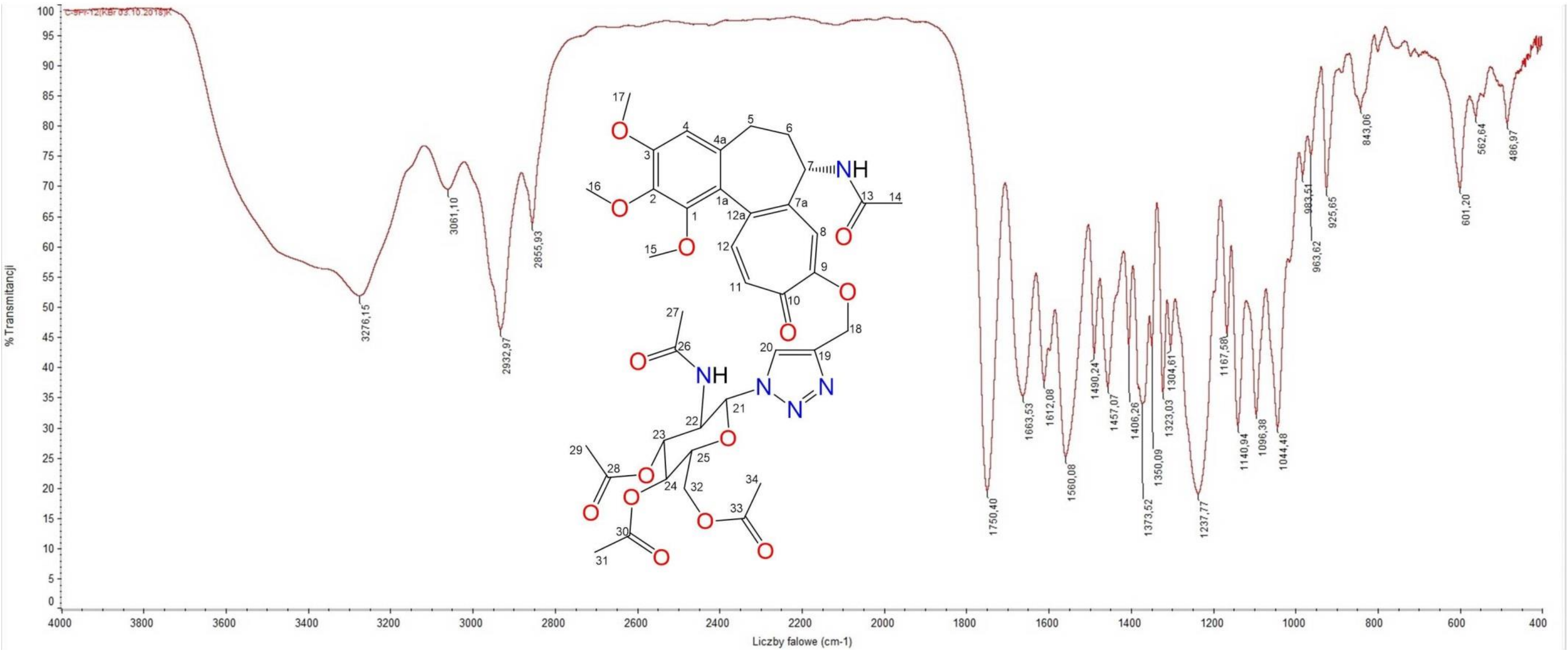

Figure 31S. FT-IR spectrum of compound **5d** (in KBr).

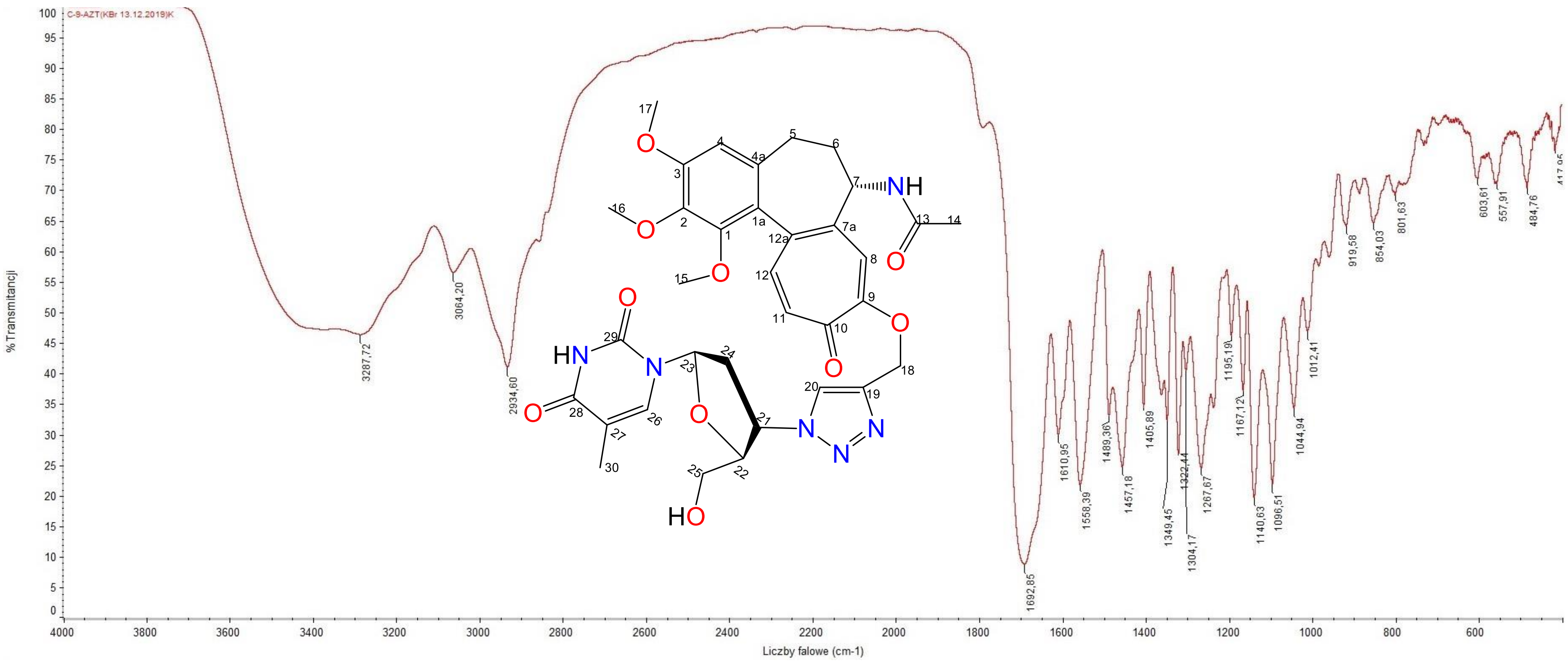

Figure 32S. FT-IR spectrum of compound **5e** (in KBr).

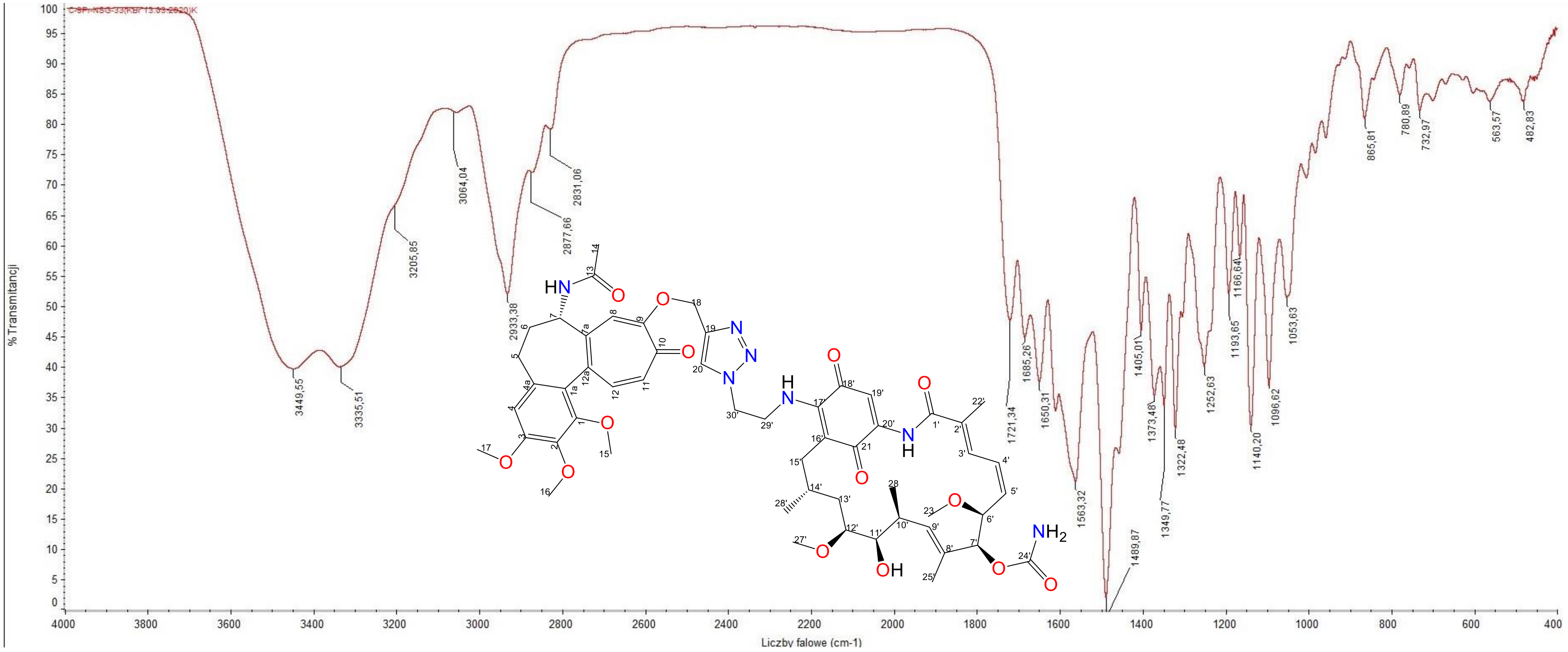

Figure 33S. FT-IR spectrum of compound **5f** (in KBr).

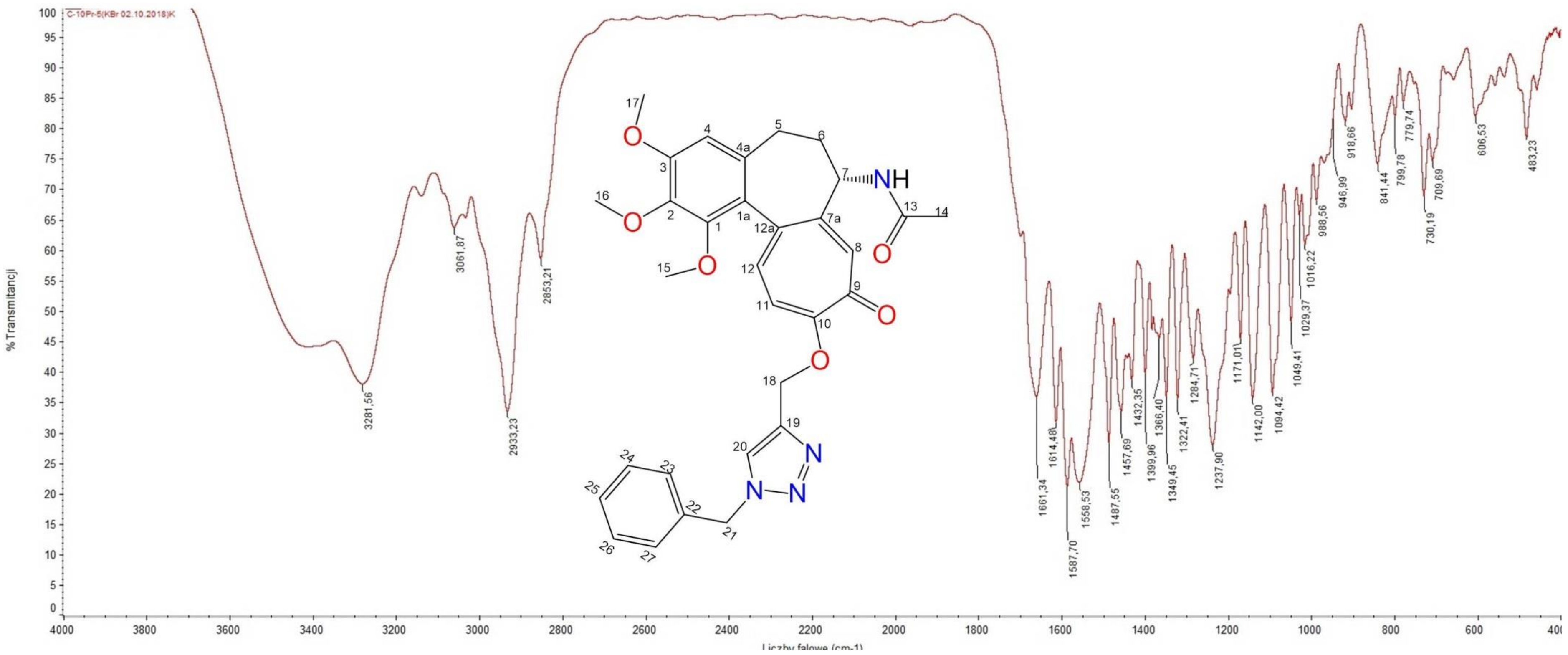

Figure 34S. FT-IR spectrum of compound **6a** (in KBr).

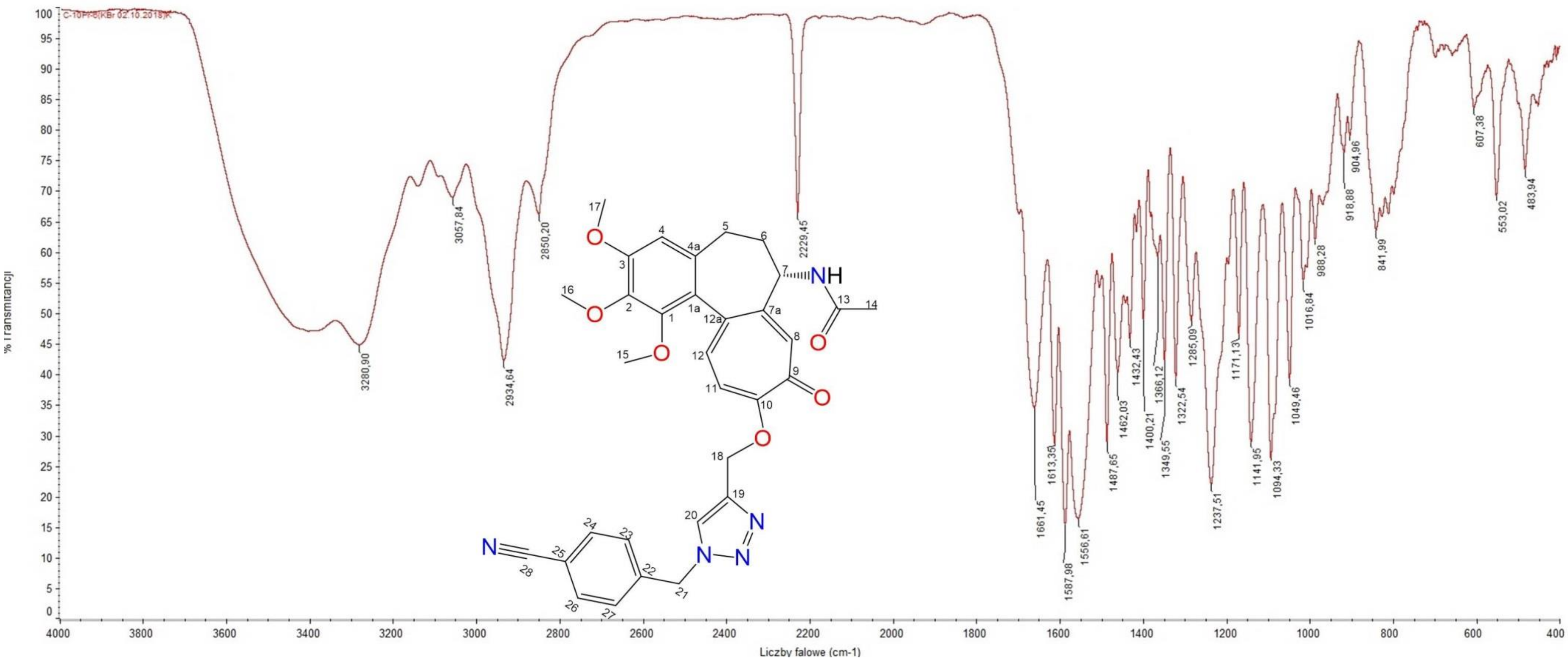

Figure 35S. FT-IR spectrum of compound **6b** (in KBr).

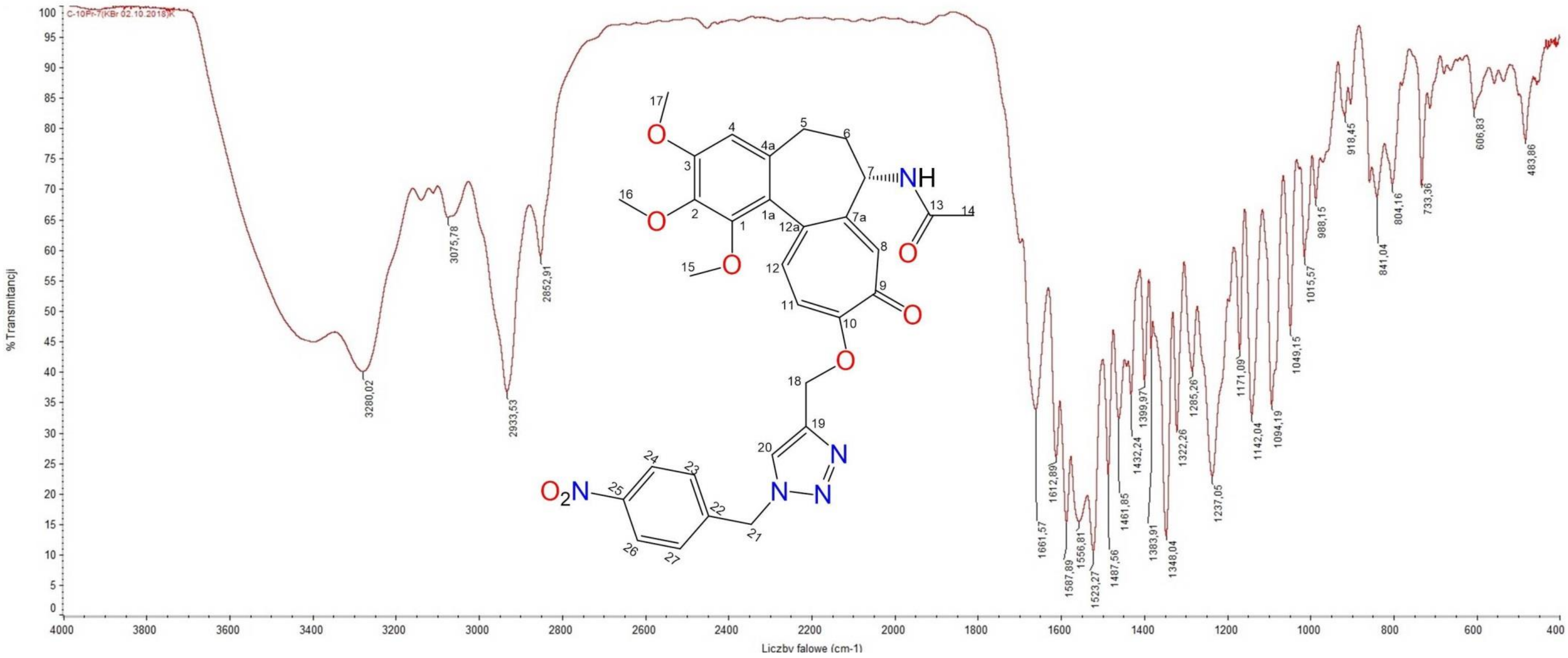

Figure 36S. FT-IR spectrum of compound **6c** (in KBr).

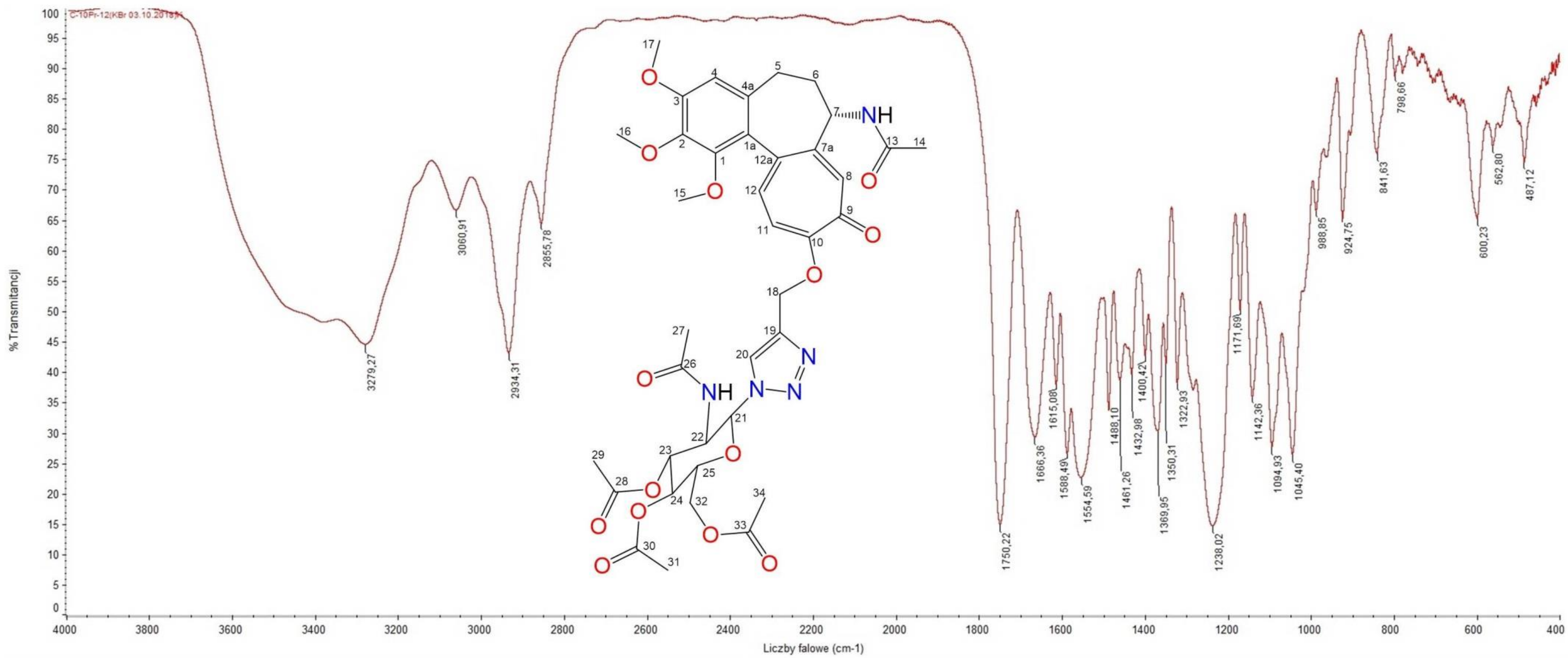

Figure 37S. FT-IR spectrum of compound **6d** (in KBr).

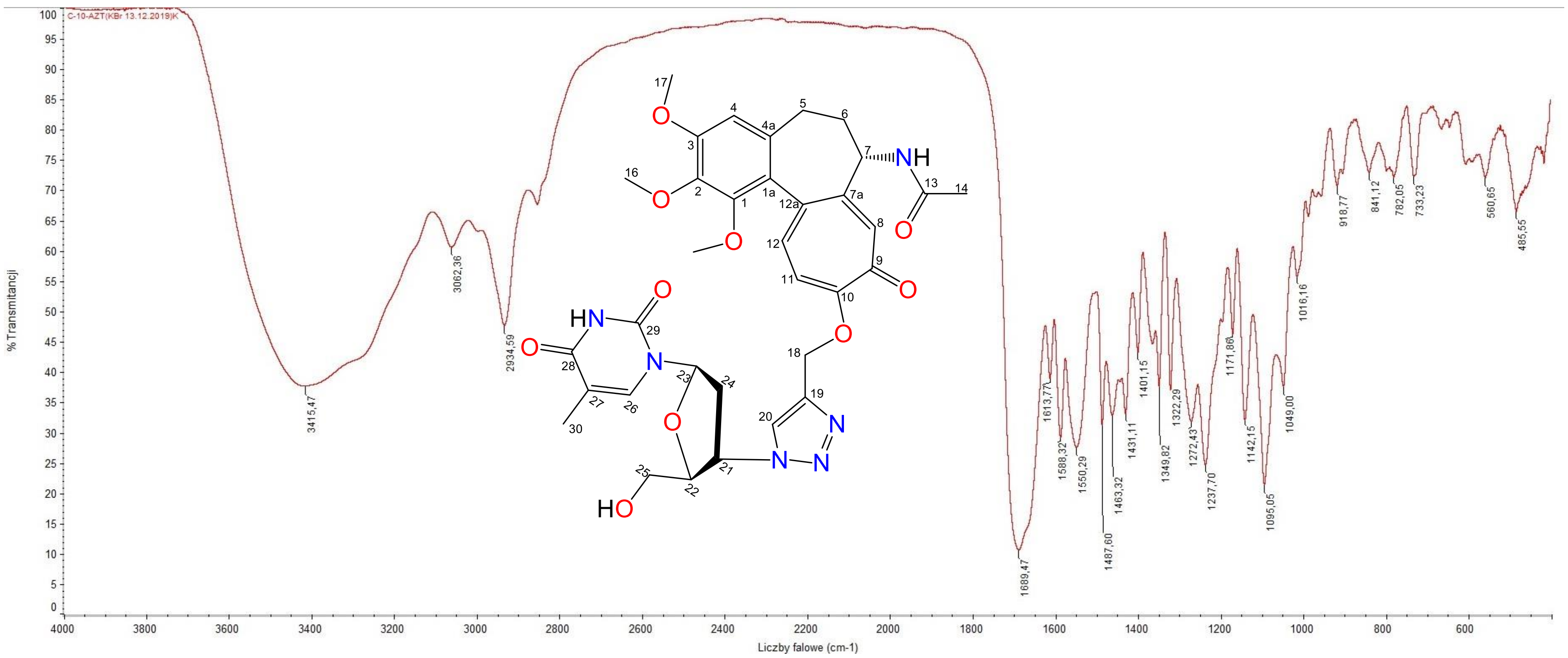

Figure 38S. FT-IR spectrum of compound **6e** (in KBr).

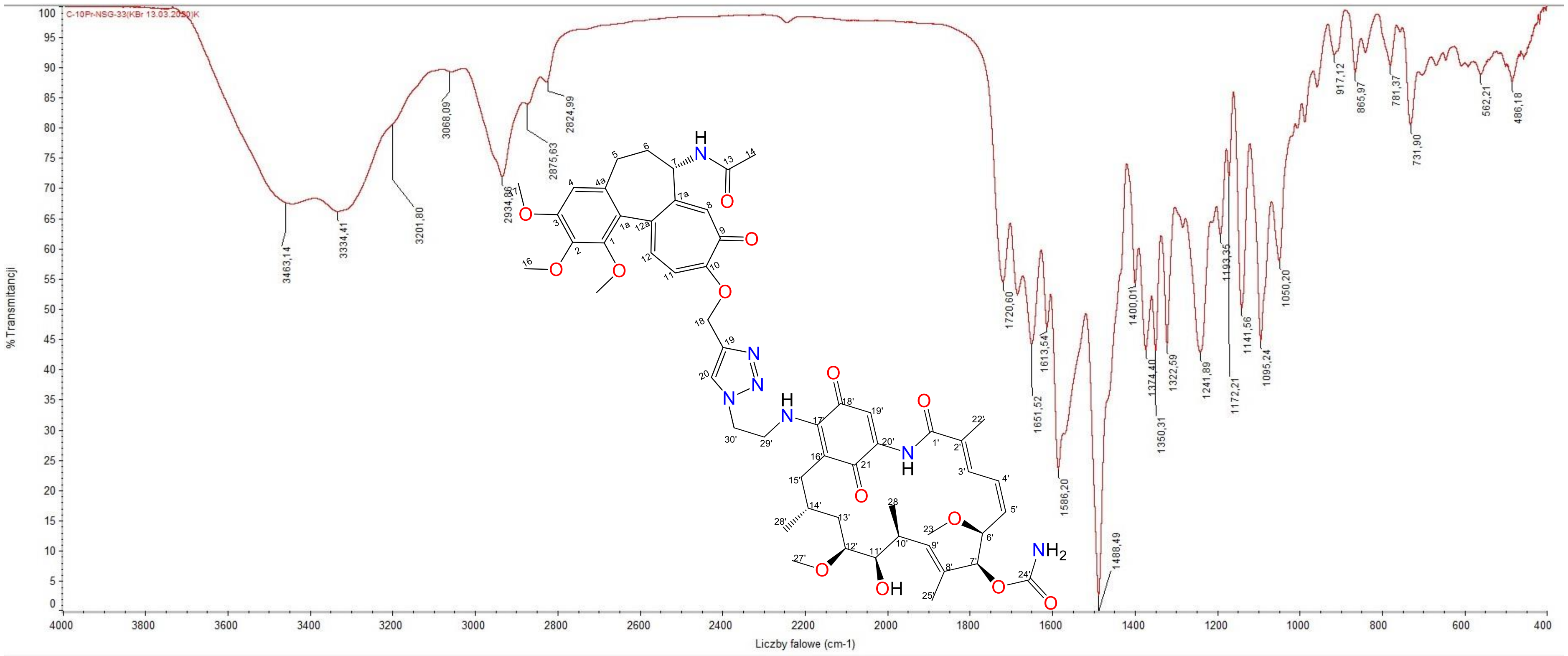

Figure 39S. FT-IR spectrum of compound **6f** (in KBr).

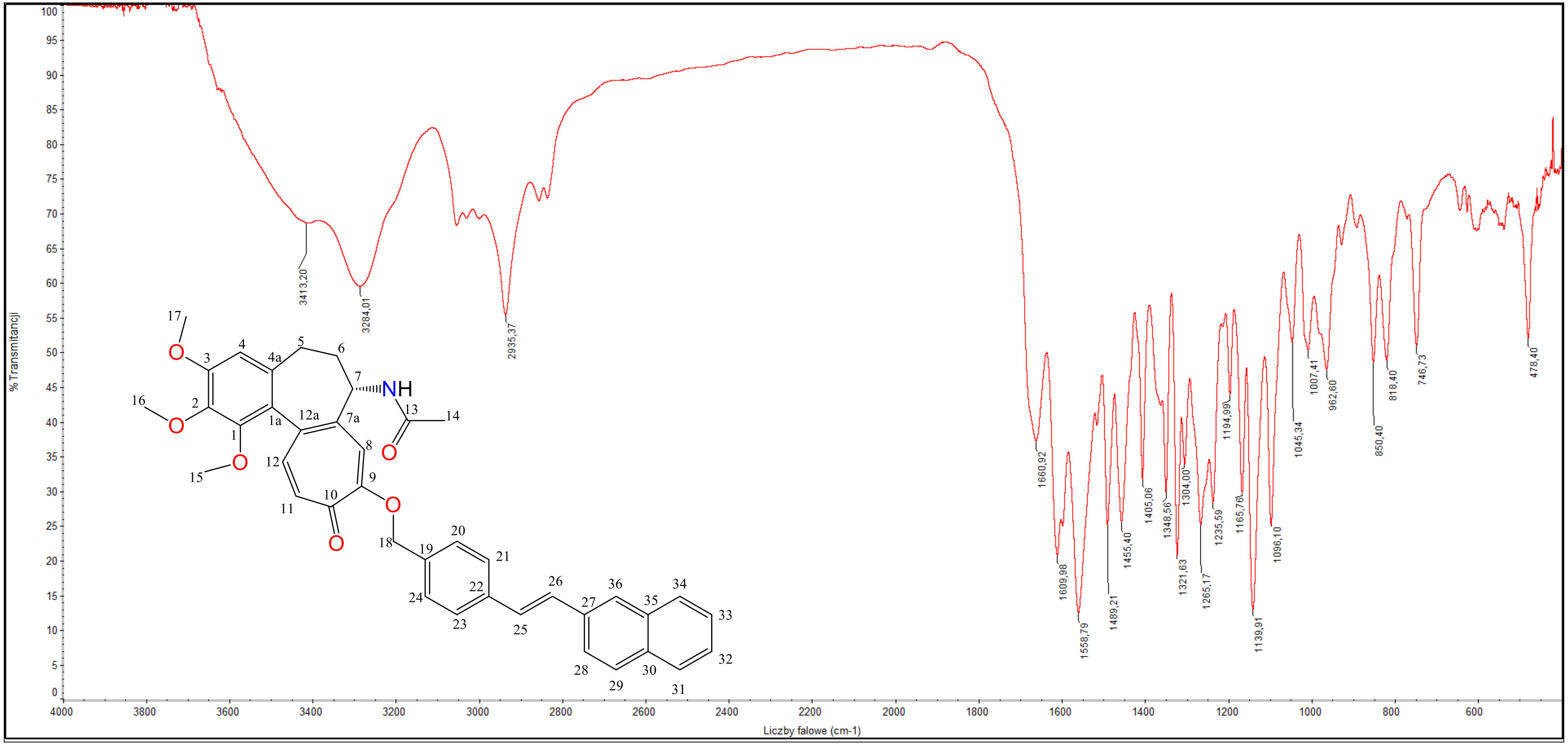

Figure 40S. FT-IR spectrum of compound **7a** (in KBr).

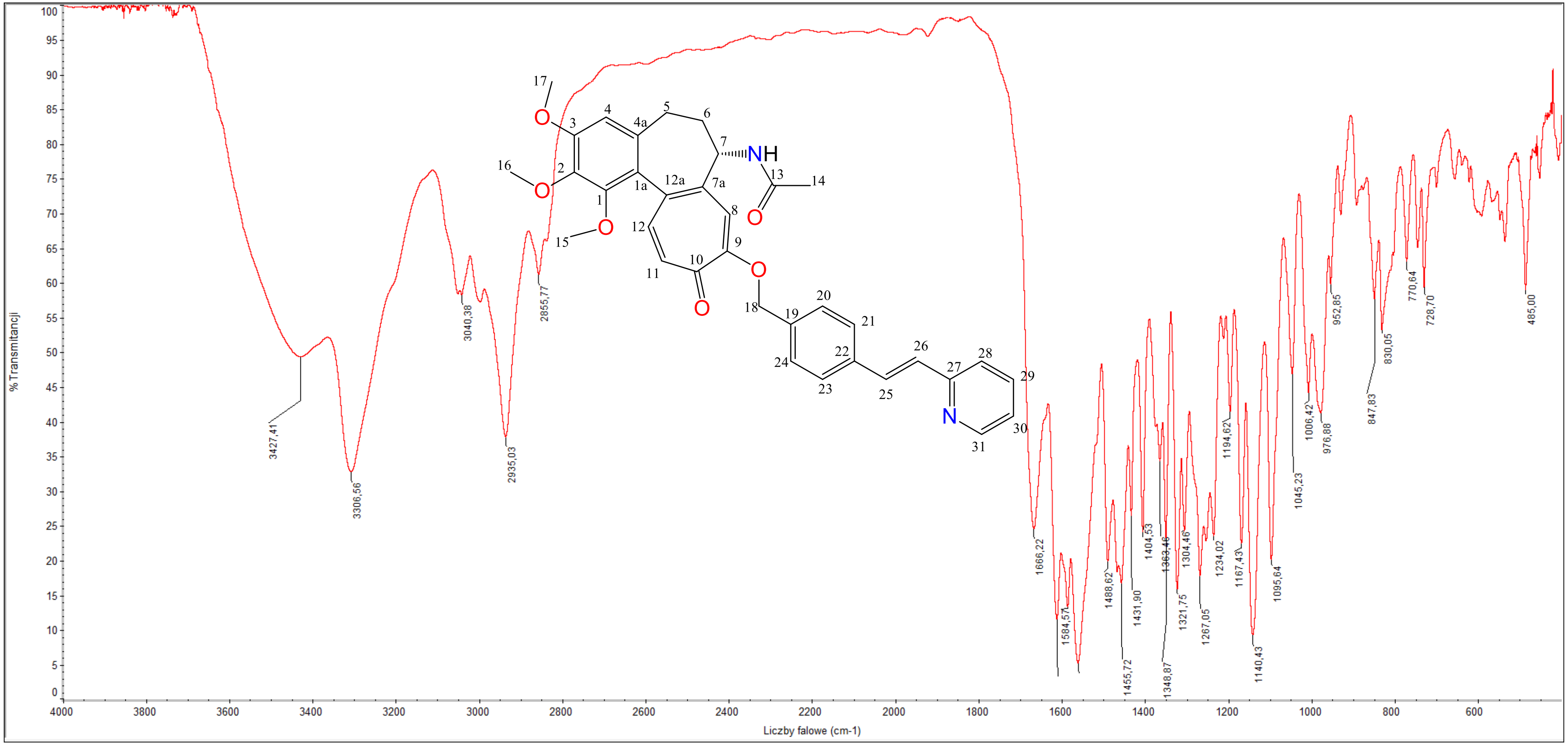

Figure 41S. FT-IR spectrum of compound **7b** (in KBr).

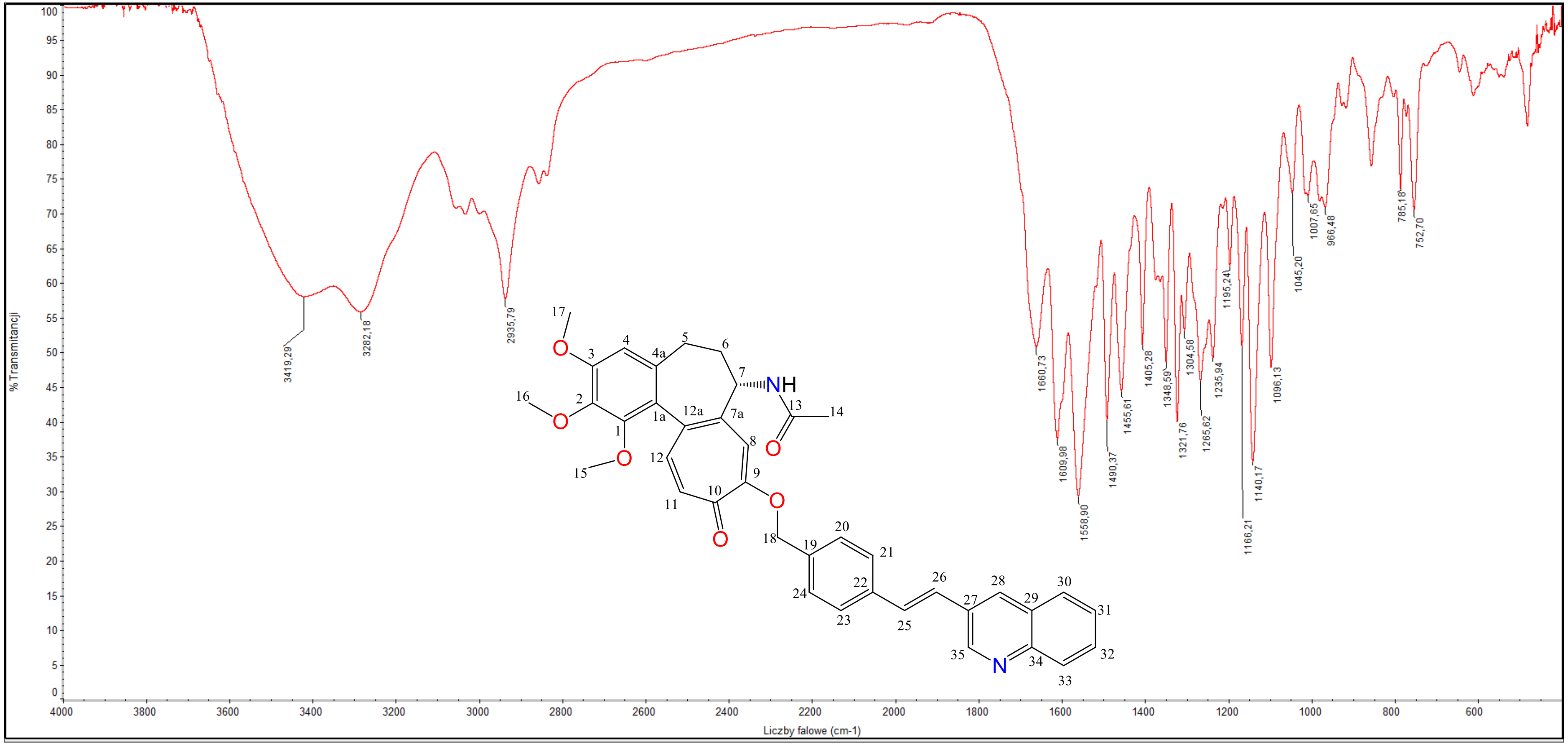

Figure 42S. FT-IR spectrum of compound **7c** (in KBr).

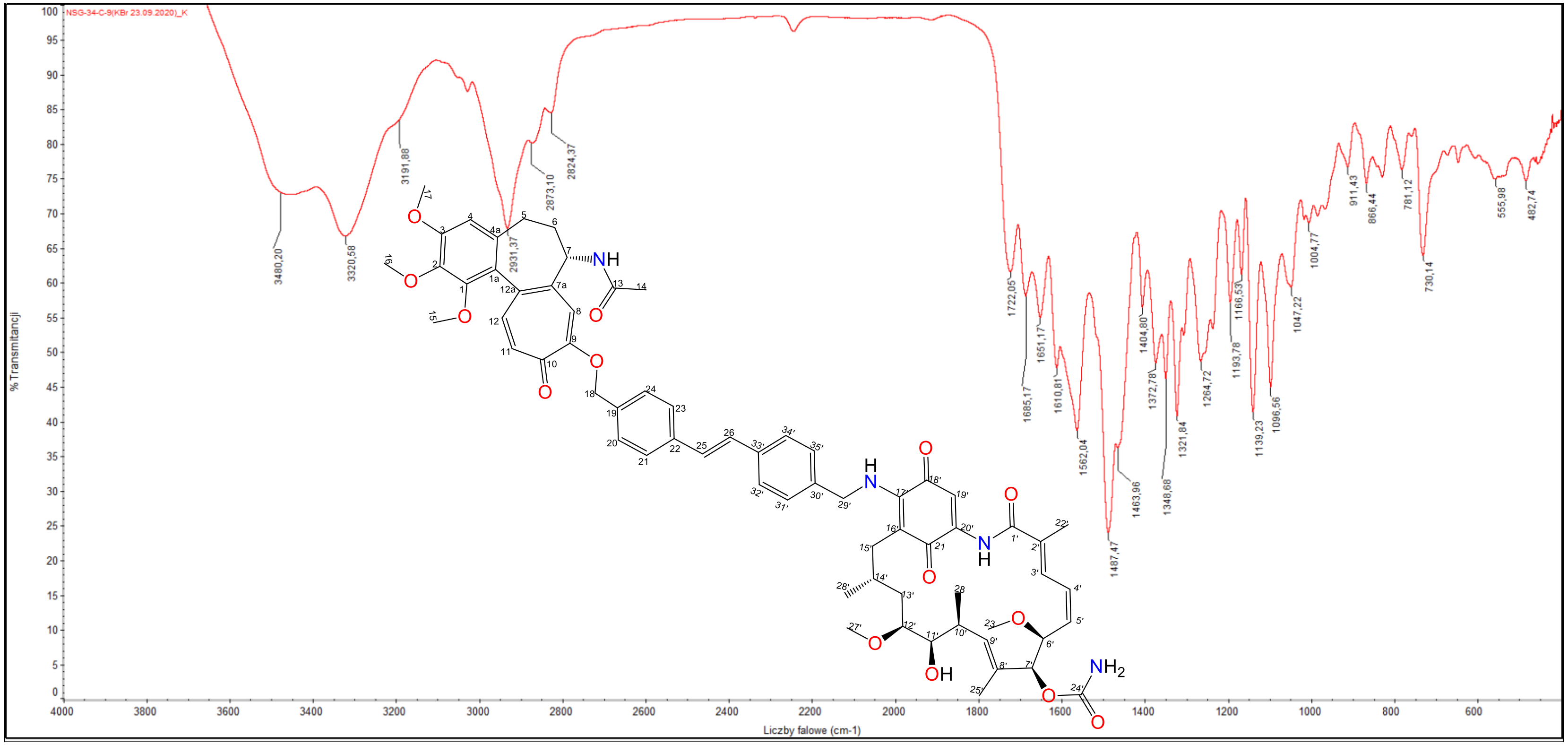

Figure 43S. FT-IR spectrum of compound **7d** (in KBr).

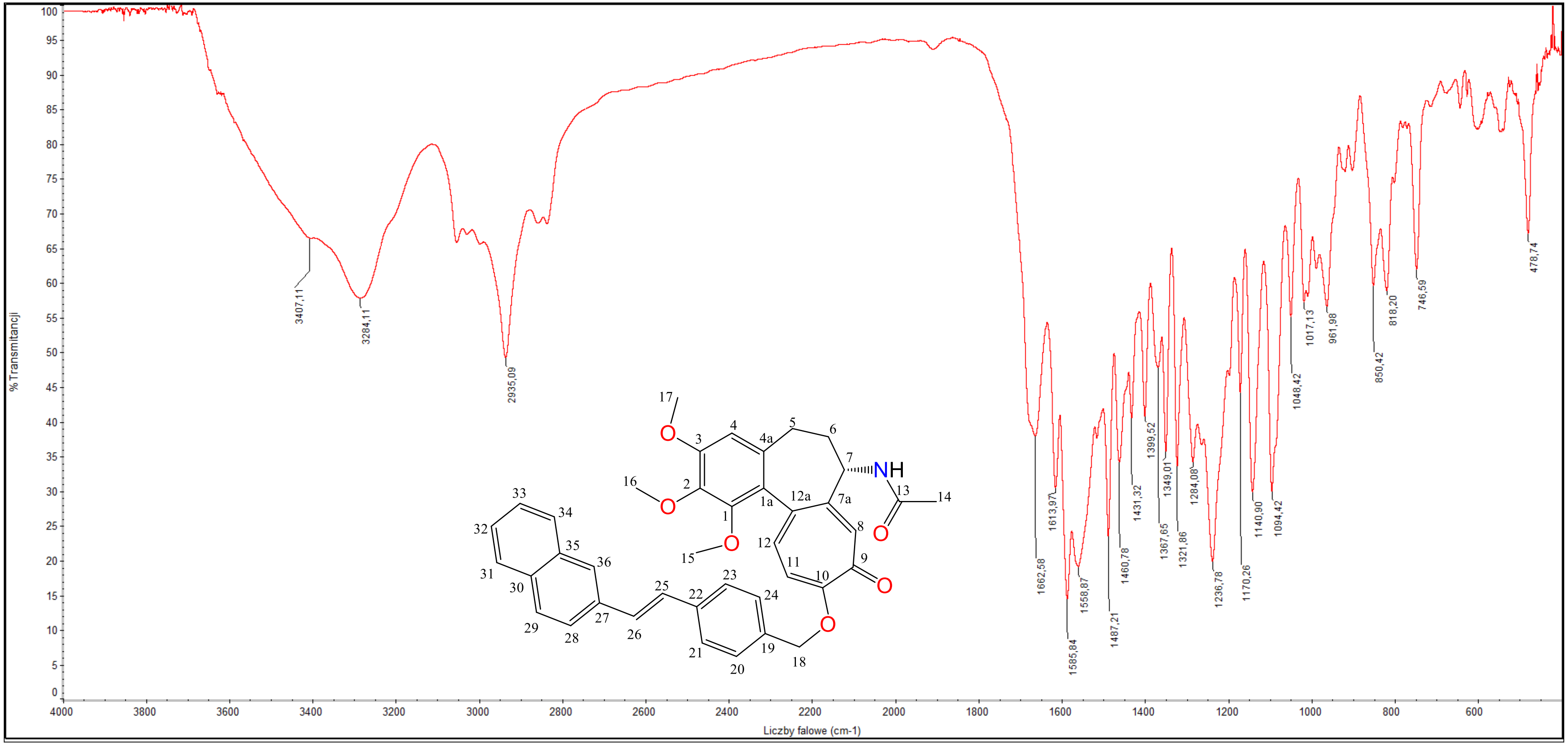

Figure 44S. FT-IR spectrum of compound **8a** (in KBr).

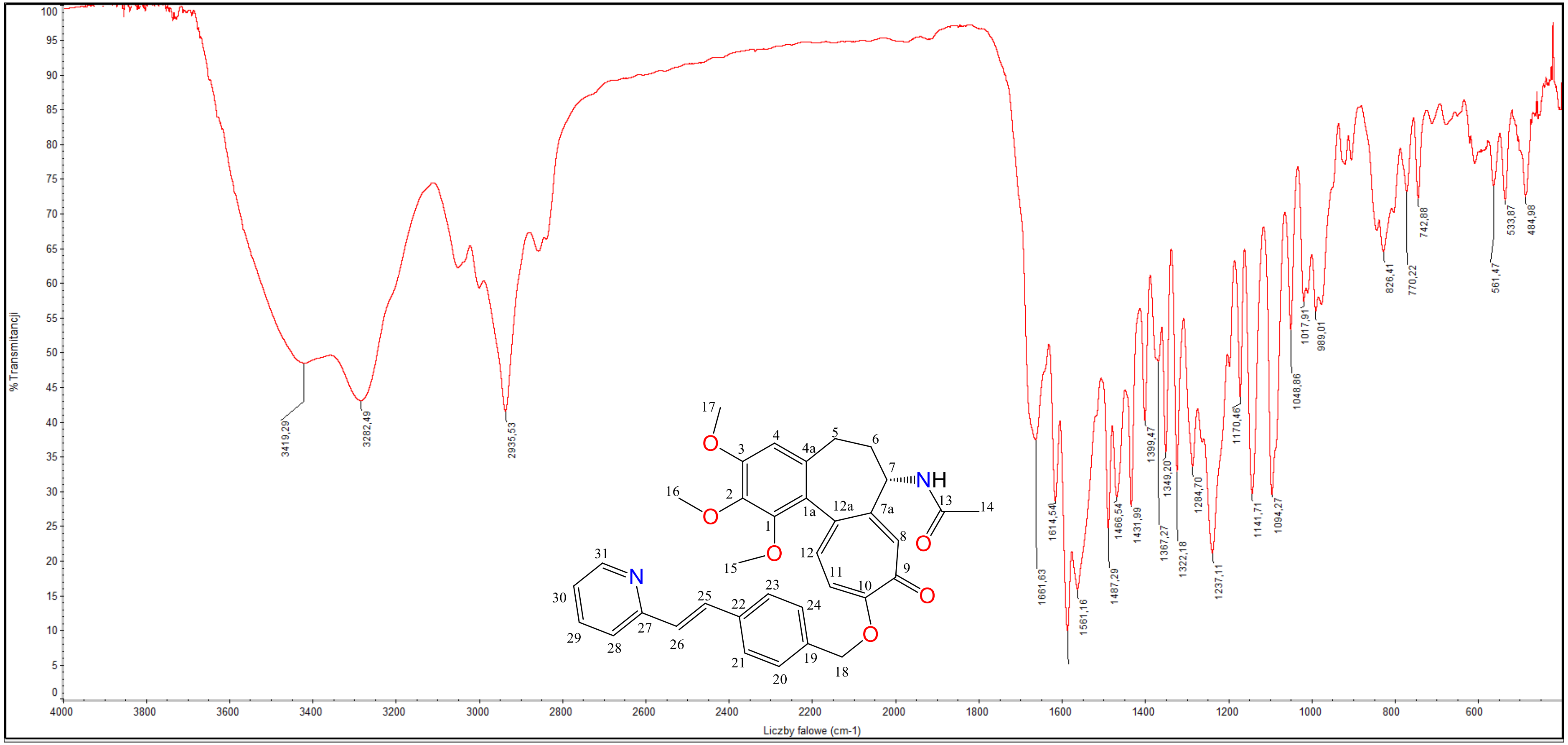

Figure 45S. FT-IR spectrum of compound **8b** (in KBr).

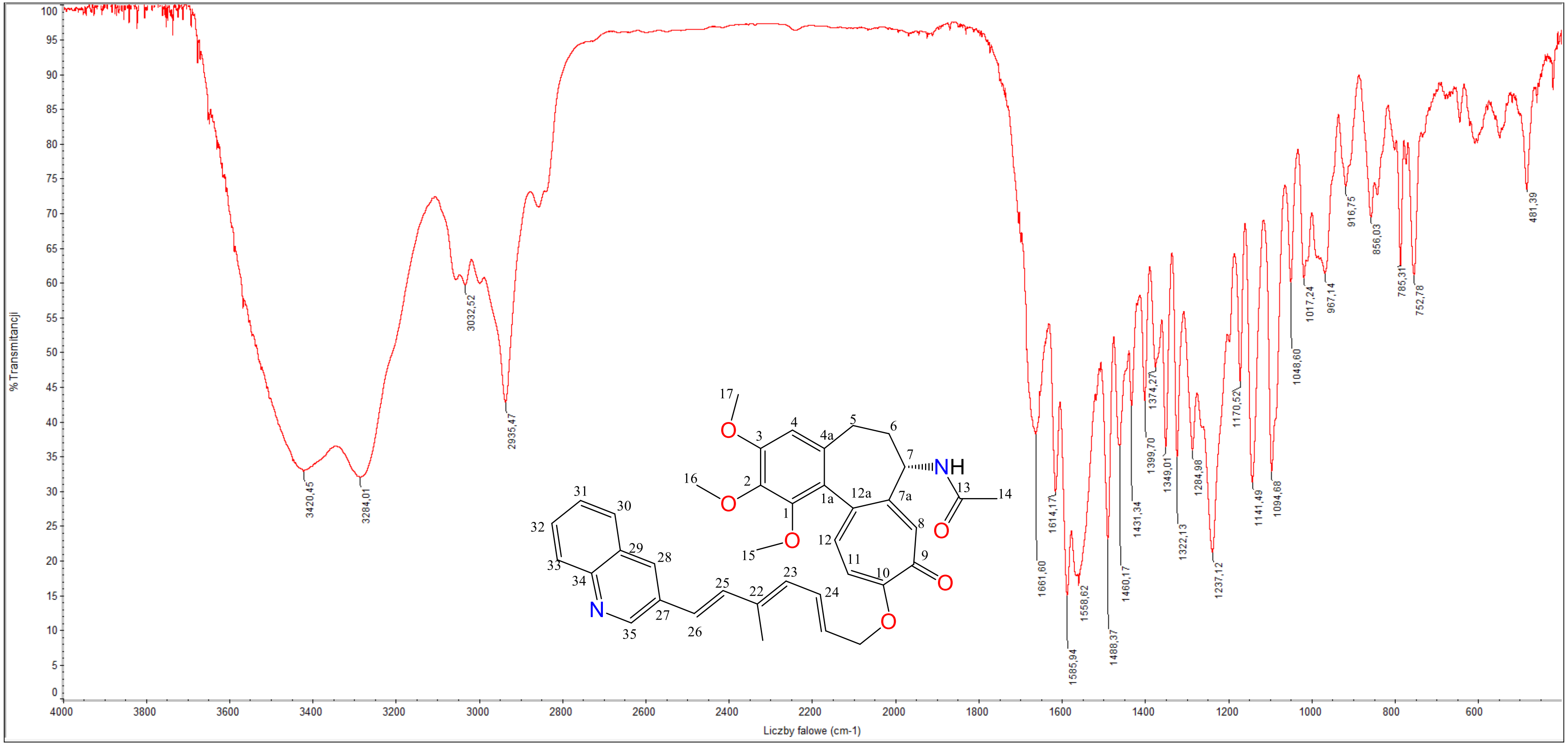

Figure 46S. FT-IR spectrum of compound **8c** (in KBr).

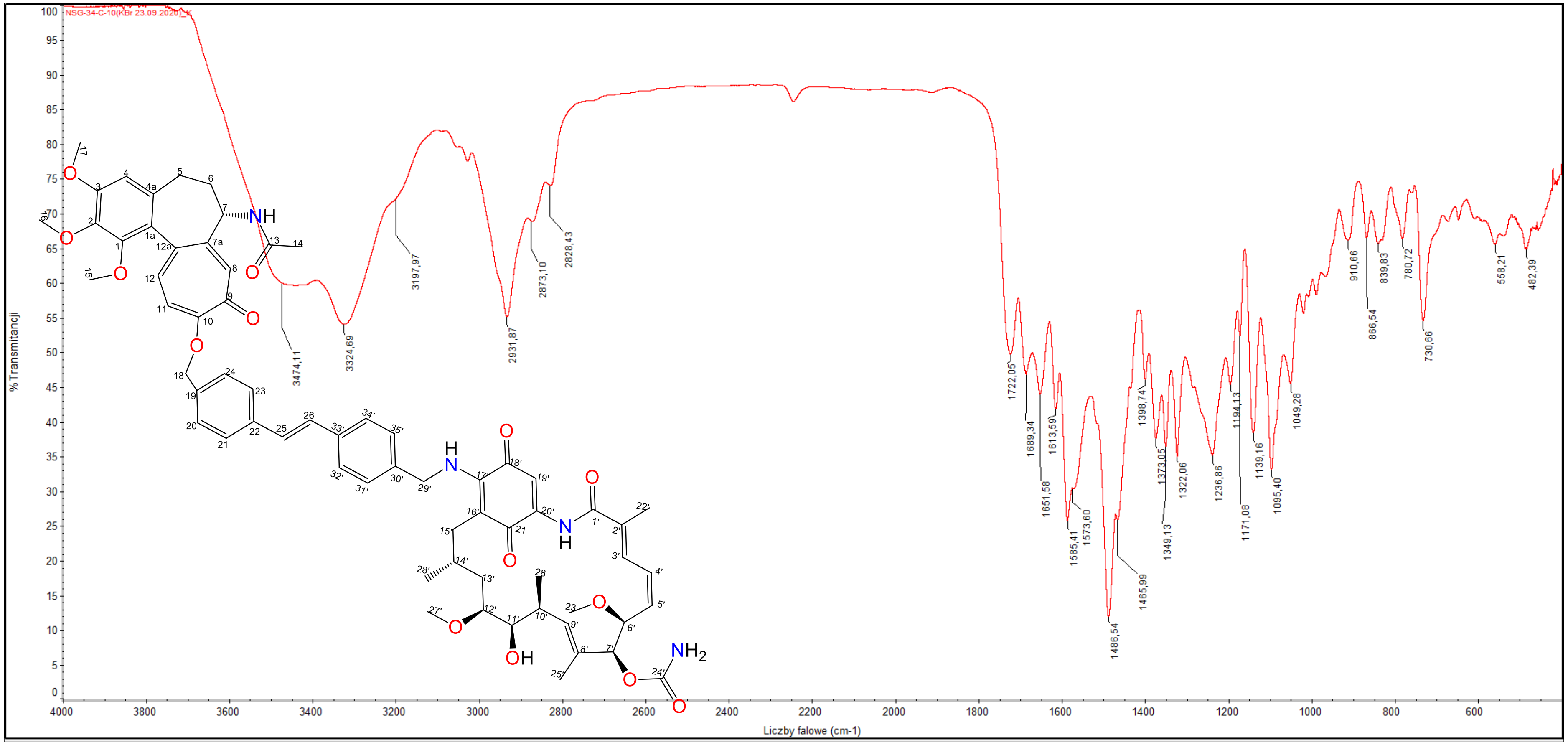

Figure 47S. FT-IR spectrum of compound **8d** (in KBr).

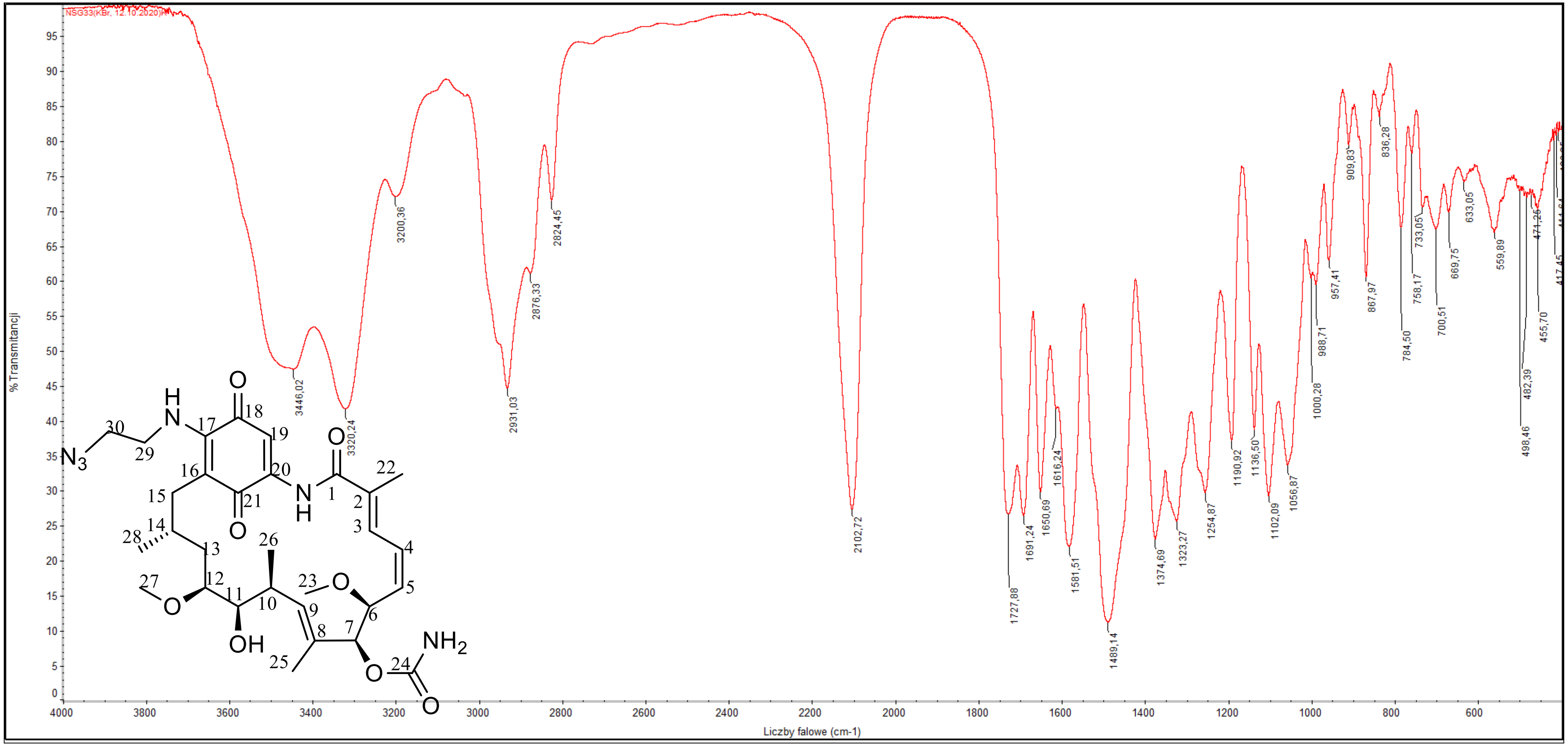

Figure 48S. FT-IR spectrum of compound **9** (in KBr).

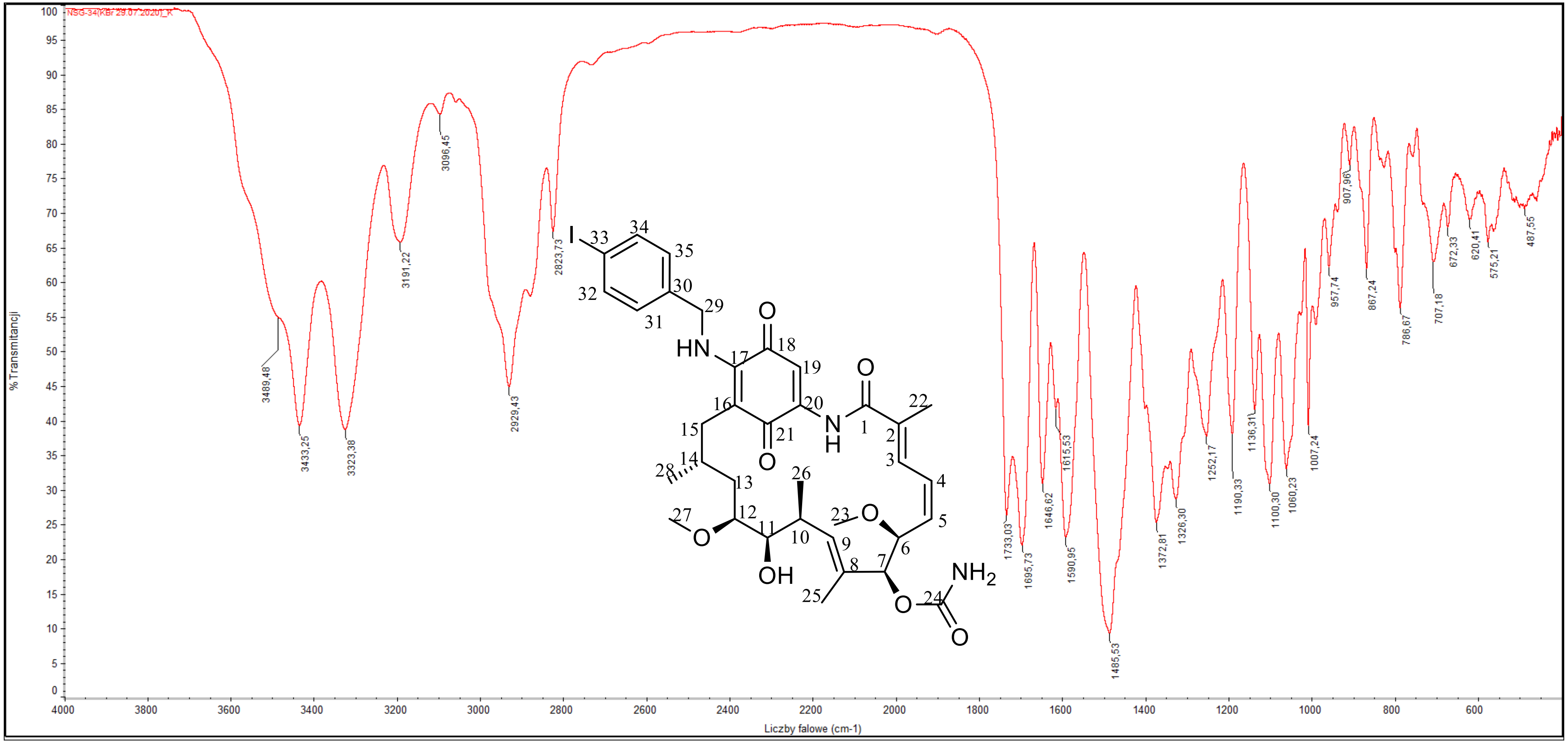

Figure 49S. FT-IR spectrum of compound **10** (in KBr).

NMR spectra

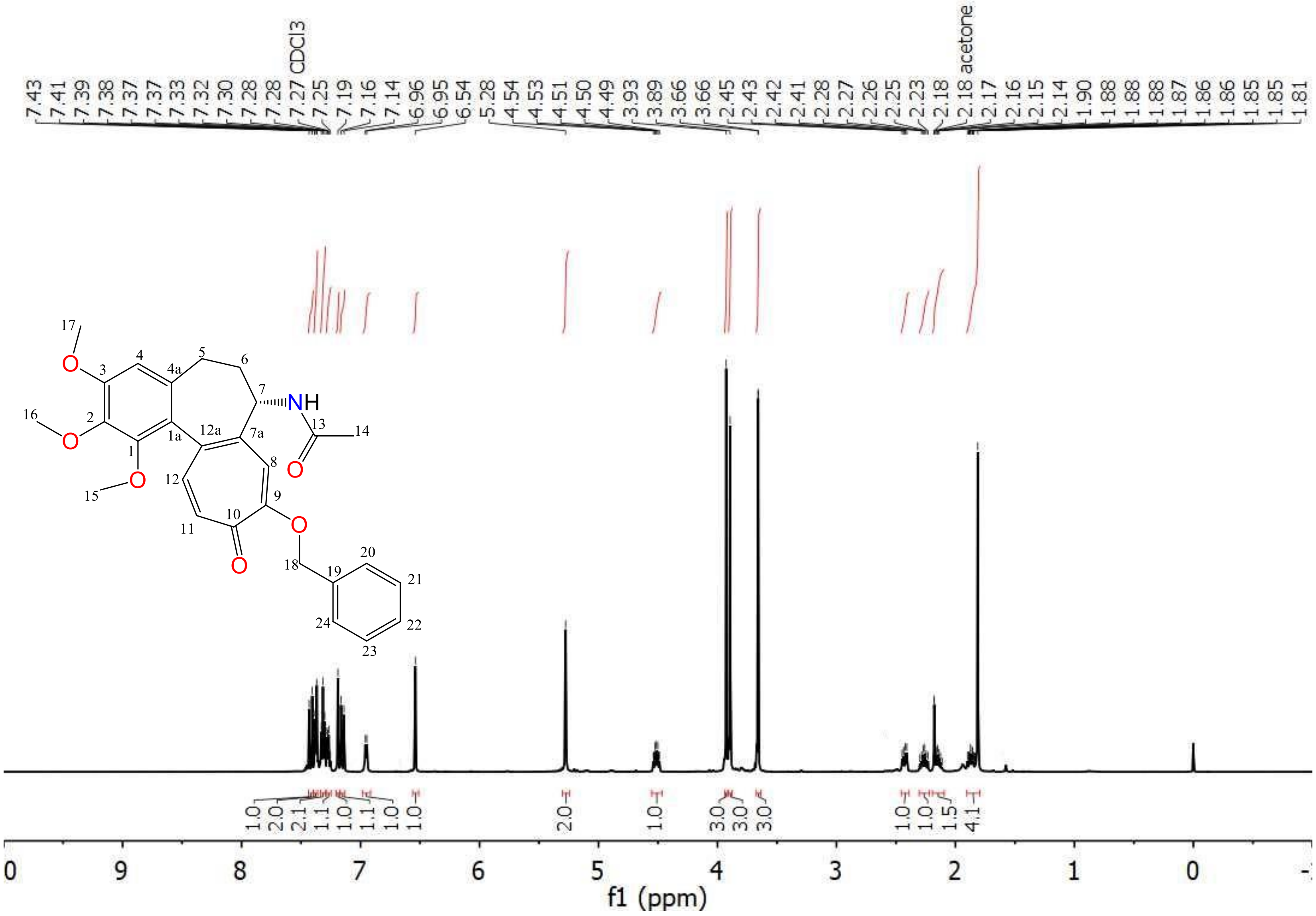

Figure 50S. ^{1}H NMR spectrum of compound **3a** in $CDCl_3$.

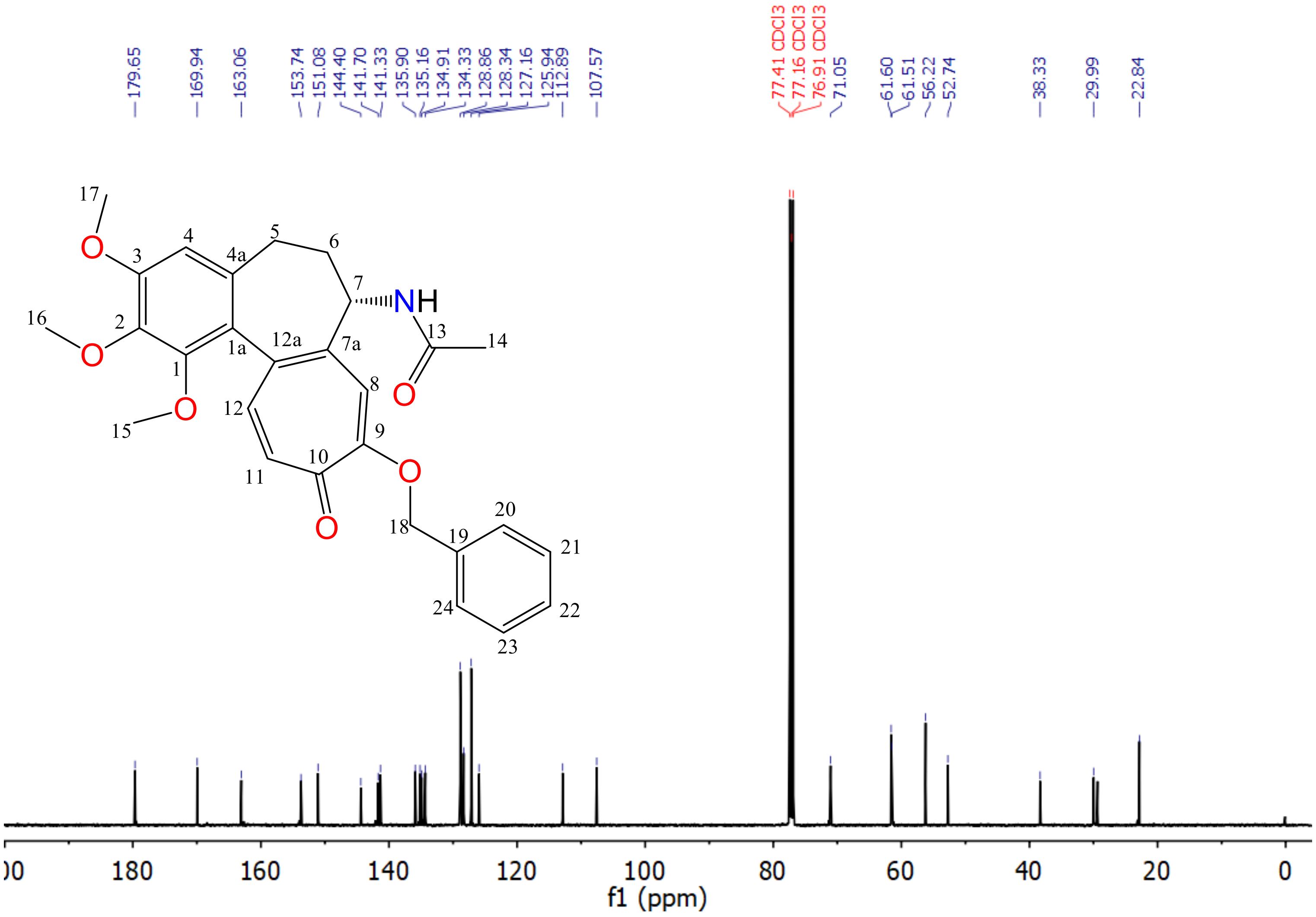

Figure 51S. ^{13}C NMR spectrum of compound **3a** in $CDCl_3$.

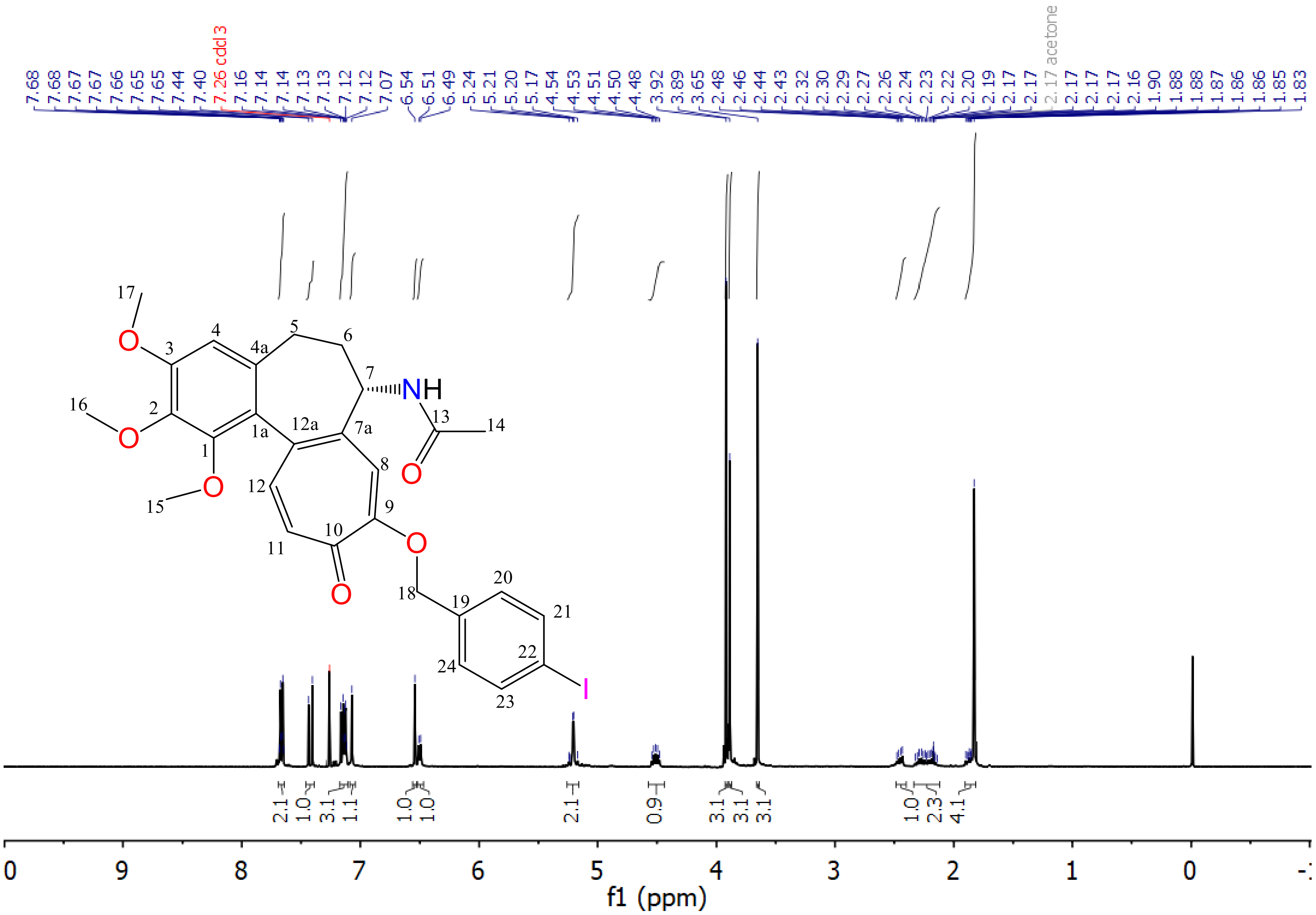

Figure 52S. ^{1}H NMR spectrum of compound **3b** in $CDCl_3$.

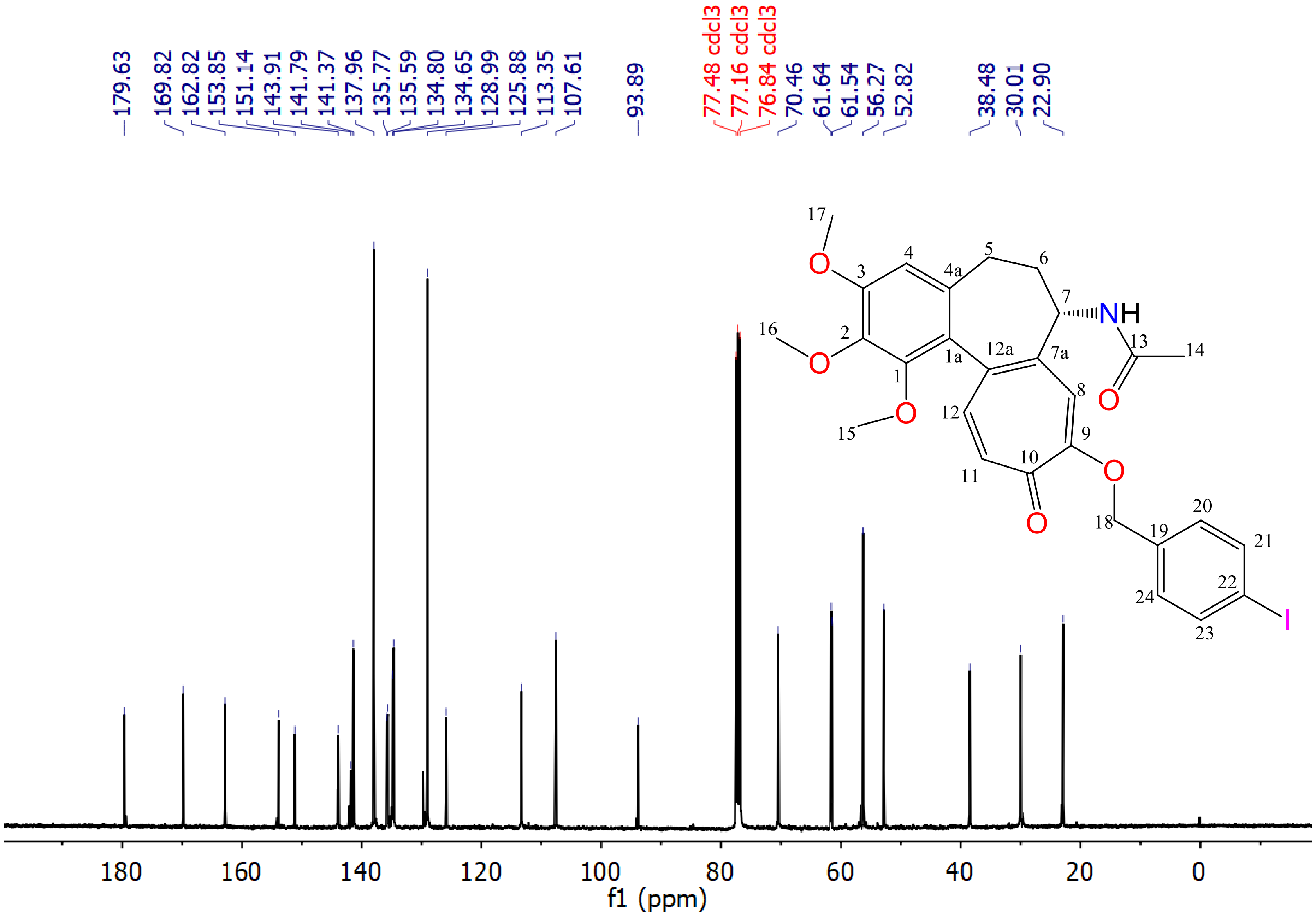

Figure 53S. ^{13}C NMR spectrum of compound **3b** in $CDCl_3$

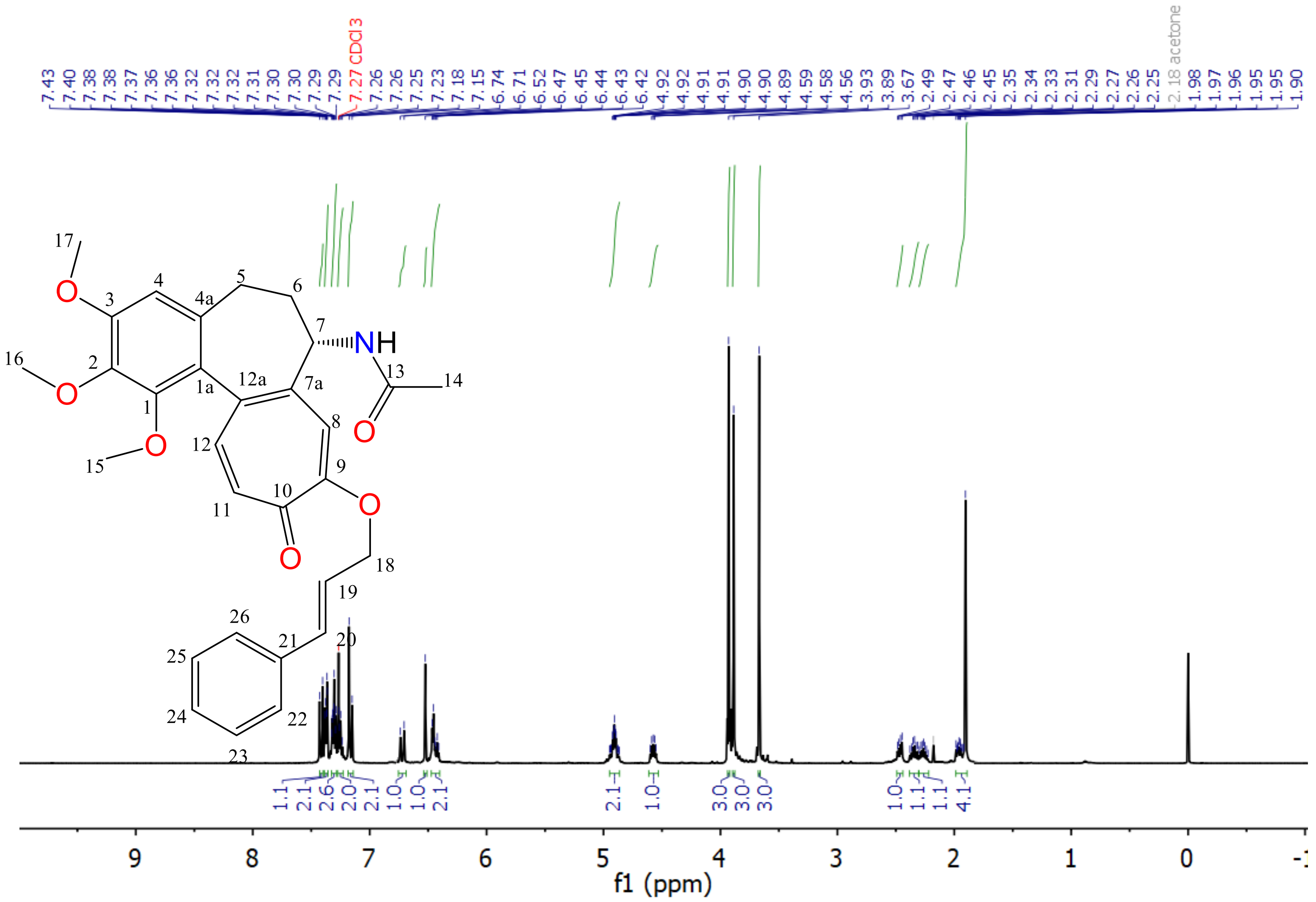

Figure 54S. ^{1}H NMR spectrum of compound **3c** in $CDCl_3$.

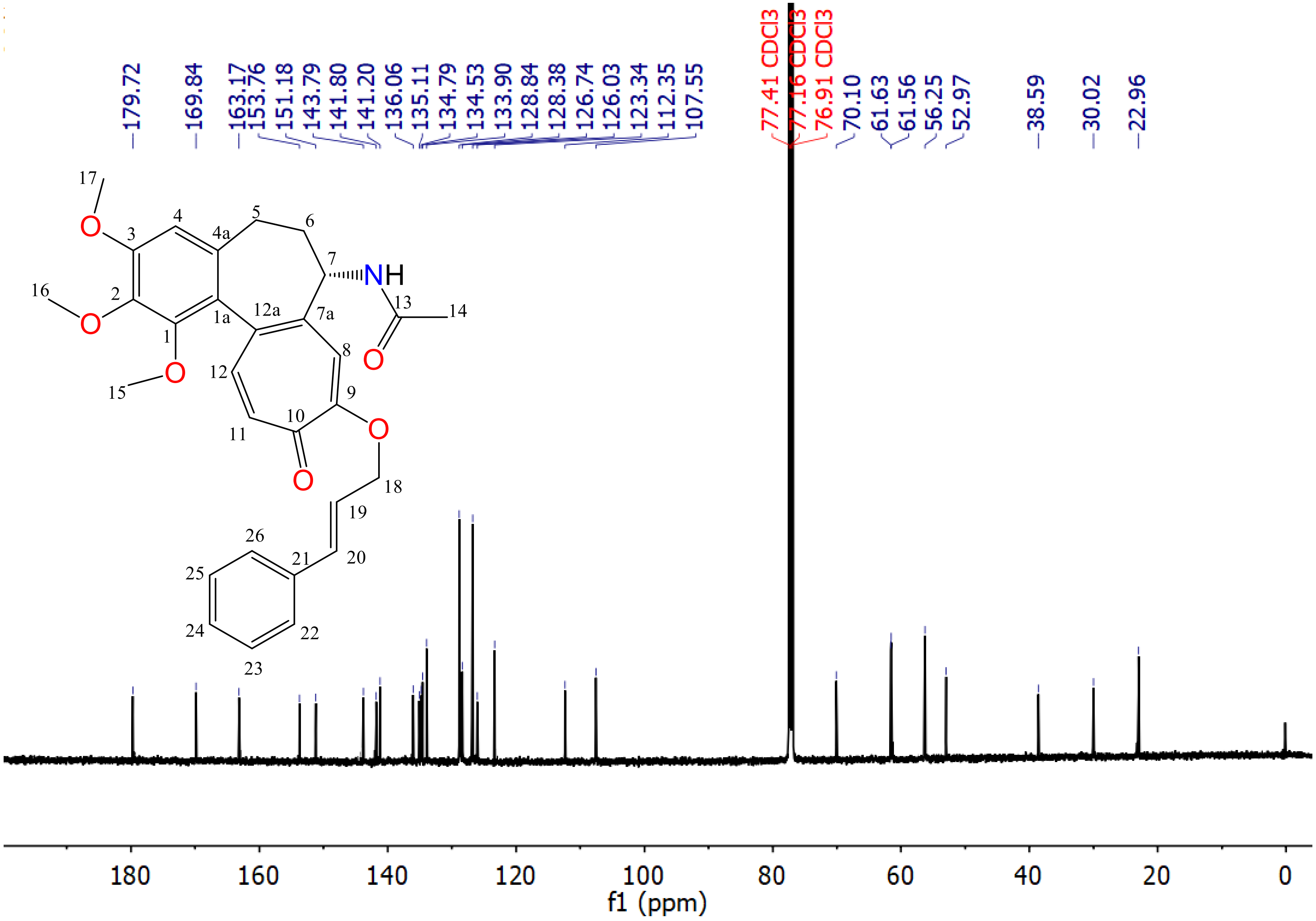

Figure 55S. ^{1}H NMR spectrum of compound **3c** in $CDCl_3$.

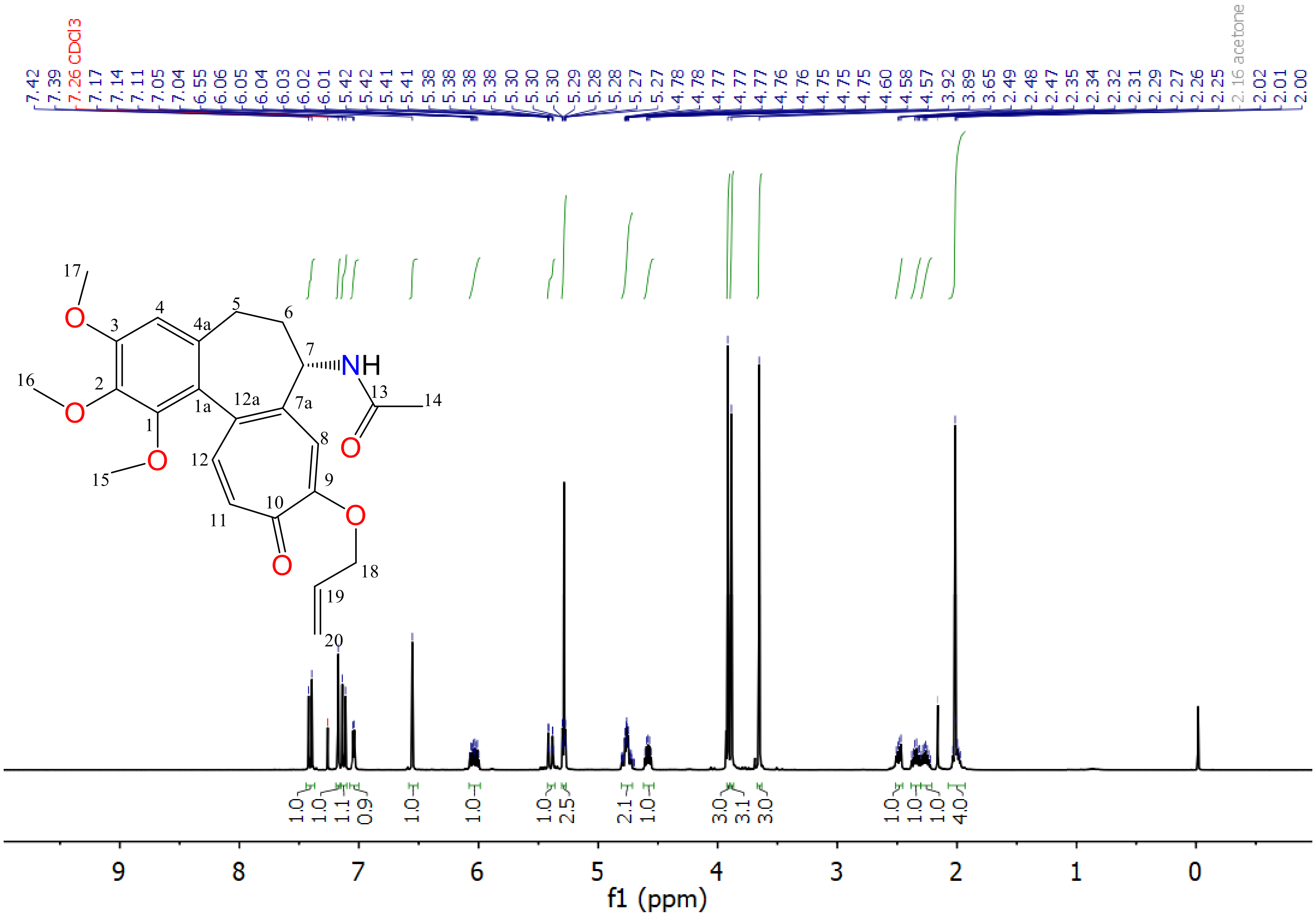

Figure 56S. ^{1}H NMR spectrum of compound **3d** in $CDCl_3$.

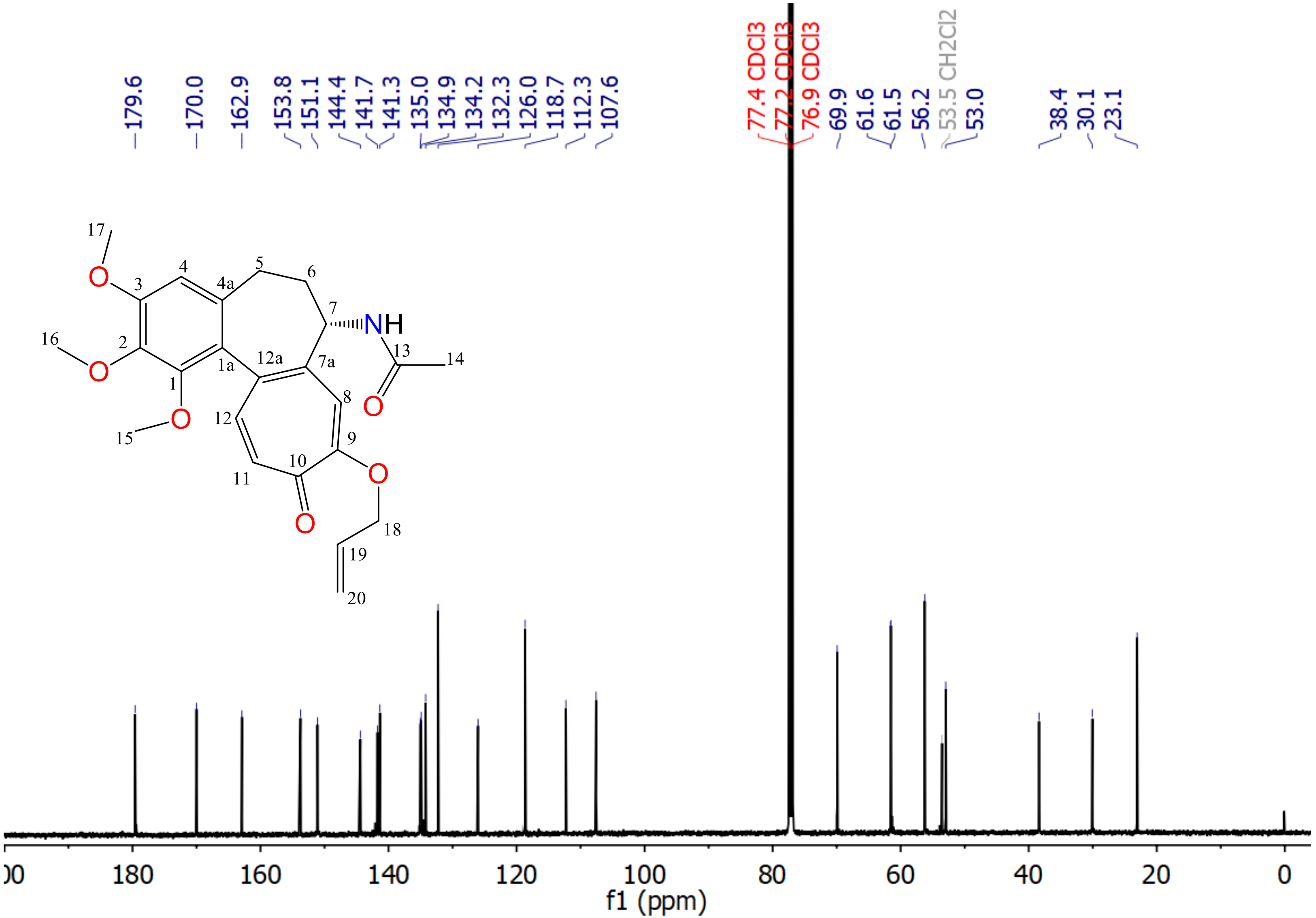

Figure 57S. ^{1}H NMR spectrum of compound **3d** in $CDCl_3$.

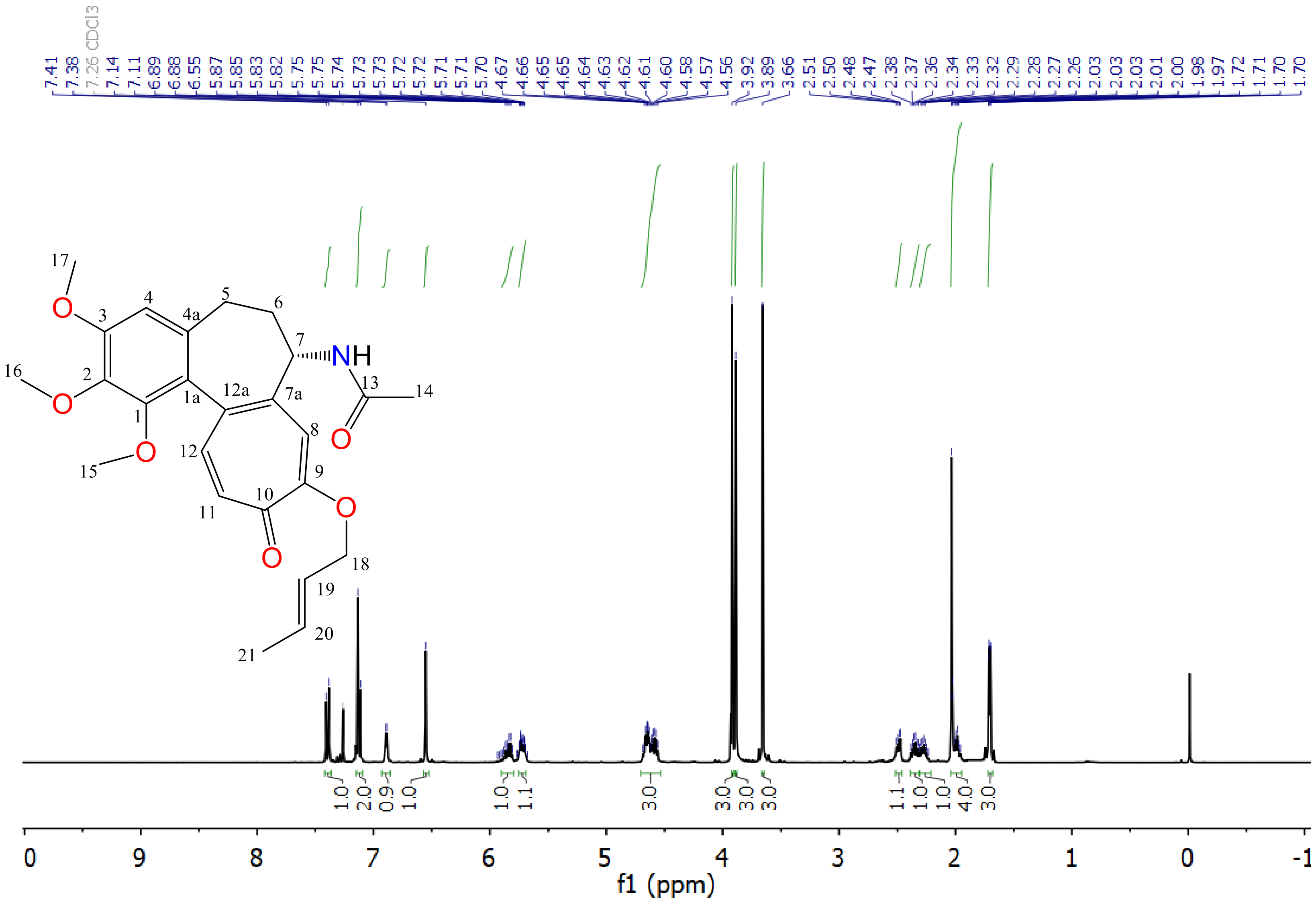

Figure 58S. ^{1}H NMR spectrum of compound **3e** in $CDCl_3$.

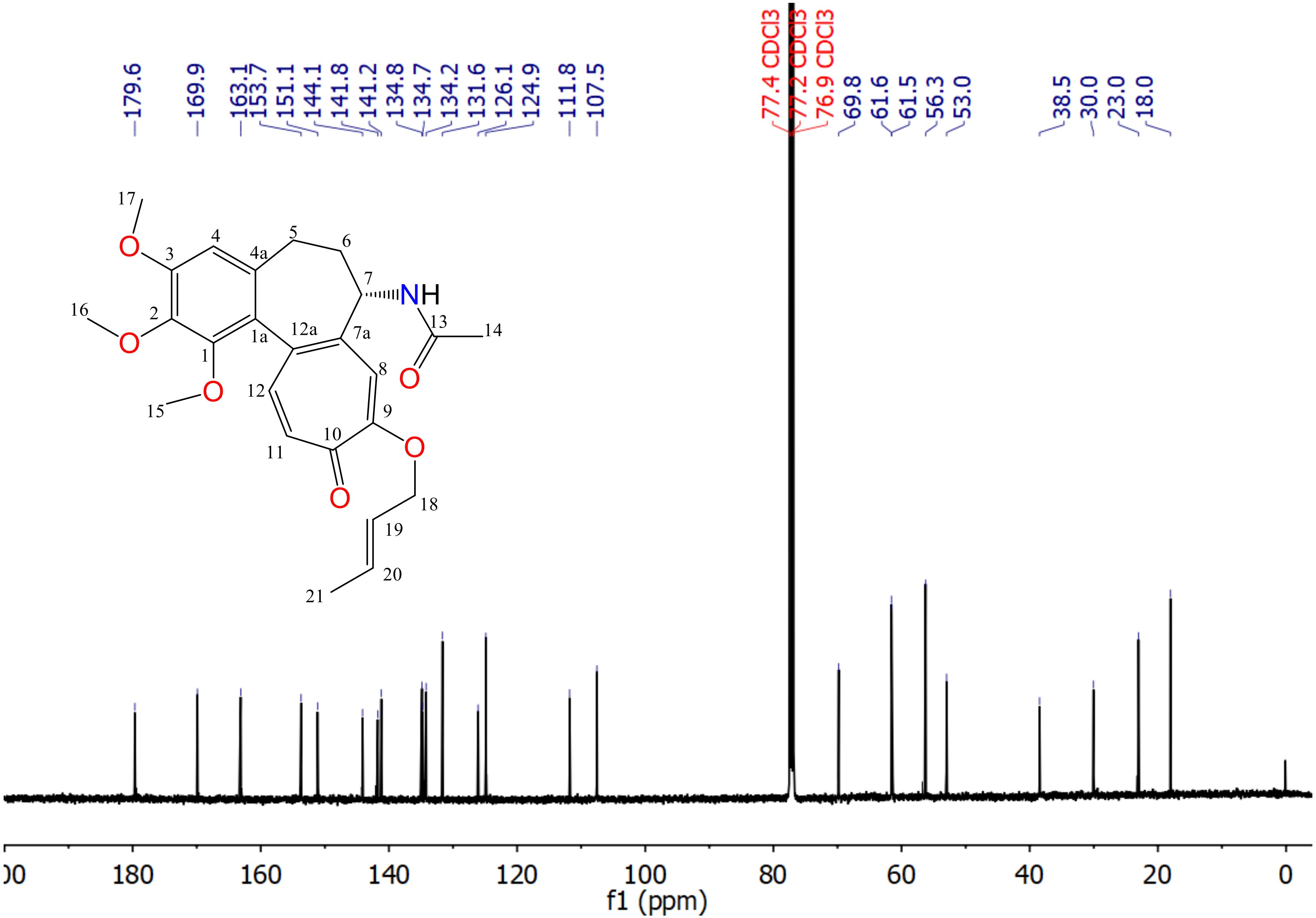

Figure 59S. ^{13}C NMR spectrum of compound **3e** in $CDCl_3$.

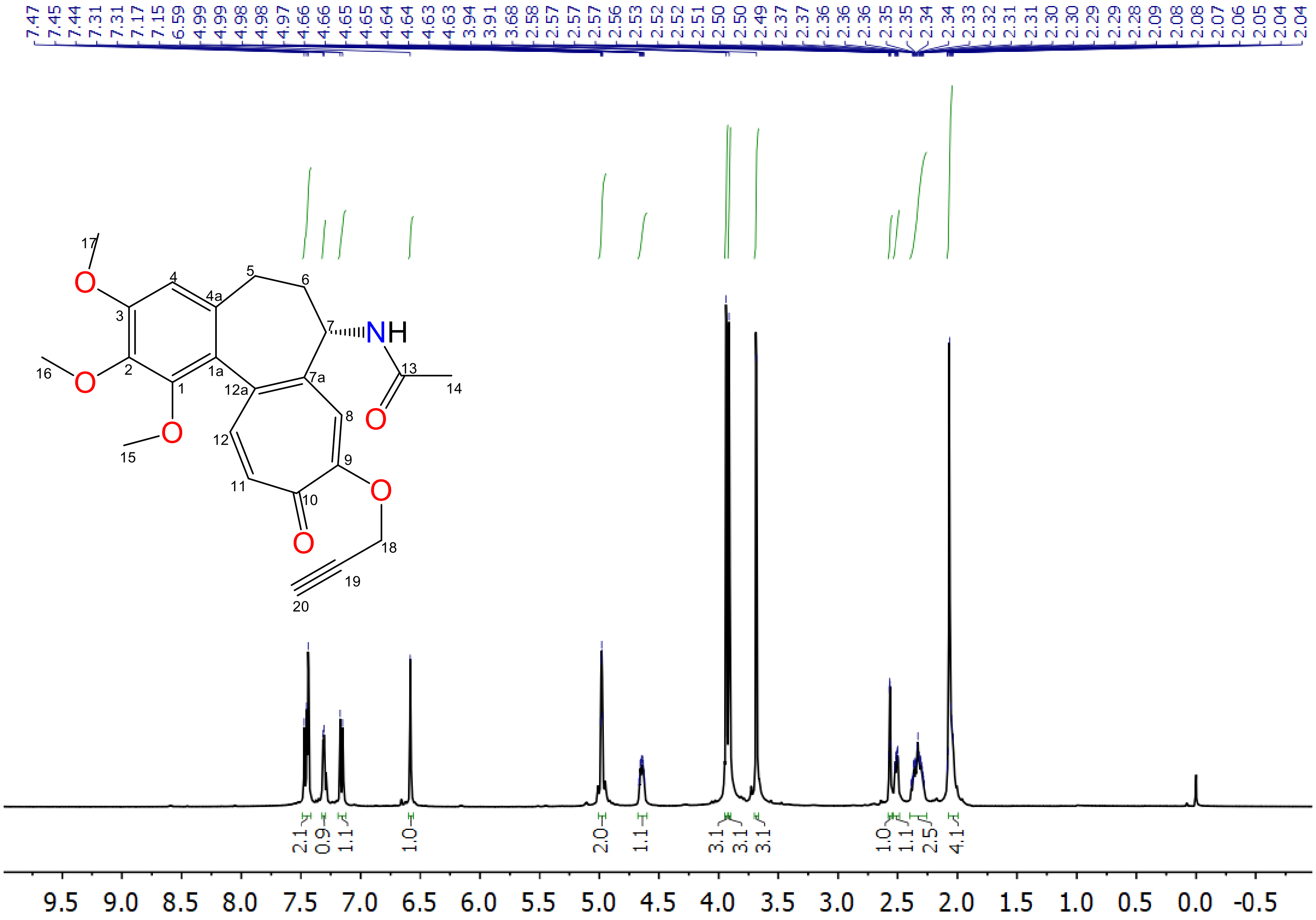

Figure 60S. ^{1}H NMR spectrum of compound **3f** in $CDCl_3$.

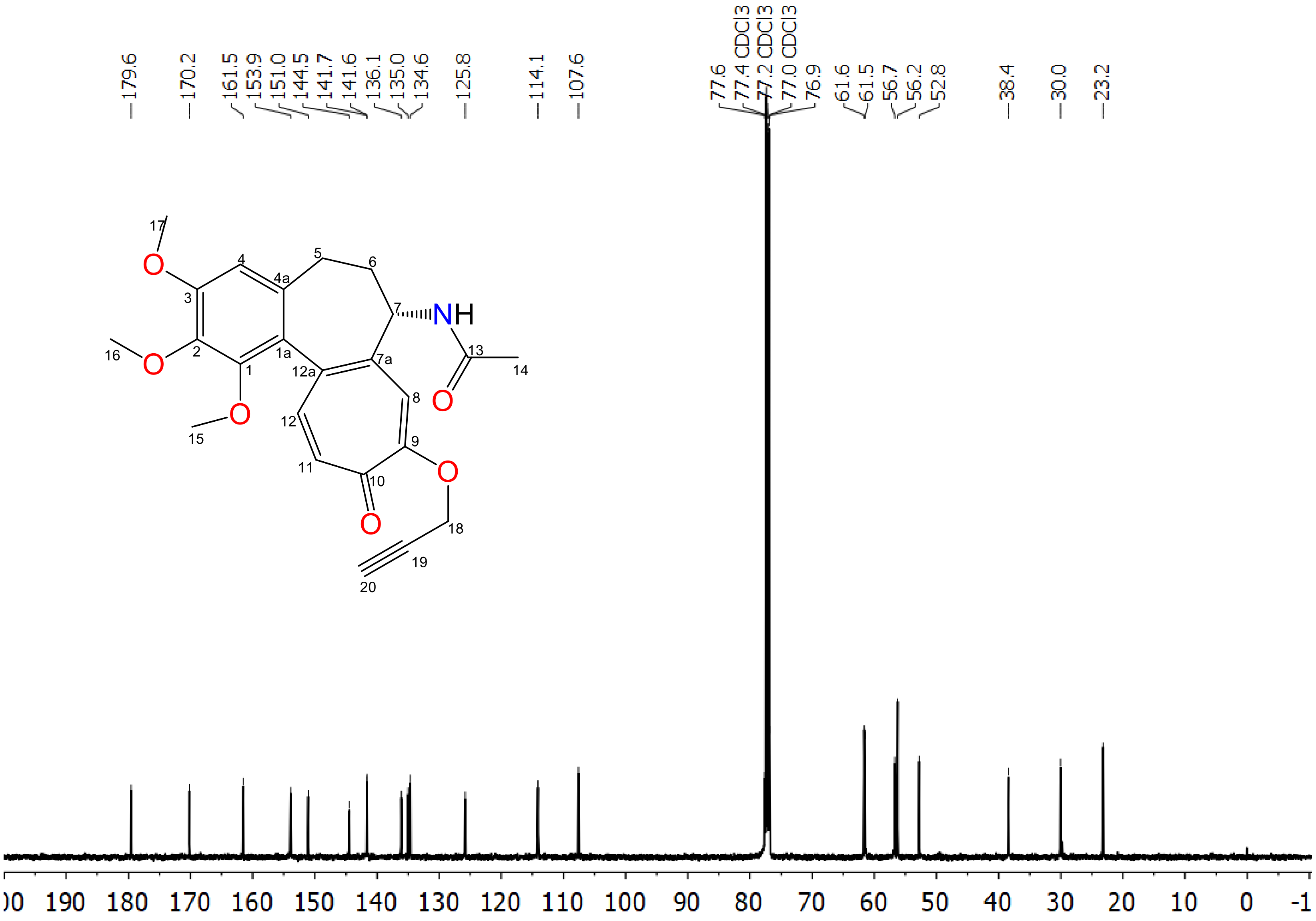

Figure 61S. ^{13}C NMR spectrum of compound **3f** in $CDCl_3$.

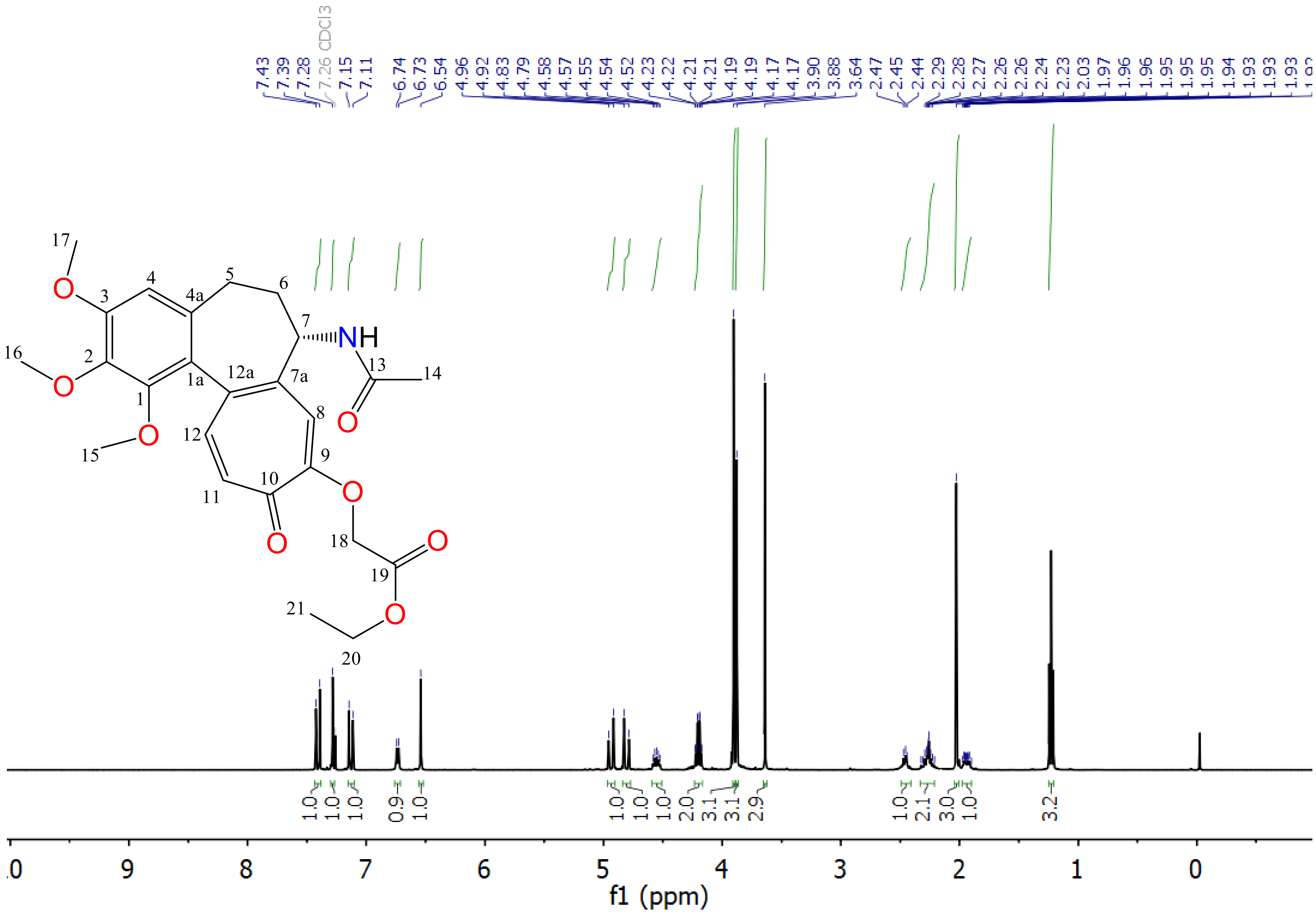

Figure 62S. ^{1}H NMR spectrum of compound **3g** in $CDCl_3$.

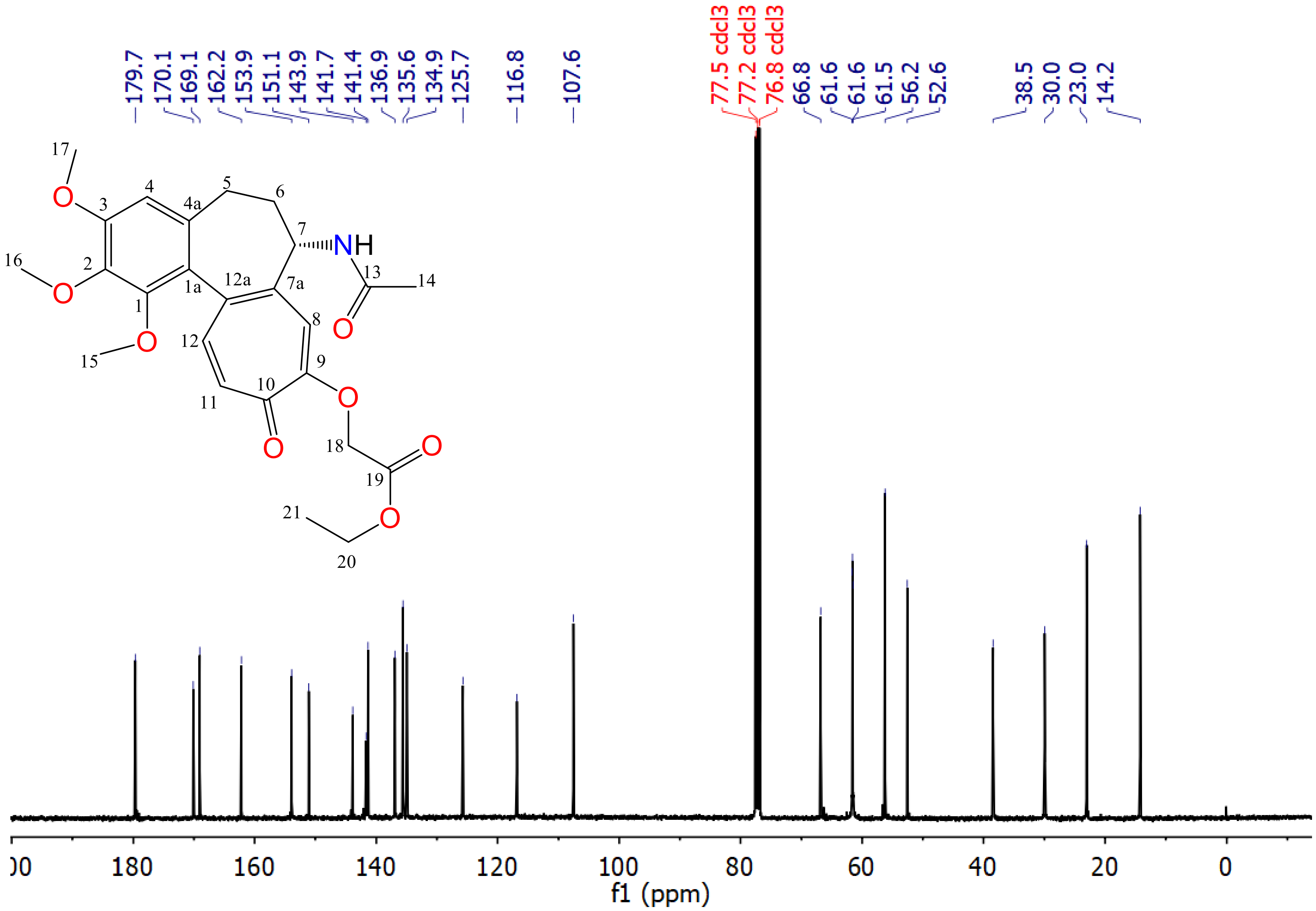

Figure 63S. ^{13}C NMR spectrum of compound **3g** in $CDCl_3$.

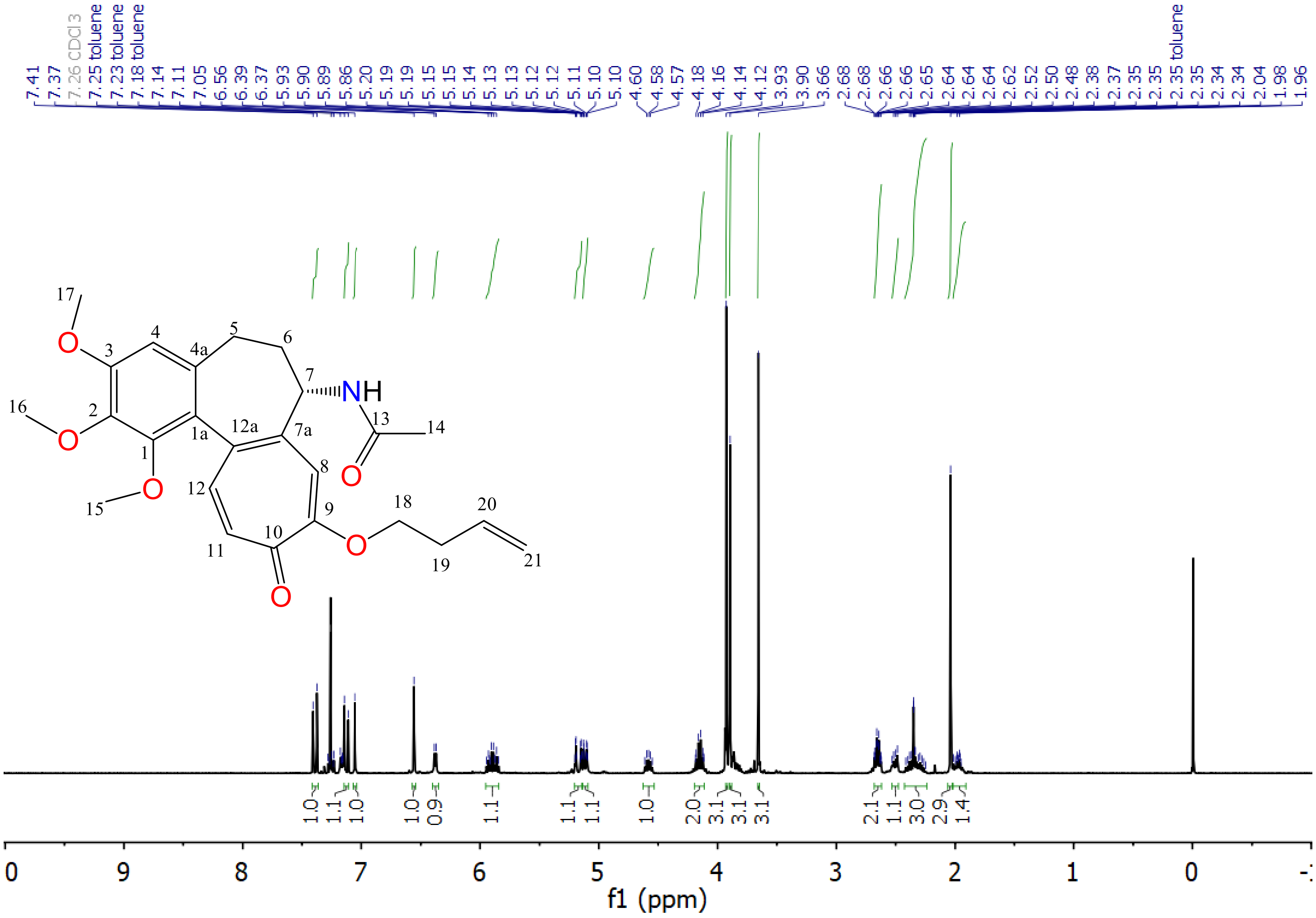

Figure 64S. ^{1}H NMR spectrum of compound **3h** in $CDCl_3$.

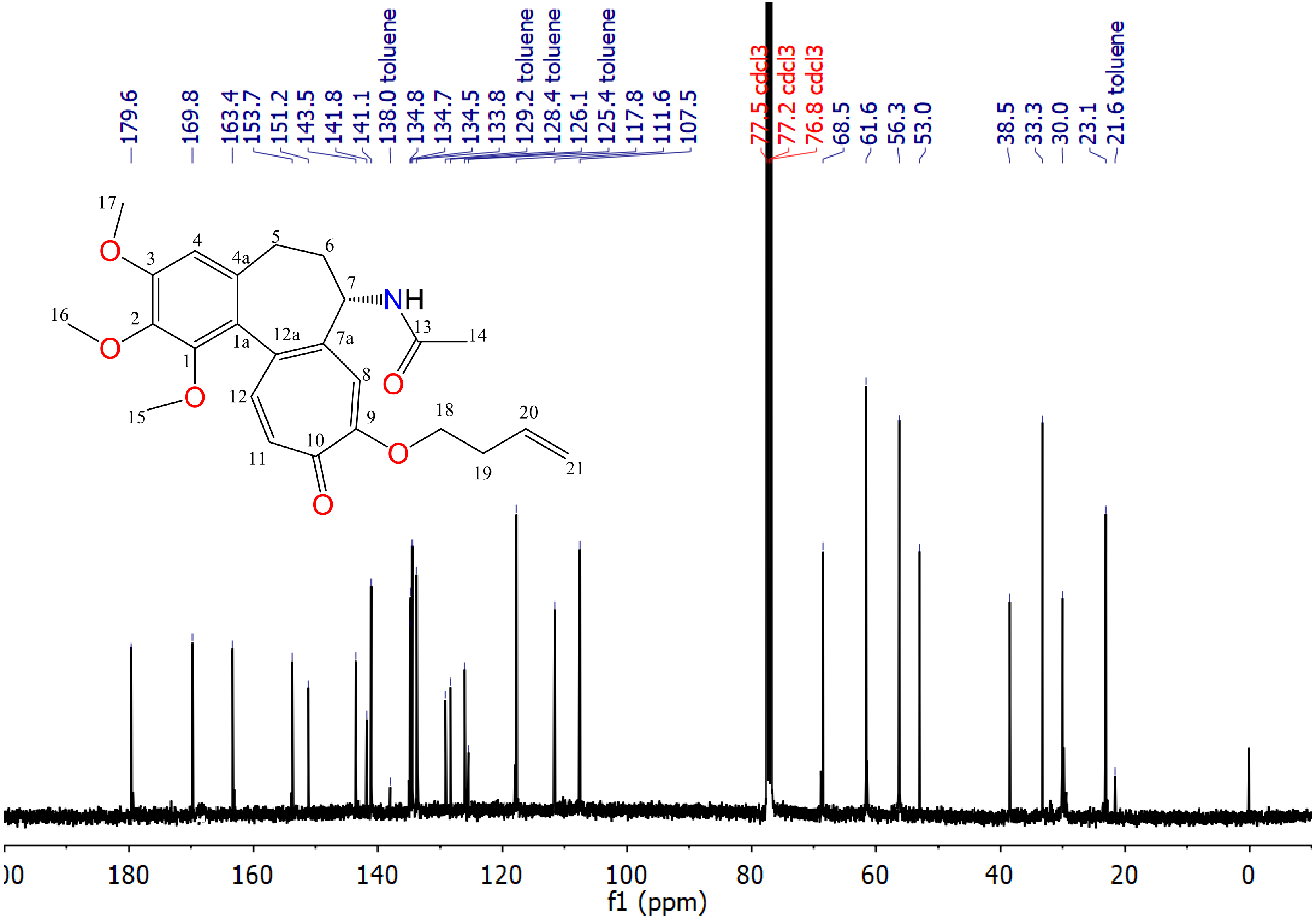

Figure 65S. ^{13}C NMR spectrum of compound **3h** in $CDCl_3$.

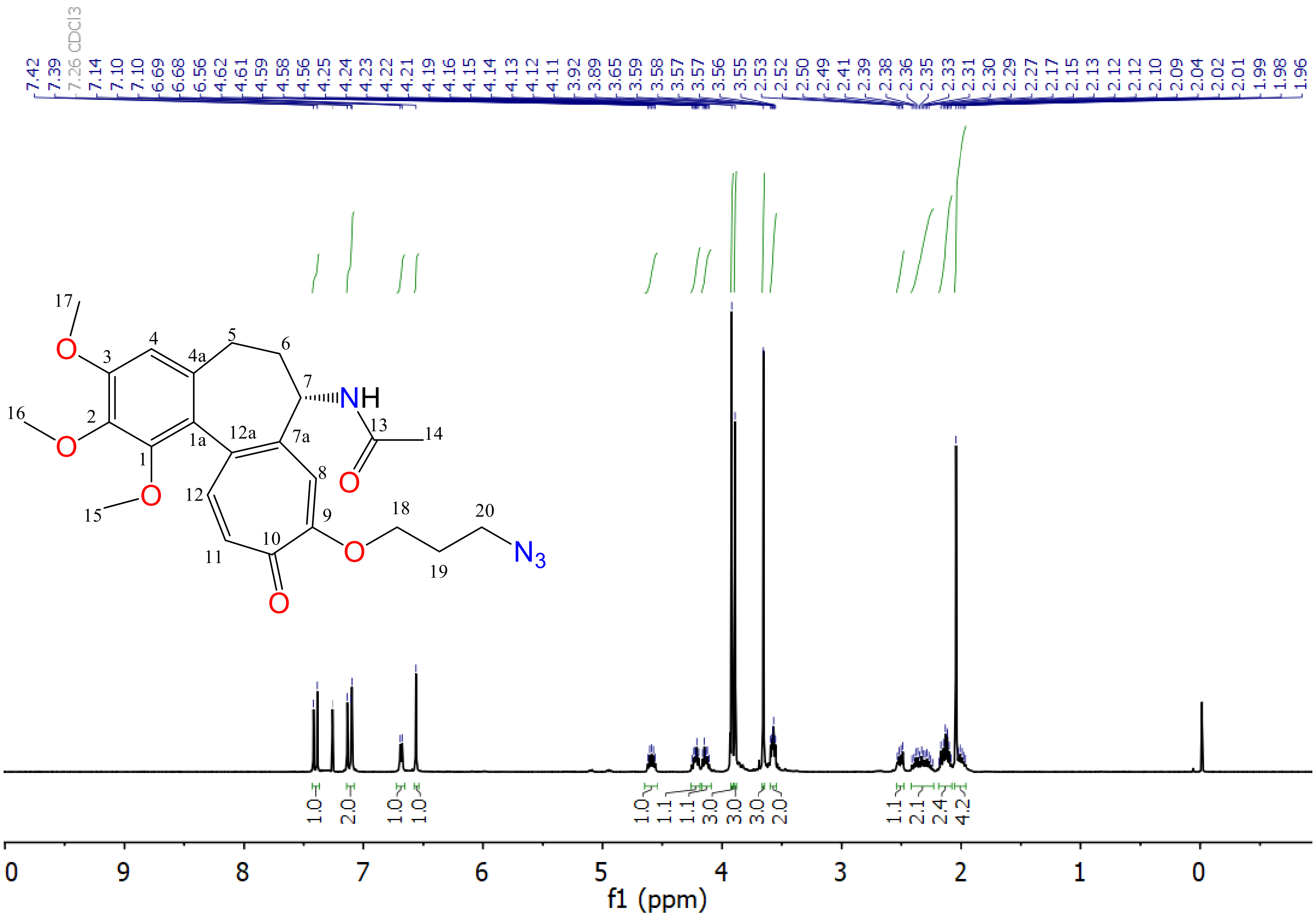

Figure 66S. ^{1}H NMR spectrum of compound **3i** in $CDCl_3$.

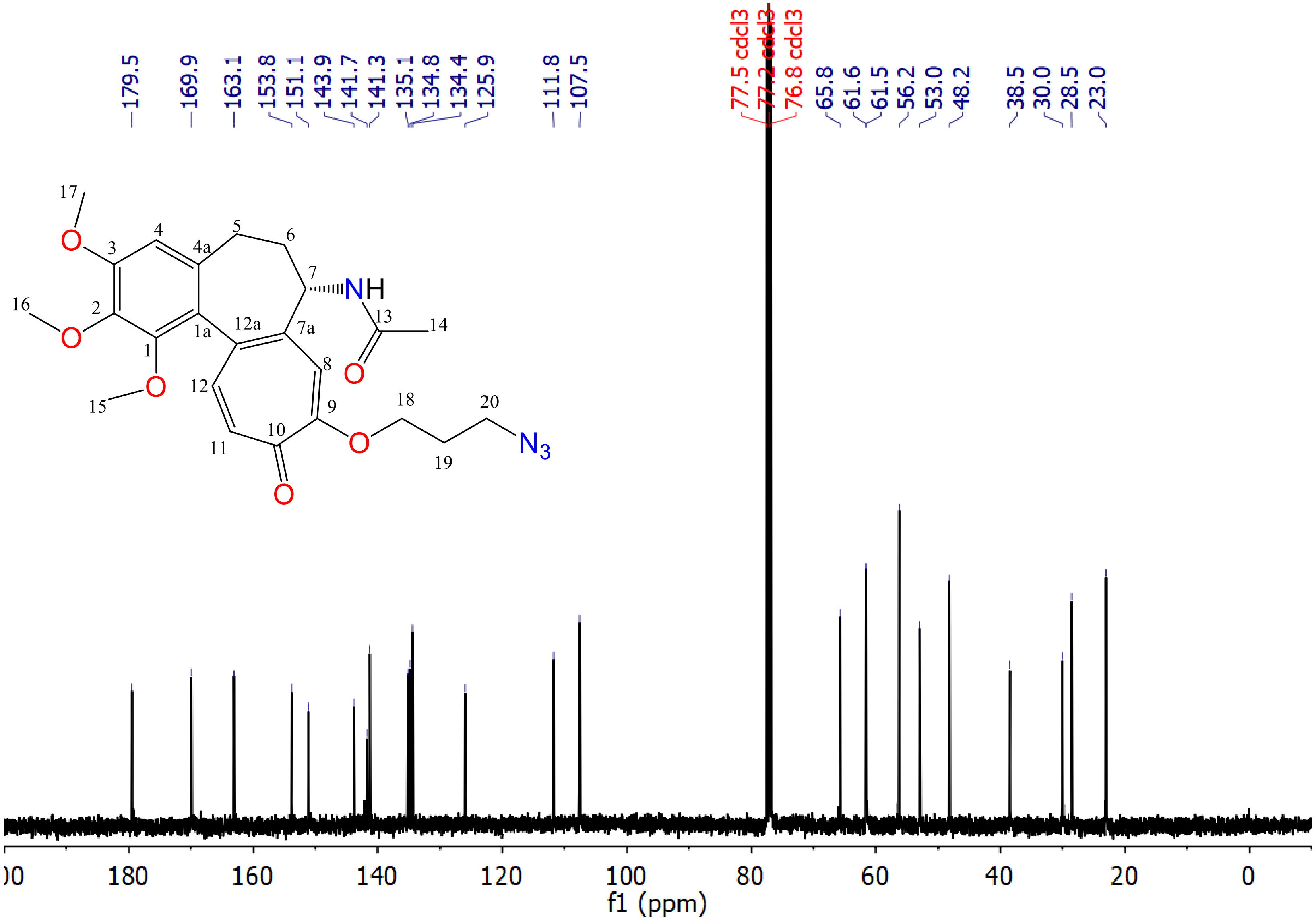

Figure 67S. ^{13}C NMR spectrum of compound **3i** in $CDCl_3$.

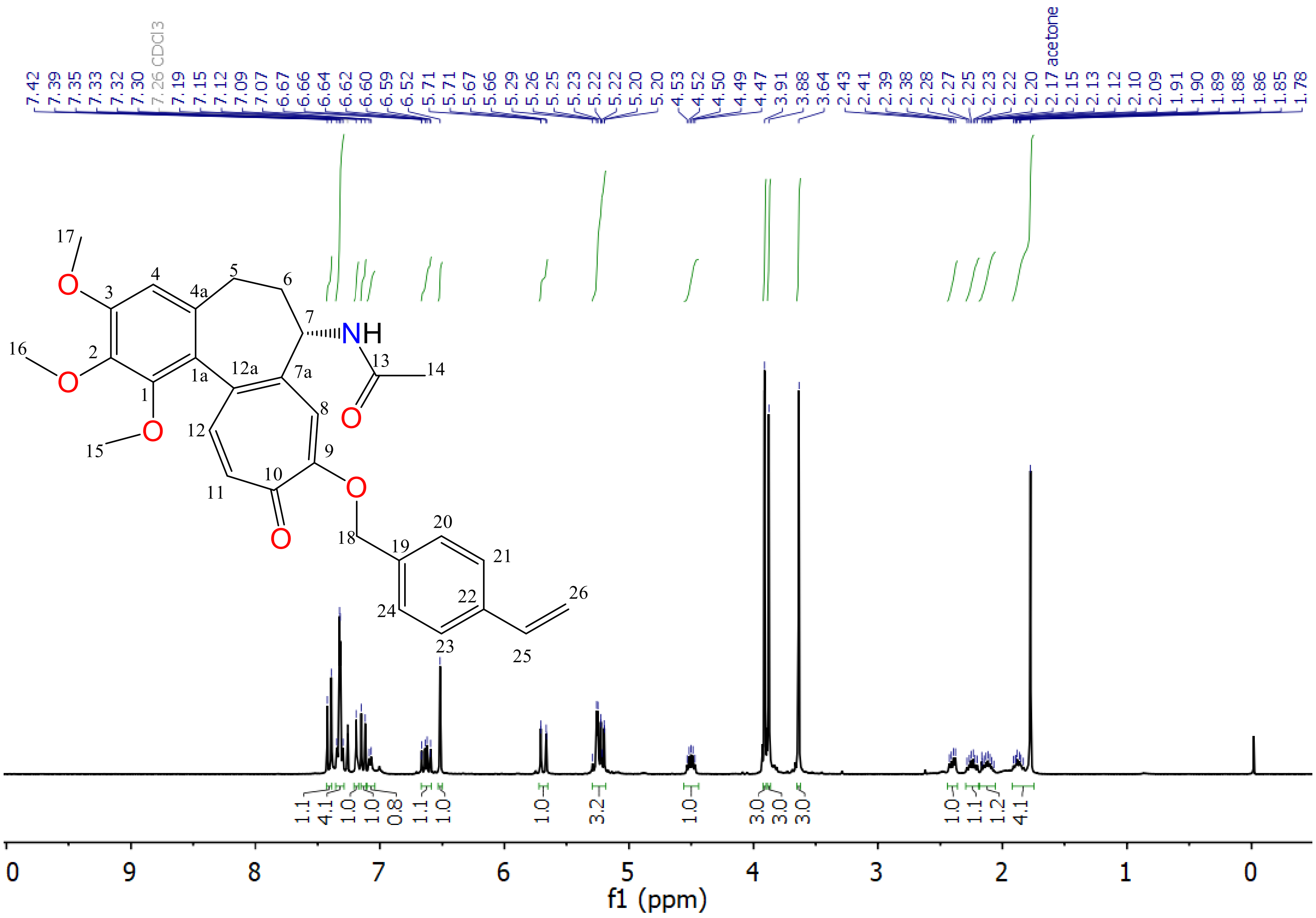

Figure 68S. ^{1}H NMR spectrum of compound **3j** in $CDCl_3$.

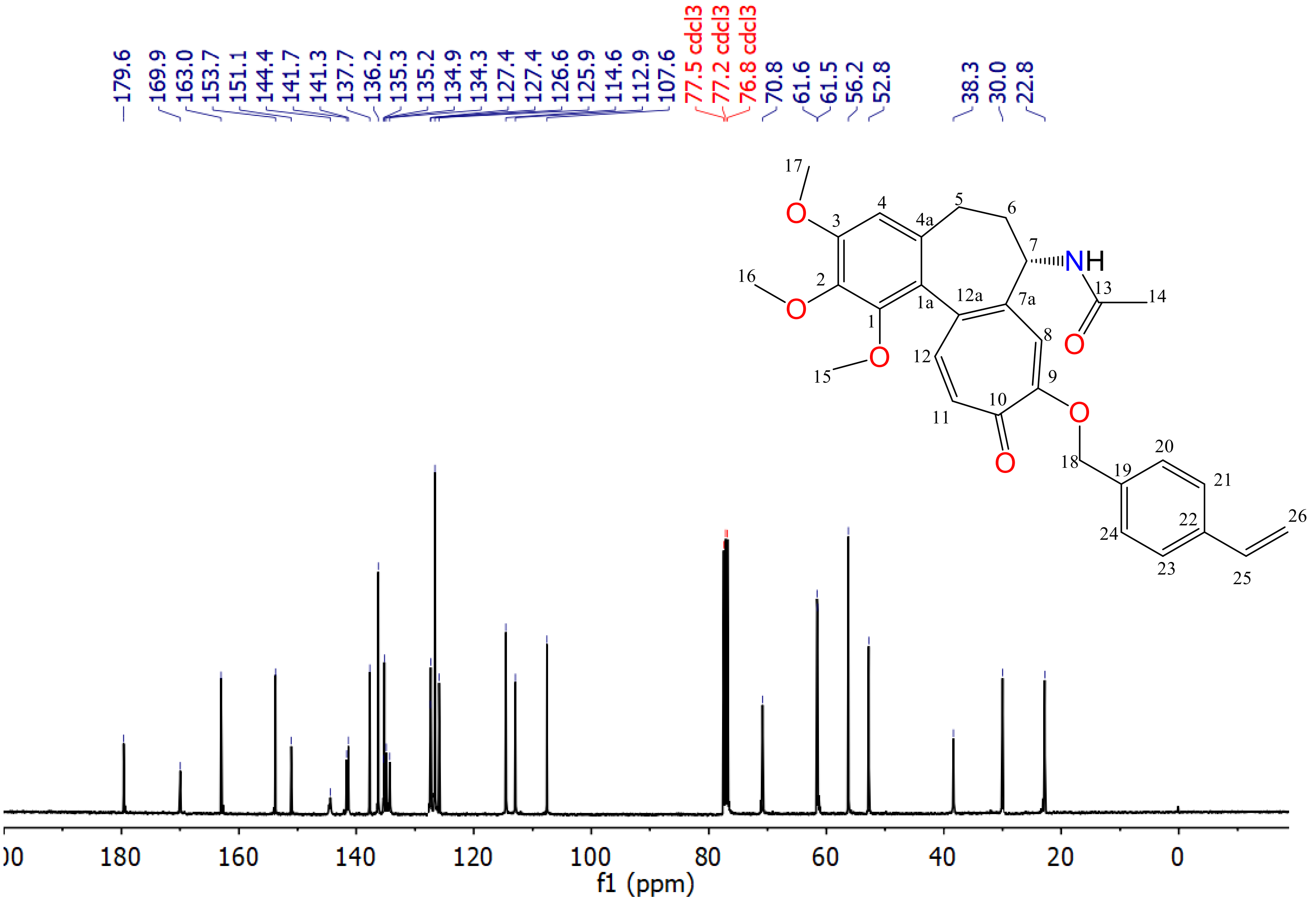

Figure 69S. ^{13}C NMR spectrum of compound **3j** in $CDCl_3$.

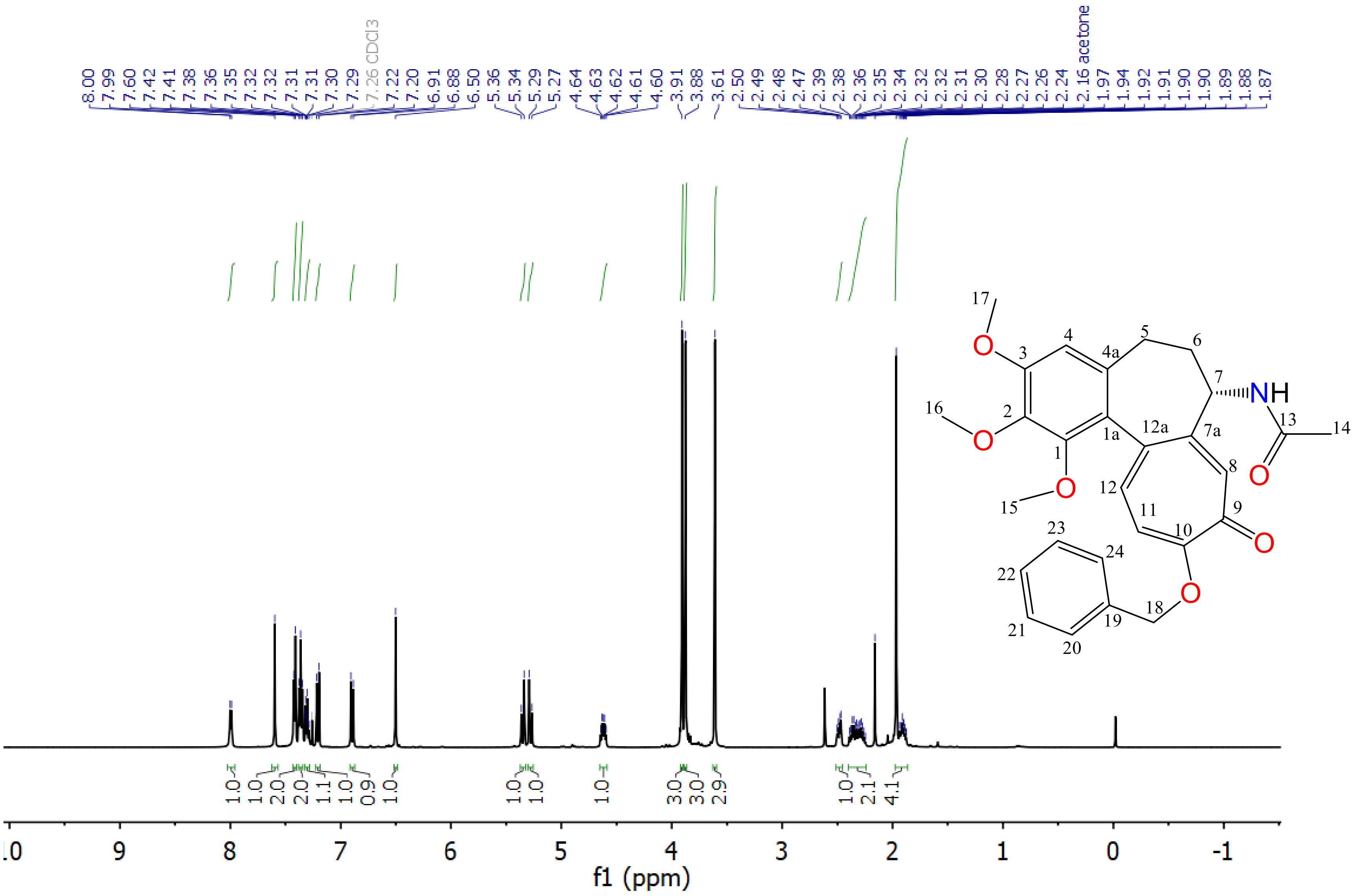

Figure 70S. ^{1}H NMR spectrum of compound **4a** in $CDCl_3$.

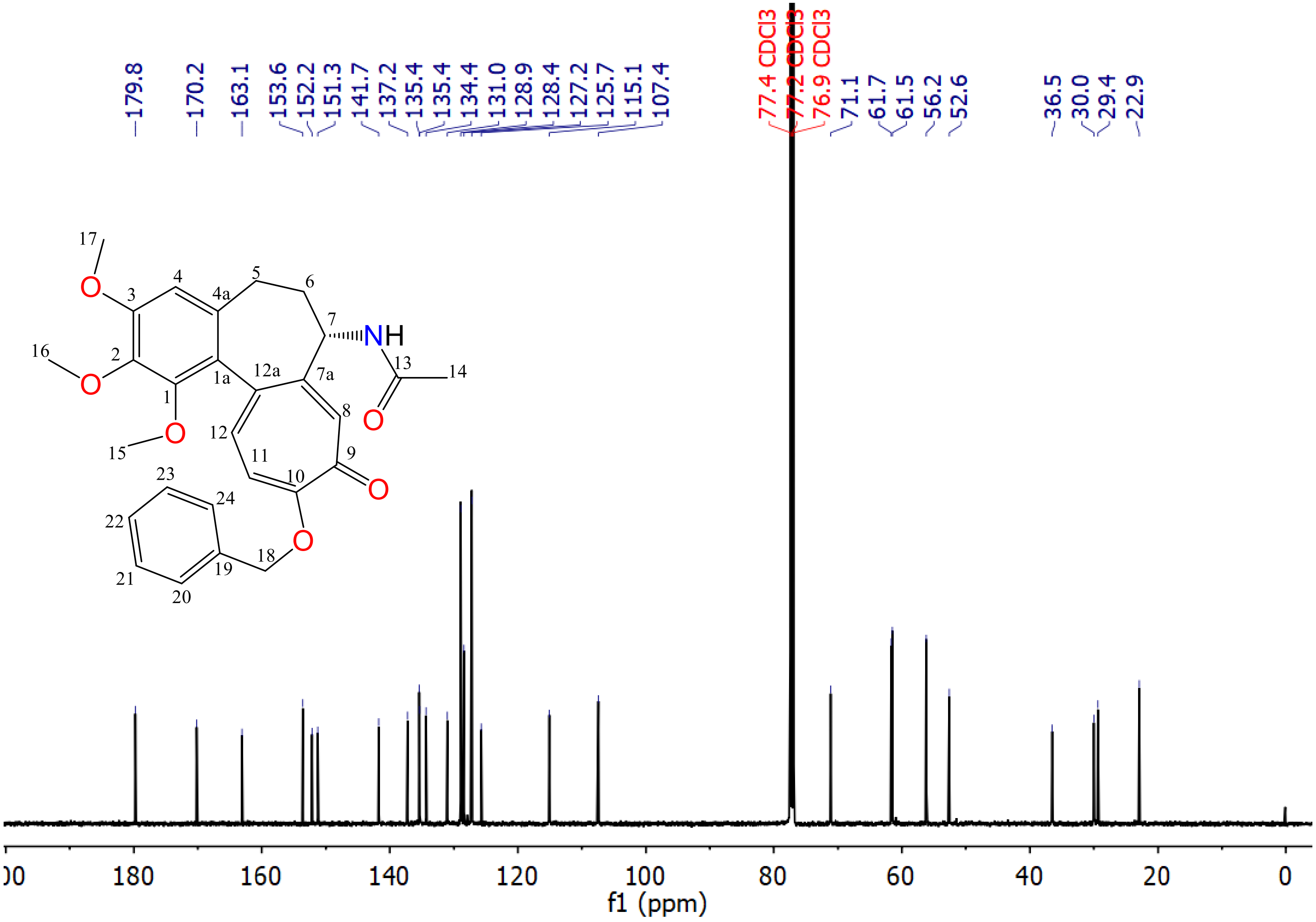

Figure 71S. ^{13}C NMR spectrum of compound **4a** in $CDCl_3$.

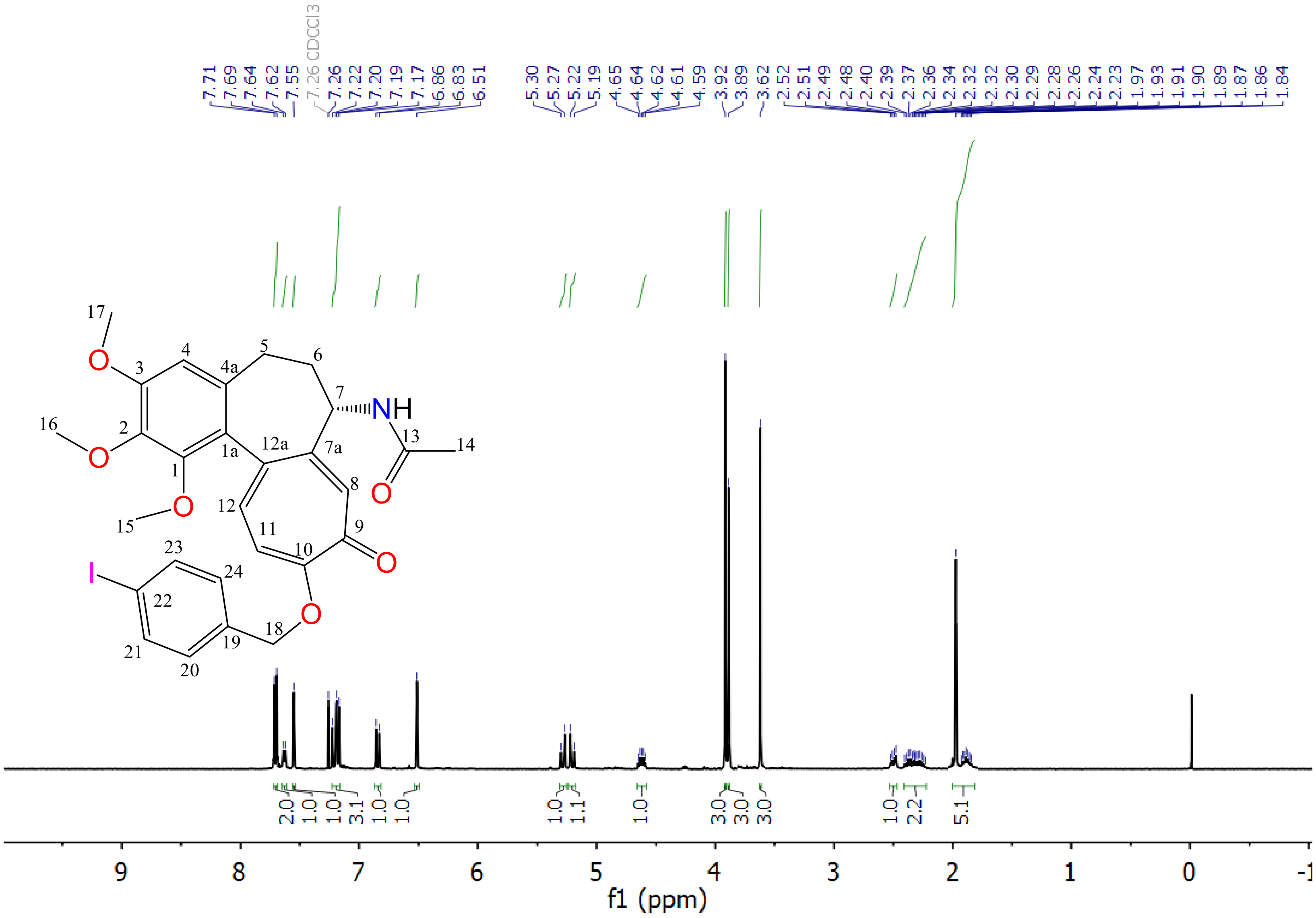

Figure 72S. ^{1}H NMR spectrum of compound **4b** in $CDCl_3$.

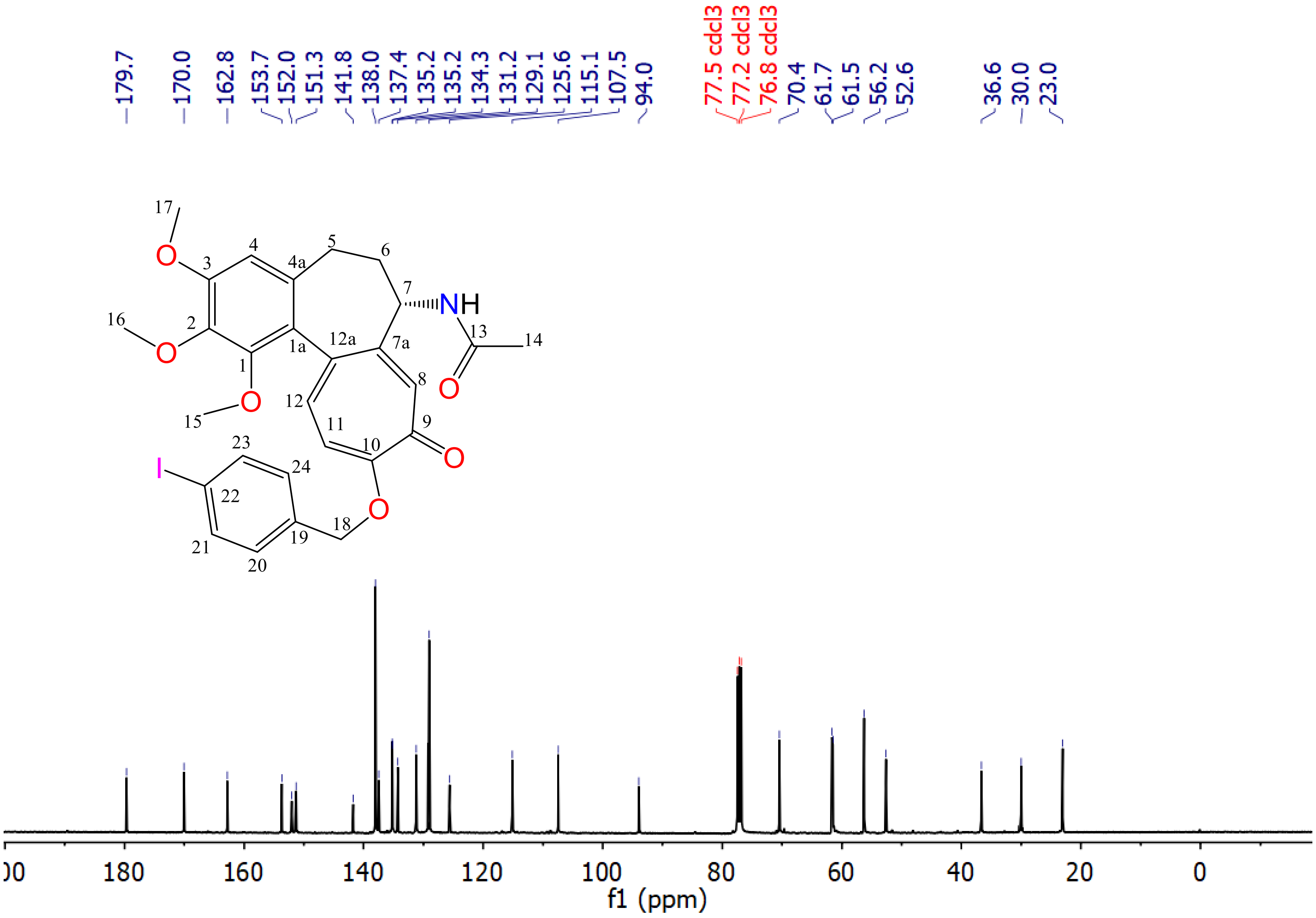

Figure 73S. ^{13}C NMR spectrum of compound **4b** in $CDCl_3$.

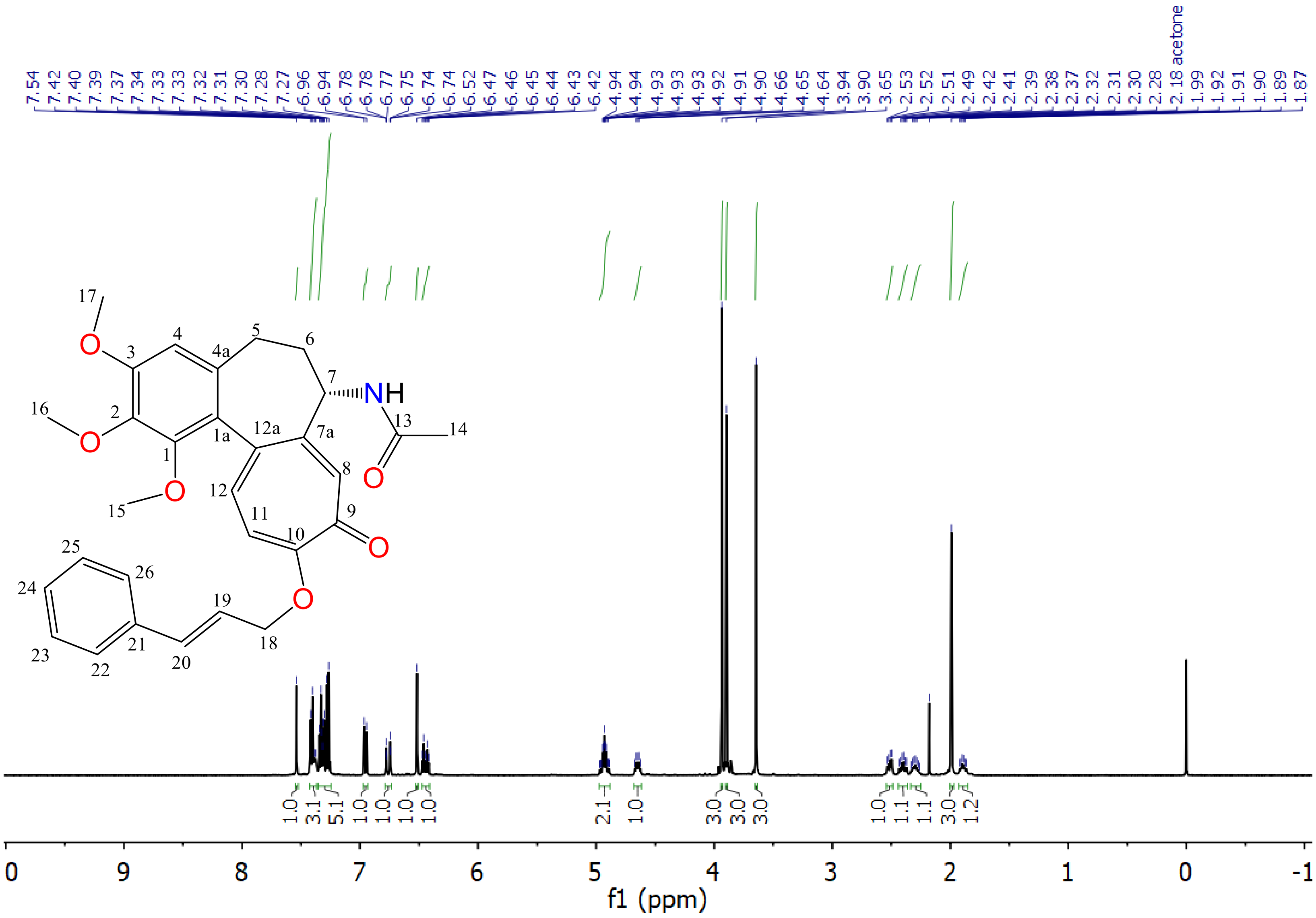

Figure 74S. ^{1}H NMR spectrum of compound **4c** in $CDCl_3$.

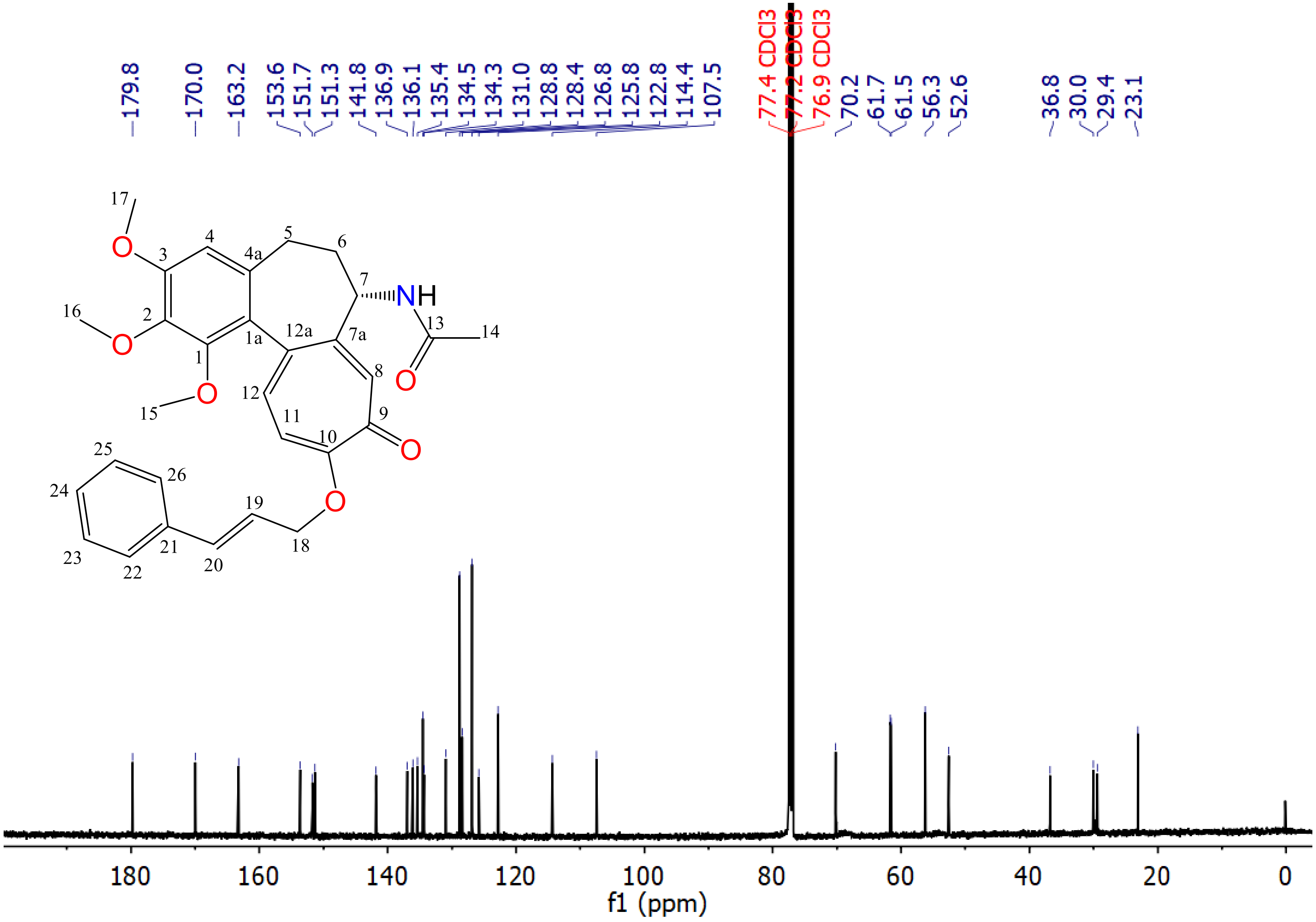

Figure 75S. ^{13}C NMR spectrum of compound **4c** in $CDCl_3$.

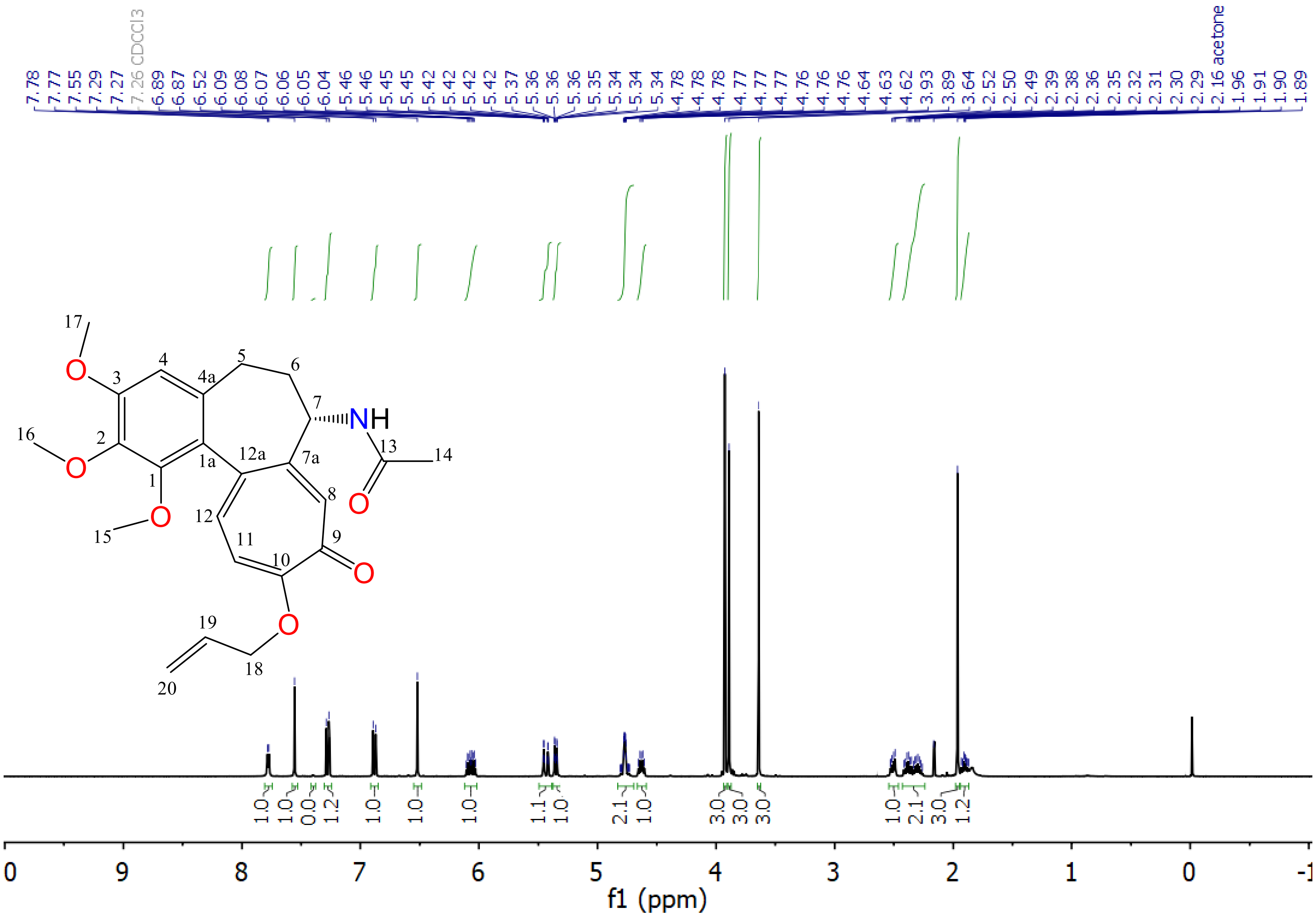

Figure 76S. ^{1}H NMR spectrum of compound **4d** in $CDCl_3$.

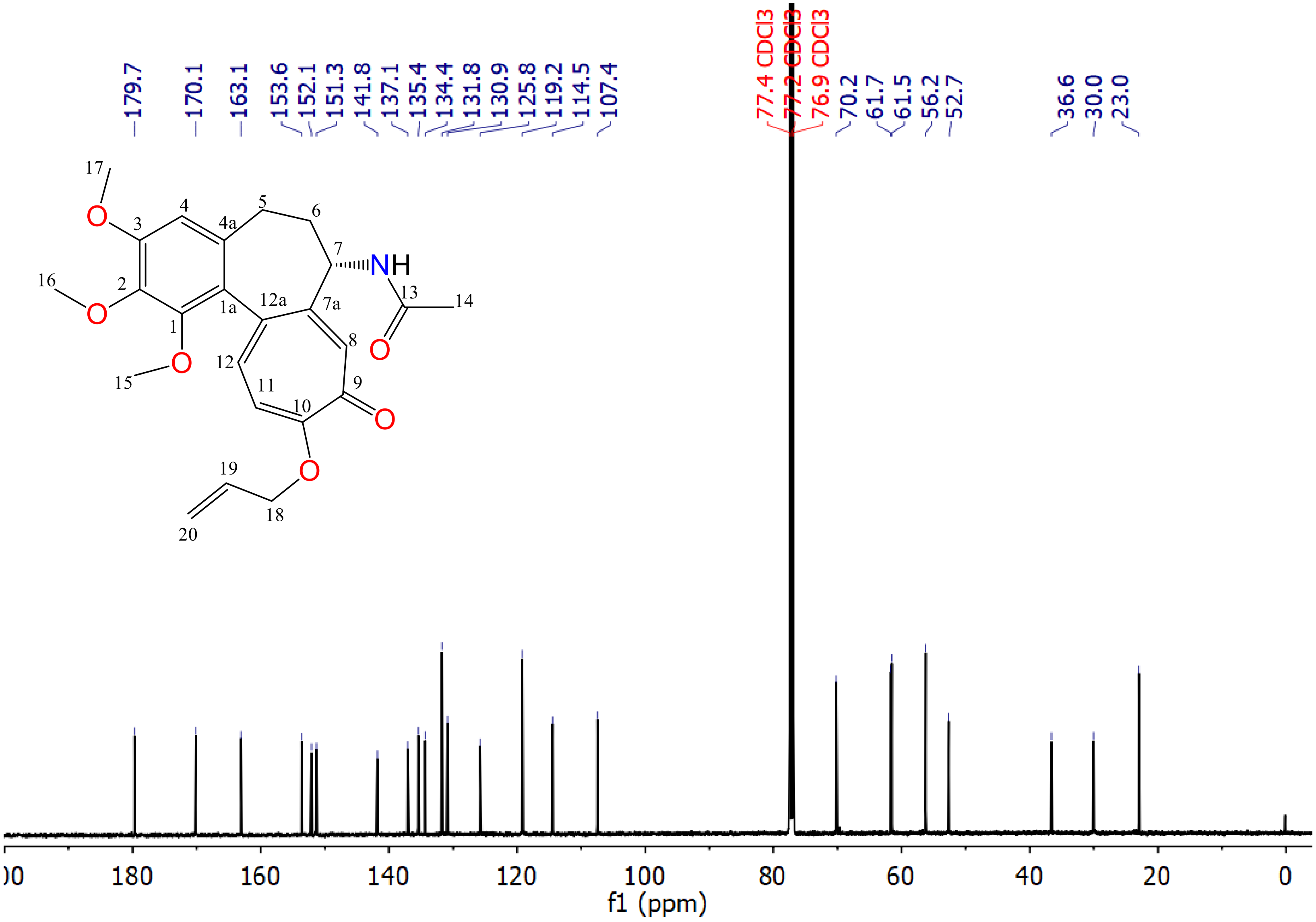

Figure 77S. ^{13}C NMR spectrum of compound **4d** in $CDCl_3$.

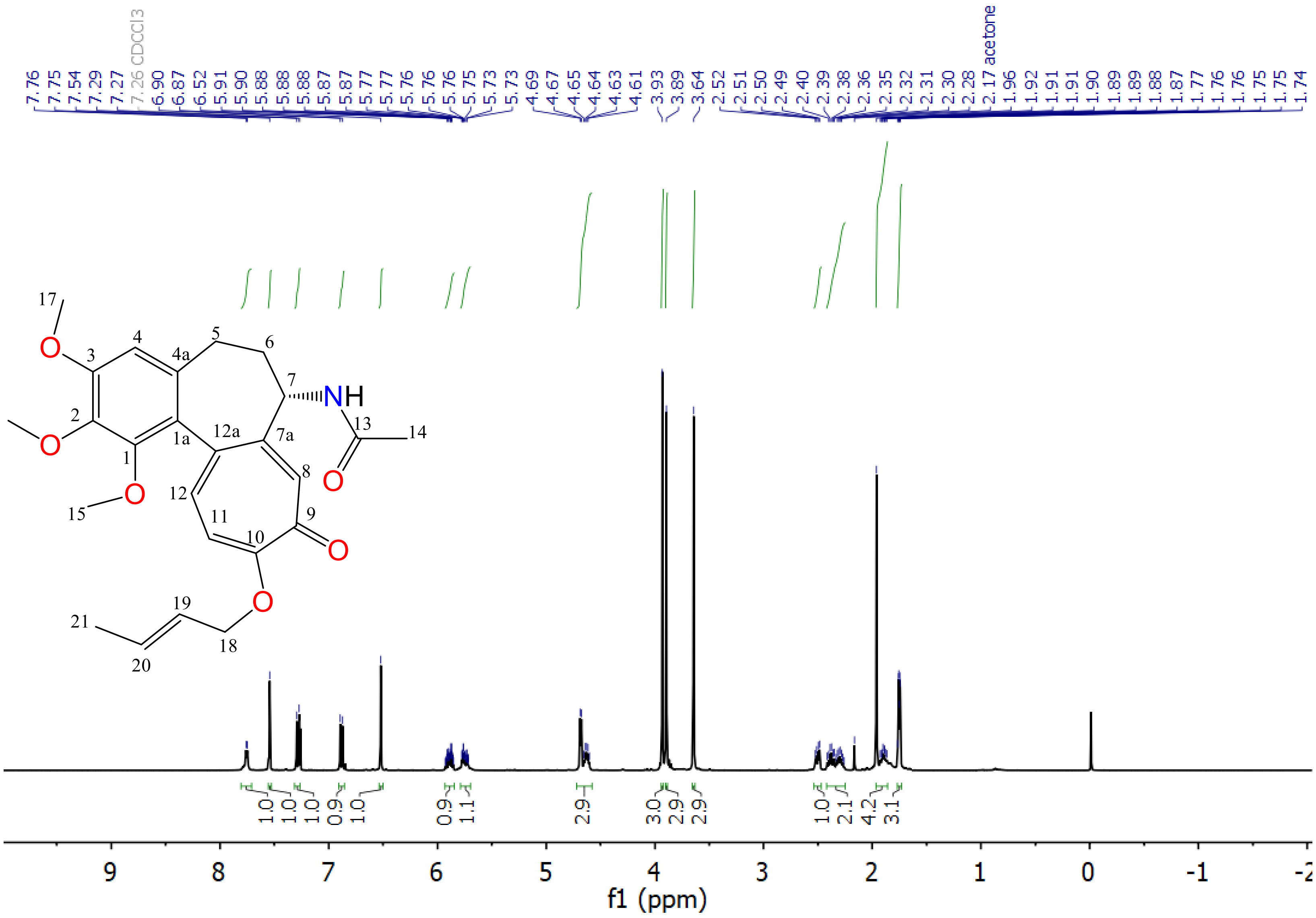

Figure 78S. ^{1}H NMR spectrum of compound **4e** in $CDCl_3$.

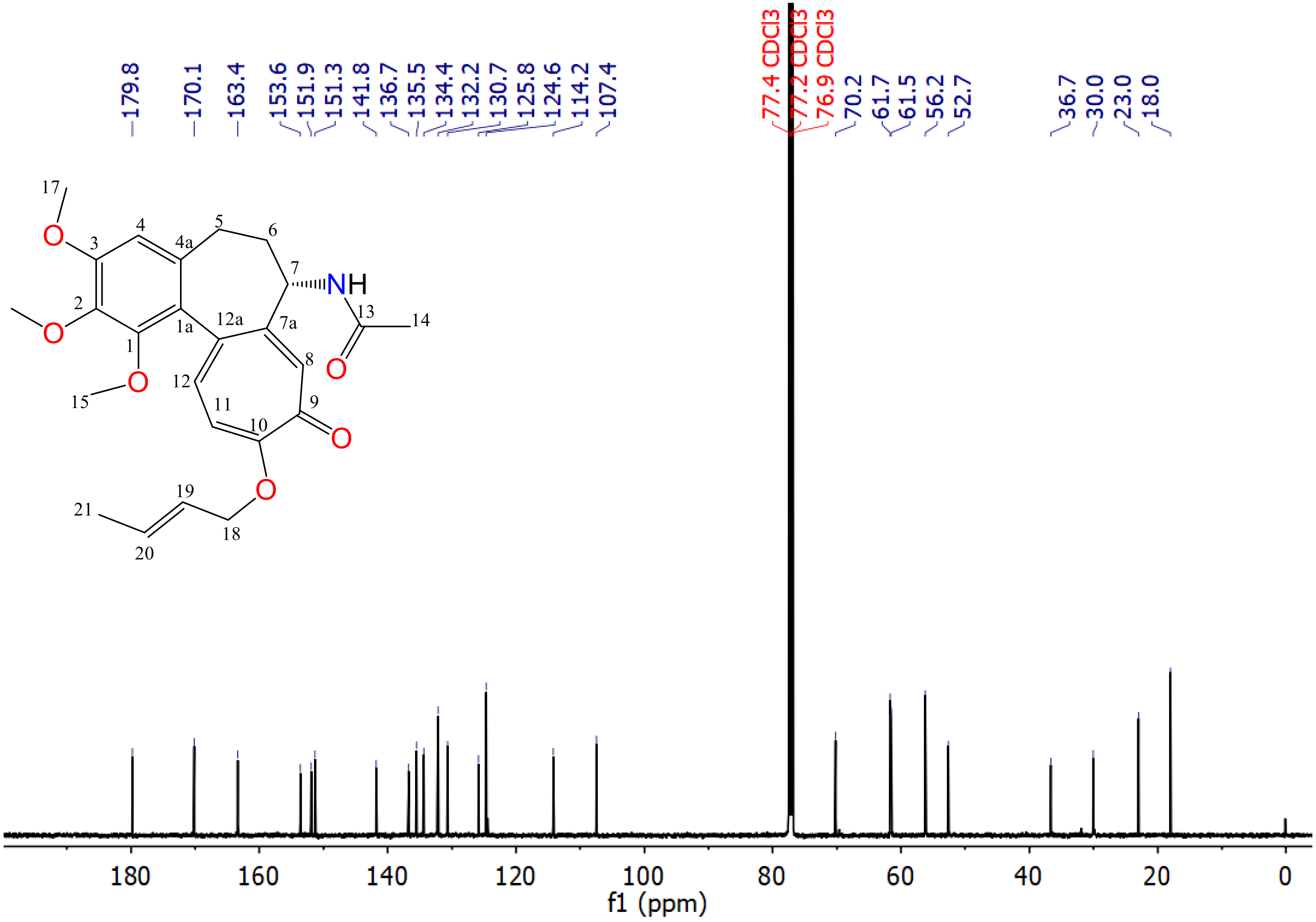

Figure 79S. ^{13}C NMR spectrum of compound **4e** in $CDCl_3$.

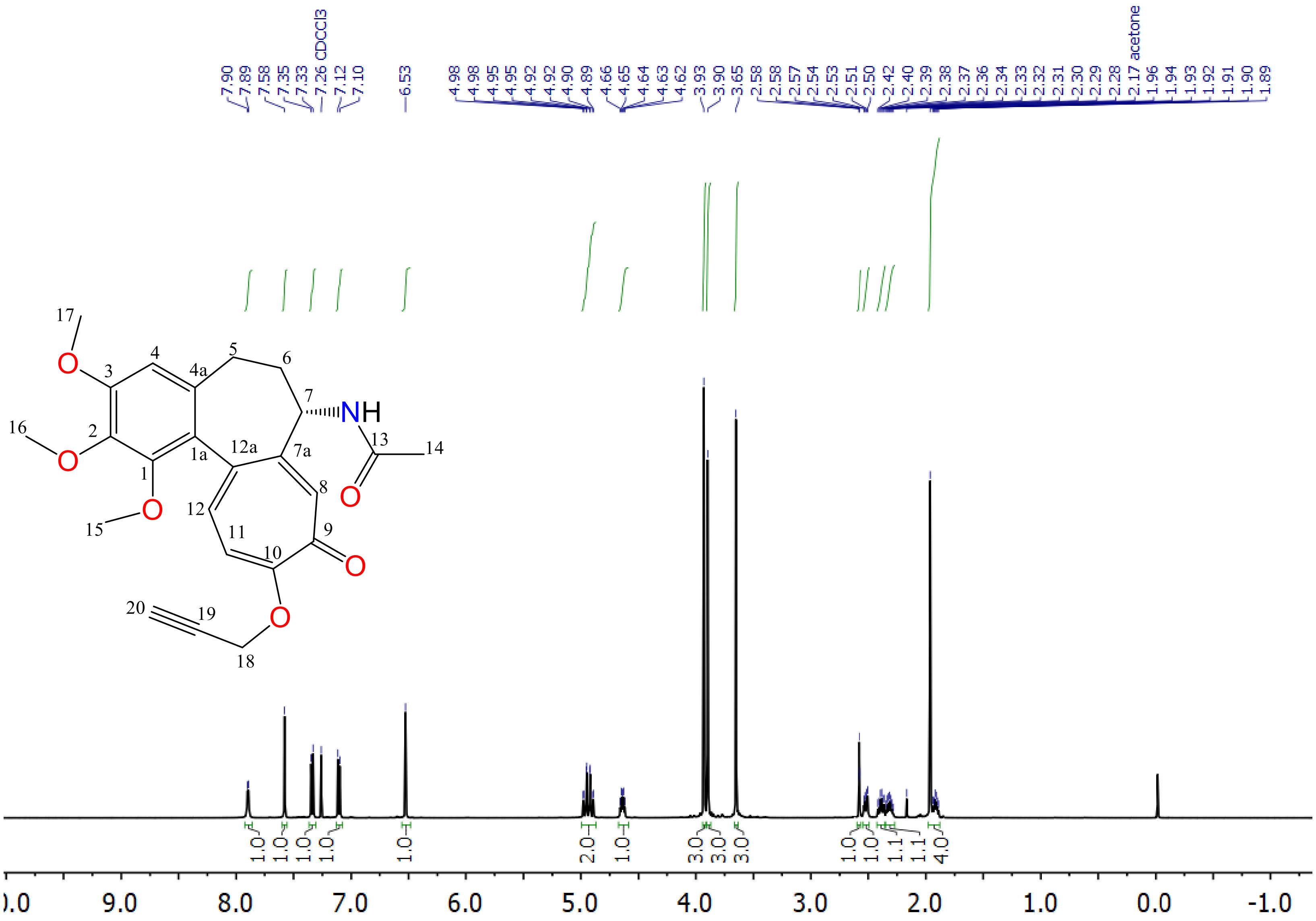

Figure 80S. ^{1}H NMR spectrum of compound **4f** in $CDCl_3$.

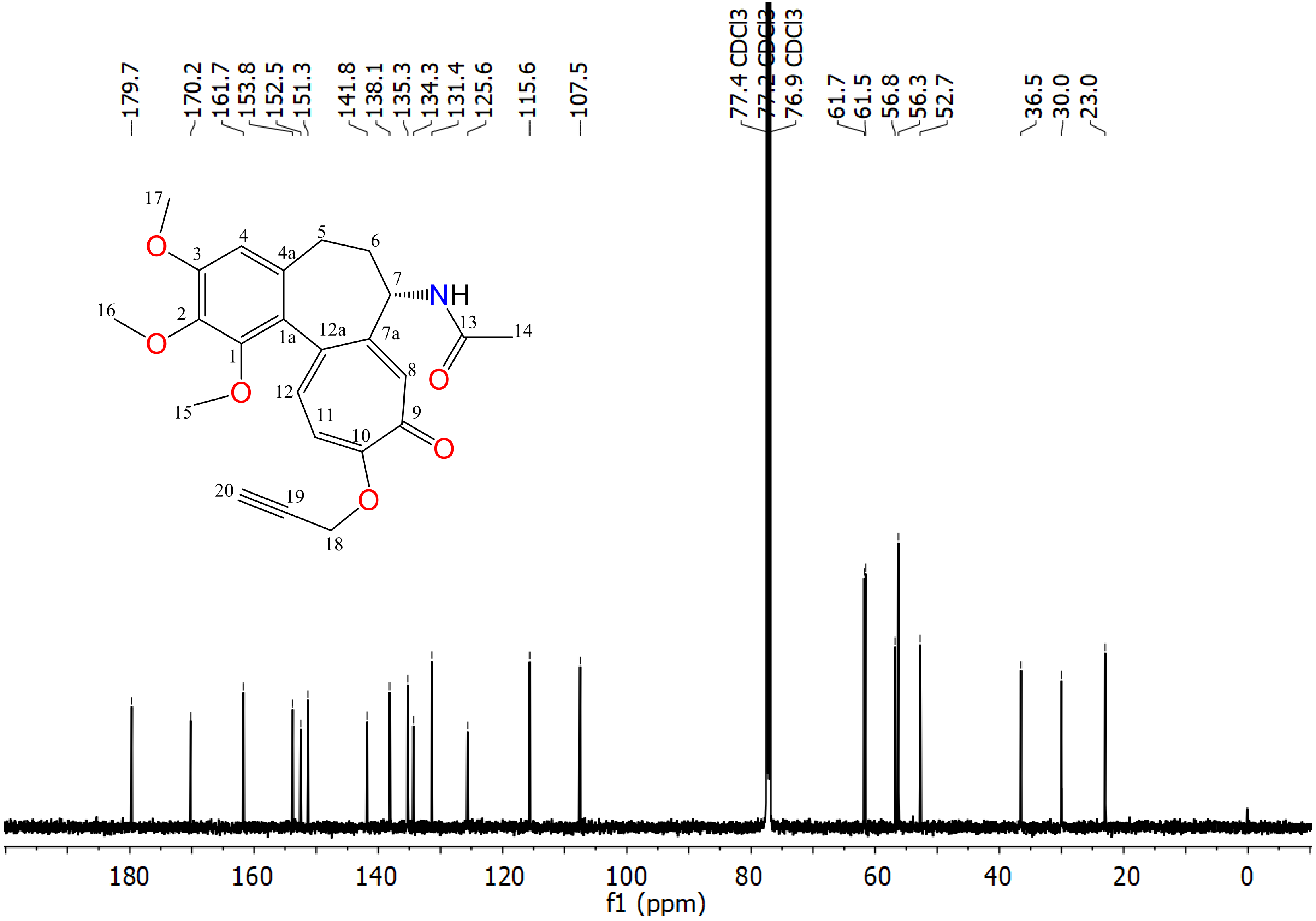

Figure 81S. ^{13}C NMR spectrum of compound **4f** in $CDCl_3$.

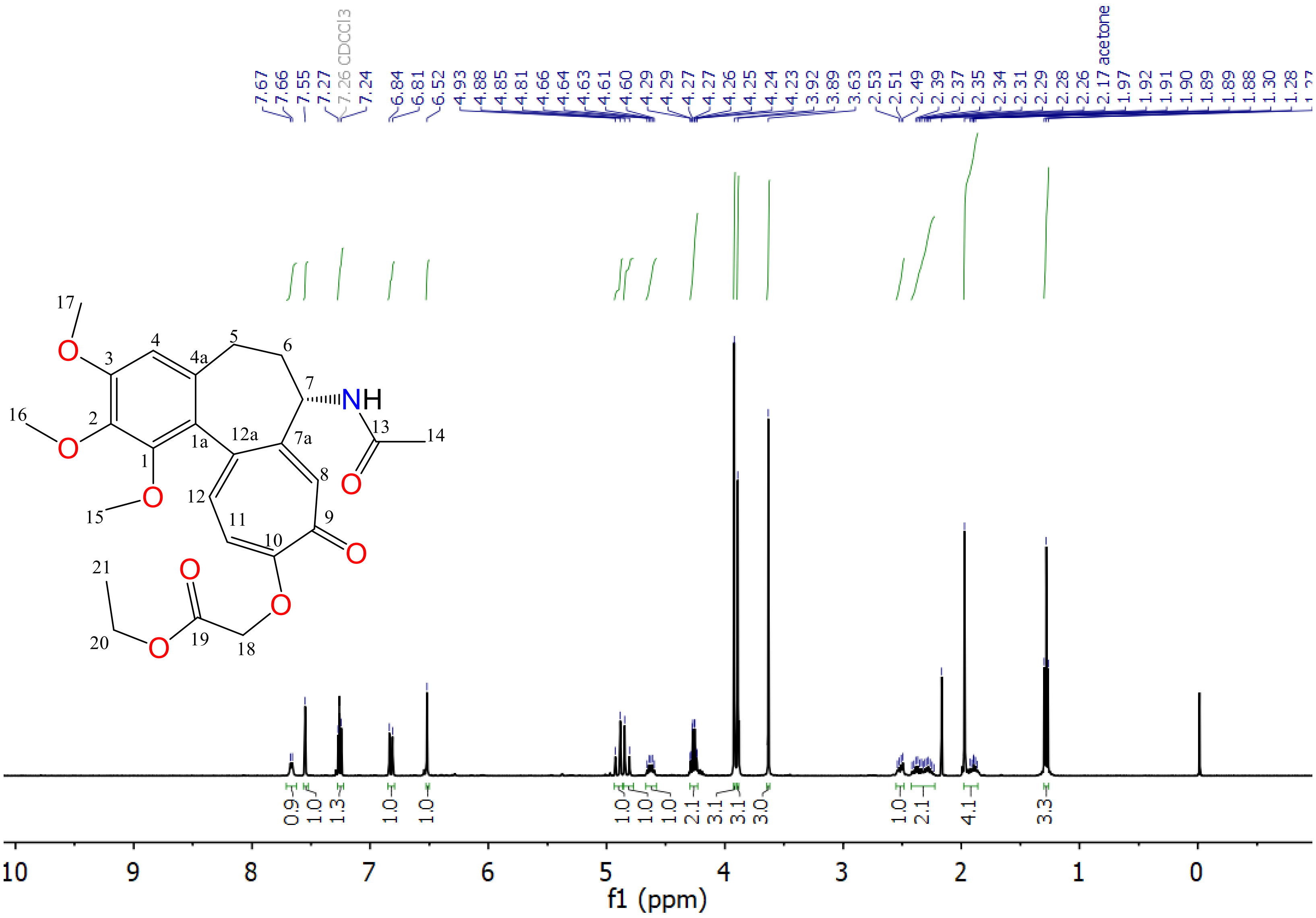

Figure 82S. ^{1}H NMR spectrum of compound **4g** in $CDCl_3$.

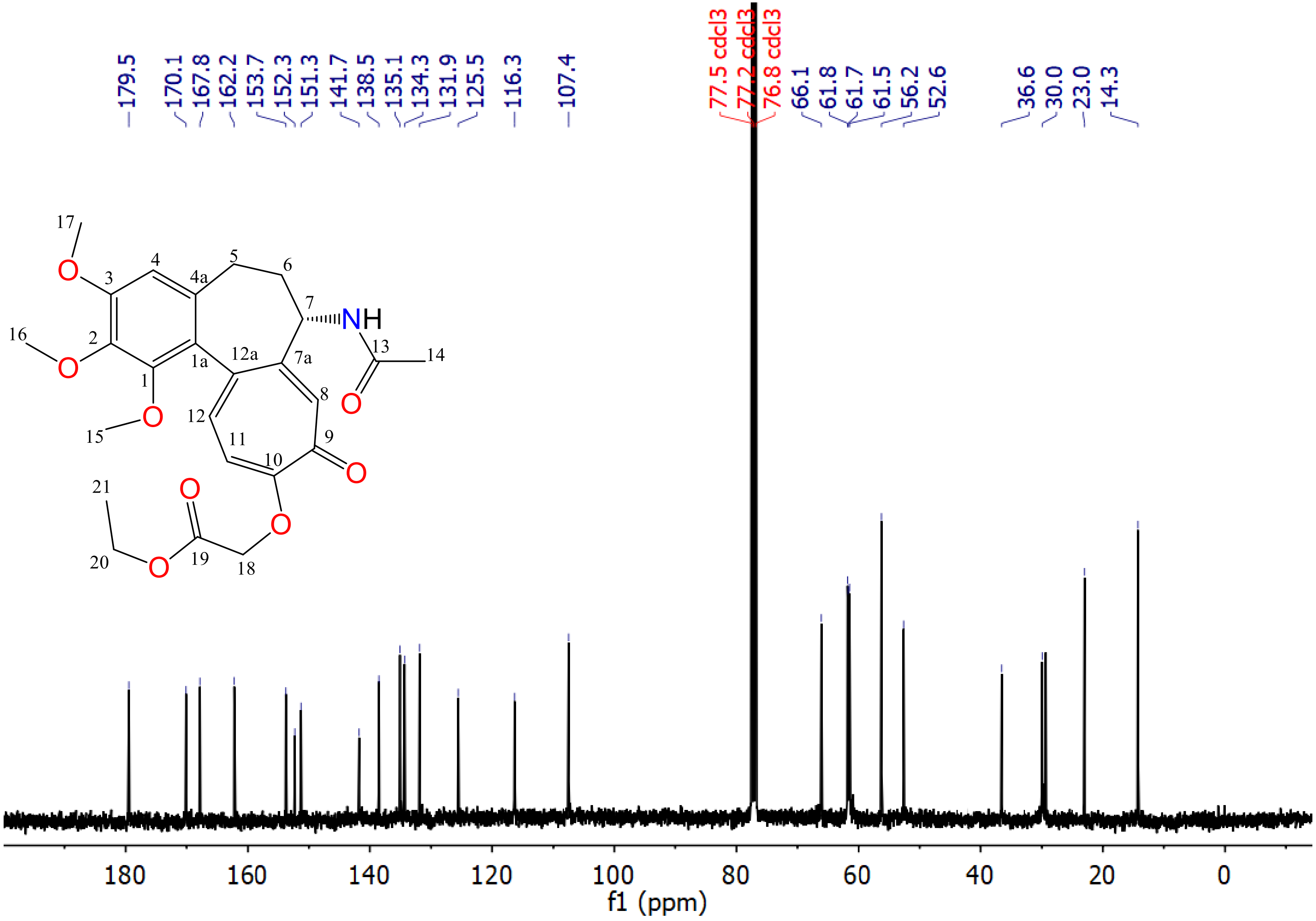

Figure 83S. ^{13}C NMR spectrum of compound **4g** in $CDCl_3$.

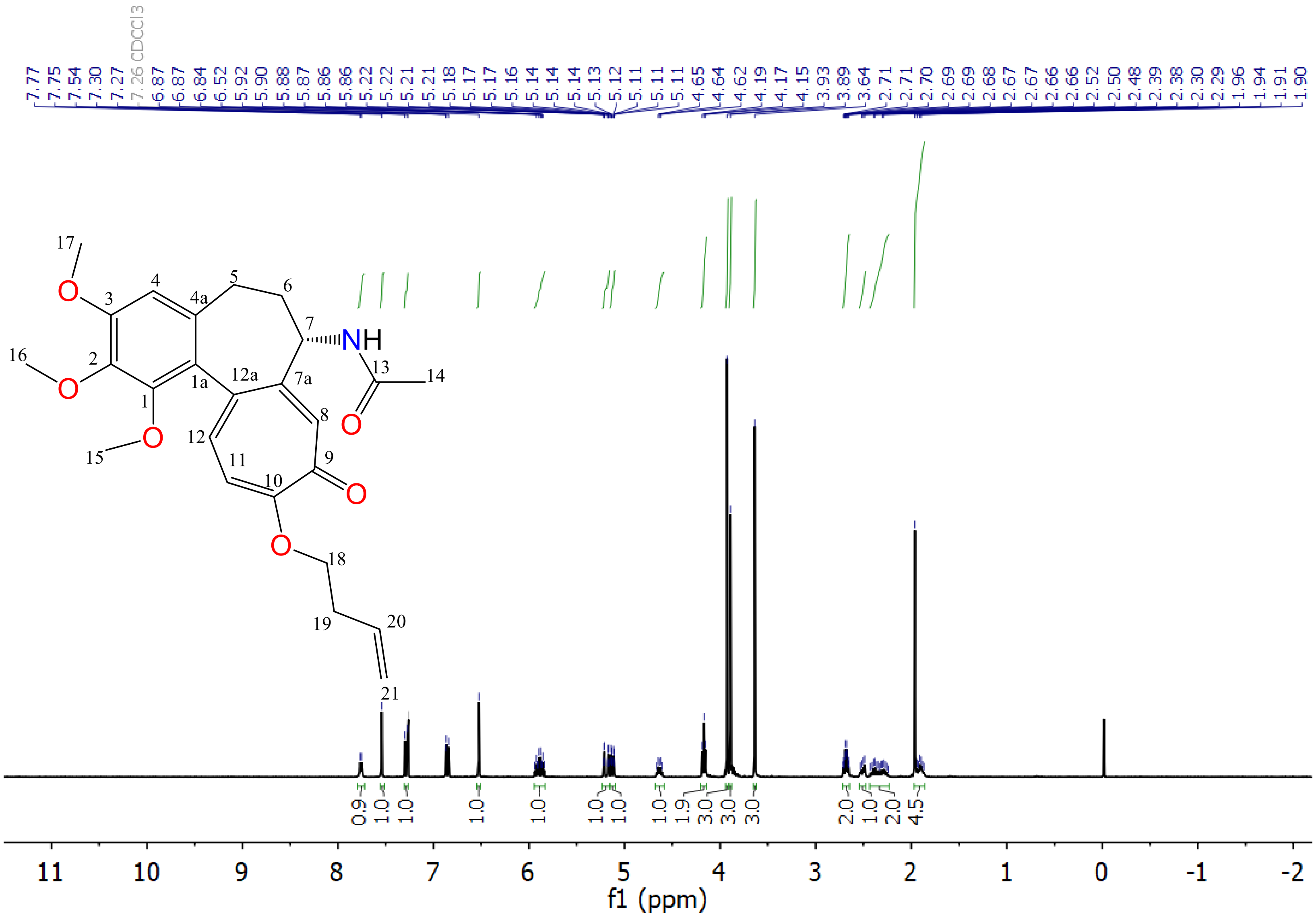

Figure 84S. ^{1}H NMR spectrum of compound **4h** in $CDCl_3$.

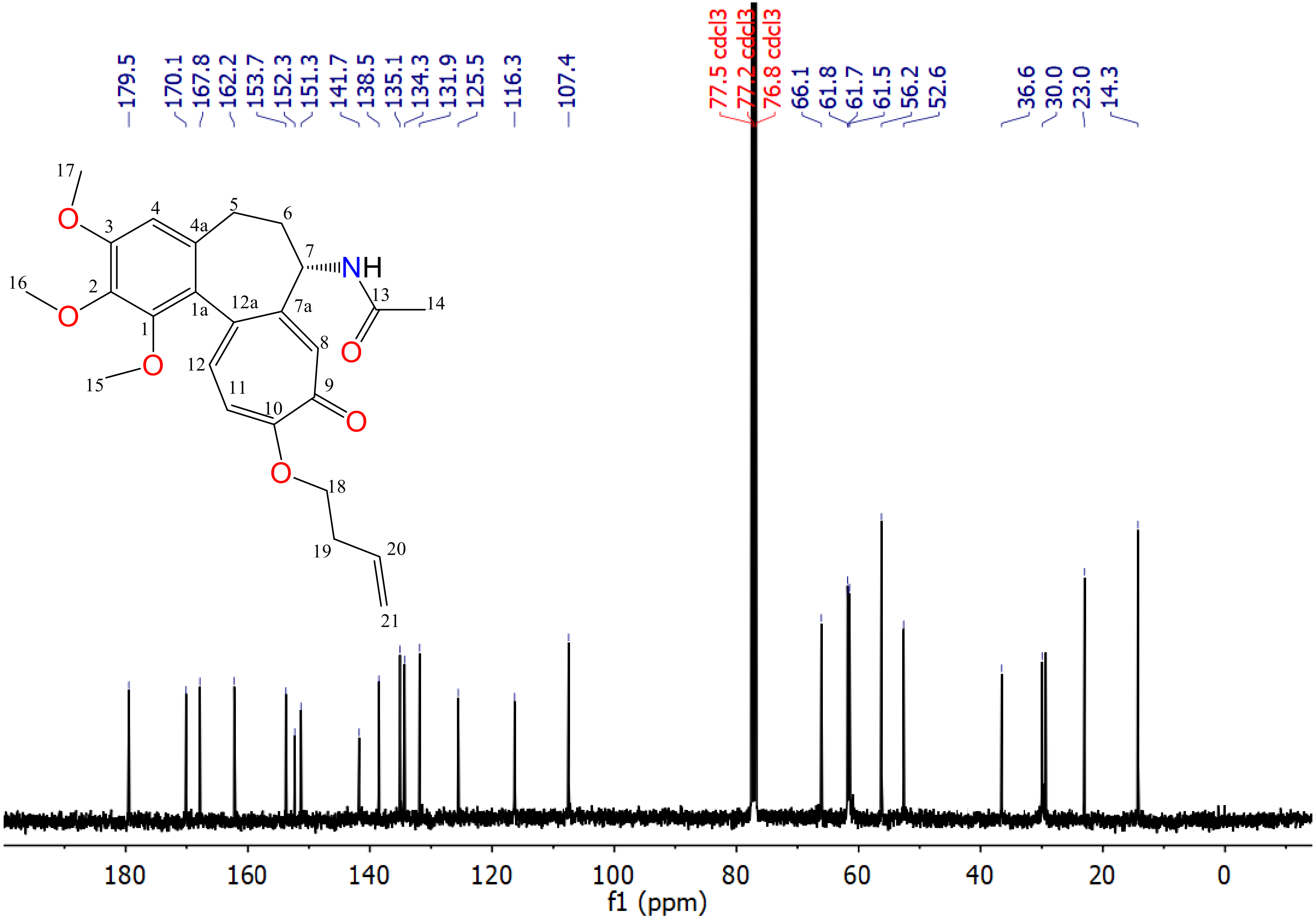

Figure 85S. ^{13}C NMR spectrum of compound **4h** in $CDCl_3$.

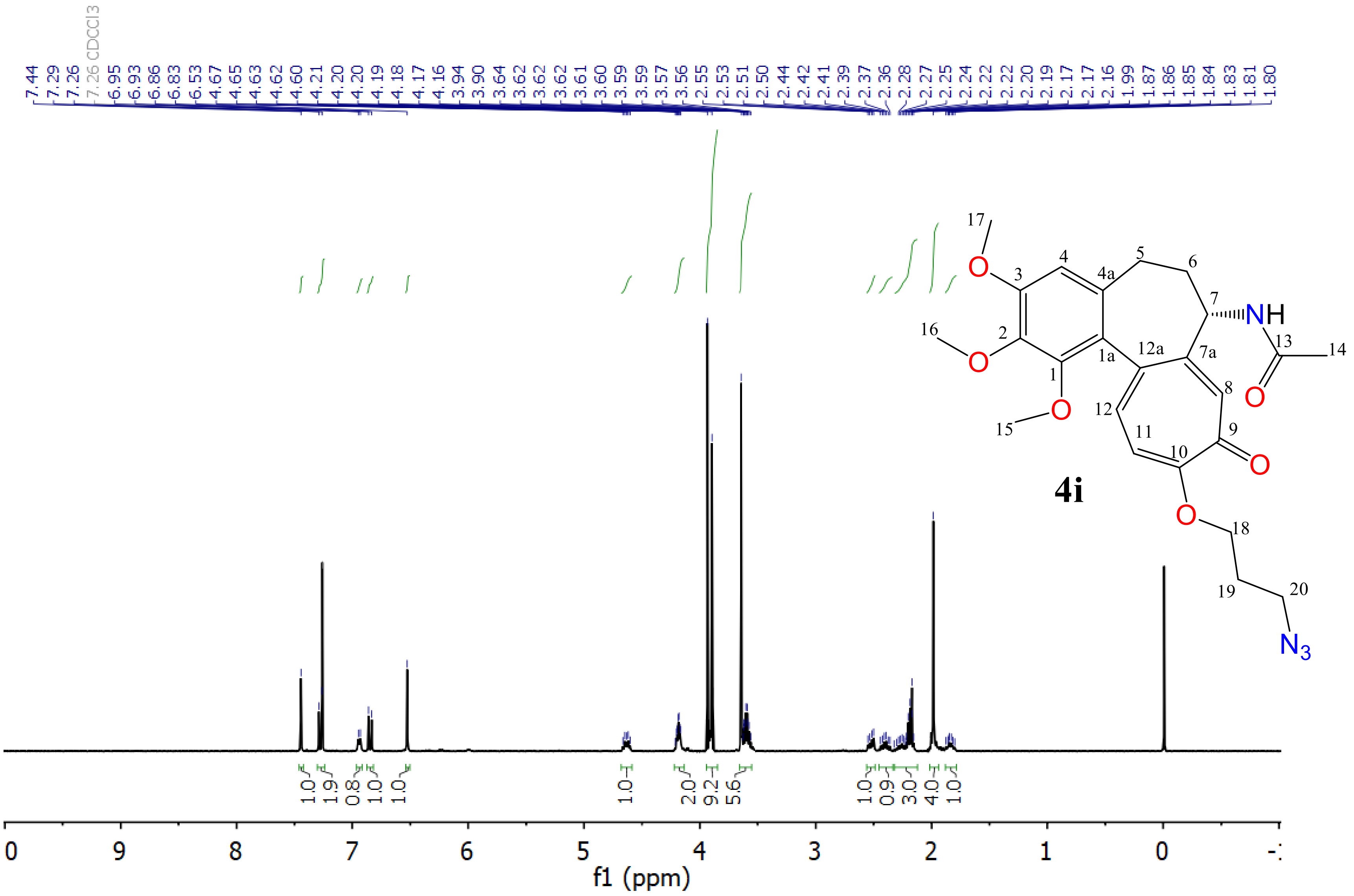

Figure 86S. ^{1}H NMR spectrum of compound **4i** in $CDCl_3$.

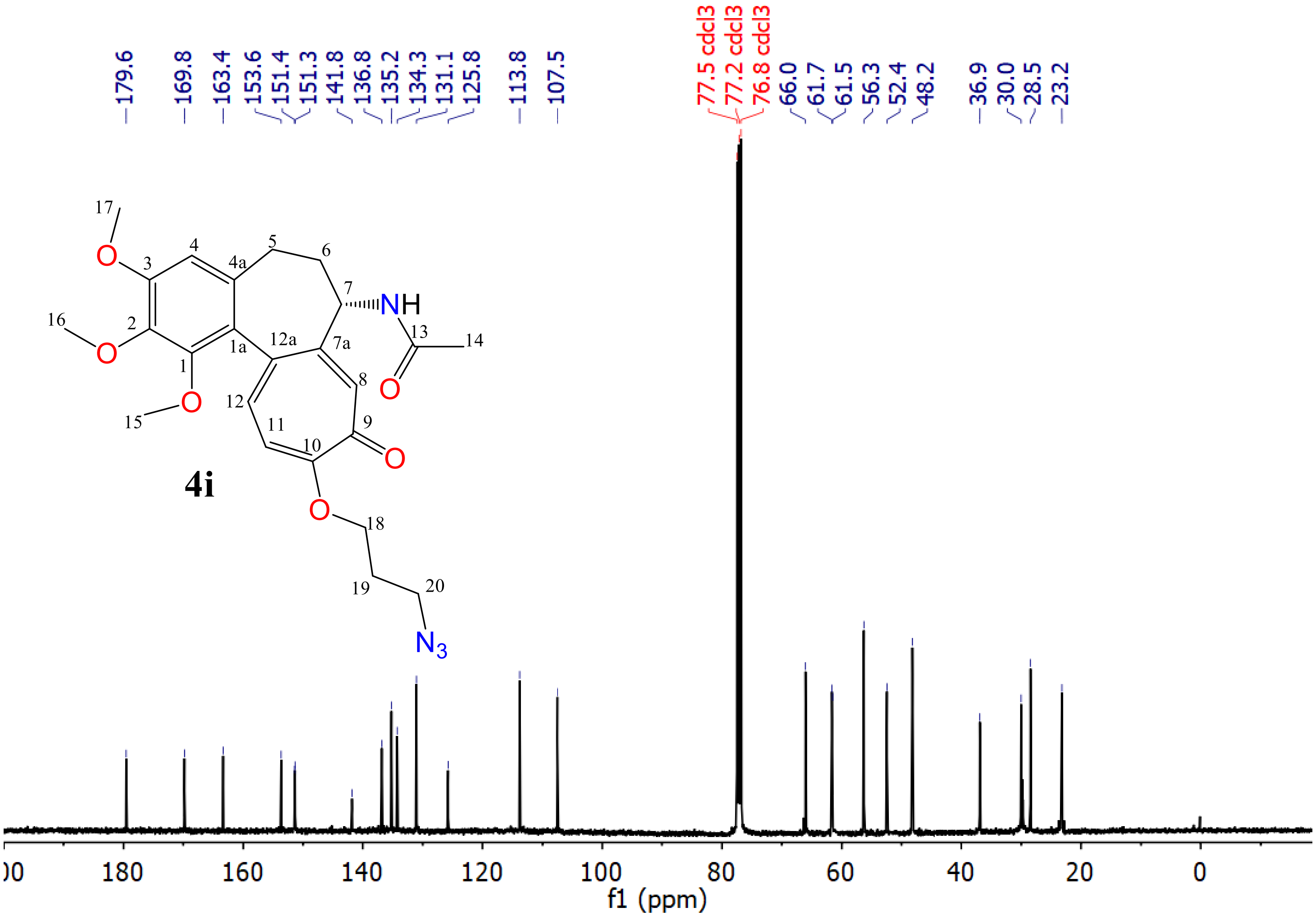

Figure 87S. ^{13}C NMR spectrum of compound **4i** in $CDCl_3$.

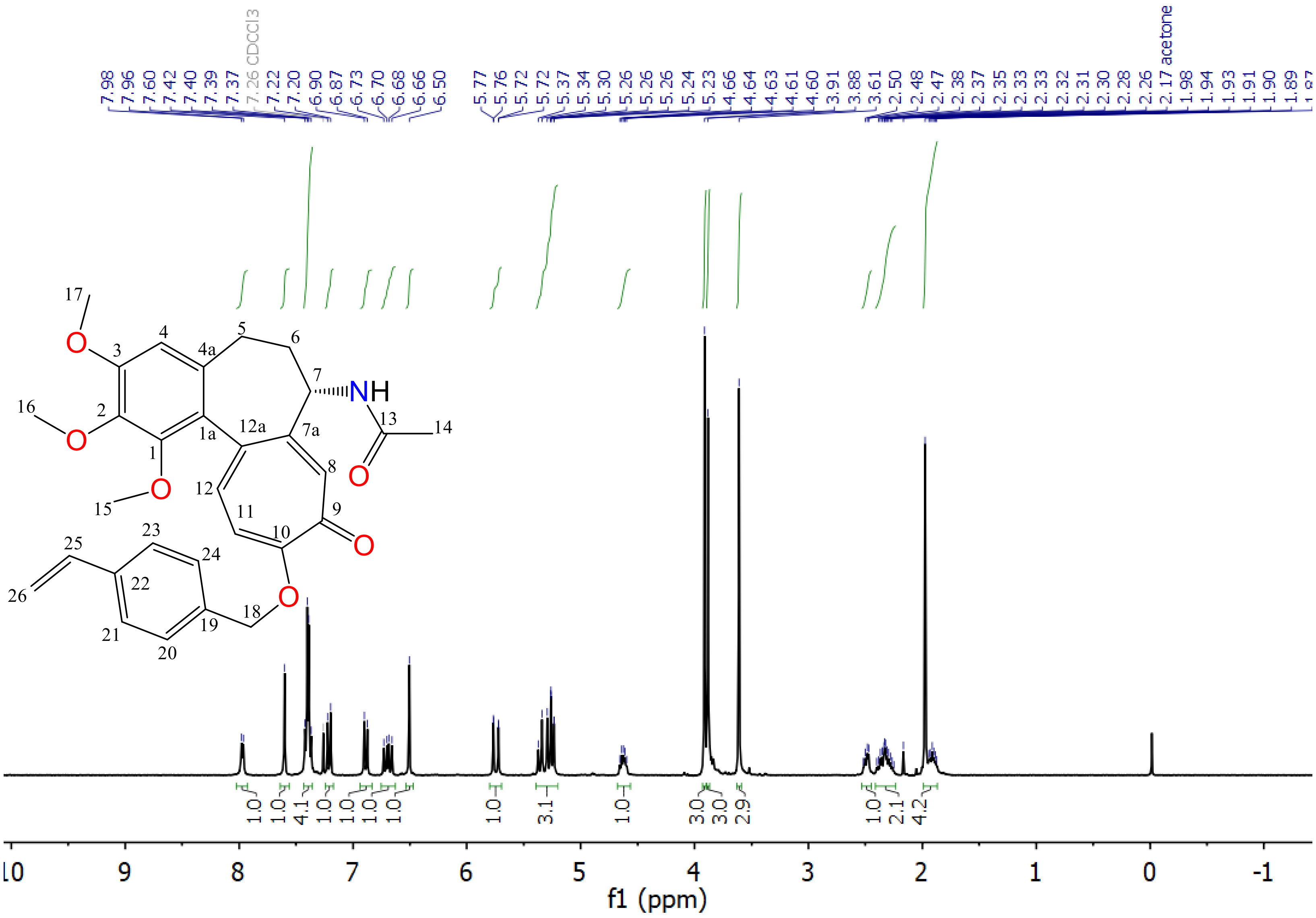

Figure 88S. ^{1}H NMR spectrum of compound **4j** in $CDCl_3$.

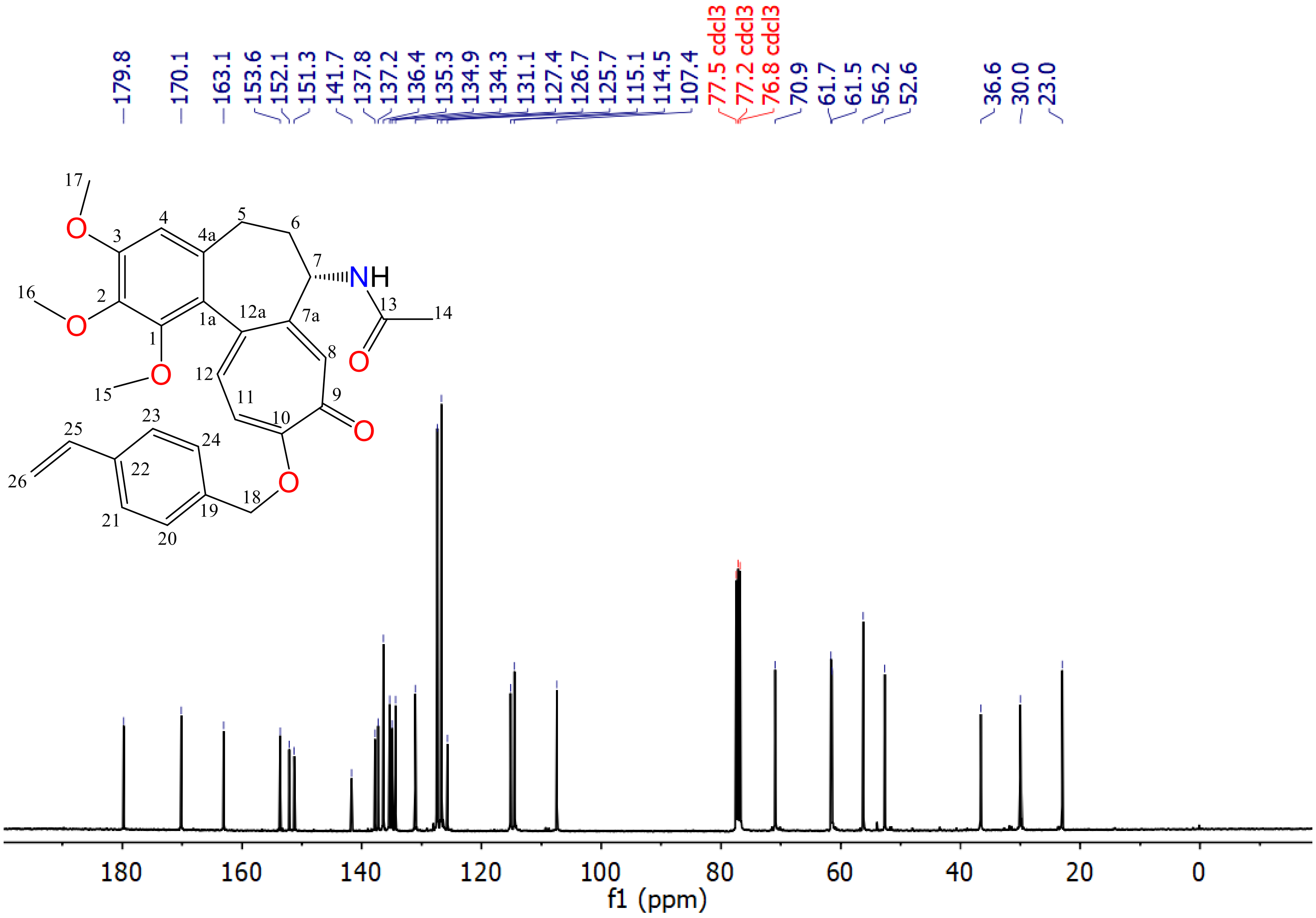

Figure 89S. ^{13}C NMR spectrum of compound **4j** in $CDCl_3$.

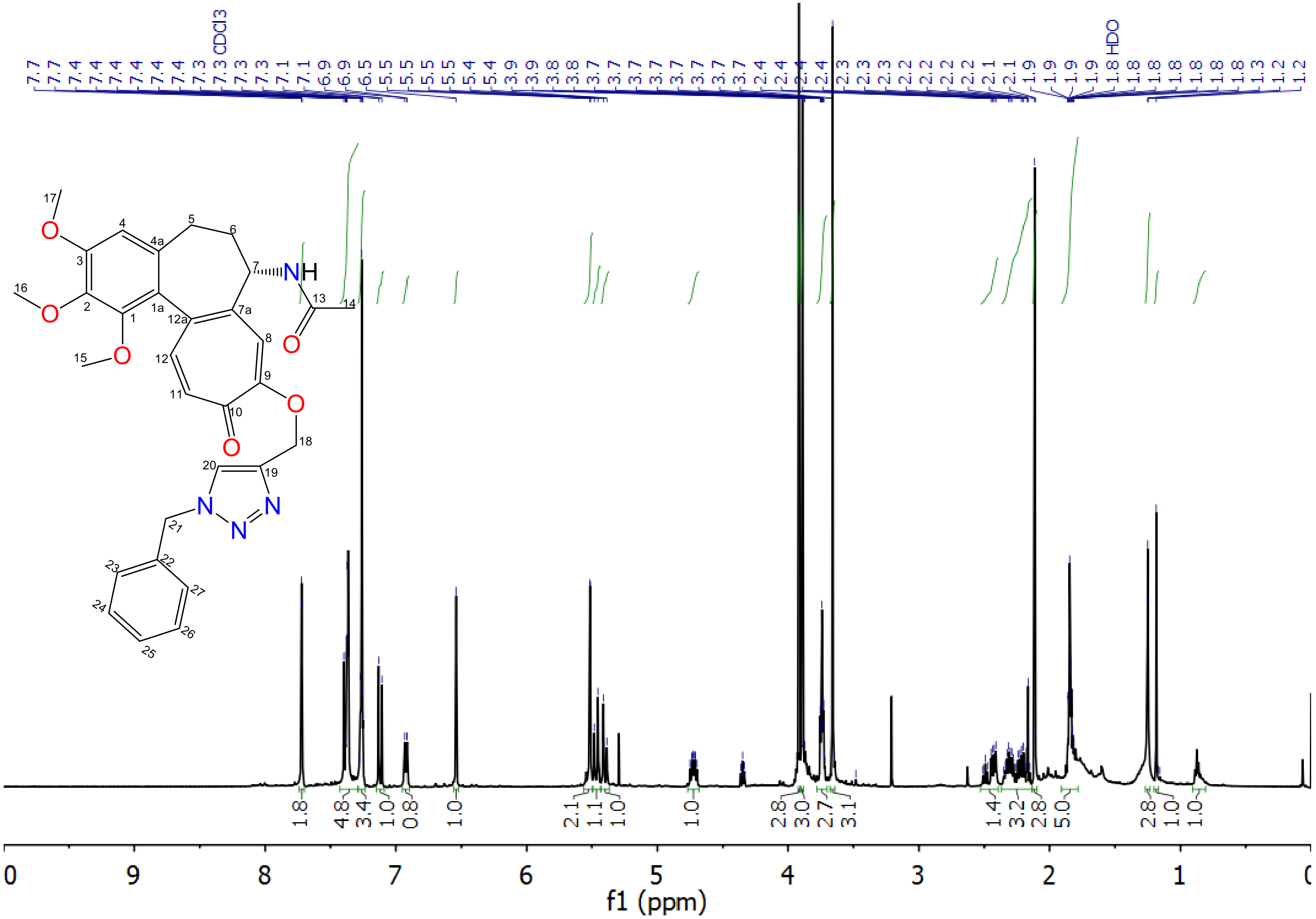

Figure 90S. ^{1}H NMR spectrum of compound **5a** in $CDCl_3$.

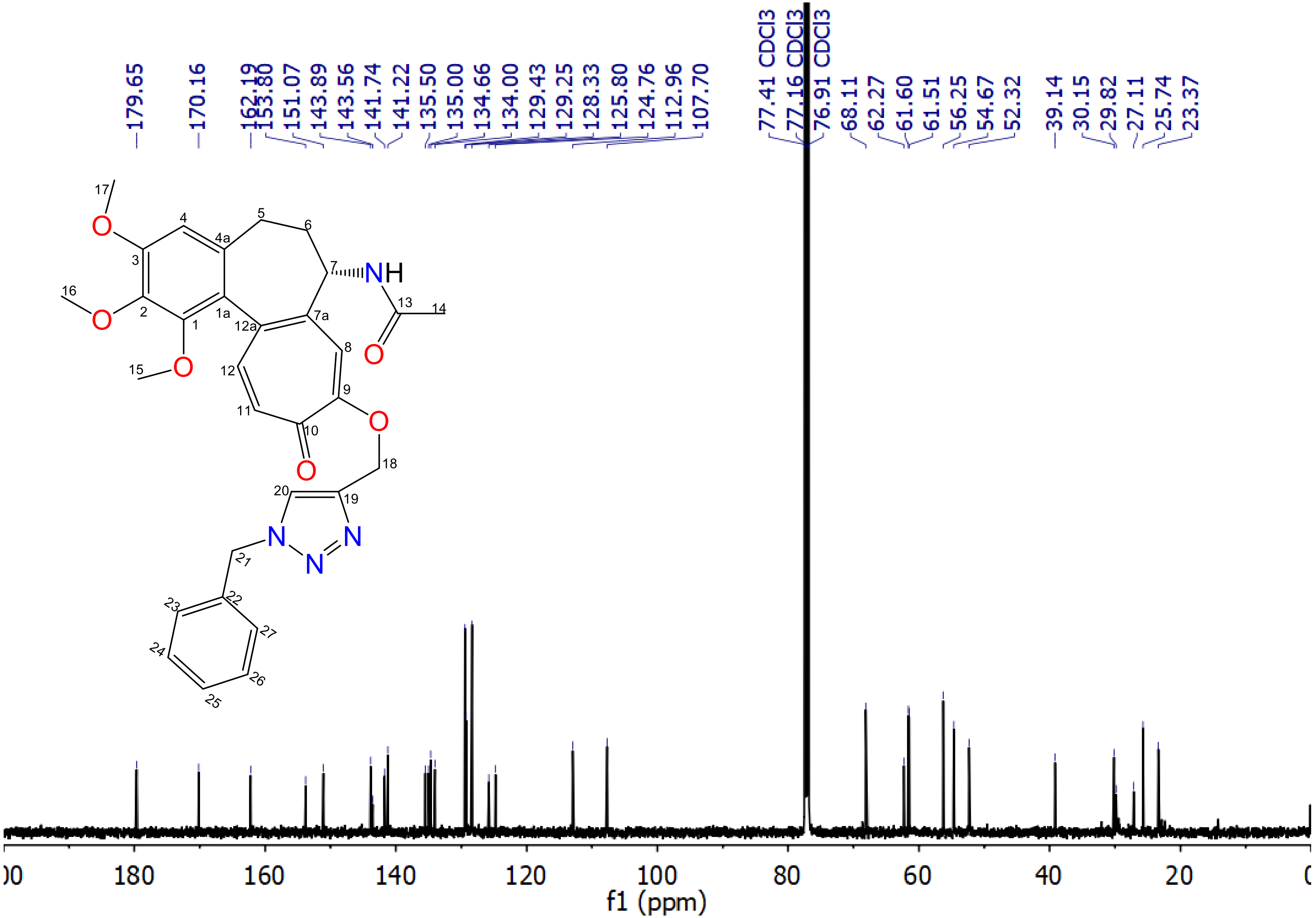

Figure 91S. ^{13}C NMR spectrum of compound **5a** in $CDCl_3$.

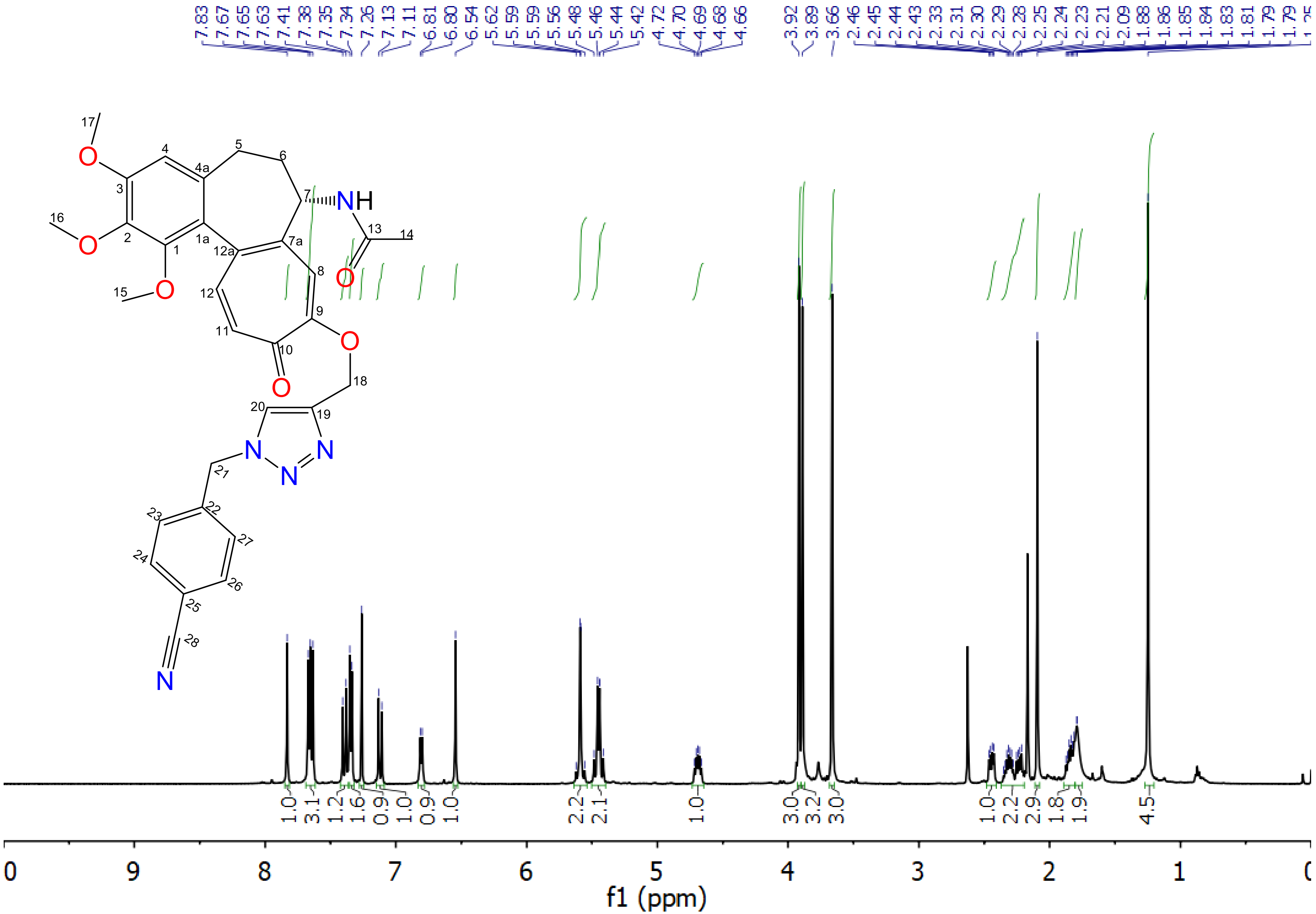

Figure 92S. ^{1}H NMR spectrum of compound **5b** in $CDCl_3$.

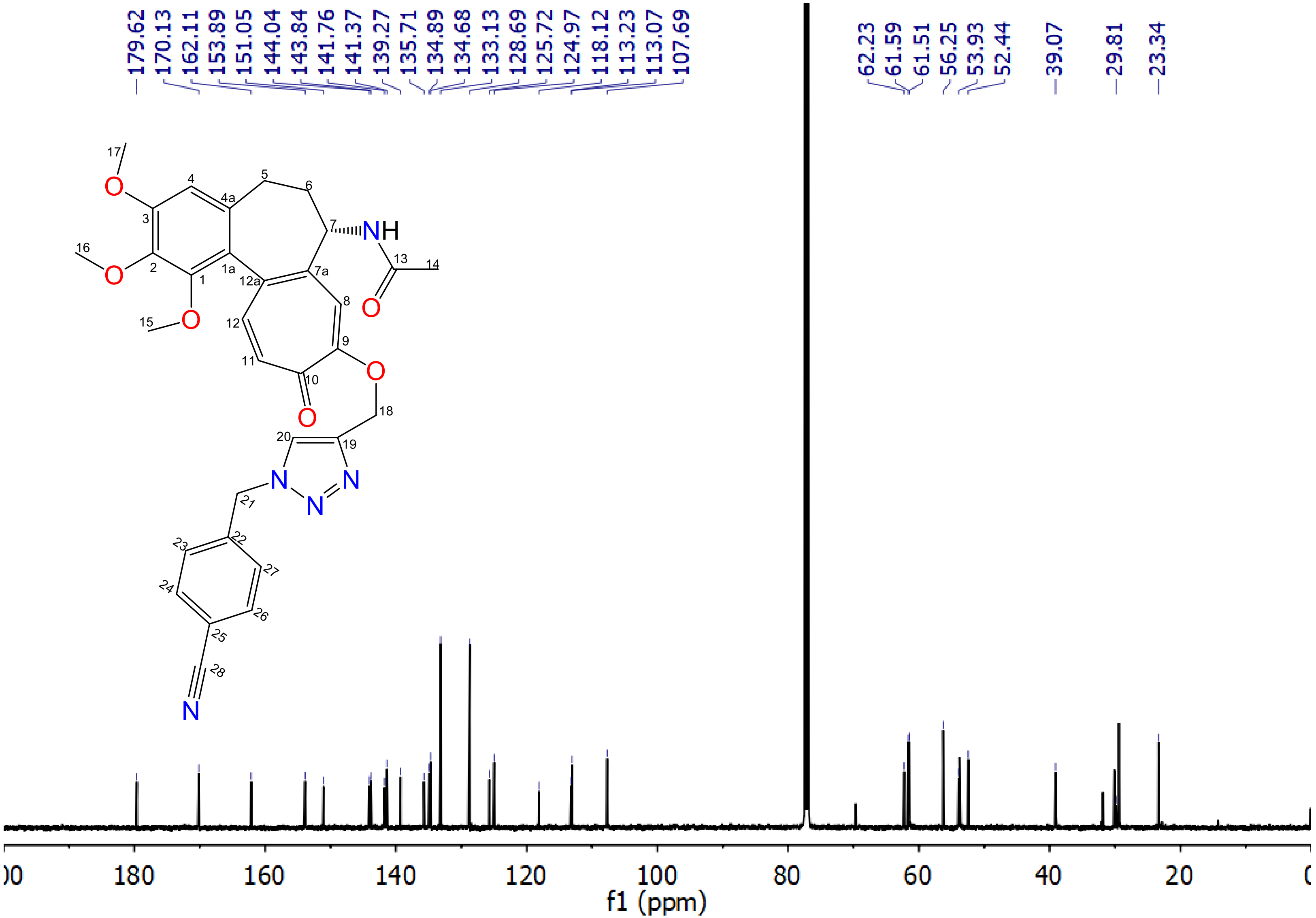

Figure 93S. ^{13}C NMR spectrum of compound **5b** in $CDCl_3$.

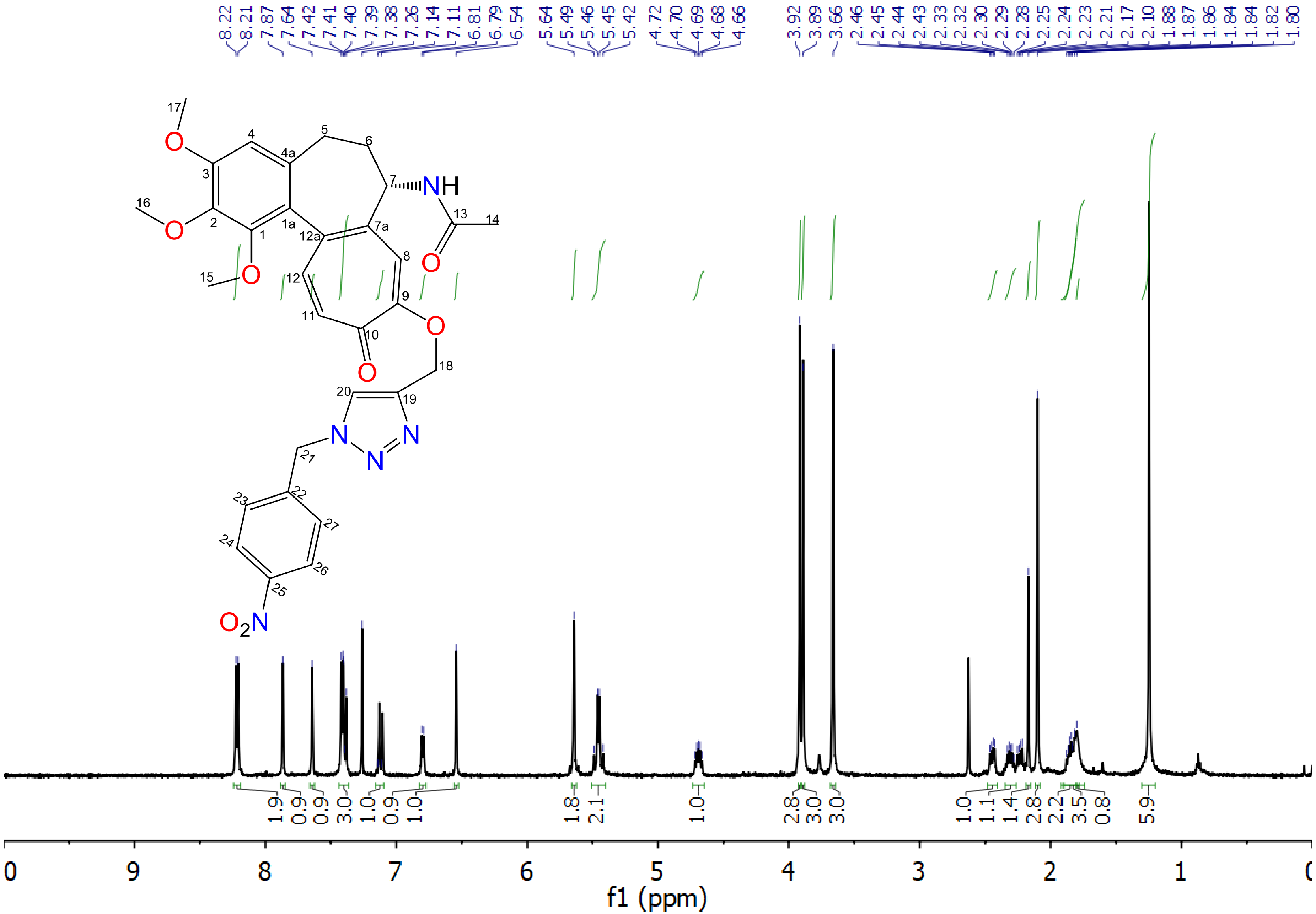

Figure 94S. ^{1}H NMR spectrum of compound **5c** in $CDCl_3$.

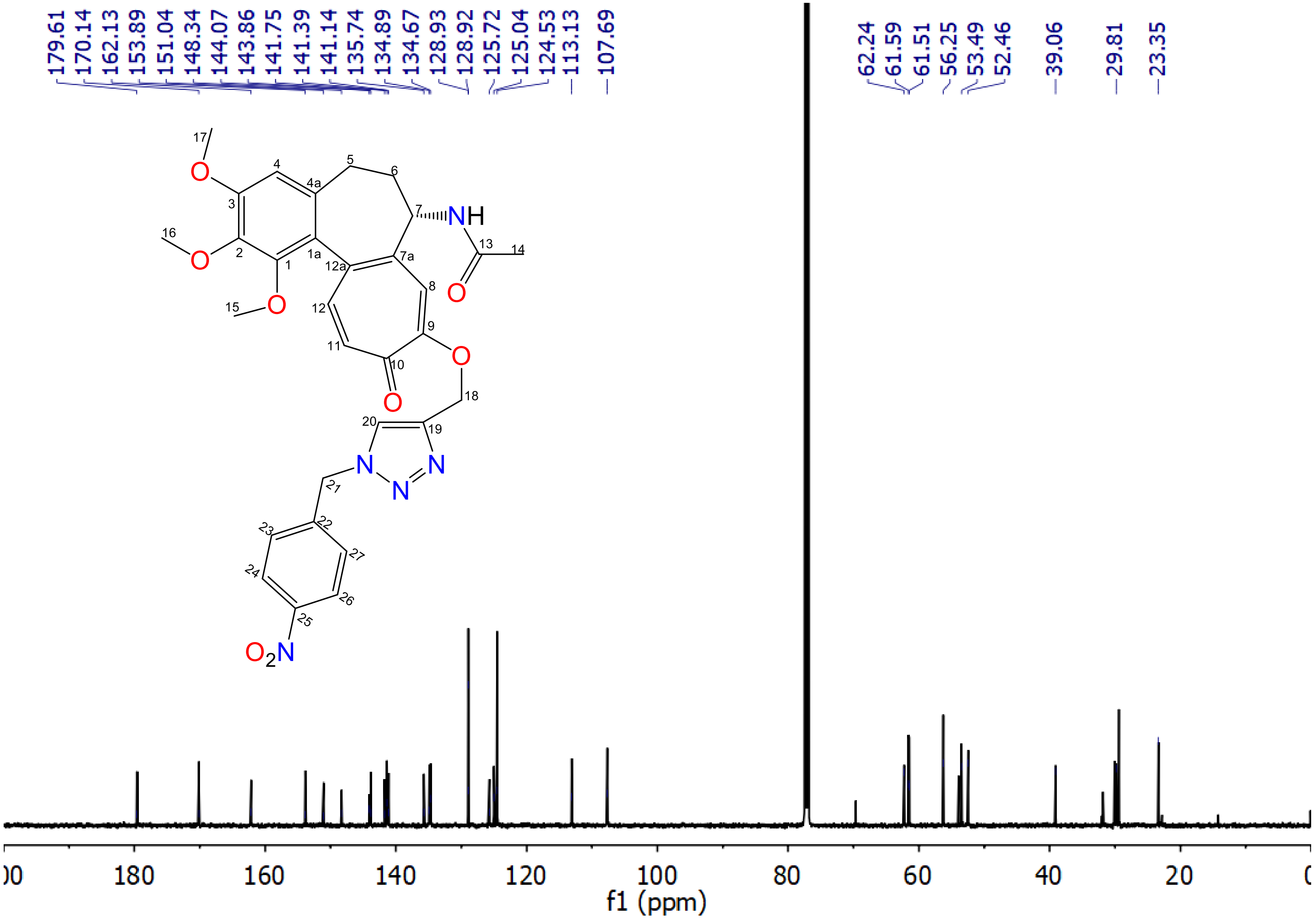

Figure 95S. ^{13}C NMR spectrum of compound **5c** in $CDCl_3$.

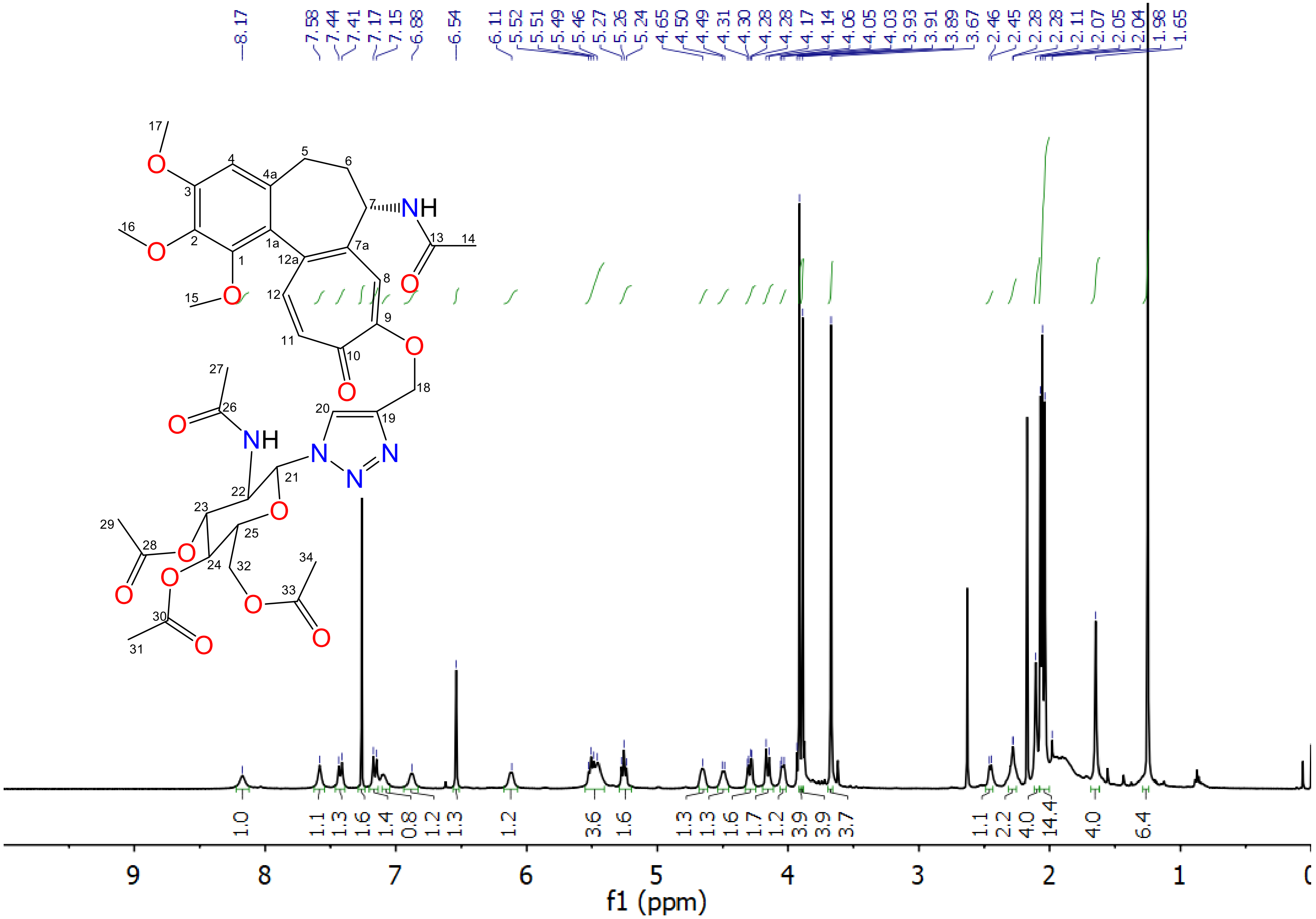

Figure 96S. ^{1}H NMR spectrum of compound **5d** in $CDCl_3$.

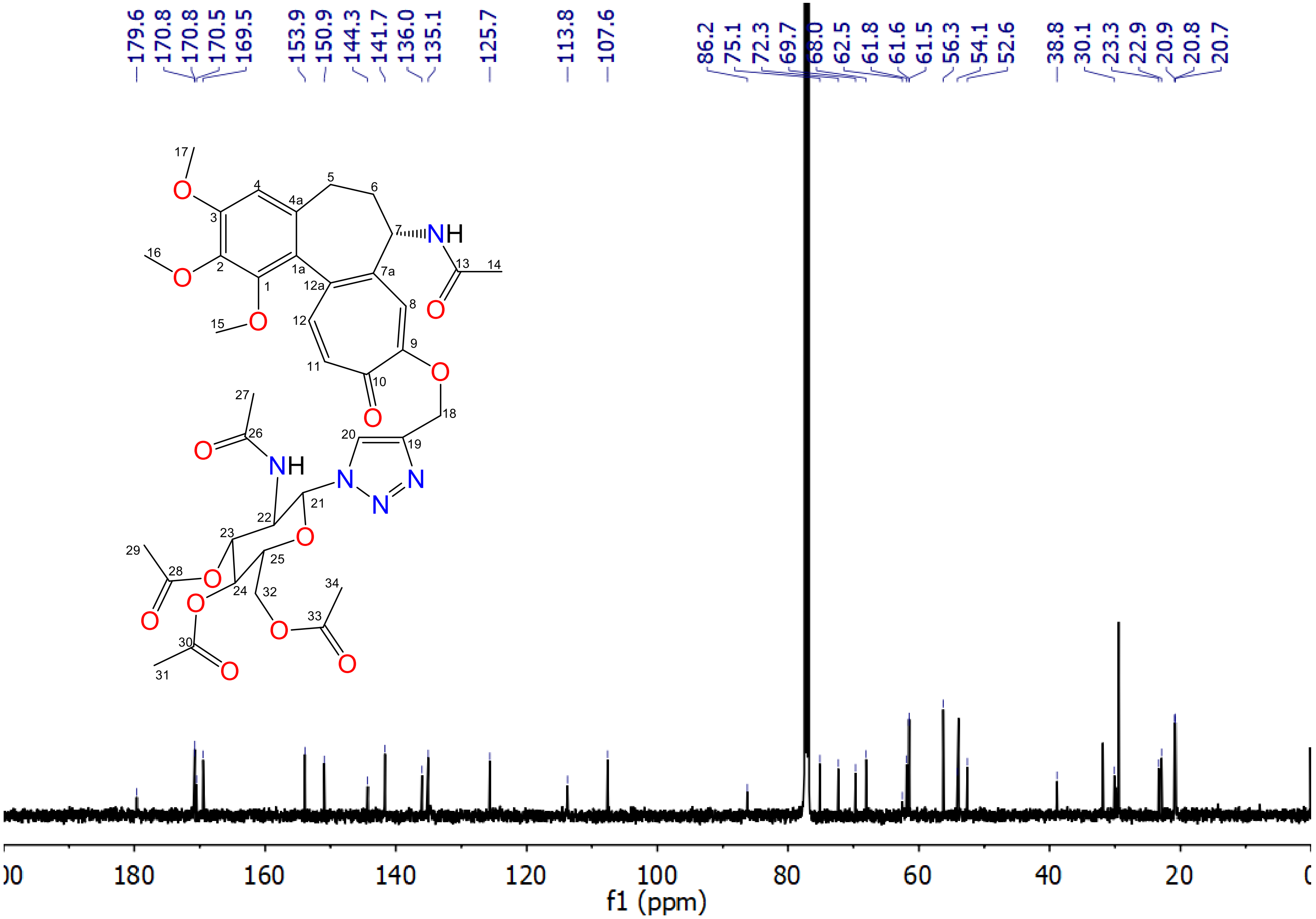

Figure 97S. ^{13}C NMR spectrum of compound **5d** in $CDCl_3$.

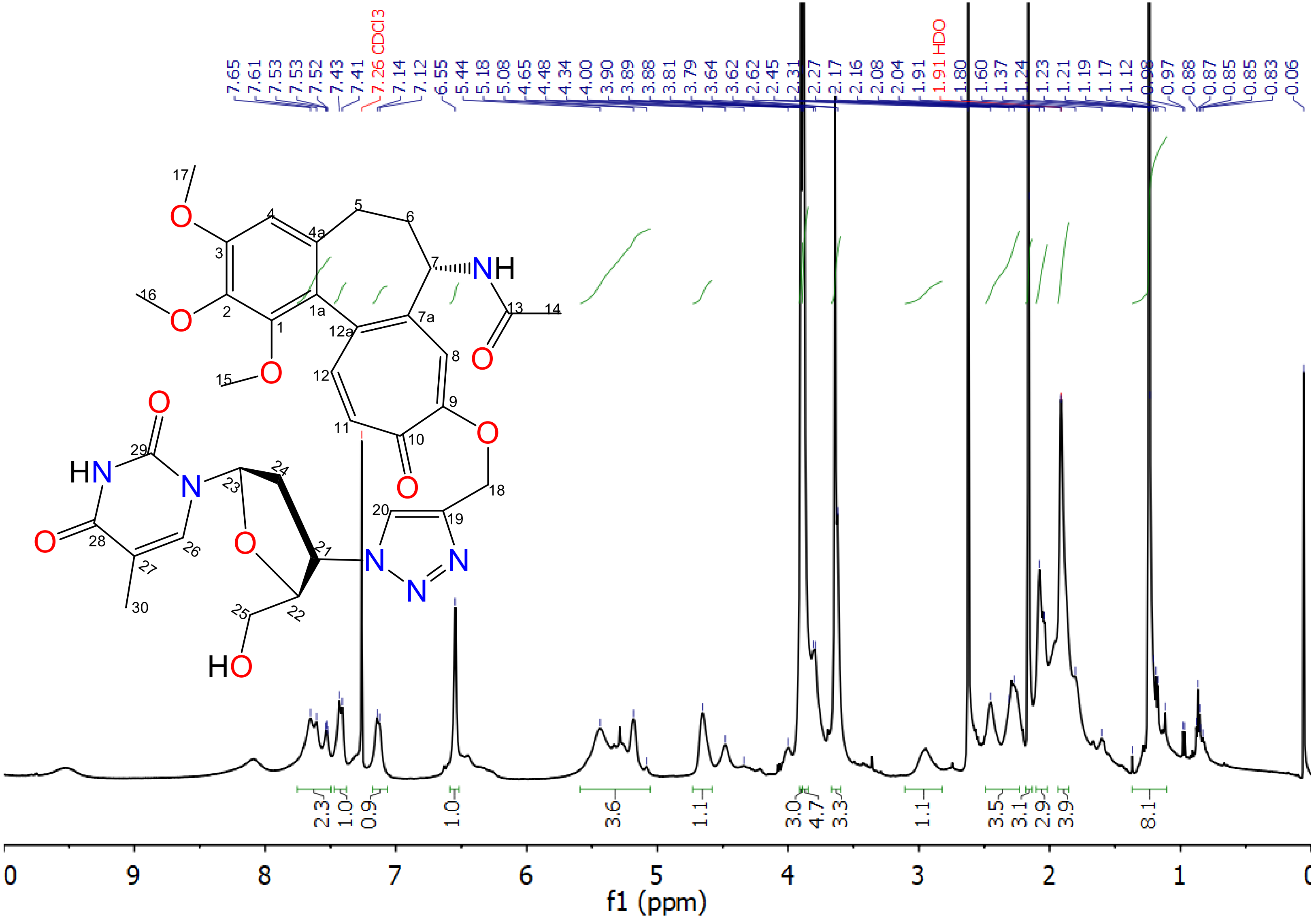

Figure 98S. ^{1}H NMR spectrum of compound **5e** in $CDCl_3$.

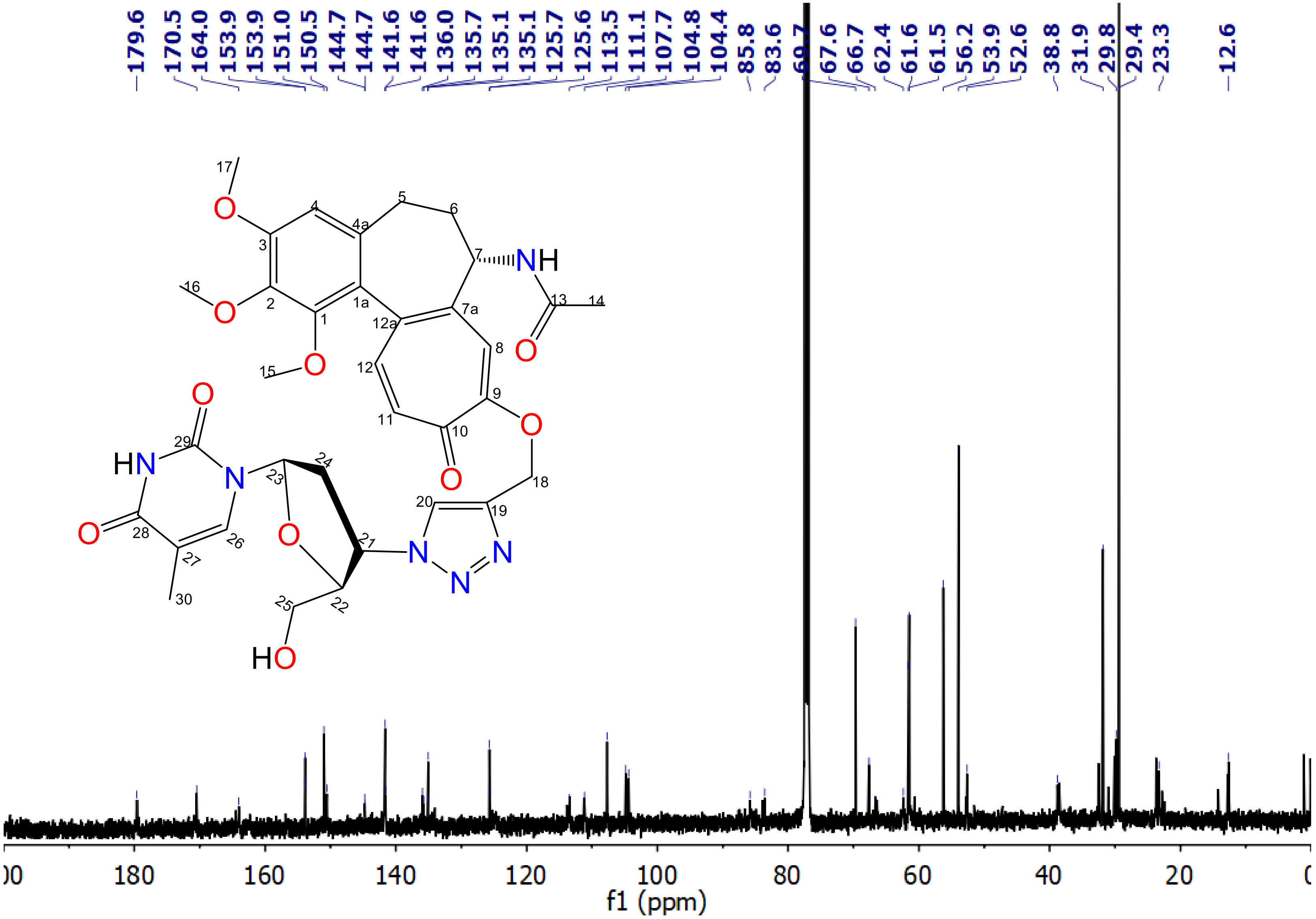

Figure 99S. ^{13}C NMR spectrum of compound **5e** in $CDCl_3$.

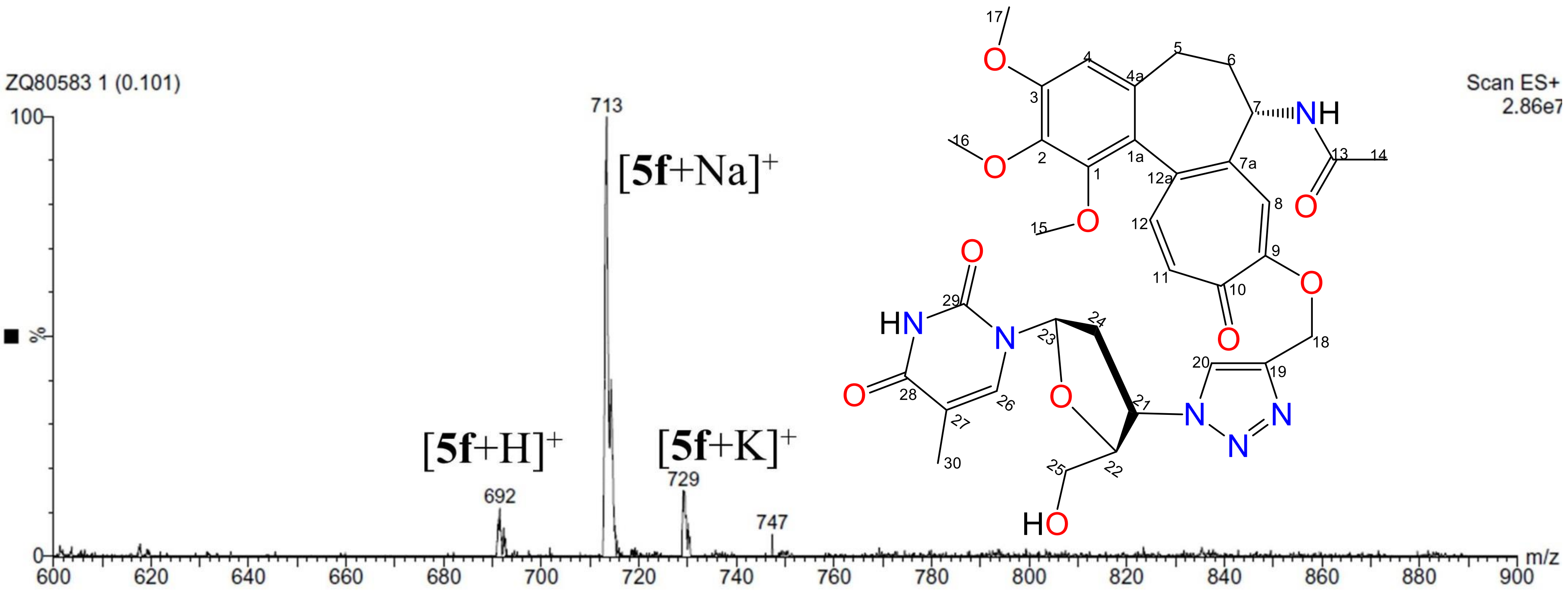

Figure 100S. ESI MS$^+$ spectrum of **5e**.

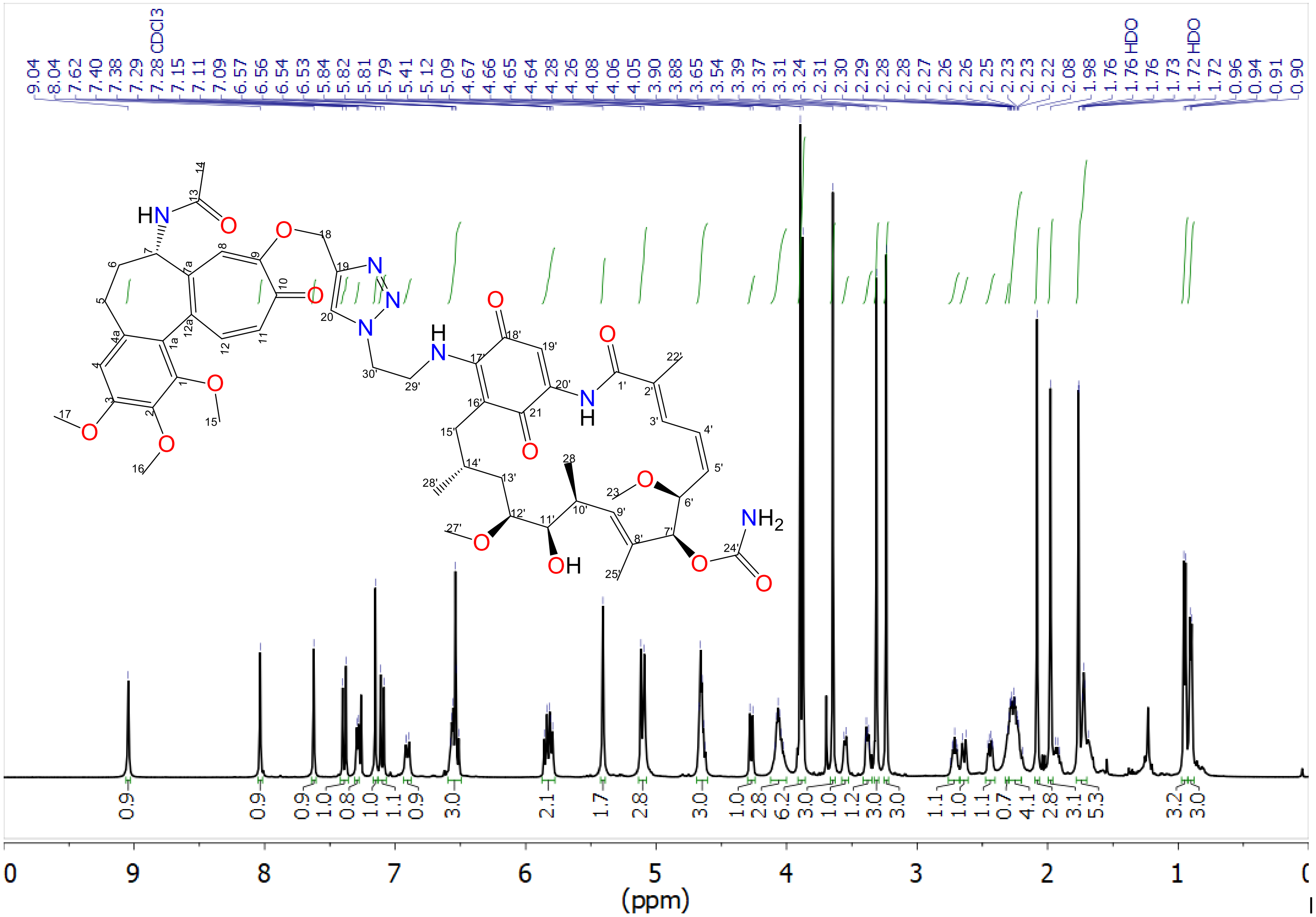

Figure 101S. ^{1}H NMR spectrum of compound **5f** in $CDCl_3$.

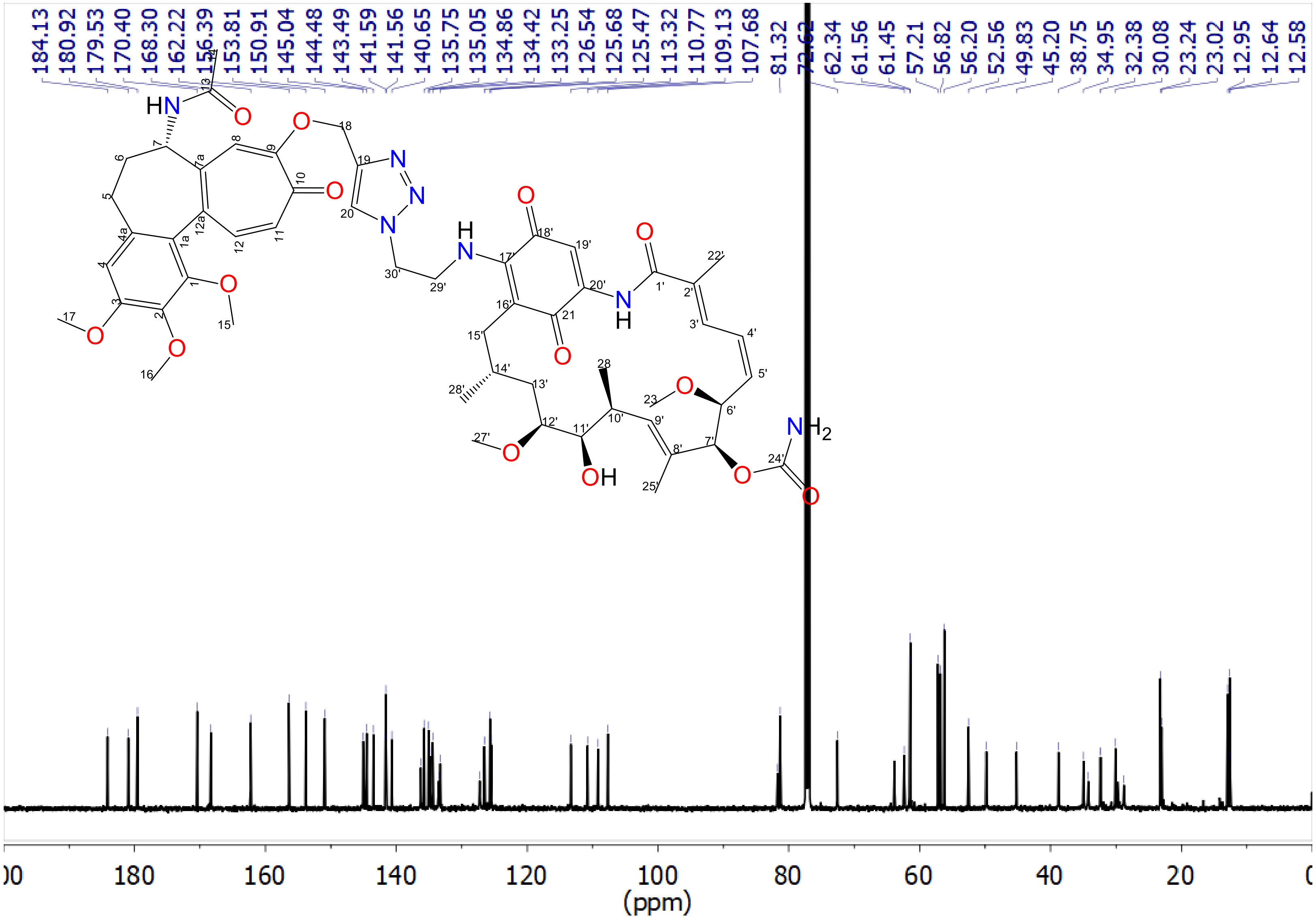

Figure 102S. ^{13}C NMR spectrum of compound **5f** in $CDCl_3$.

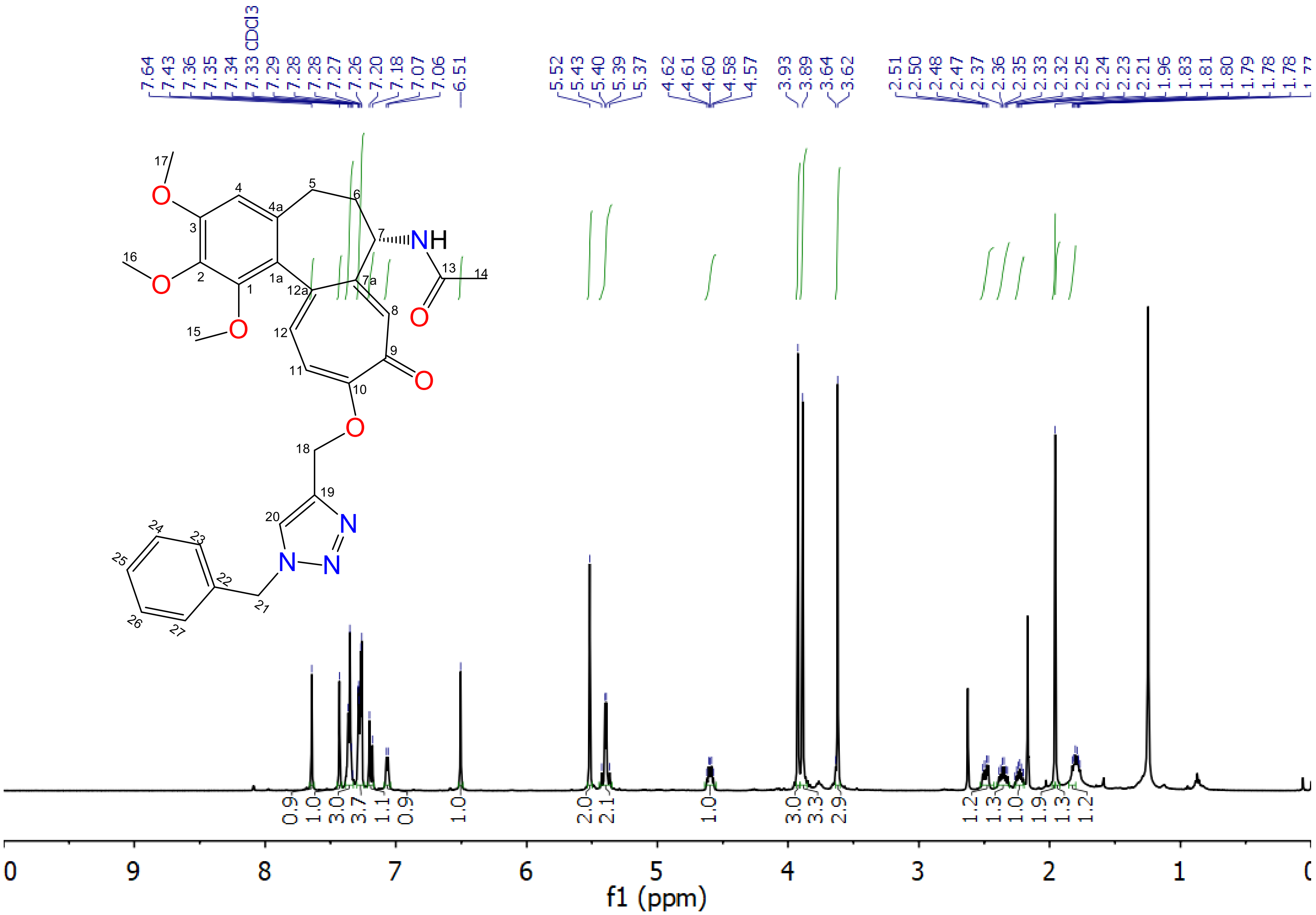

Figure 103S. ^{1}H NMR spectrum of compound **6a** in $CDCl_3$.

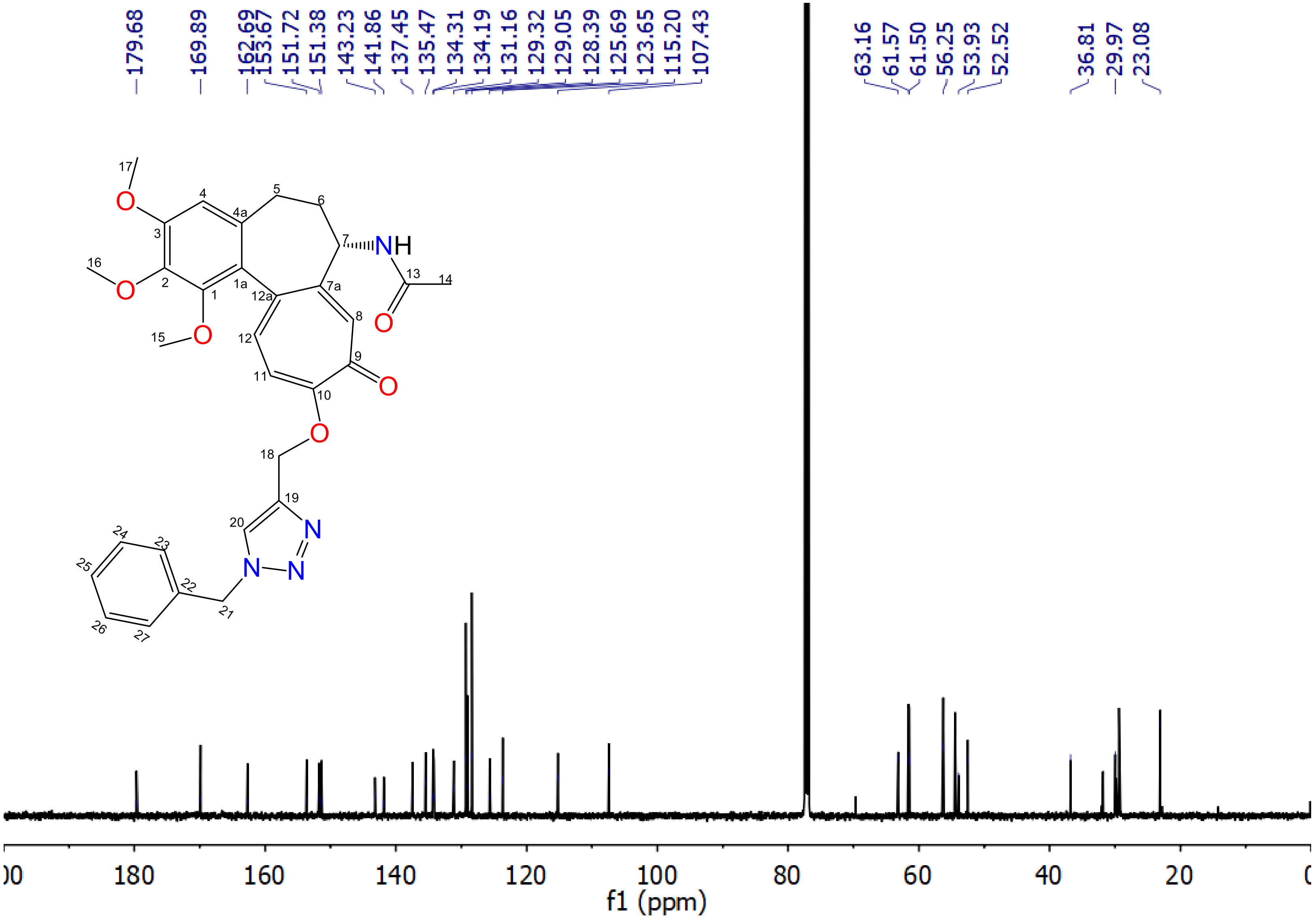

Figure 104S. ^{13}C NMR spectrum of compound **6a** in $CDCl_3$.

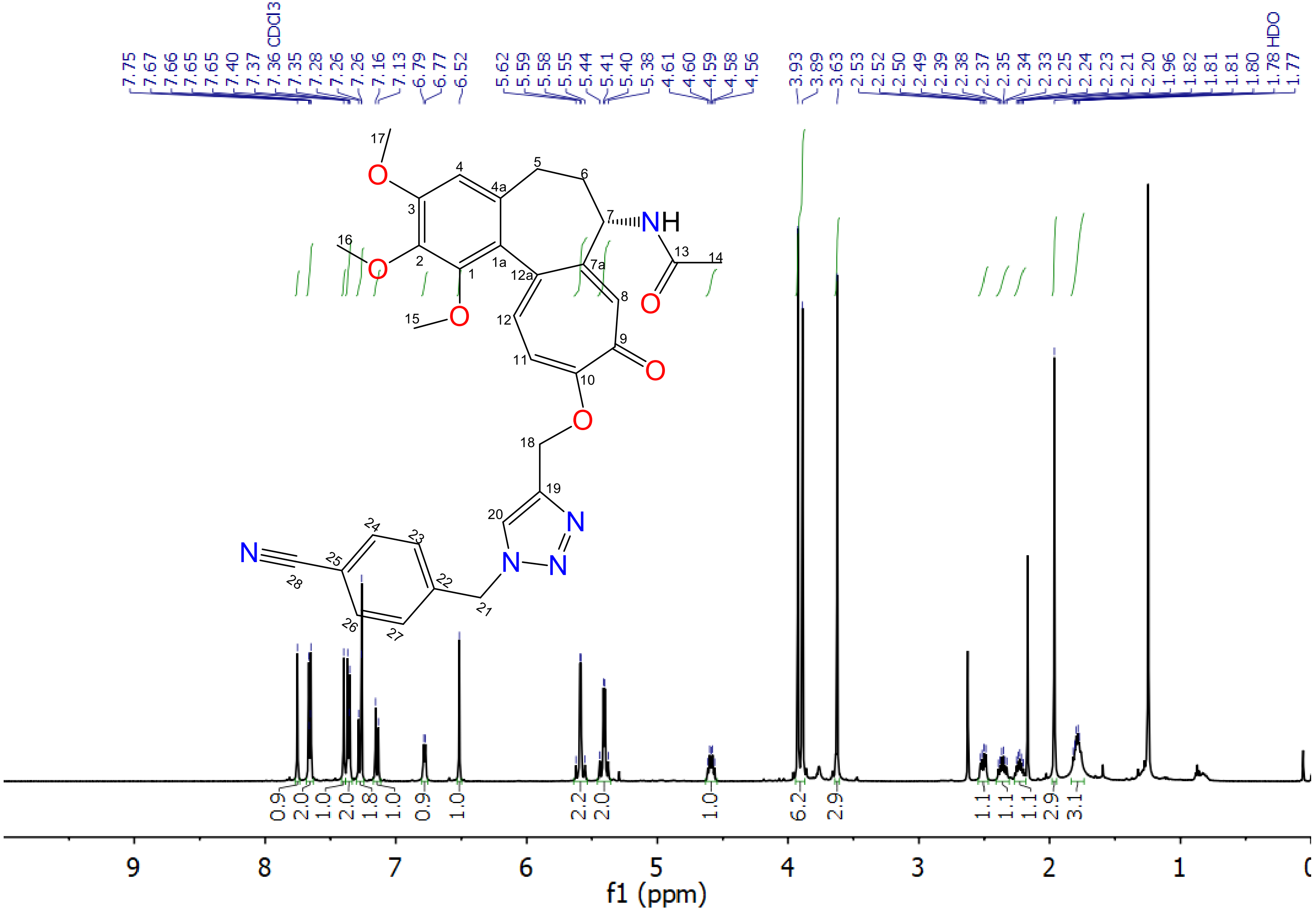

Figure 105S. ^{1}H NMR spectrum of compound **6b** in $CDCl_3$.

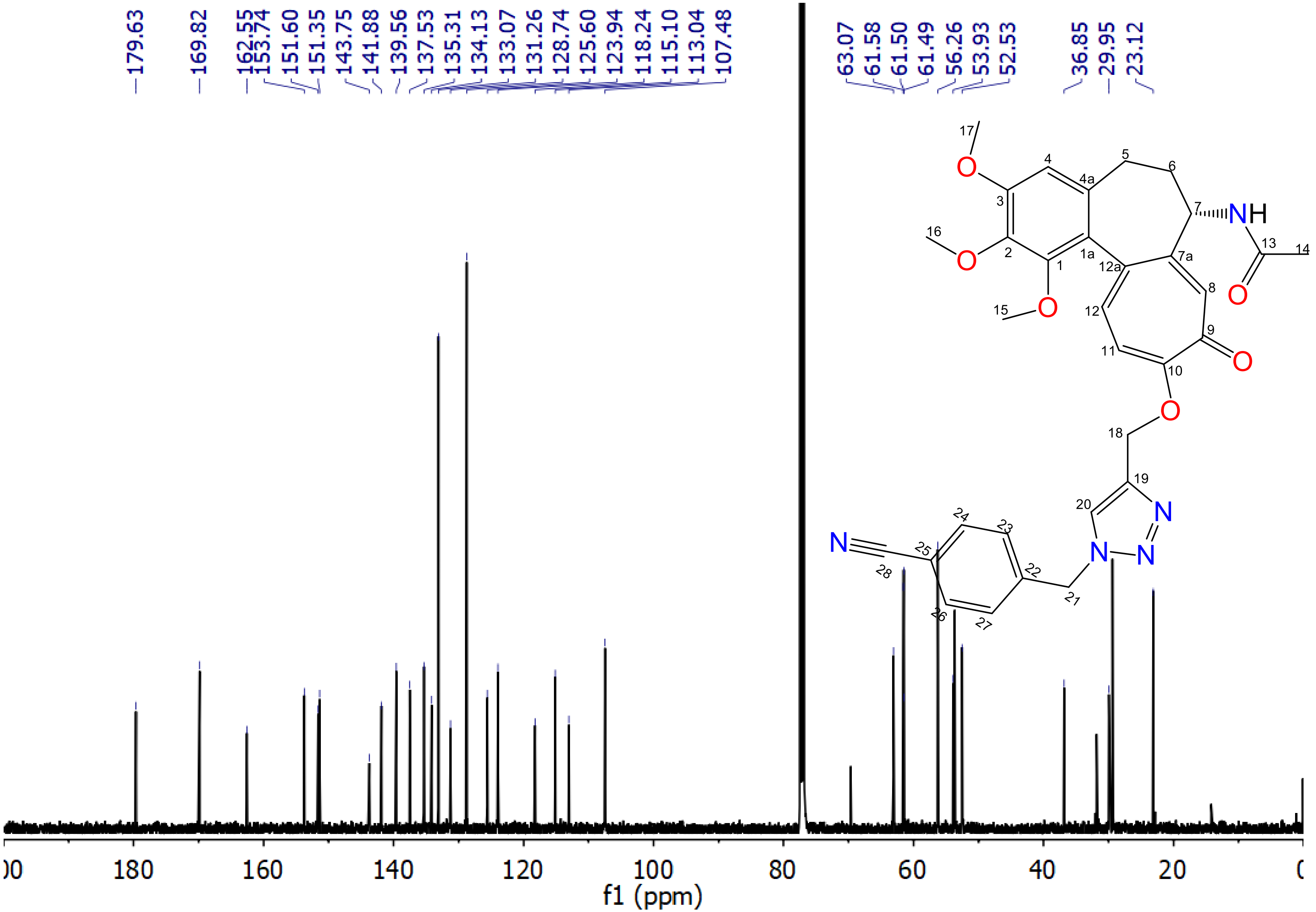

Figure 106S. ^{13}C NMR spectrum of compound **6b** in $CDCl_3$.

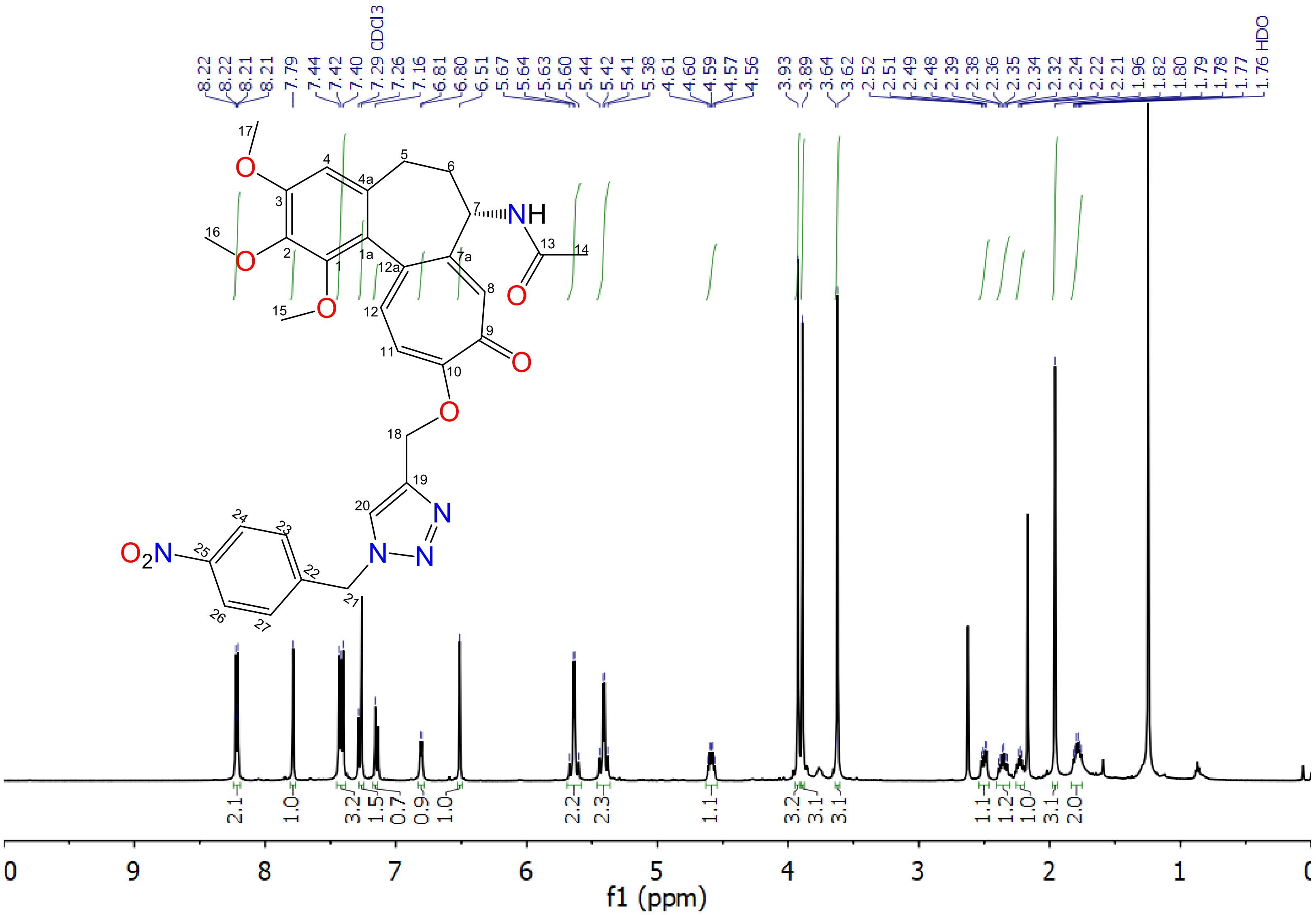

Figure 107S. ^{1}H NMR spectrum of compound **6c** in $CDCl_3$.

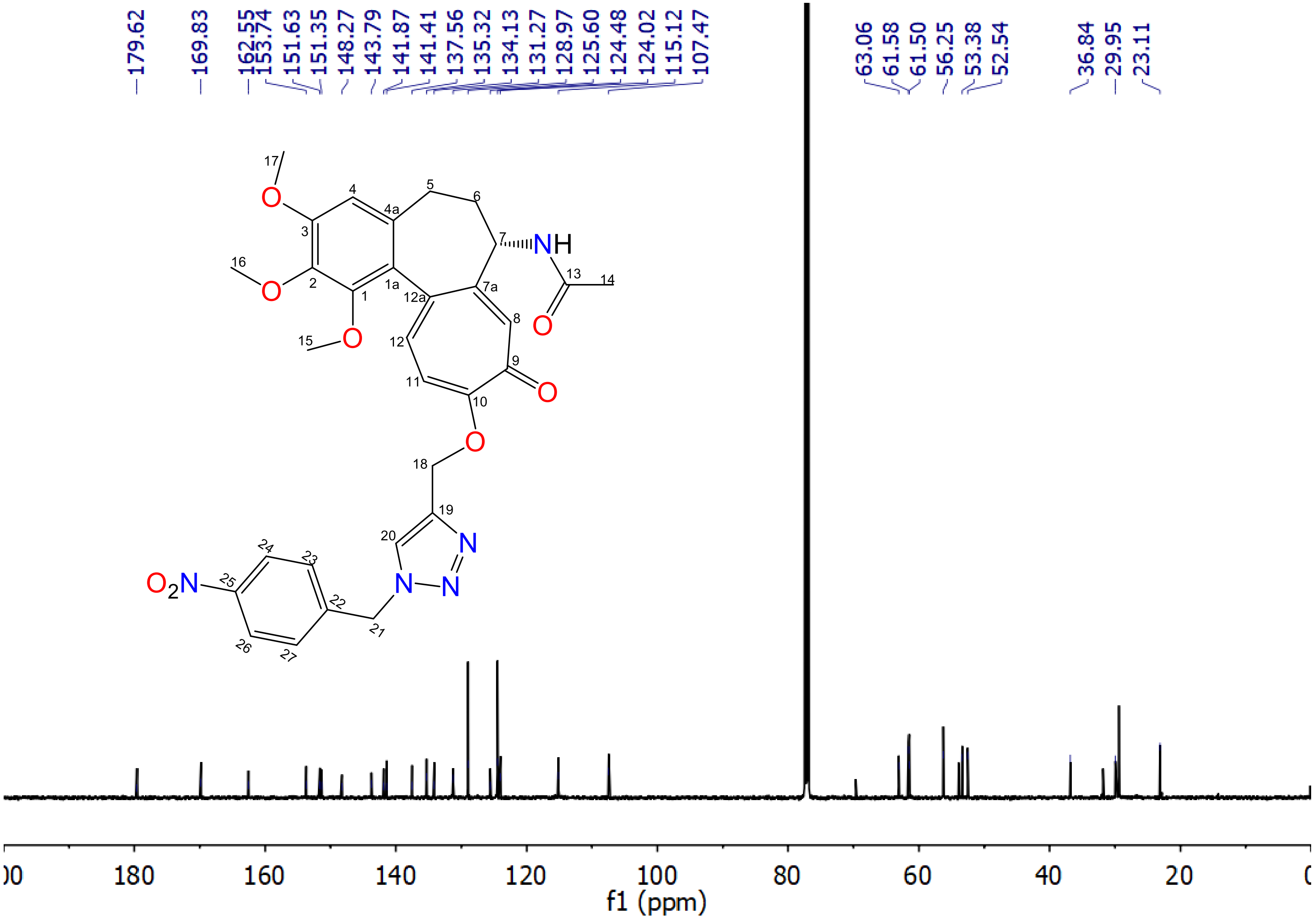

Figure 108S. ^{13}C NMR spectrum of compound **6c** in $CDCl_3$.

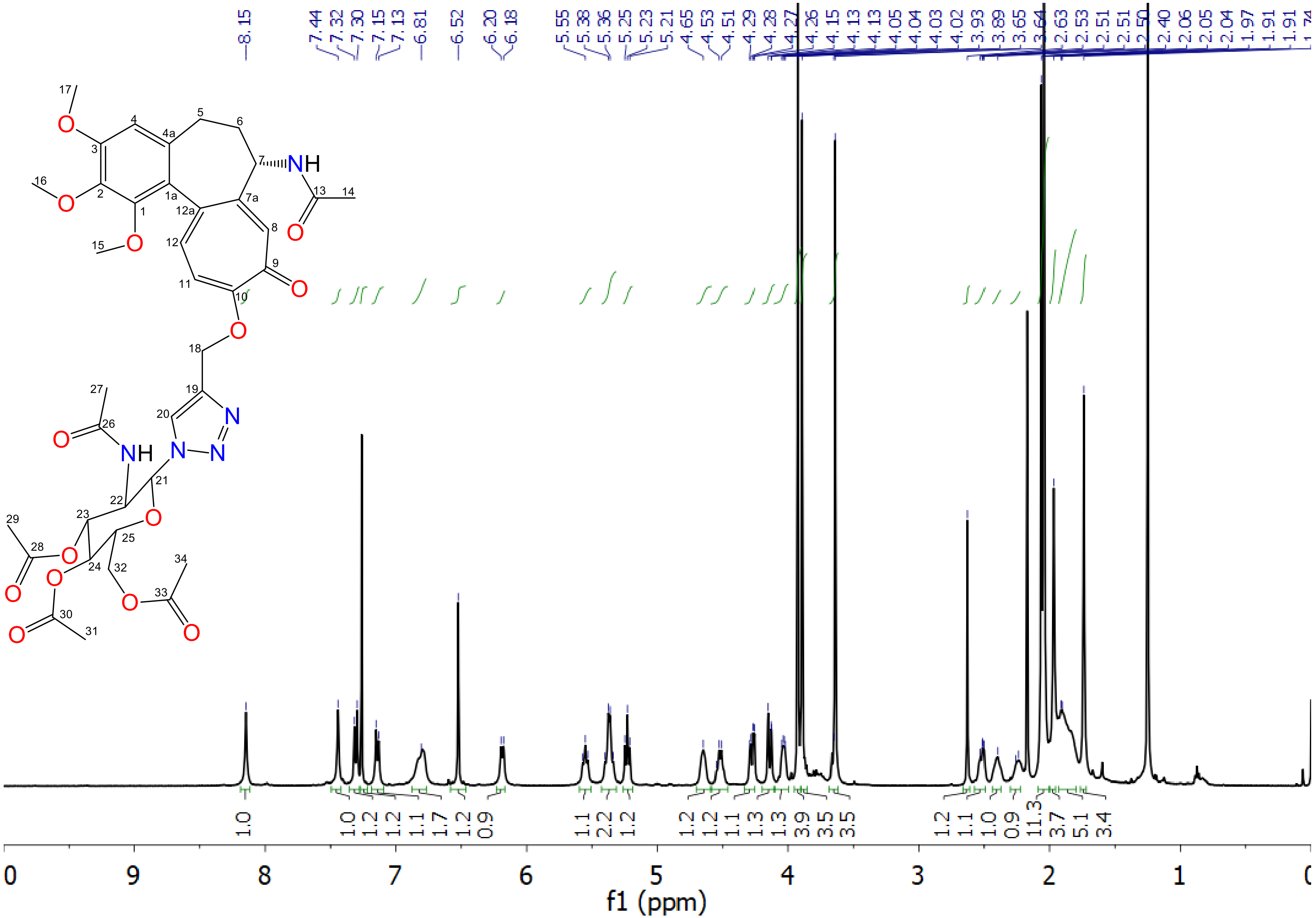

Figure 109S. ^{1}H NMR spectrum of compound **6d** in $CDCl_3$.

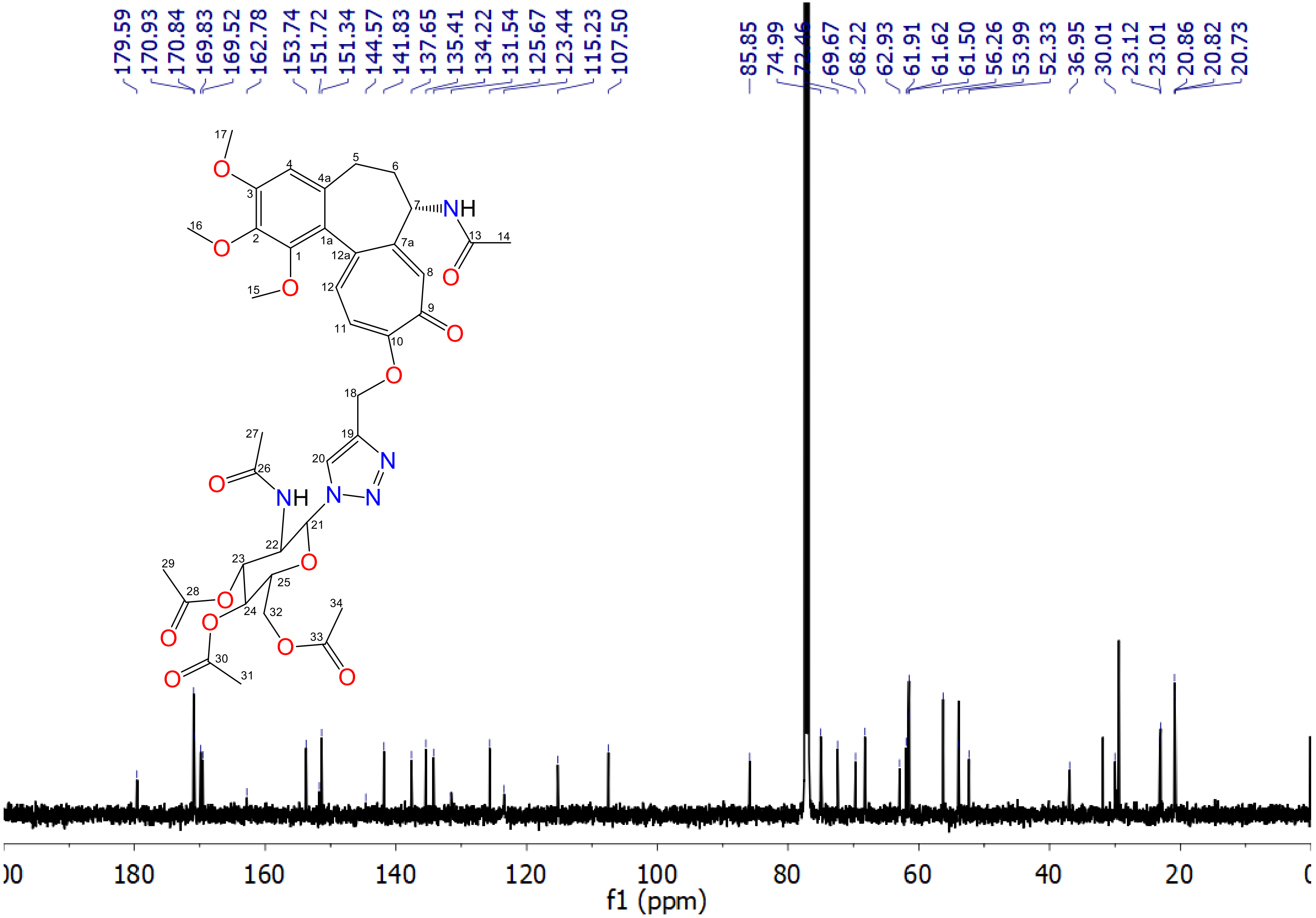

Figure 110S. ^{13}C NMR spectrum of compound **6d** in $CDCl_3$.

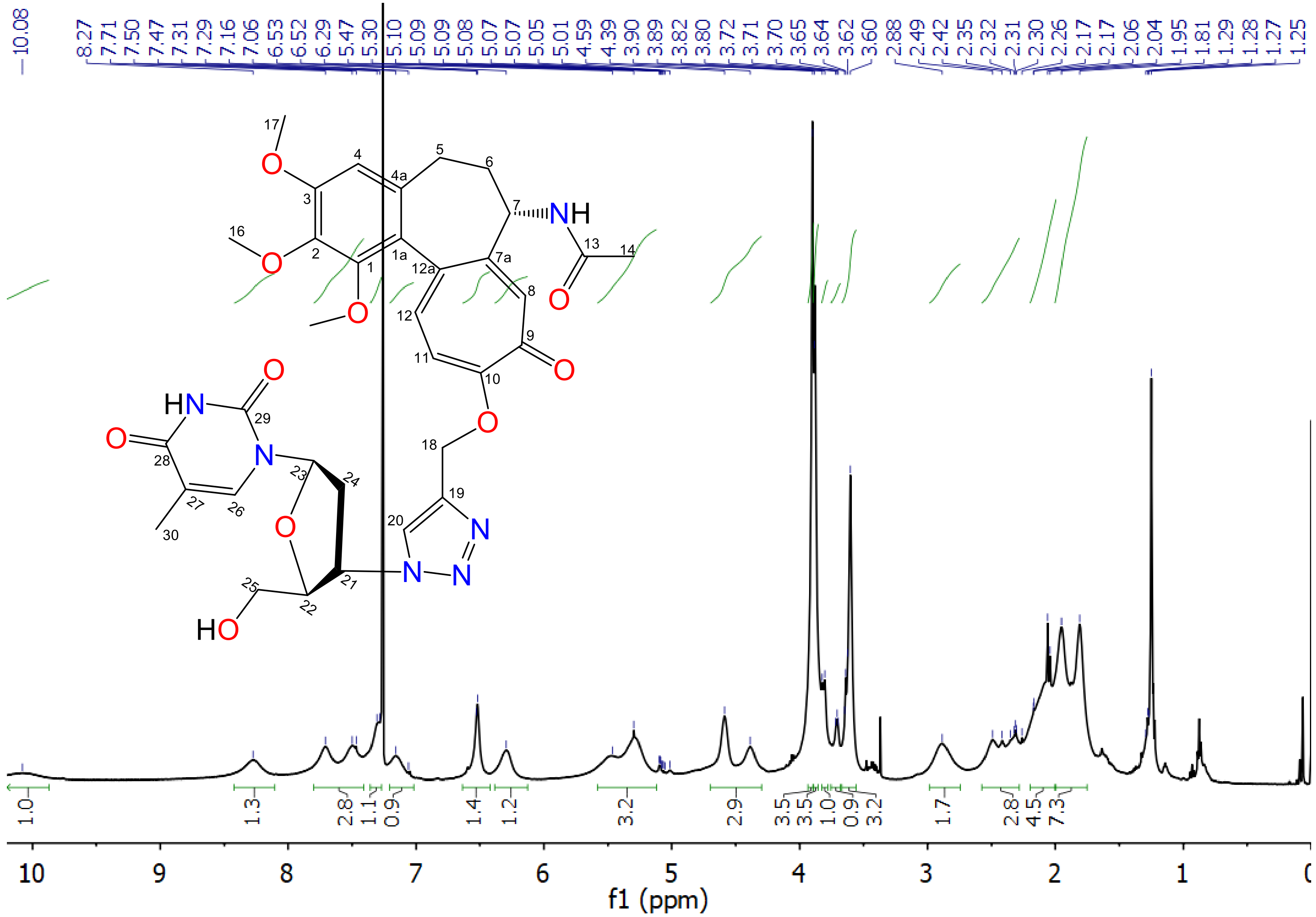

Figure 111S. ^{1}H NMR spectrum of compound **6e** in $CDCl_3$.

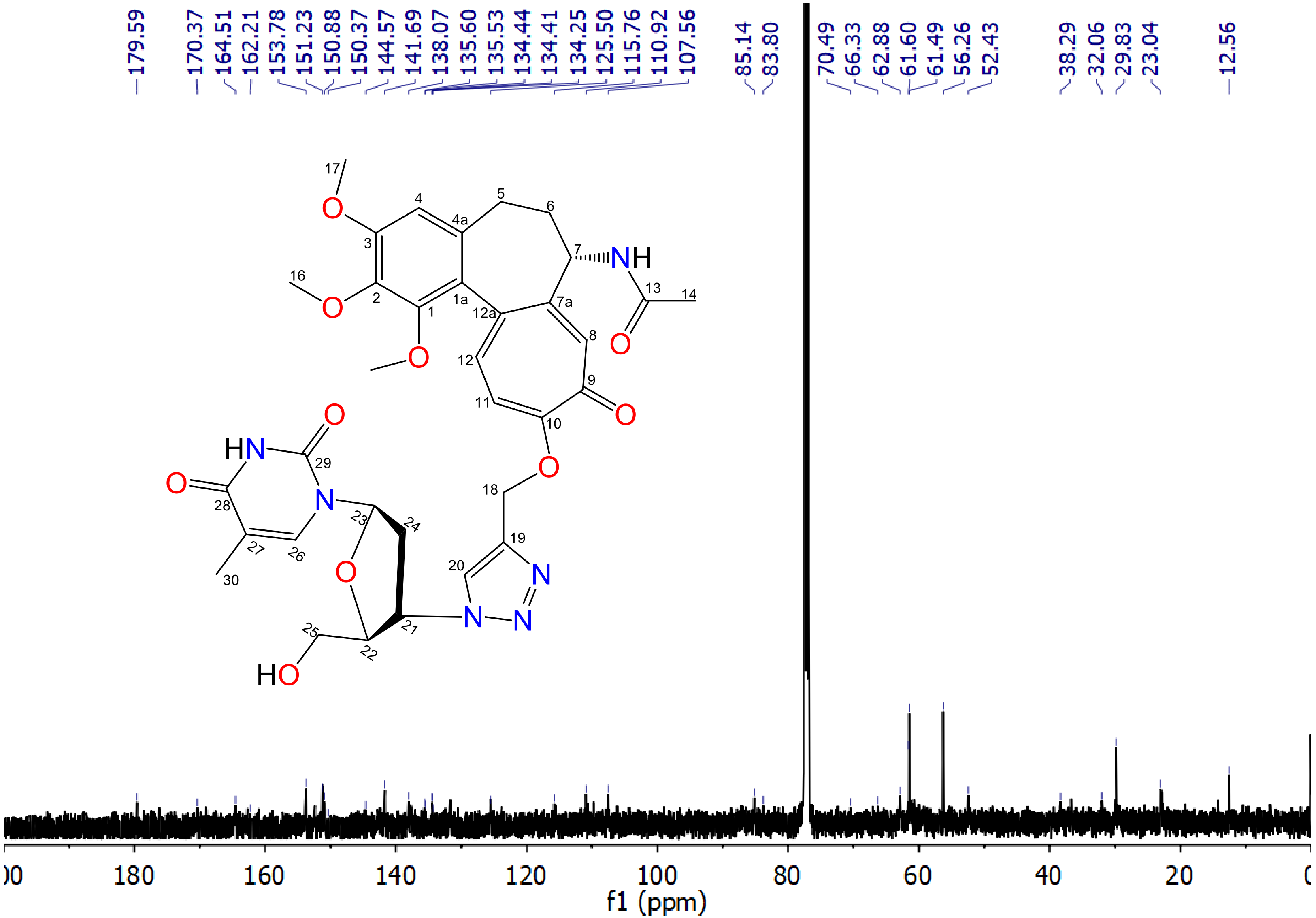

Figure 112S. ^{13}C NMR spectrum of compound **6e** in $CDCl_3$.

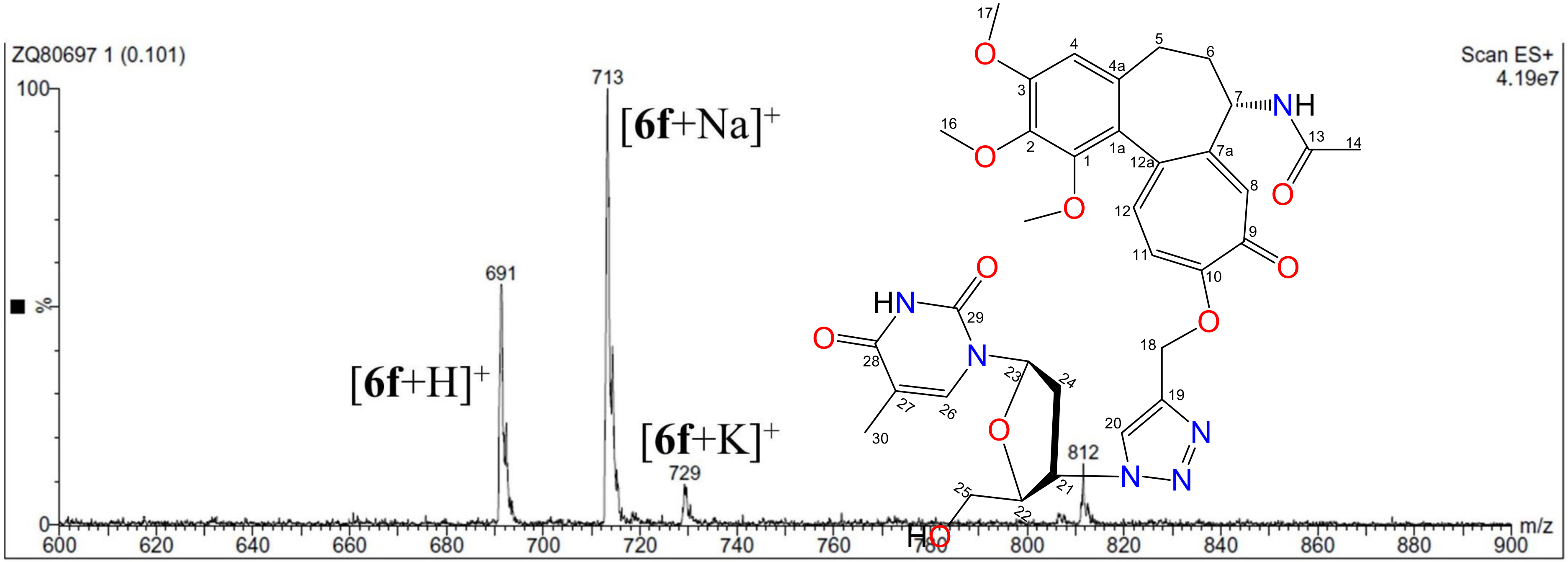

Figure 113S. ESI MS^{+} spectrum of **6e**.

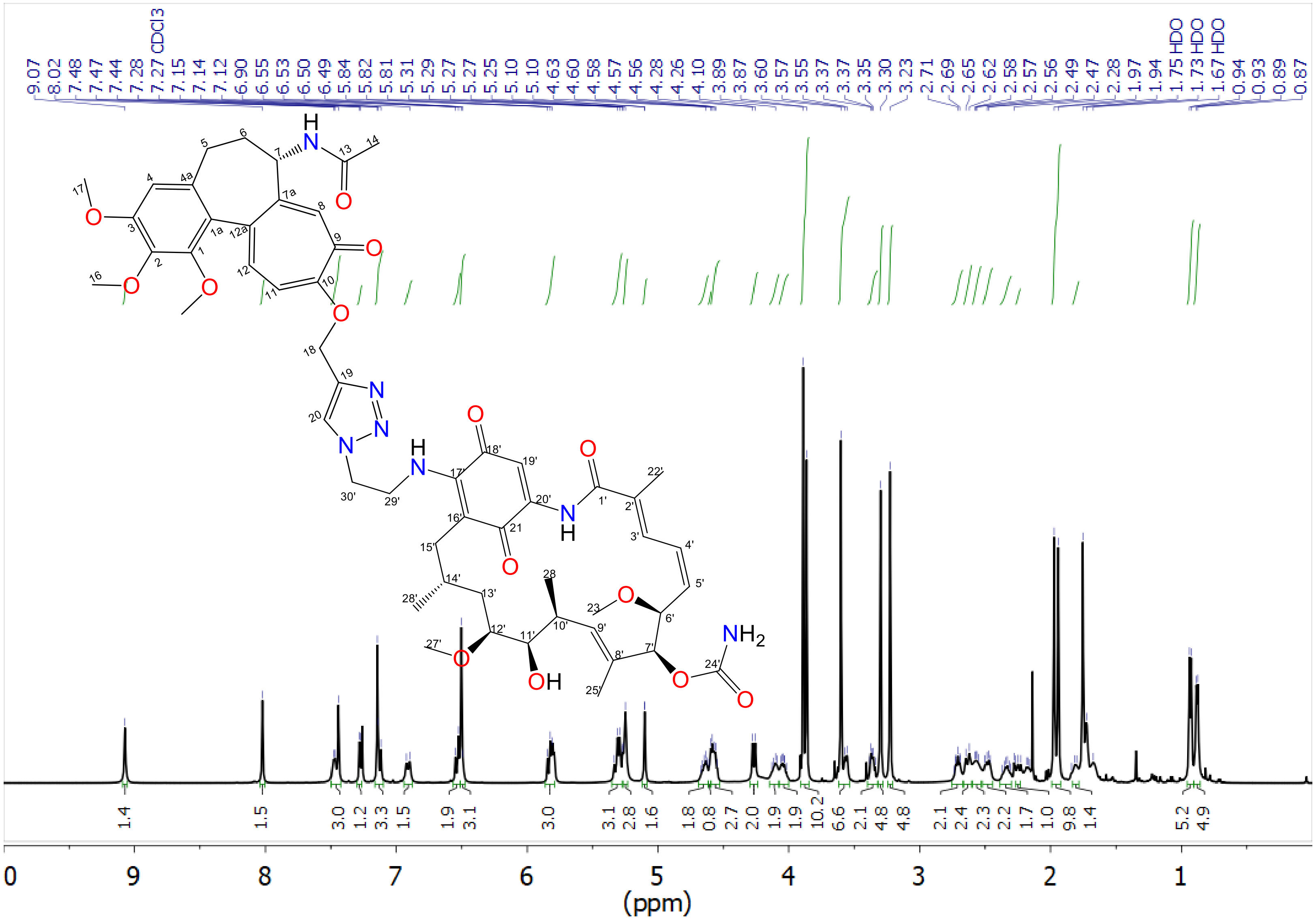

Figure 114S. ^{1}H NMR spectrum of compound **6f** in $CDCl_3$.

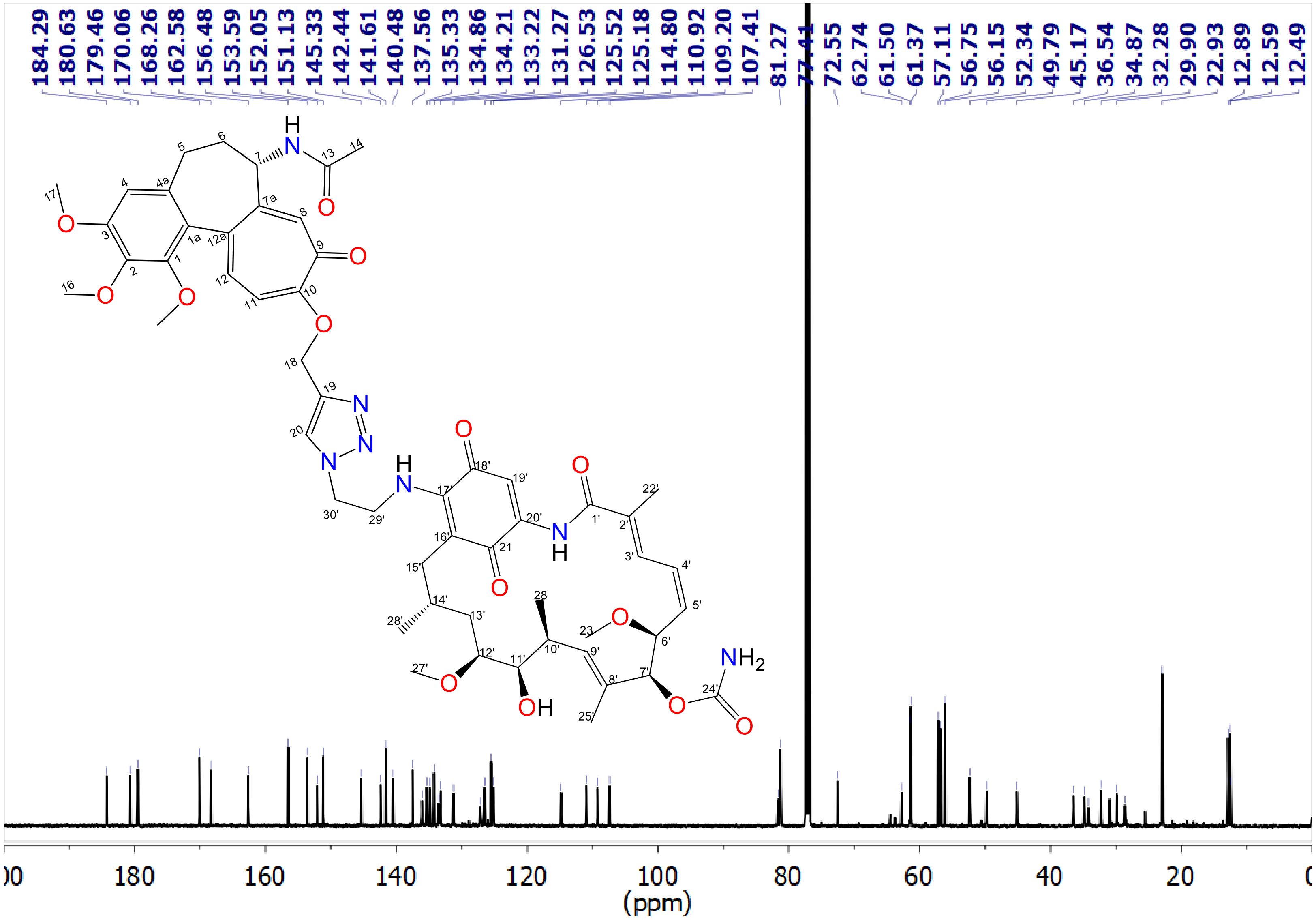

Figure 115S. ^{13}C NMR spectrum of compound **6f** in $CDCl_3$.

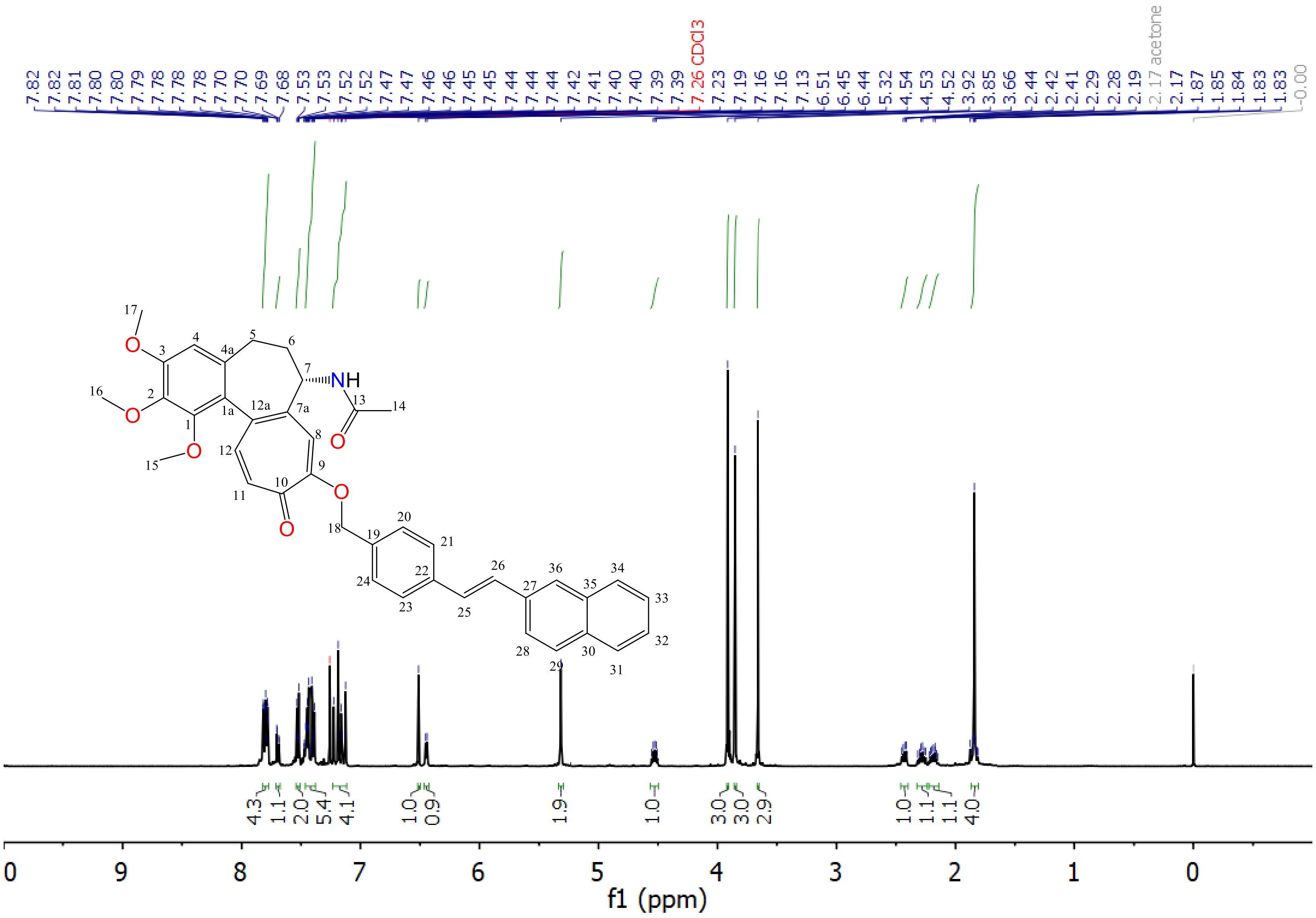

Figure 116S. ^1H NMR spectrum of compound **7a** in $CDCl_3$.

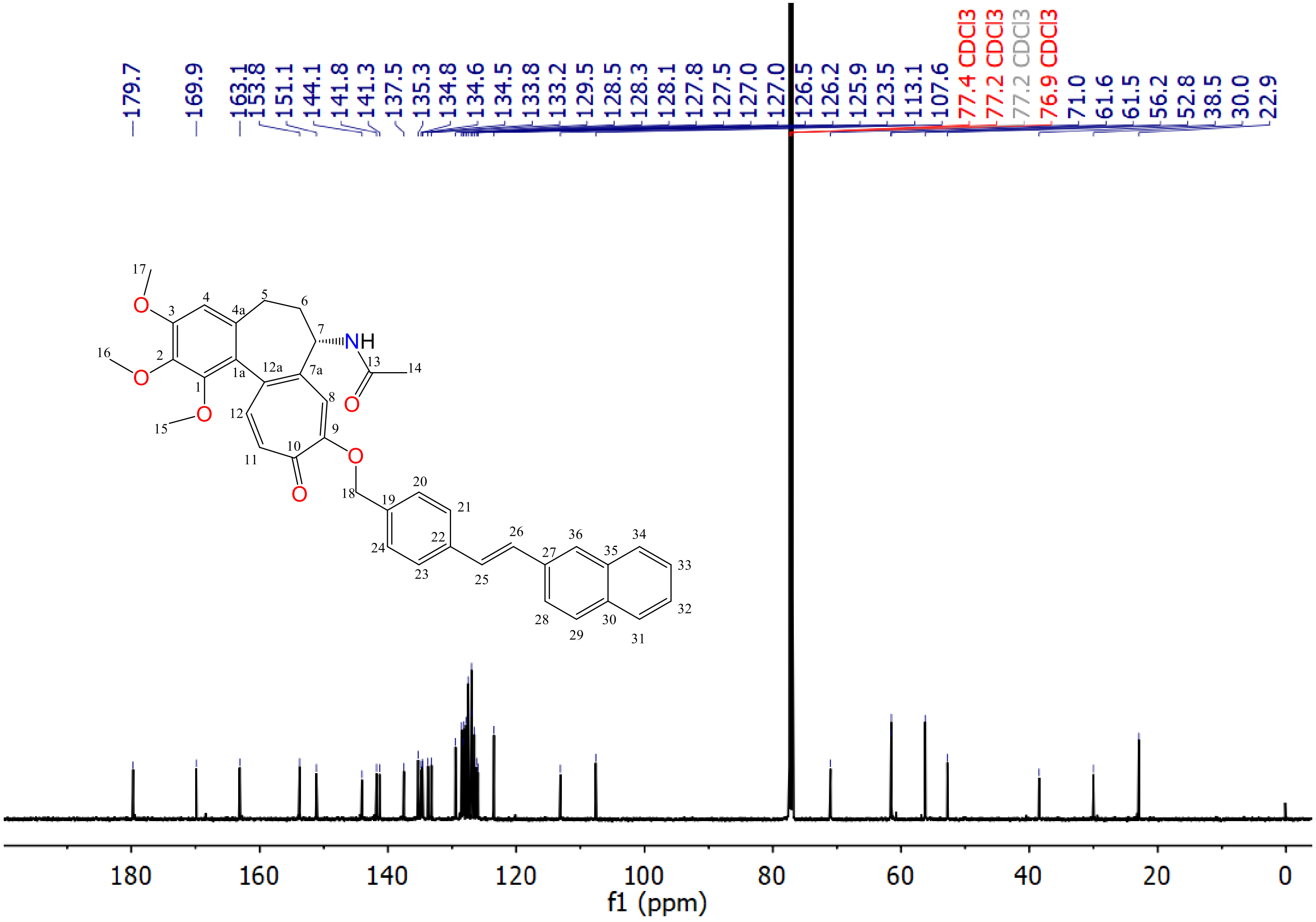

Figure 117S. ^{13}C NMR spectrum of compound **7a** in $CDCl_3$.

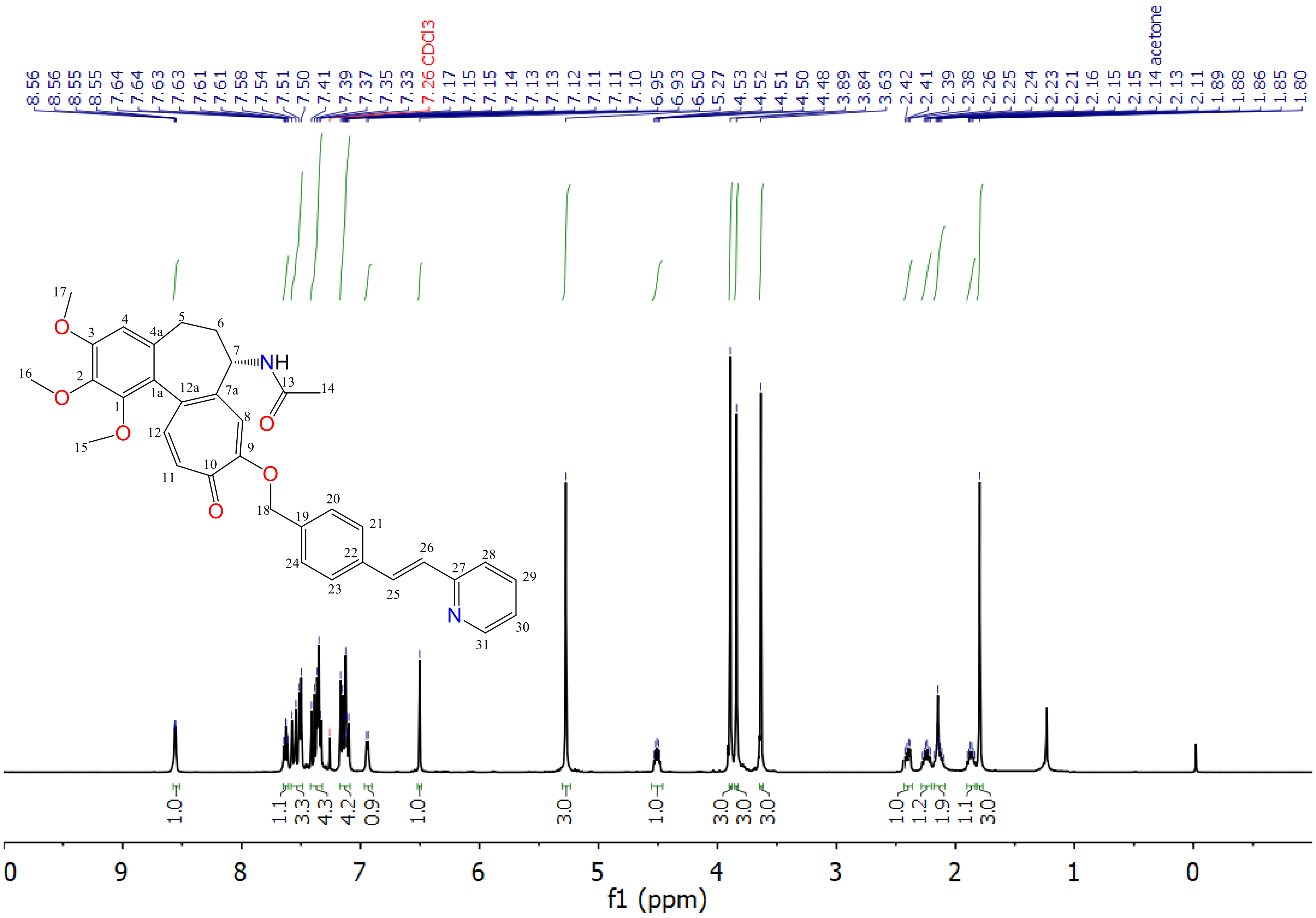

Figure 118S. ^{1}H NMR spectrum of compound **7b** in $CDCl_3$.

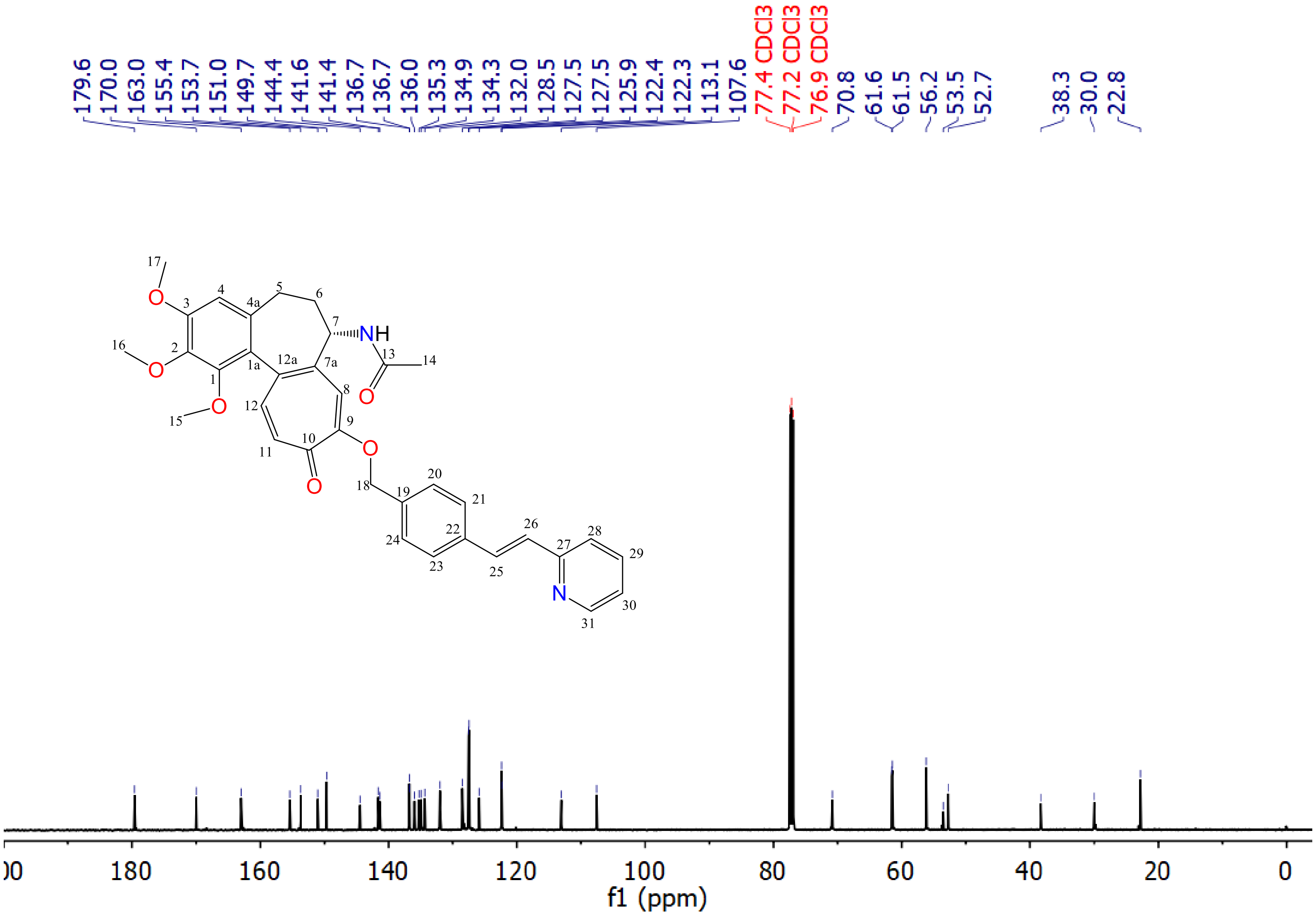

Figure 119S. ^{13}C NMR spectrum of compound **7b** in $CDCl_3$.

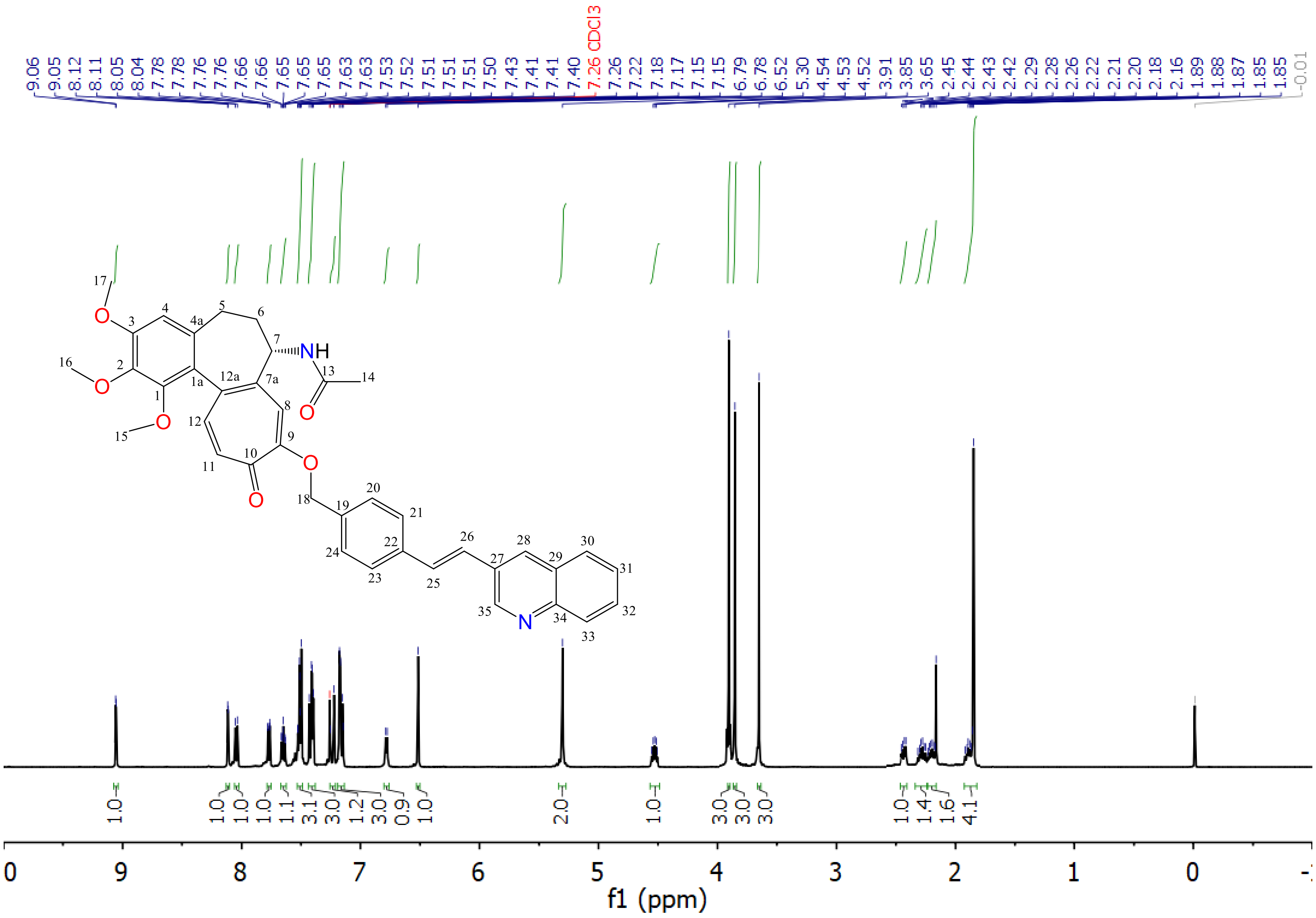

Figure 120S. ^{1}H NMR spectrum of compound **7c** in $CDCl_3$.

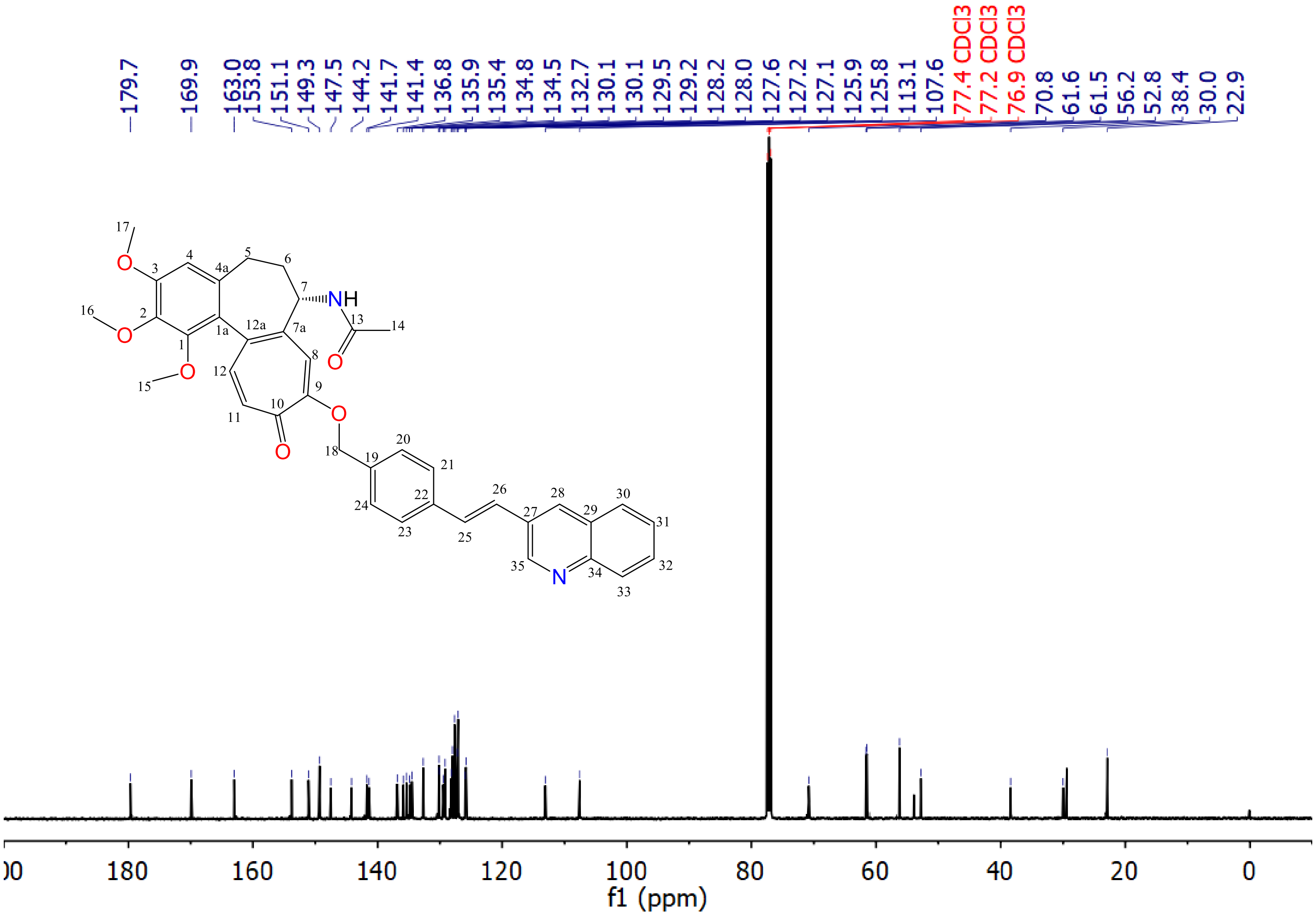

Figure 121S. ^{13}C NMR spectrum of compound **7c** in $CDCl_3$.

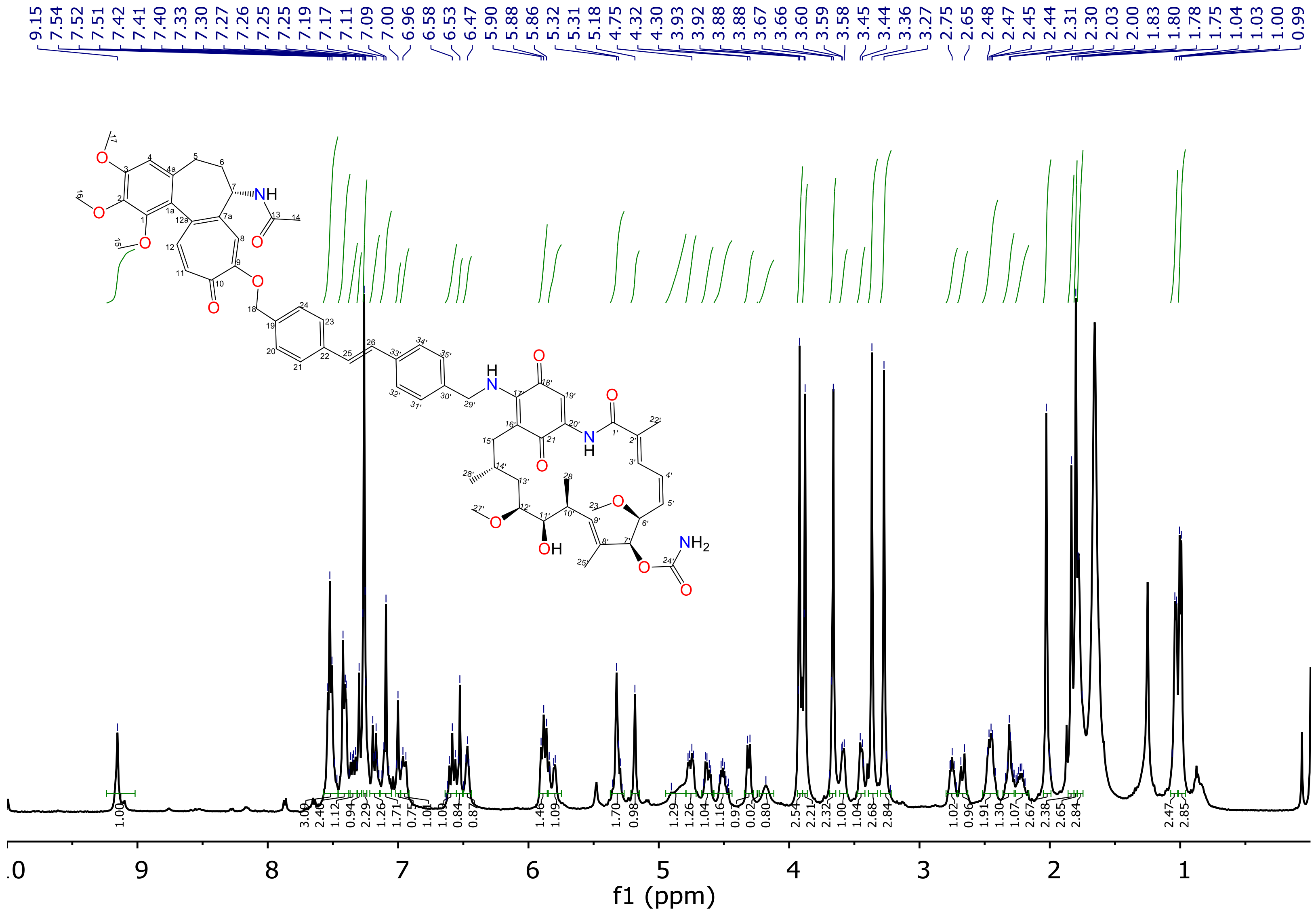

Figure 122S. ^{1}H NMR spectrum of compound **7d** in $CDCl_3$.

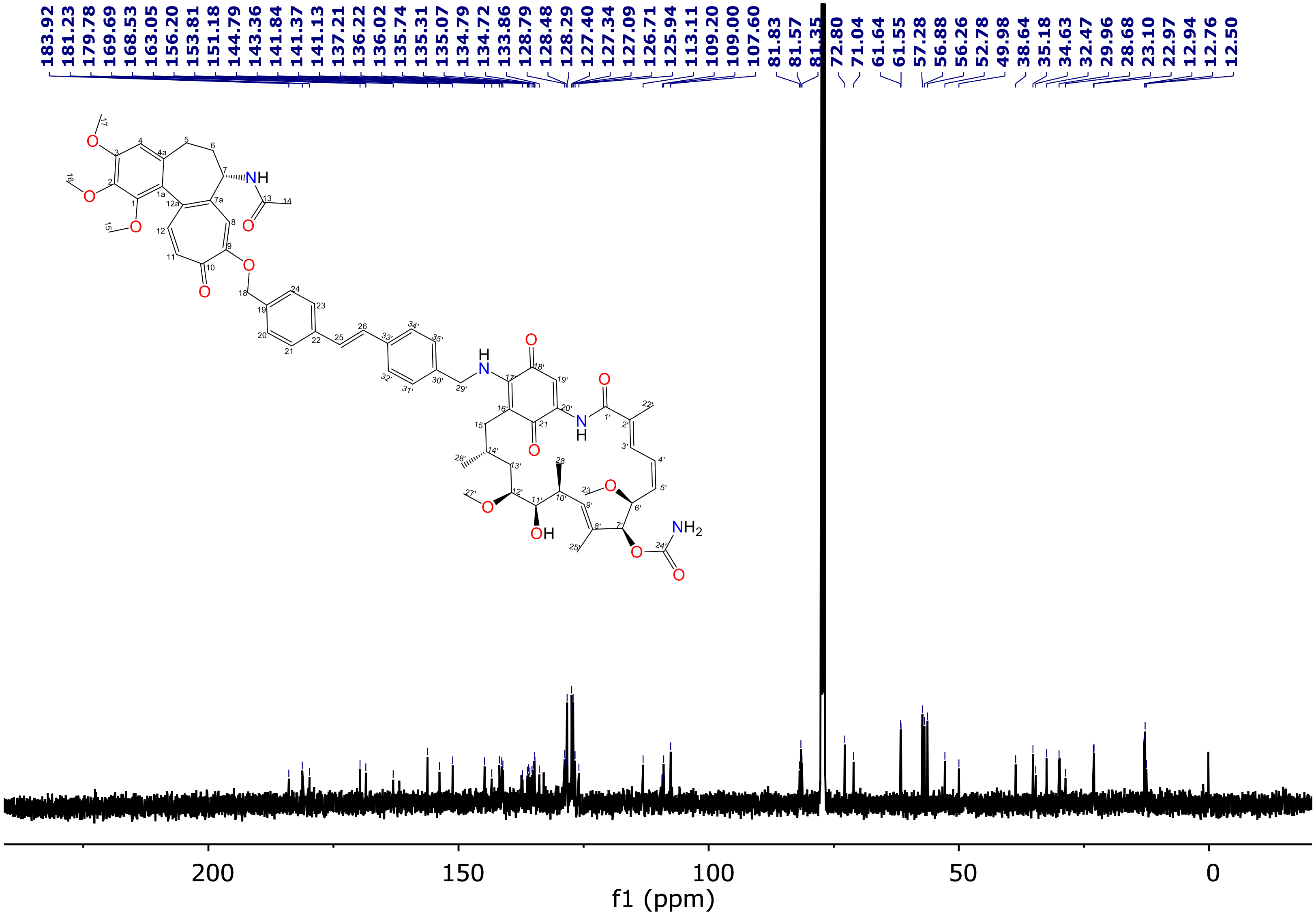

Figure 123S. ^{13}C NMR spectrum of compound **7d** in $CDCl_3$.

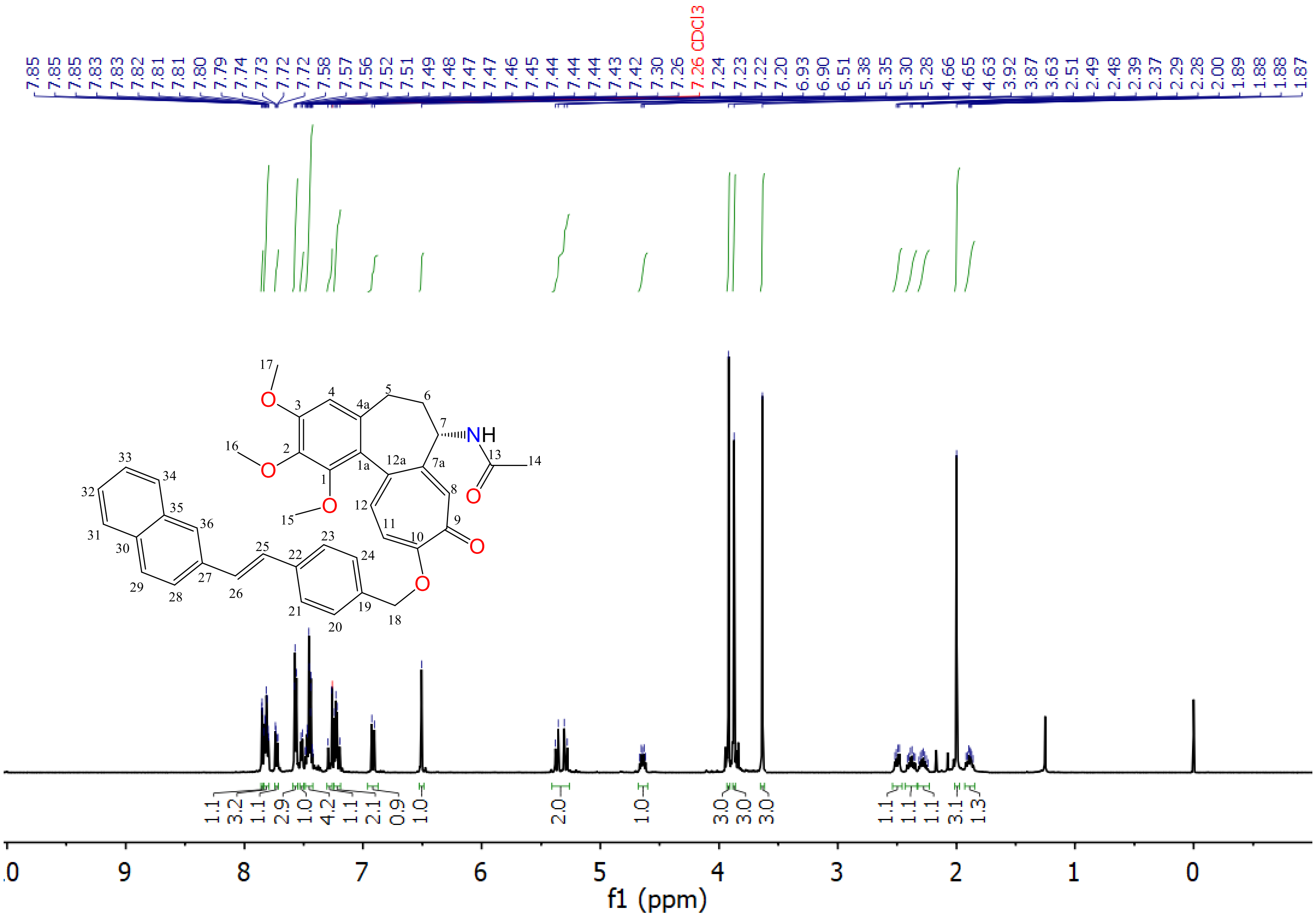

Figure 124S. ^{1}H NMR spectrum of compound **8a** in $CDCl_3$.

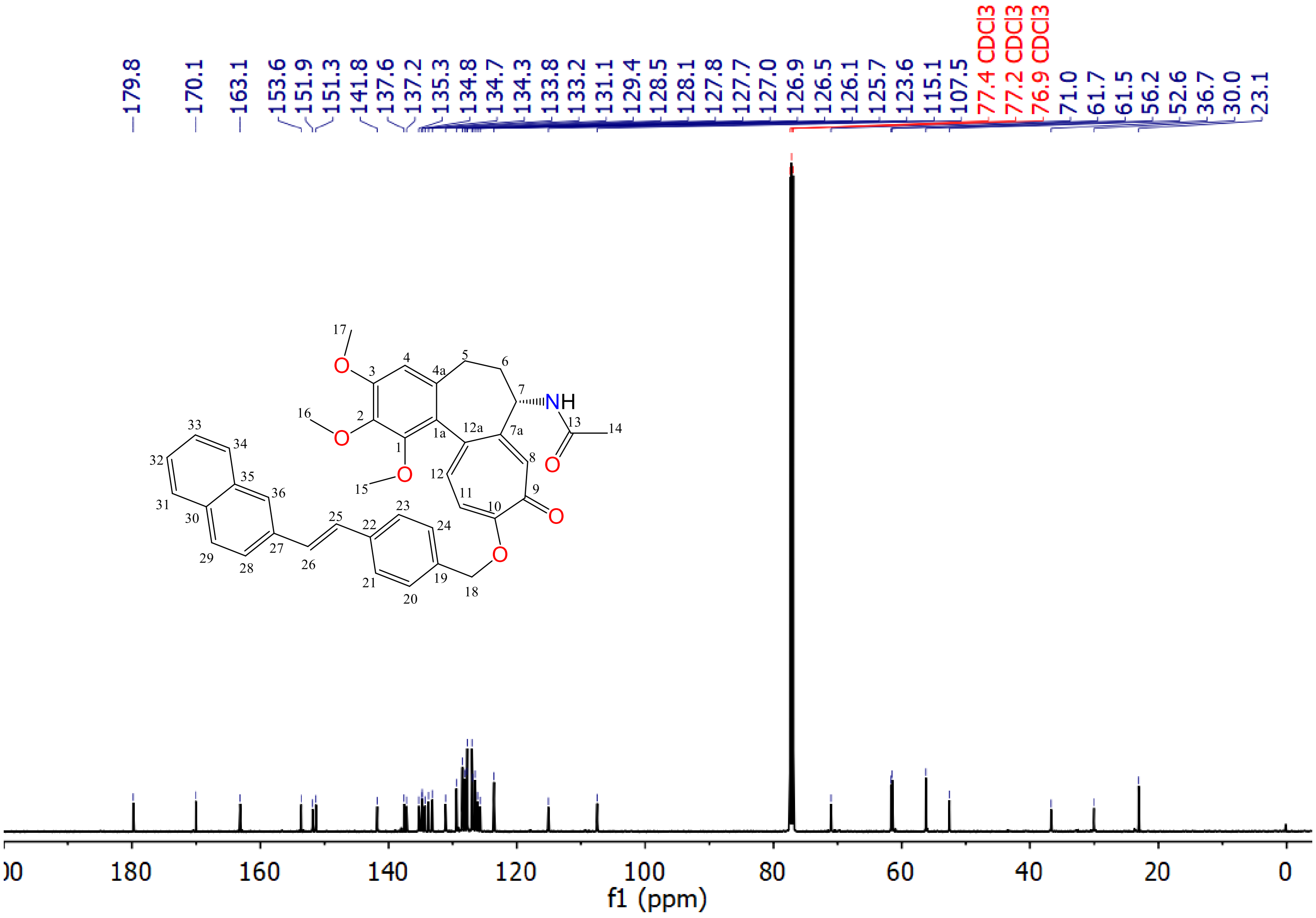

Figure 125S. ^{13}C NMR spectrum of compound **8a** in $CDCl_3$.

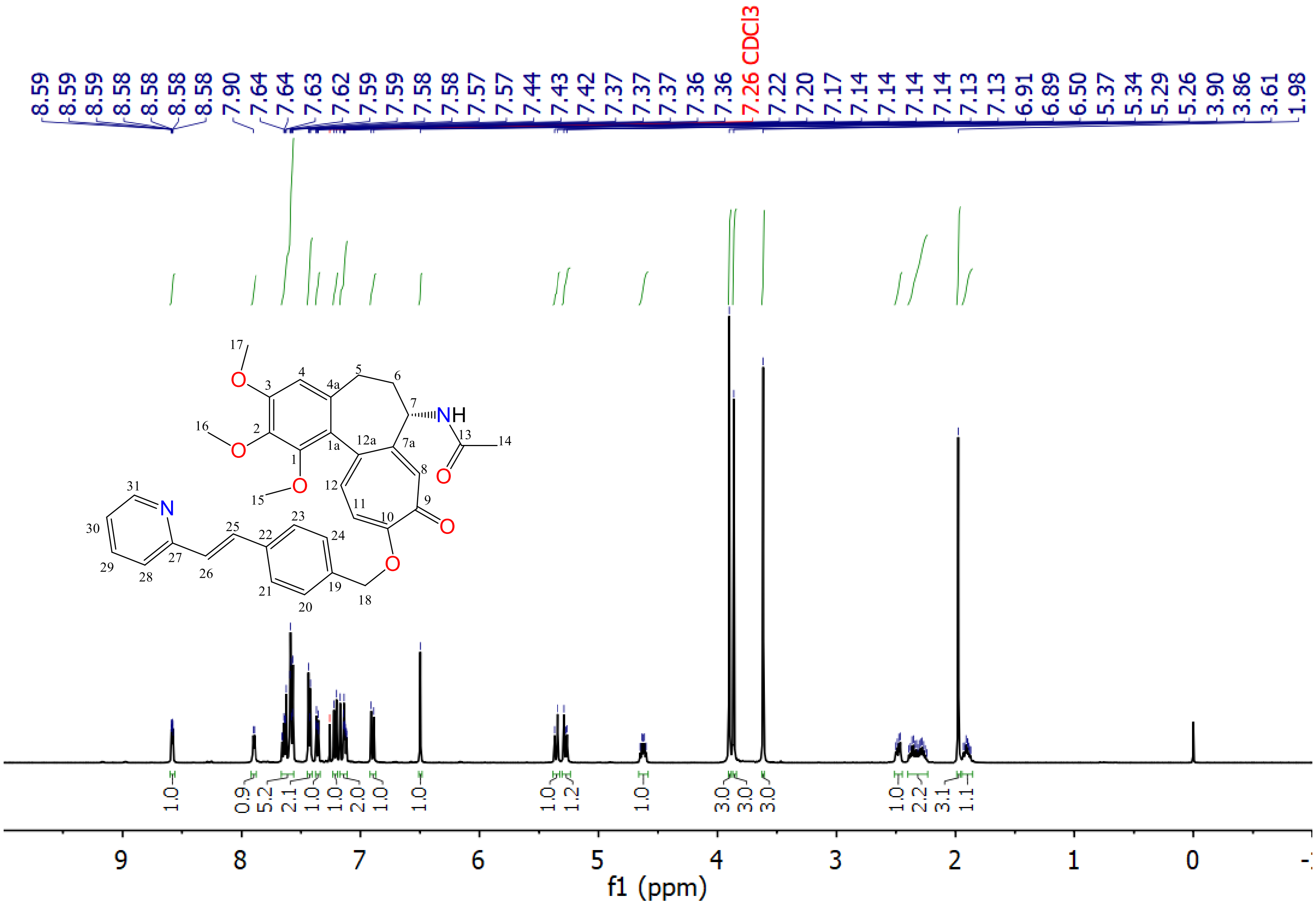

Figure 126S. ^{1}H NMR spectrum of compound **8b** in $CDCl_3$.

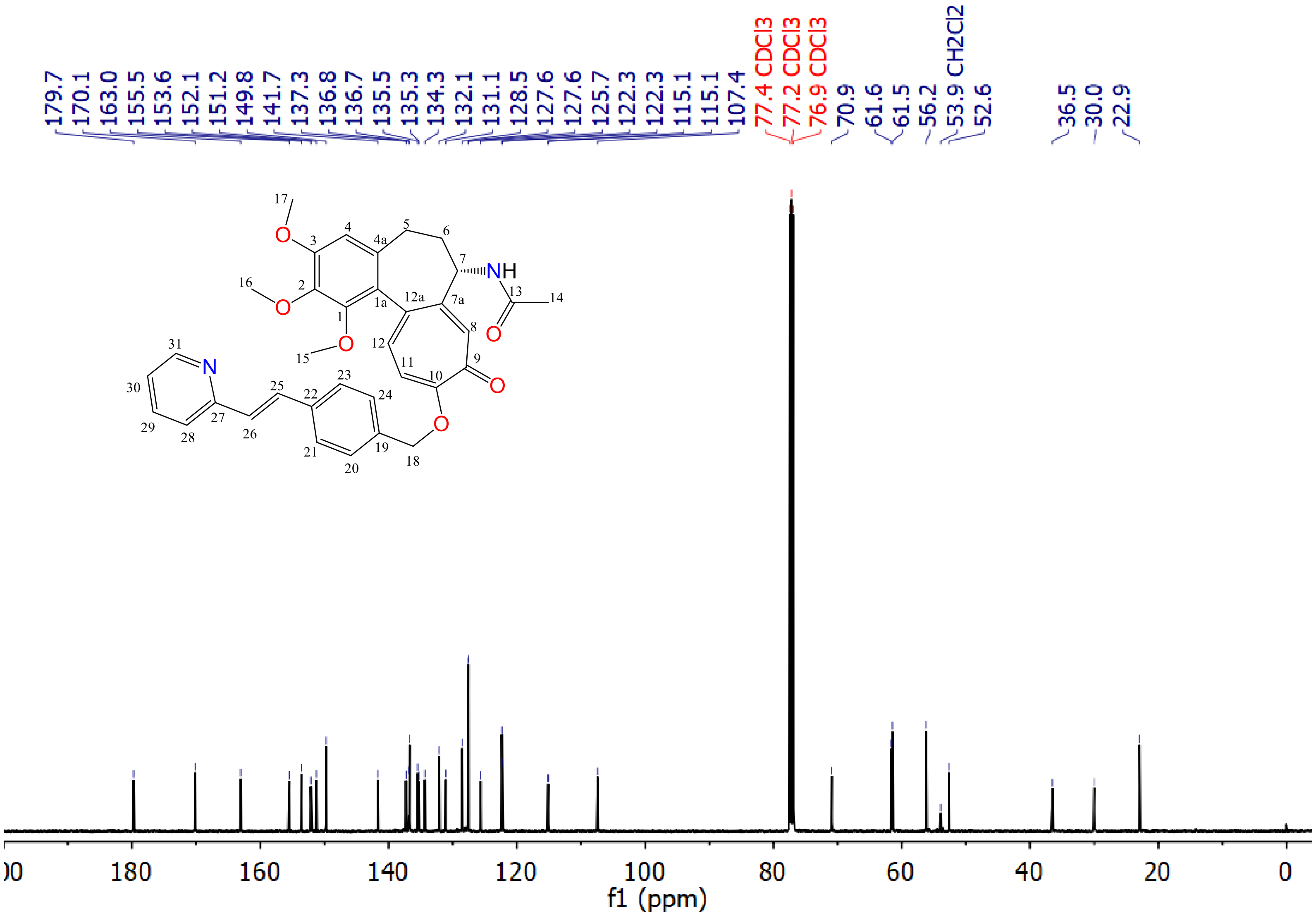

Figure 127S. ^{13}C NMR spectrum of compound **8b** in $CDCl_3$.

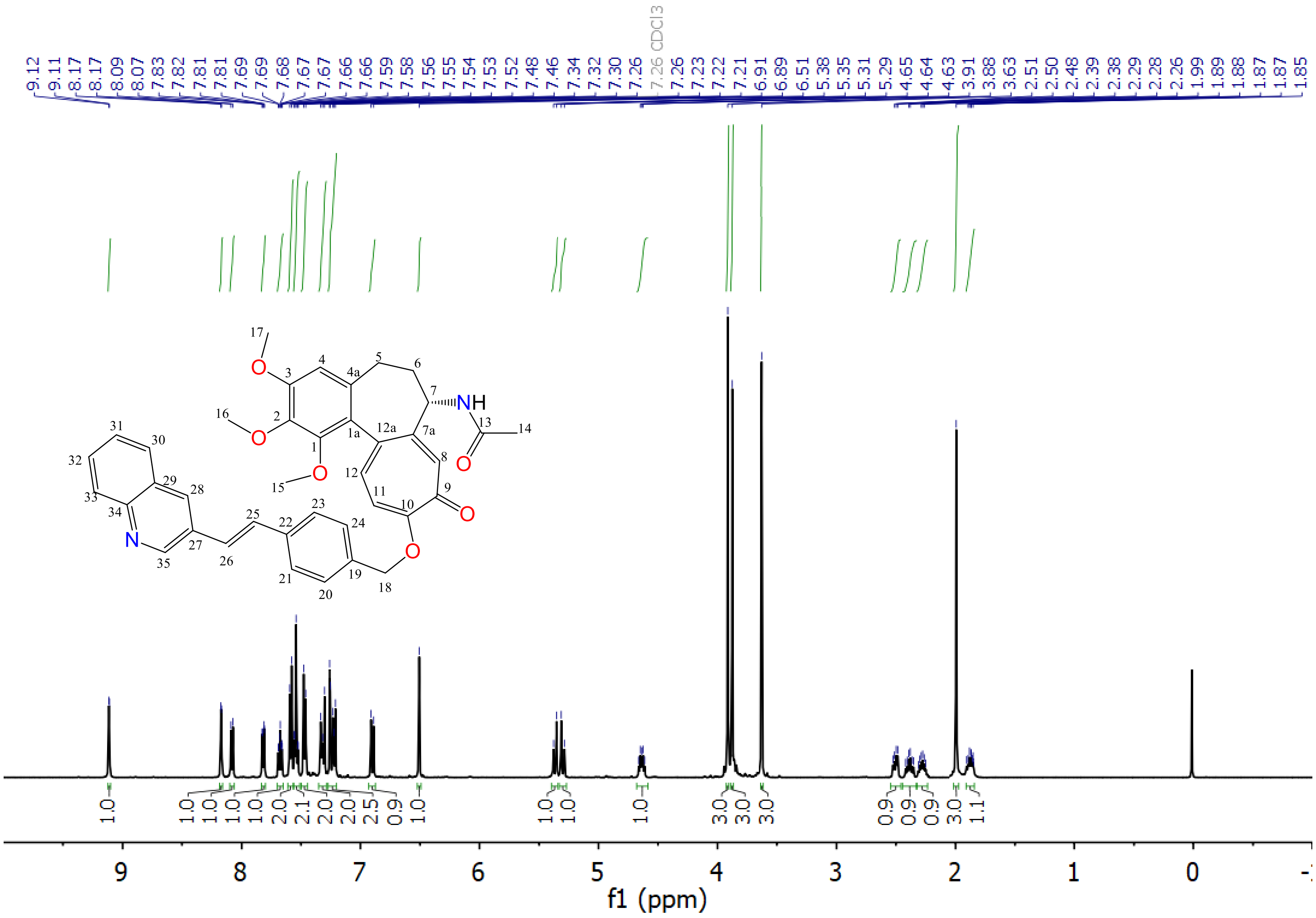

Figure 128S. ^{1}H NMR spectrum of compound **8c** in $CDCl_3$.

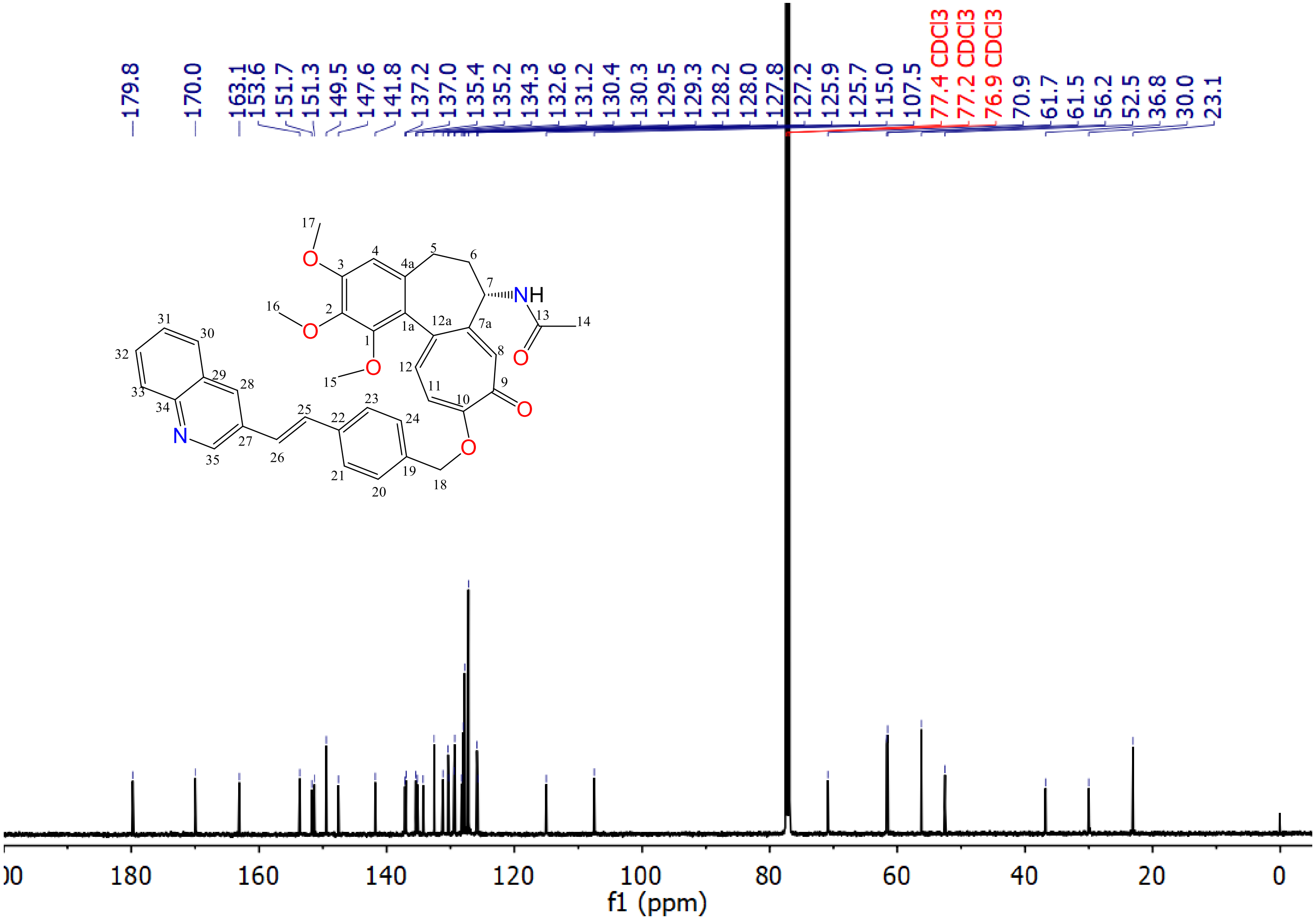

Figure 129S. ^{13}C NMR spectrum of compound **8c** in $CDCl_3$.

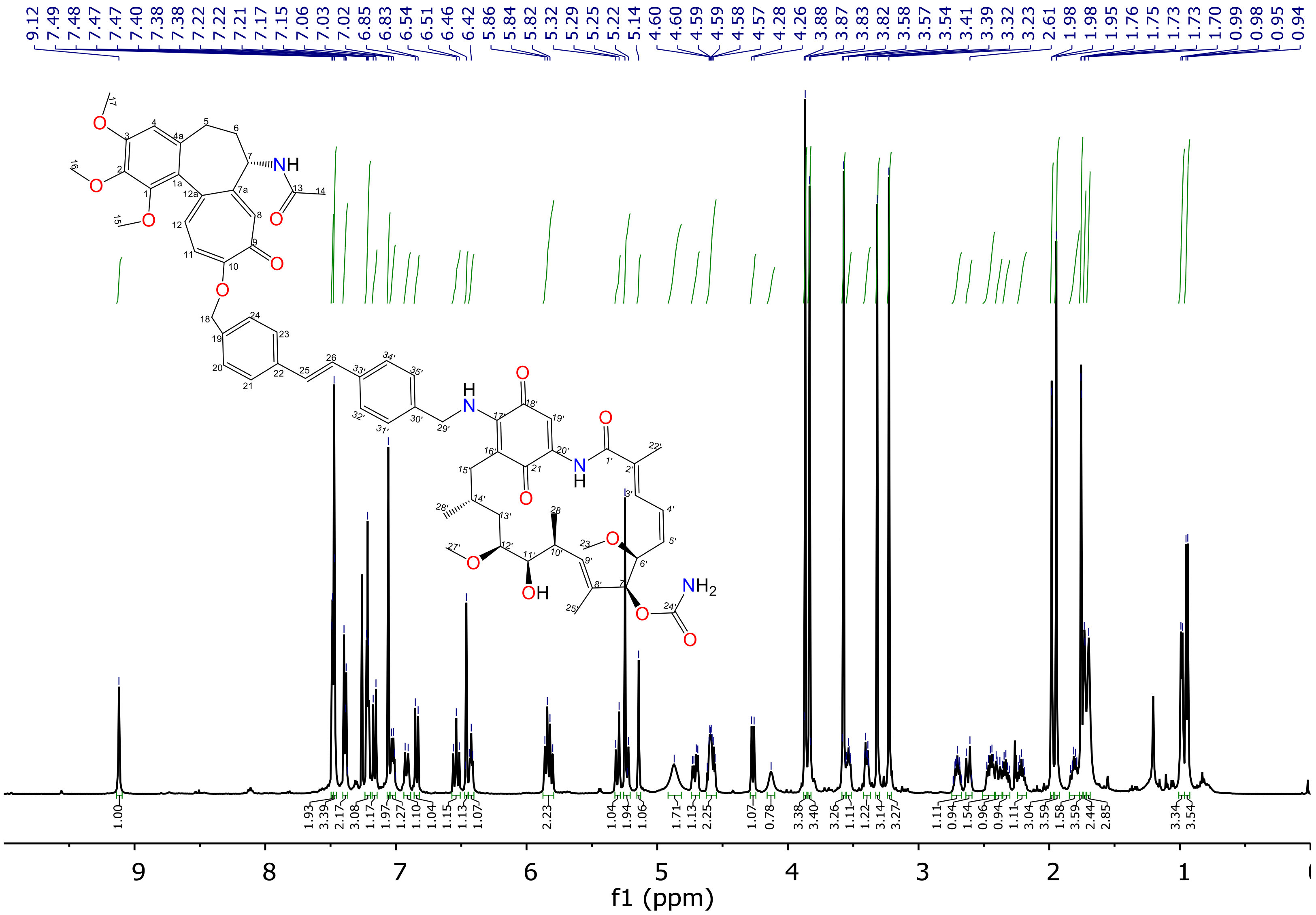

Figure 130S. ^{1}H NMR spectrum of compound **8d** in $CDCl_3$.

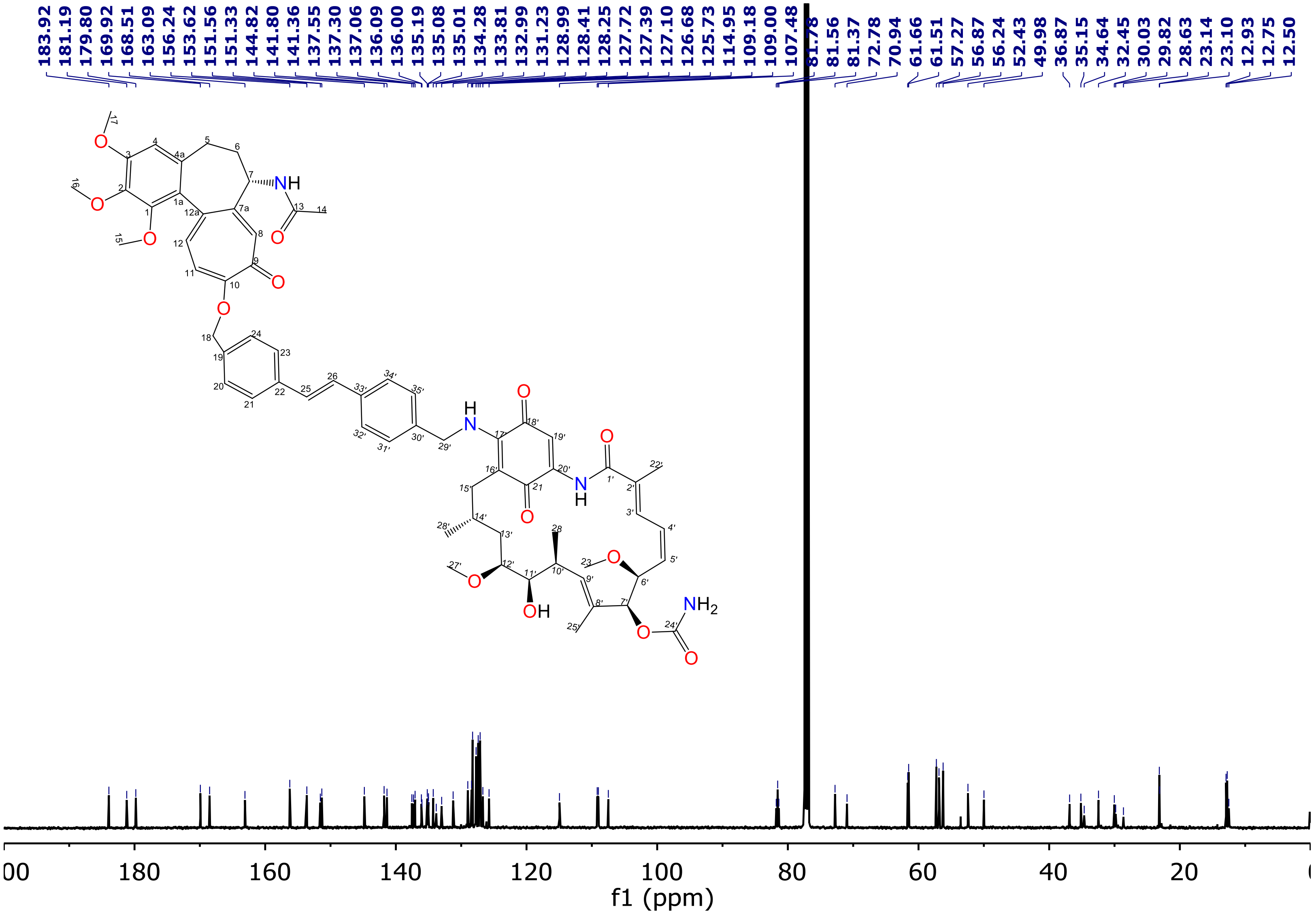

Figure 131S. ^{13}C NMR spectrum of compound **8d** in $CDCl_3$.

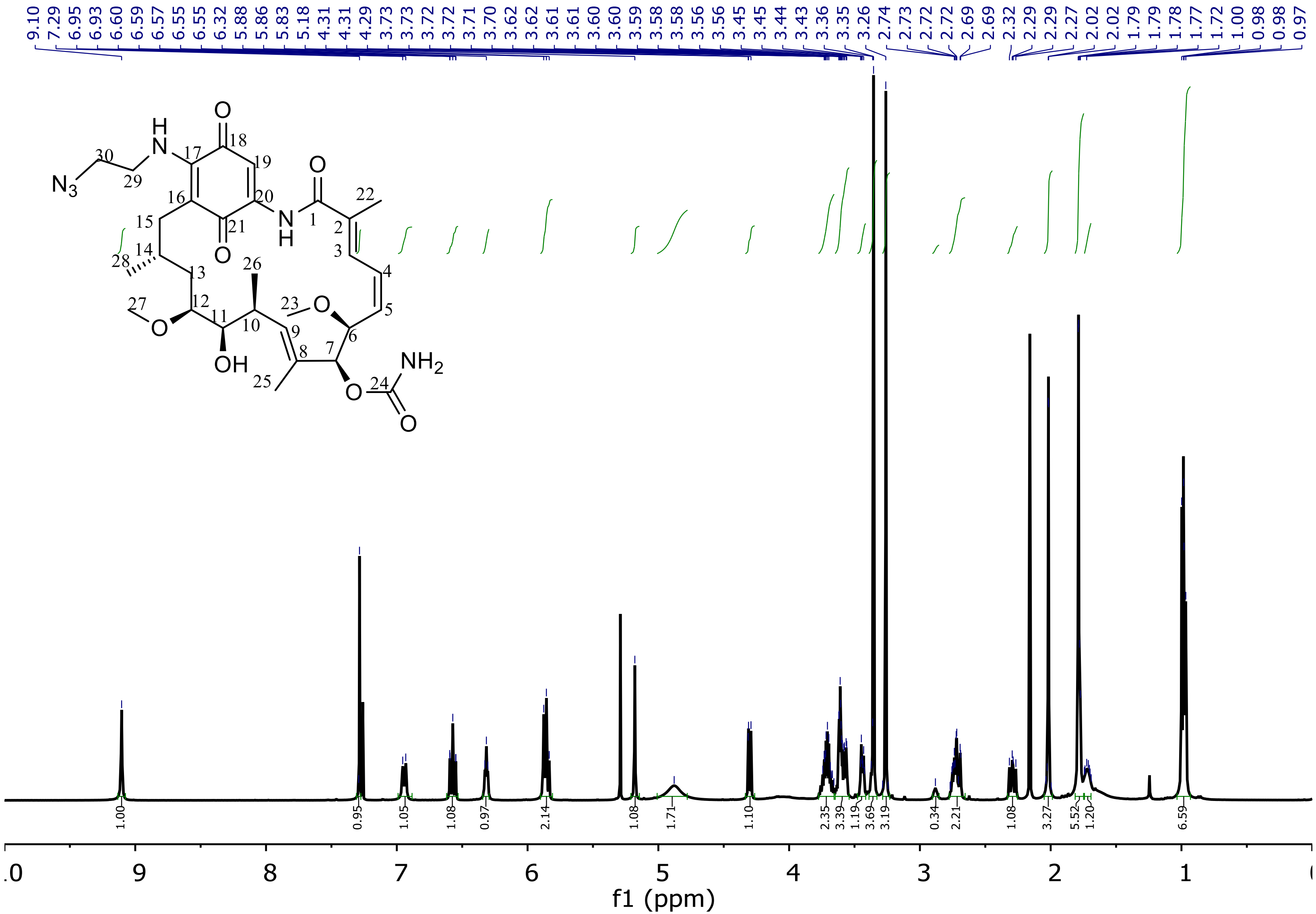

Figure 132S. ^{1}H NMR spectrum of compound **9** in $CDCl_3$.

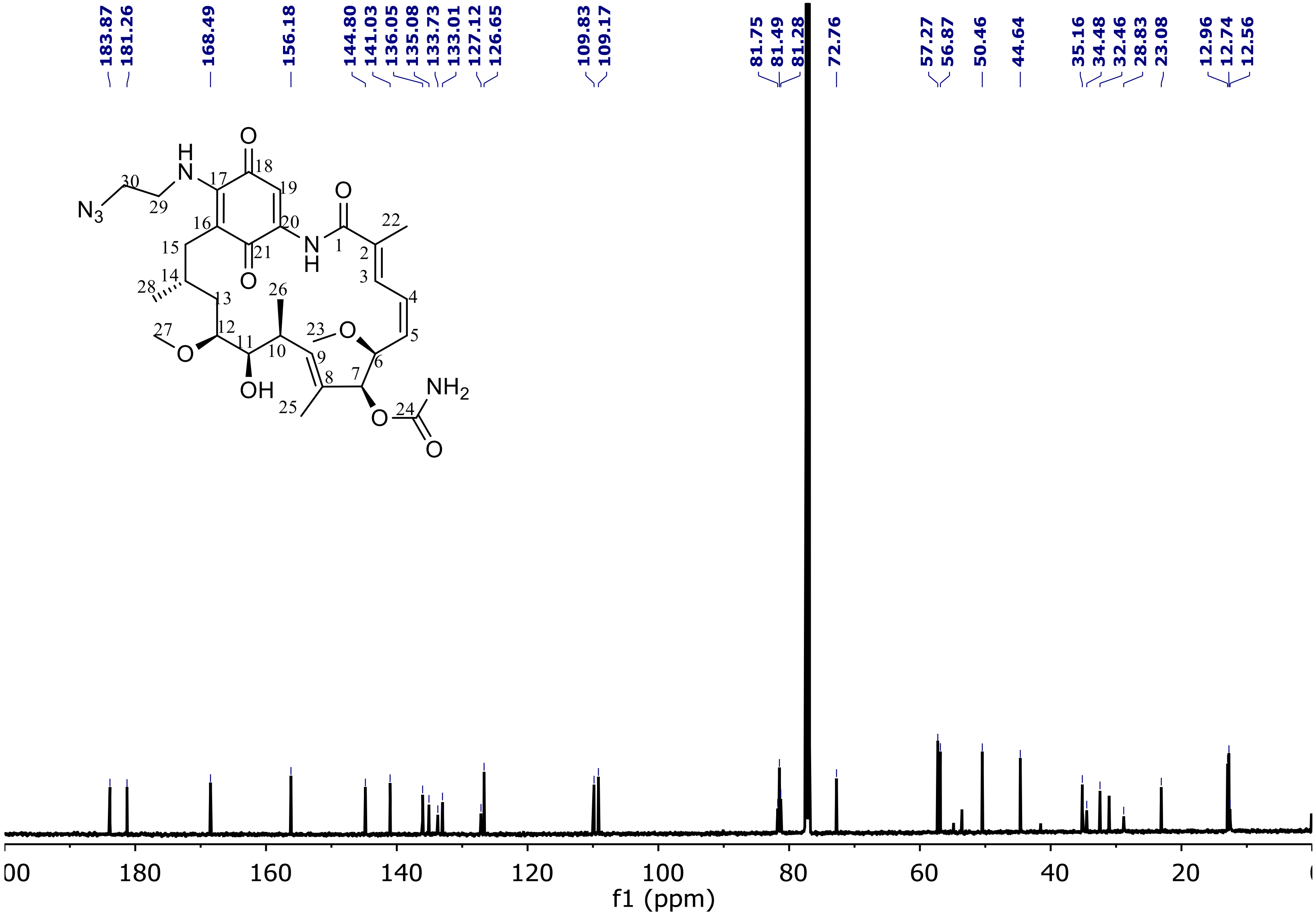

Figure 133S. ^{13}C NMR spectrum of compound **9** in $CDCl_3$.

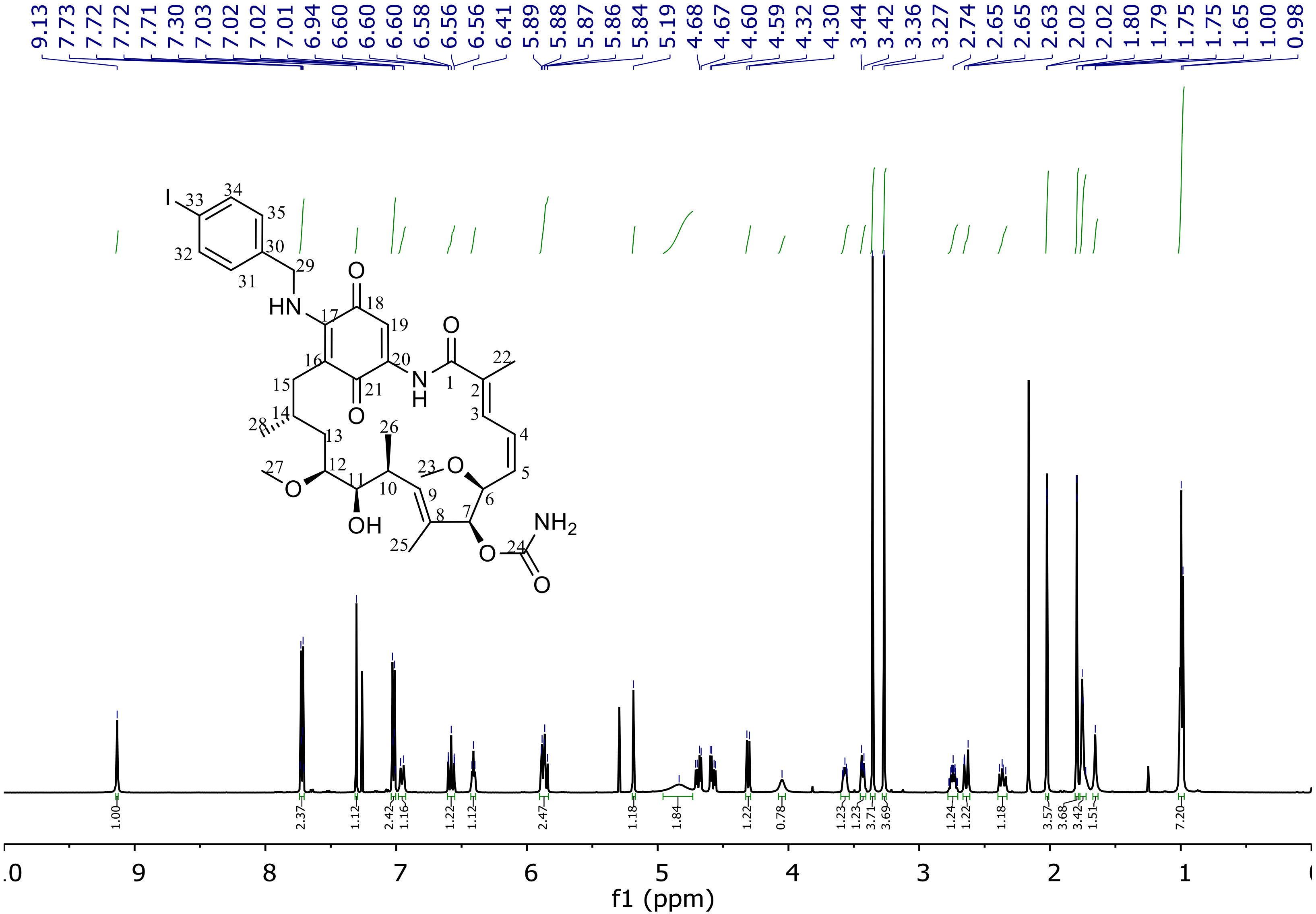

Figure 134S. ^{1}H NMR spectrum of compound **10** in $CDCl_3$.

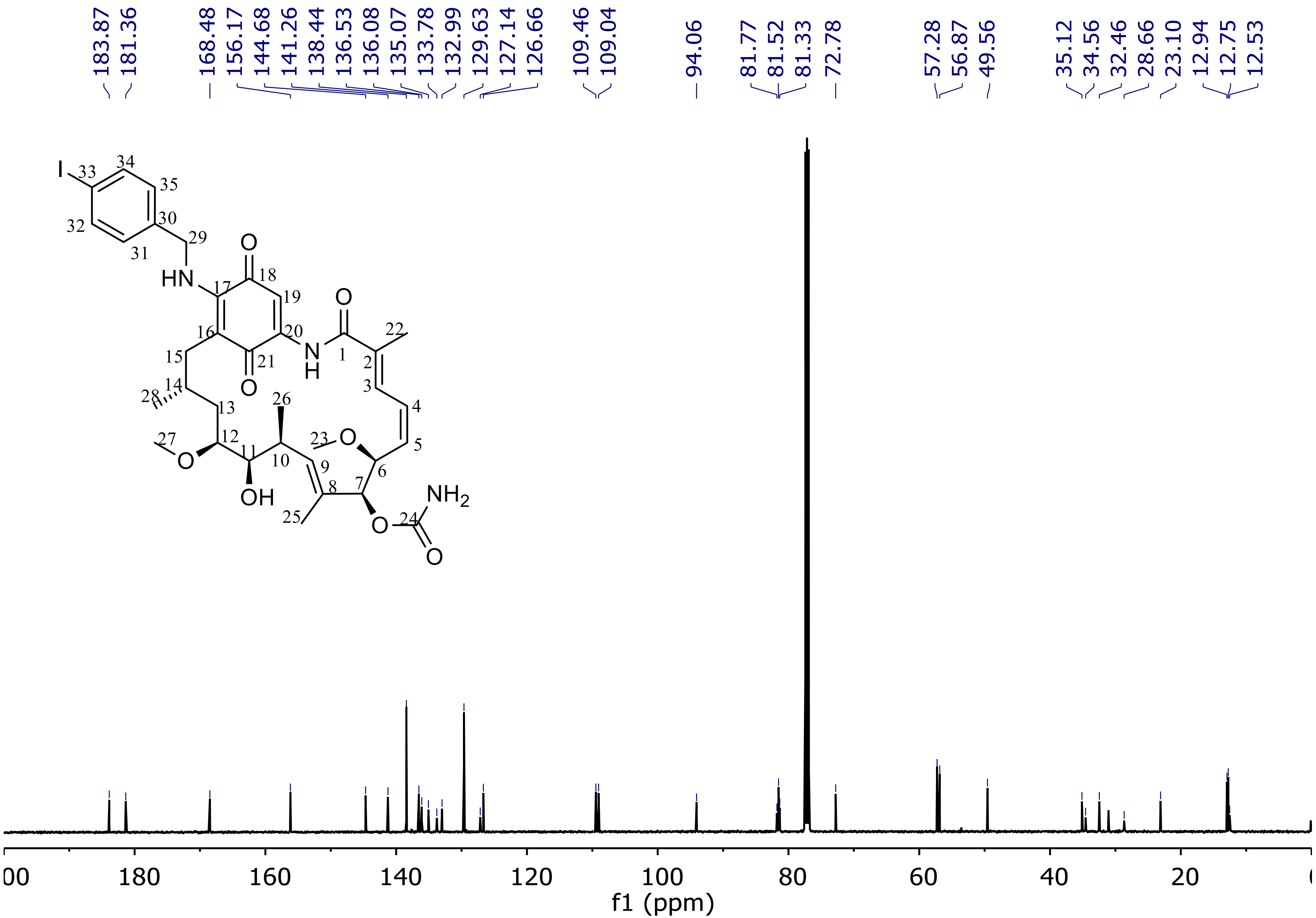

Figure 135S. ^{13}C NMR spectrum of compound **10** in $CDCl_3$.

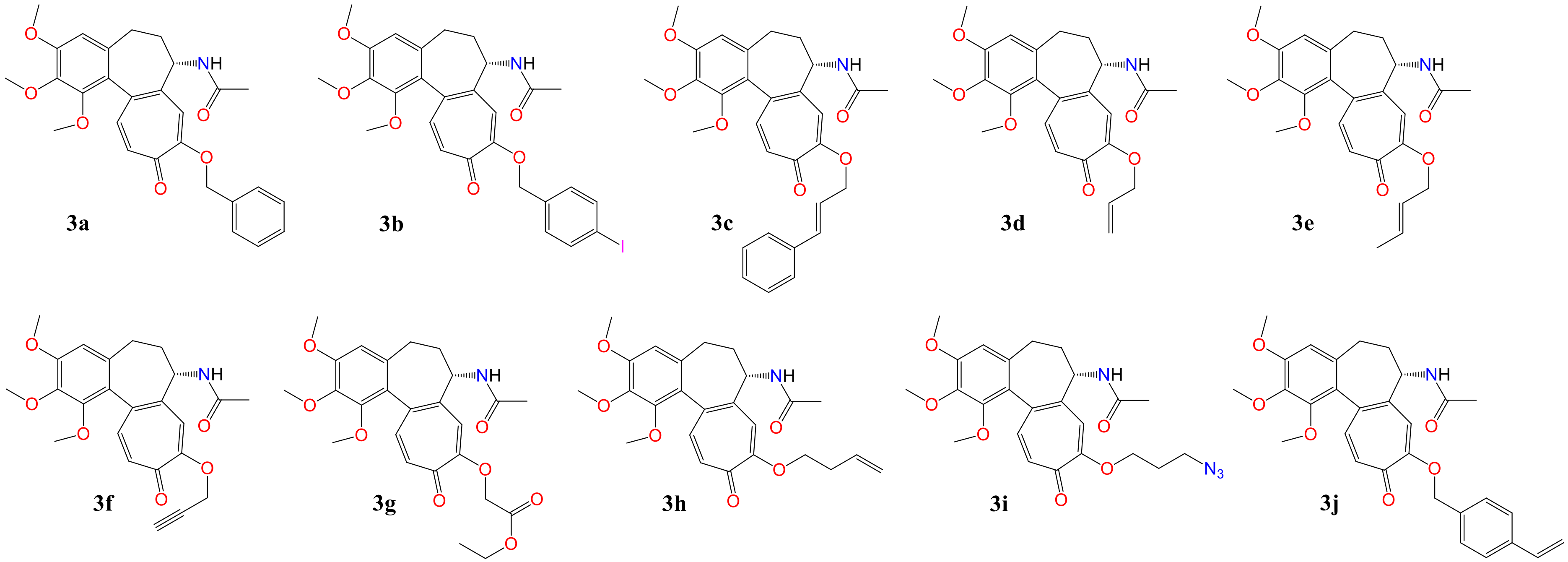

Figure 136S. Structures of all novel chemical entities **3a-3j** presented in ChemDraw 12.0 format.

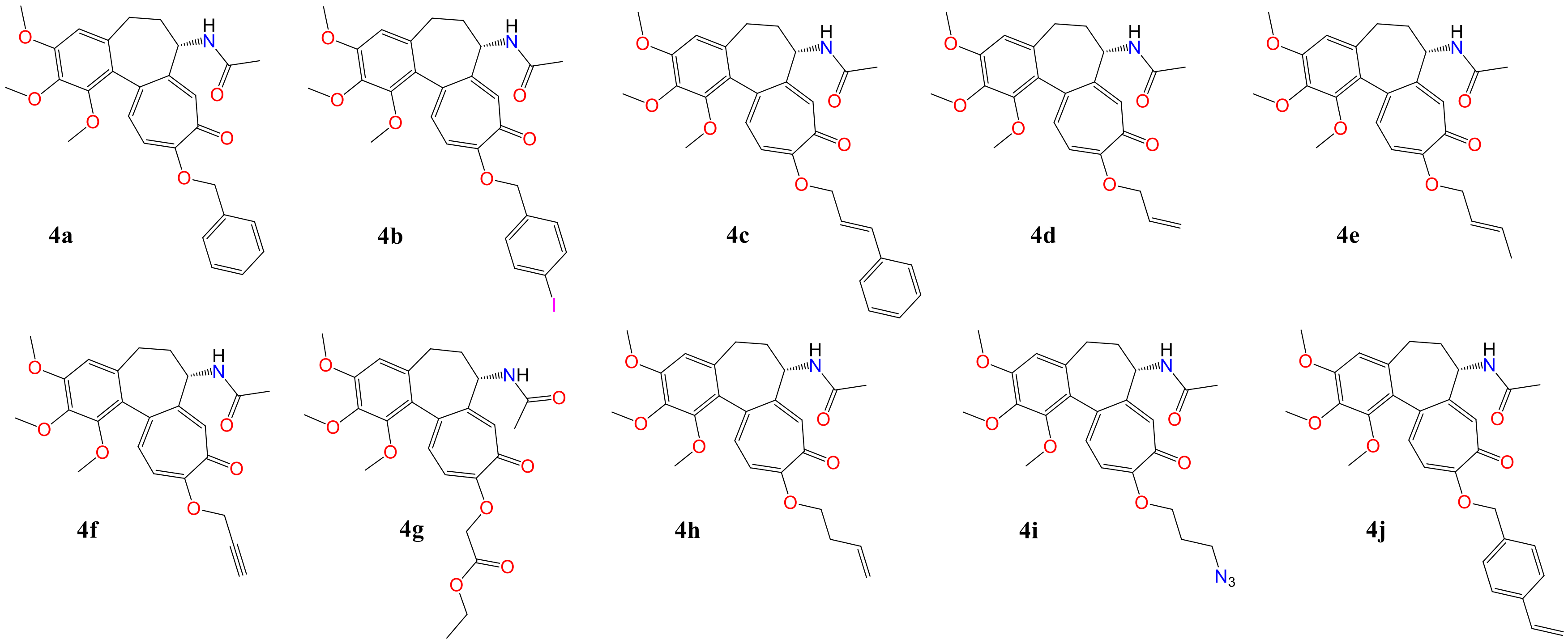

Figure 137S. Structures of all novel chemical entities **4a-4j** presented in ChemDraw 12.0 format.

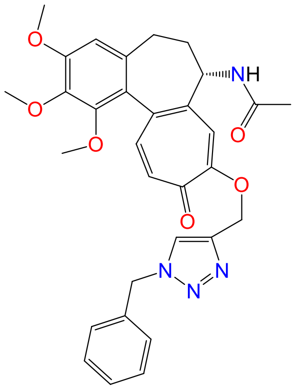

5a

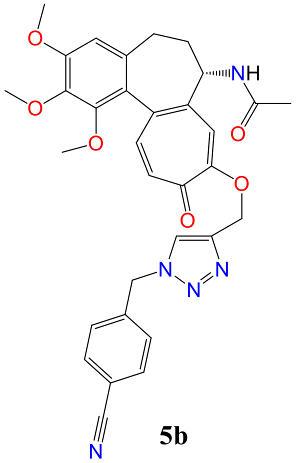

5b

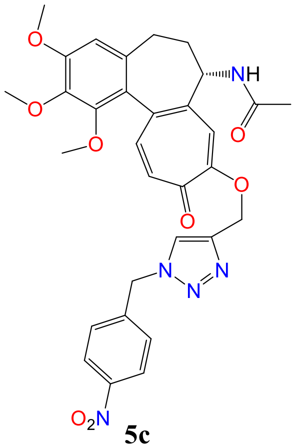

5c

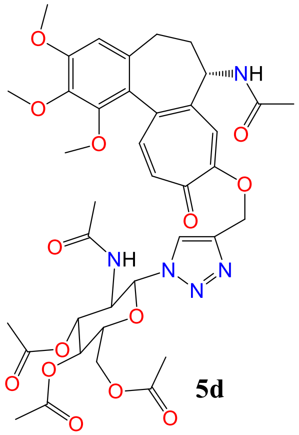

5d

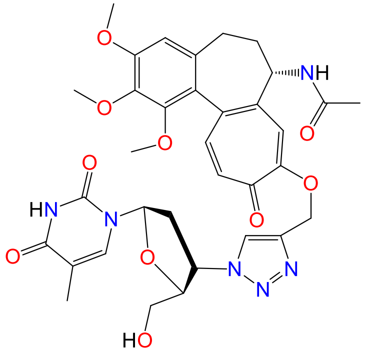

5e

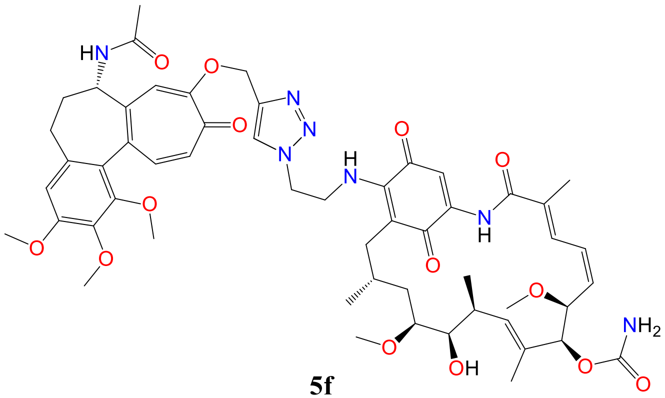

5f

Figure 138S. Structures of all novel chemical entities **5a-5f** presented in ChemDraw 12.0 format.

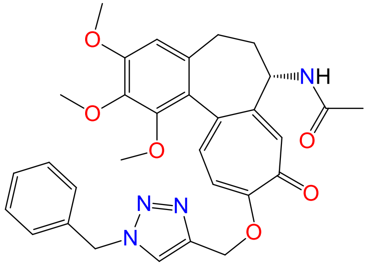

6a

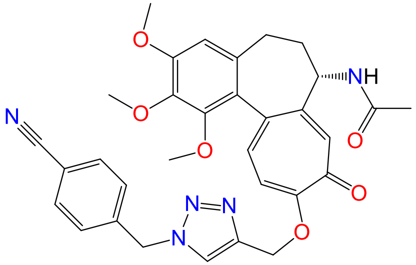

6b

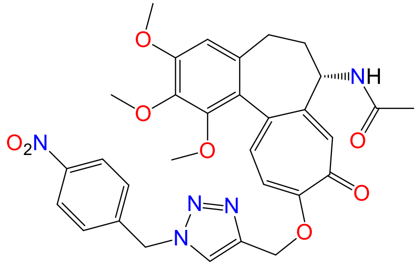

6c

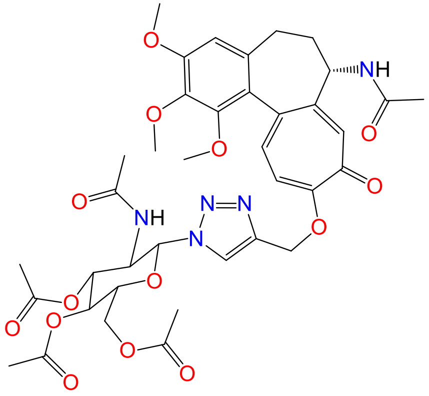

6d

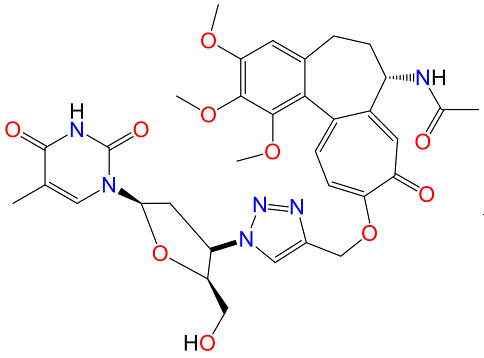

6e

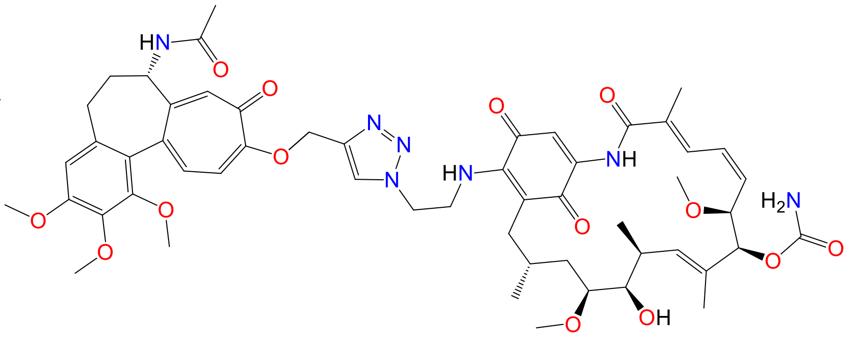

6f

Figure 139S. Structures of all novel chemical entities **6a-6f** presented in ChemDraw 12.0 format.

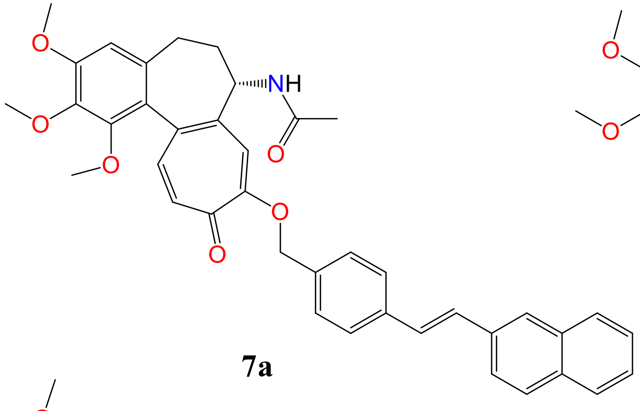

7a

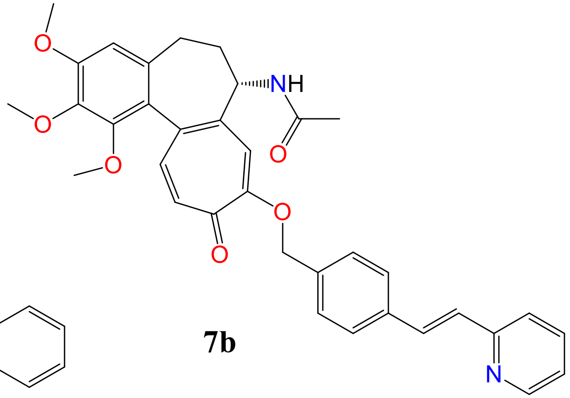

7b

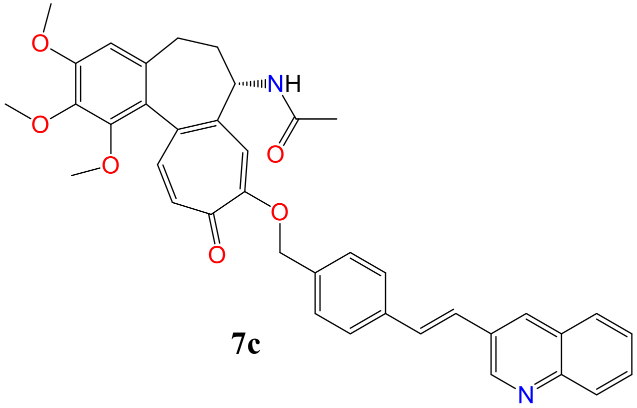

7c

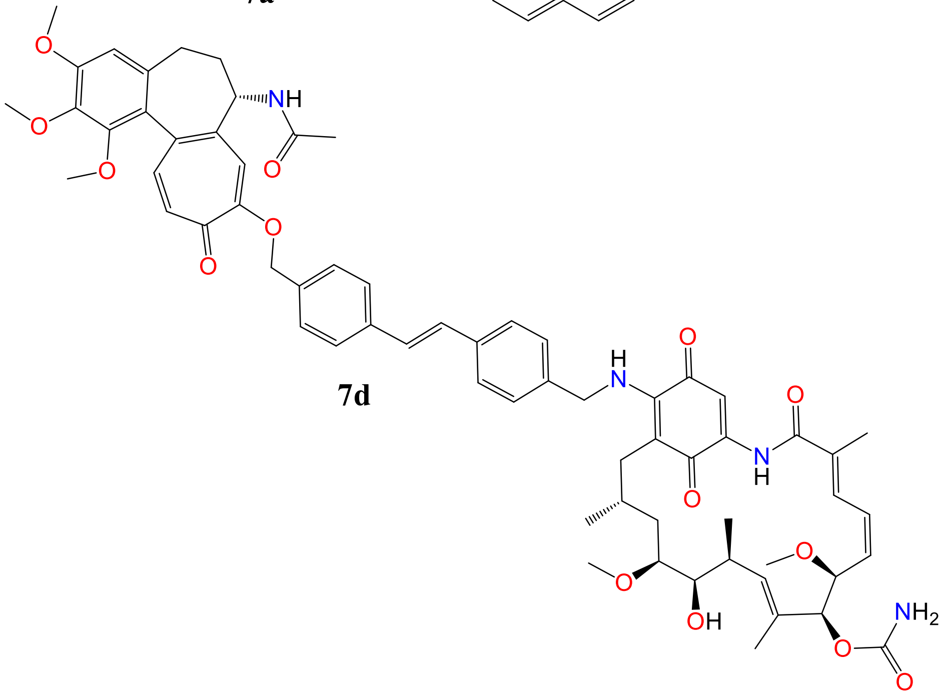

7d

Figure 140S. Structures of all novel chemical entities **7a-7d** presented in ChemDraw 12.0 format.

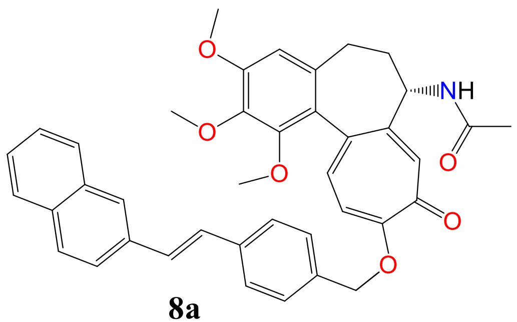

8a

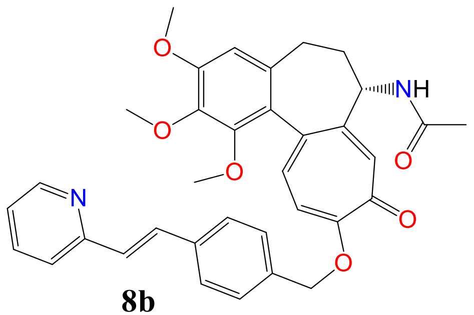

8b

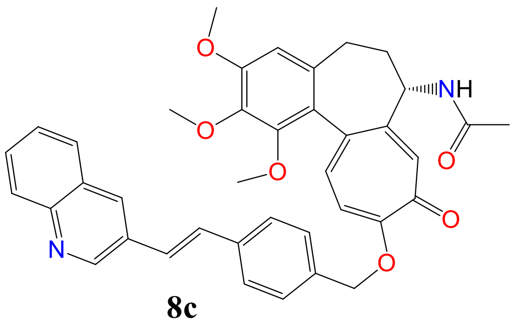

8c

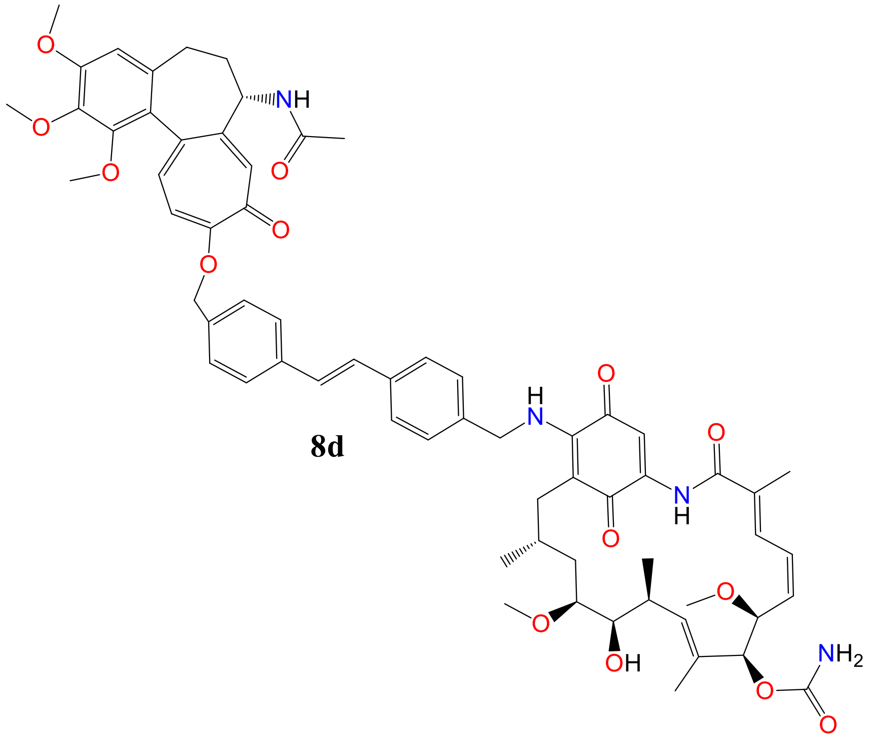

8d

Figure 141S. Structures of all novel chemical entities **8a-8d** presented in ChemDraw 12.0 format.

(1) Ravelli, R. B. G.; Gigant, B.; Curmi, P. A.; Jourdain, I.; Lachkar, S.; Sobel, A.; Knossow, M. Insight into Tubulin Regulation from a Complex with Colchicine and a Stathmin-like Domain. *Nature* **2004**, *428* (6979), 198–202. https://doi.org/10.1038/nature02393.
(2) *Scigress Package FJ 2.6 /EU 3.1.9./ 2008-2019*; Fujitsu, Japan.
(3) Cook, J. W.; Loudon, J. D. *The Alkaloids*; Academic Press: New York, NY, 1952; Vol. 2.
